# Clinical outcomes of hyaluronic acid–sorbitol injection in athletes with patellofemoral pain: Evaluation of the potential influence of body mass index

**DOI:** 10.1371/journal.pone.0346269

**Published:** 2026-09-30

**Authors:** Julio Carlos Vélez de Lachica, Susana Serrano, Eduard Vidovic, Antonio Farfan

**Affiliations:** 1 Social Security Institute of the State of Mexico and its Municipalities (ISSEMyM), Tlalnepantla, México; 2 Star Medica Tlalnepantla, Tlalnepantla, México; 3 Medical Department, Aptissen SA, Plan-les-Ouates, Switzerland; 4 Instituto Nacional de Rehabilitación, Ciudad de México, México; Shahrood University of Technology, IRAN, ISLAMIC REPUBLIC OF

## Abstract

**Background:**

Patellofemoral pain (PFP) is a frequent cause of activity-related knee pain and functional limitation in athletic populations. Elevated body mass index (BMI) may contribute to patellofemoral joint loading, but evidence on hyaluronic acid–sorbitol injection outcomes in athletes with elevated BMI remains limited.

**Objective:**

To describe longitudinal changes in knee function and symptoms over 6 months after intra-articular hyaluronic acid–sorbitol injection in athletes with PFP, and to explore whether baseline BMI was associated with post-injection improvement.

**Methods:**

This prospective, single-arm, non-randomized interventional study included 60 athletes aged 30–55 years who received a single 4 mL intra-articular hyaluronic acid–sorbitol injection. IKDC and KOOS scores were assessed at baseline, 15 days, 3 months, and 6 months. Longitudinal changes were evaluated using linear mixed models, with generalized estimating equations as a sensitivity analysis; exploratory regression and subgroup analyses assessed the association between BMI and improvement.

**Results:**

Mean IKDC and KOOS scores increased from baseline to 6 months, with estimated changes of +18.58 points for IKDC (95% CI 16.81–20.36) and +19.28 points for KOOS (95% CI 17.52–21.04; both p < 0.001). At 6 months, 75.0% of participants reached the predefined IKDC threshold for clinically meaningful improvement and 83.3% reached the KOOS threshold. Participants with BMI ≥ 25 kg/m^2^ also showed within-group improvement. BMI was not independently associated with improvement rate after adjustment for baseline score; however, the small elevated-BMI subgroup (*n* = 22) limited these exploratory comparisons, and a clinically relevant difference could not be excluded. Minor local adverse events occurred in 4 of 60 participants and resolved with cryotherapy.

**Conclusion:**

Athletes with PFP, including those with elevated BMI, showed consistent improvement in knee function and symptoms over 6 months following hyaluronic acid–sorbitol injection. These findings support intra-articular hyaluronic acid–sorbitol as a feasible, well-tolerated option for athletes with PFP, regardless of body weight. Controlled trials are needed to confirm the treatment’s contribution, given the single-arm design.

**Trial Registration:**

CEI/0504/PI/2022; ClinicalTrials.gov NCT05450458.

## Introduction

Over the past few decades, a high incidence of knee pain has been documented secondary to new high-intensity sports trends characterized by longer durations and increased joint loading [1–4]. Improper training methods and suboptimal equipment also contribute to these disorders. Athletes who push their physical limits are particularly prone to overuse-related anterior knee pain [5–10]. Consequently, patellofemoral pain (PFP) is commonly associated with chronic, repetitive sports injuries [11–13]. This condition arises when repetitive microtrauma accumulates without adequate recovery, typically because knee loading exceeds the mechanical tolerance of the affected tissues [14]. Such injuries are a leading cause of attrition among elite athletes and substantially limit performance and training continuity. A high body mass index often coexists with increased body fat and reduced lean mass, which impairs isometric hip and knee strength and thereby increases both the risk and severity of patellofemoral pain in athletes [15]. Their debilitating nature frequently forces athletes to reduce or suspend competitive participation. Consequently, prevention strategies are increasingly critical in high-performance athletic populations [16].

Accurate diagnosis of PFP relies primarily on a thorough clinical history and physical examination, with symptoms correlated to the specific sport or activity involved; imaging is reserved for excluding alternative diagnoses rather than establishing PFP itself [5,8]. Currently, there are no established treatment guidelines for most PFP-related conditions in athletes [7,17]. Consequently, initial management typically focuses on conservative approaches including non-steroidal anti-inflammatory drugs (NSAIDs), reducing mechanical overload, correcting gait abnormalities, improving training techniques, incorporating strengthening and stretching exercises, and optimizing footwear [18]. Furthermore, physical therapy modalities such as cryotherapy, low-level laser therapy, therapeutic ultrasound, and electrotherapy are commonly used as first-line treatments, particularly for tissue repair and inflammation reduction [19–21].

Evidence supporting the use of hyaluronic acid (HA) in athletes remains limited [22,23]. While HA is well-documented for osteoarthritis (OA), its efficacy in sports-related overuse conditions remains unproven [24,25]. Similarly, conclusive studies have not demonstrated that HA combined with sorbitol, a proposed antioxidant additive, accelerates pain relief or prolongs therapeutic effects in this population [26]. To date insufficient data exist on HA–sorbitol and antioxidative formulations in athletes with elevated body mass index, despite the biomechanical impact of increased weight on knee joints [27,28]. In patients with high mechanical joint loading or obesity, oxidative stress has been implicated as part of the underlying pathophysiology [29–32], and HA–sorbitol combines viscoelastic and proposed antioxidant properties that may be biologically relevant in this context, although clinical evidence remains preliminary.

This prospective, single-arm, non-randomized interventional study aims to describe longitudinal within-participant changes in IKDC and KOOS scores during 6 months of follow-up after HA–sorbitol injection in athletes with PFP. As a secondary exploratory objective, we examined whether athletes with elevated BMI (≥25 kg/m^2^) — including overweight and obesity — also showed clinically relevant within-participant improvements while maintaining usual sport activity. Because elevated BMI increases patellofemoral joint loading, this subgroup was of particular interest; although oxidative stress has been proposed as a contributing mechanism in this context, it was not directly assessed in the present study, and findings are intended to be hypothesis-generating and to inform future controlled trials.

## Methods

### Study design and ethics

This prospective, single-arm, non-randomized interventional study was designed and reported in accordance with the TREND (Transparent Reporting of Evaluations with Nonrandomized Designs) guidelines for non-randomized interventional studies. The study was conducted at the ISSEMyM Tlalnepantla Orthopedics Department after obtaining ethical approval (CEI/0504/PI/2022), and all participants provided written informed consent. The study was registered at ClinicalTrials.gov (NCT05450458), and the authors confirm that all ongoing and related trials for this intervention are registered. Participant recruitment and enrollment took place between 1 February 2023 and 30 October 2024, with final follow-up completed on 30 April 2025.

### Participants

Initially, 64 consecutive athletes (39 women and 25 men) with a clinical diagnosis of PFP were enrolled. PFP was diagnosed clinically based on anterior or retro-patellar pain aggravated by activities that load the patellofemoral joint (stairs, squatting, running, prolonged sitting). Imaging (ultrasound/X-ray) was used only to exclude alternative diagnoses, not to establish PFP. Symptom behavior was documented, including pain during stair ascent/descent, squatting, running, and prolonged sitting. Pain severity, symptom duration, and unilateral/bilateral involvement were recorded according to REPORT-PFP recommendations [33]. This study focused on adult athletes aged 30–55 years, who frequently present with activity-related anterior knee pain in sports-medicine practice and often present with elevated BMI, which increases joint loading and is therefore relevant to the study aims.

Four participants were excluded during follow-up: two failed to attend any post-baseline evaluation, one sustained a meniscal injury, and one discontinued due to high-risk pregnancy (Fig 1). The final complete-case analysis included 60 participants who completed the single injection and all four assessment points (baseline, 15 days, 3 months, 6 months). No missing IKDC/KOOS values were present among these 60 completers; the target of 64 accounted for ~10% anticipated attrition.

**Fig 1:**
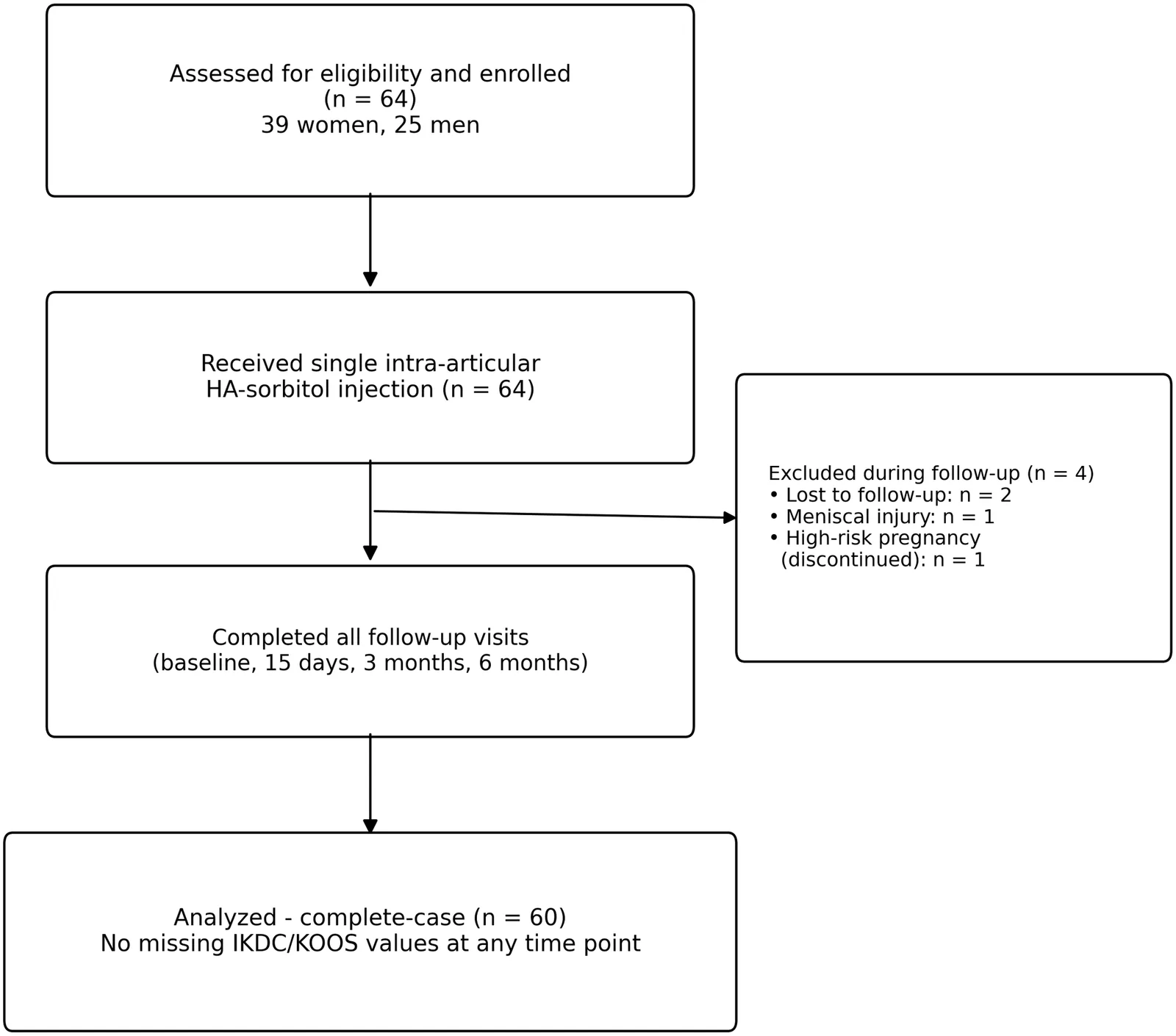
Participant flow diagram. Sixty-four consecutive athletes with a clinical diagnosis of patellofemoral pain were enrolled and received a single intra-articular hyaluronic acid–sorbitol injection. Four participants were excluded during follow-up, 60 participants completed all four assessment points and were included in the complete-case analysis.

Eligibility criteria included age 30–55 years and engagement in moderate- to high-impact knee-loading sport at least three times per week, with sessions lasting a minimum of one hour. Athlete was therefore defined broadly as active adults meeting this frequency/duration threshold, and competitive level was further classified by the Tegner activity scale as professional [9,10], amateur [7,8], or recreational [5,6,34,35]. This broad definition includes recreational athletes and limits generalizability to elite competitive populations; external validity is discussed in Limitations. Additional eligibility required no history of surgical intervention, ligament or meniscal injury, acute or chronic infection, or lower-limb sequelae, and ability to complete 6-month follow-up without NSAID use and with full adherence to protocol.

A minimum sample size of 58 was determined a priori, assuming an expected responder proportion of ~75% based on the author’s prior clinical experience with this patient population, and a two-sided 95% confidence interval half-width of ±11%. Sixty-four participants were enrolled to allow for anticipated attrition; 60 completed follow-up and were analyzed.

### Procedures

After enrollment, each participant received a single sport-specific injury-prevention education session delivered by a physiotherapist, aimed at reducing the risk of secondary soft-tissue injury during the follow-up period; no other physiotherapy, exercise, bracing, or pharmacological intervention was provided beyond the study injection.

All participants received a single intra-articular sub-patellar injection of HA–sorbitol (4 mL, 80/160; Synolis VA®, Aptissen, Switzerland), administered by a single orthopedic surgeon (principal investigator). With the knee kept fully extended, the injection site was cleaned with chlorhexidine. A 21-gauge, 1.5-inch needle was inserted just below the patella; aspiration confirmed intra-articular placement and absence of vascular entry before slow, steady injection. Light pressure was applied after withdrawal. Participant characteristics were recorded with a standardized form, including sport type and Tegner level [34,35]. Knee function and pain were assessed with IKDC and KOOS at baseline (day of injection), 15 days, 3 months, and 6 months. Adverse events (pain, swelling, erythema) were monitored at each visit [36]. Follow-up adherence was reinforced with scheduled phone/email reminders.

### Statistical analysis

All analyses were performed on patient-level data. IKDC and KOOS were analyzed on a 0–100 scale; source values recorded on a 0–1 scale were multiplied by 100 before analysis. The KOOS aggregate was the mean of the five subscales (Pain, Symptoms, Activities of Daily Living, Sport/Recreation, Quality of Life). Continuous variables are presented as mean (SD); 95% CIs were calculated with the t-distribution. Categorical variables are n (%). Missing-data handling was complete-case analysis without imputation, as no IKDC/KOOS values were missing among the 60 completers.

Primary repeated-measures analysis used a linear mixed model (LMM) with random intercept for participant and categorical time (baseline as reference; 15 days, 3 months, 6 months), estimated by restricted maximum likelihood (REML). Mean changes vs baseline with 95% CIs and overall time effect (likelihood-ratio χ² vs intercept-only model) were derived from the LMM. Generalized estimating equations (GEE) with exchangeable working correlation and robust standard errors served as sensitivity analysis. Descriptive paired changes (mean change, SD change, 95% CI, paired t-test, exact p, and Cohen’s dz) are also reported for comparability. Cohen’s dz (mean within-participant change/ SD of the change) is a descriptive standardized measure of within-participant change that should not be interpreted as a causal treatment effect in this single-arm study, as it may be influenced by regression to the mean, natural recovery, and selection.

Clinically meaningful improvement at 6 months was a secondary responder analysis using a priori thresholds of ≥11.5 points for IKDC and ≥10 points for the KOOS aggregate used in this study. The IKDC threshold was based on published MCID estimates in PFP populations [37]. The KOOS threshold of ≥10 points was defined a priori as an approximation, consistent with published PFP subscale MCIDs of 9.9–10.2 [37] and with reported KOOS MCID ranges in knee populations [38]. Because KOOS MCID values vary by subscale, population, and clinical context, this aggregate threshold was interpreted with caution and may overestimate response in athletes with mild–moderate PFP. Responder rates are reported as proportions with exact binomial 95% CIs.

Secondary analyses characterized individual post-injection trajectories after the early period. For each participant, a post-injection improvement slope (points/month) was estimated by linear regression across the three post-injection points coded as 0.5, 3, and 6 months. Baseline scores were not included in slope estimation precisely so they could subsequently be evaluated as predictors of slope; slopes are thus simplified linear summaries of the 0.5–6-month trajectory and assume approximate linearity, whereas actual recovery may be non-linear.

Simple linear regression modeled post-injection slope as a function of its baseline score (IKDC slope vs baseline IKDC; KOOS slope vs baseline KOOS). Multiple linear regression then evaluated whether baseline BMI (continuous) was independently associated with slope after adjustment for baseline score (IKDC slope ~ baseline IKDC + BMI; KOOS slope ~ baseline KOOS + BMI). Coefficients, 95% CIs, and exact p-values are reported. Regression diagnostics included Shapiro–Wilk normality of residuals, Breusch–Pagan heteroscedasticity, variance inflation factor (VIF) for multicollinearity, and Cook’s distance for influence. BMI subgroup analyses (BMI < 25 vs ≥ 25 kg/m^2^) were exploratory and descriptive: within-group mean change with 95% CI, between-group mean differences with Welch t-test (and standardized difference), and responder proportions with Fisher exact test, interpreted alongside the primary continuous BMI models.

Safety was summarized descriptively (*n*, %) without hypothesis testing. All tests were two-sided, exact *p*-values reported where appropriate (very small as *p* < 0.001), α = 0.05. Given the exploratory nature of secondary analyses, emphasis was placed on effect estimates and 95% CIs rather than significance alone. No correction for multiple comparisons was applied; the primary pre-specified contrast was baseline–6 months, and all other contrasts are hypothesis-generating, not confirmatory.

## Results

### Study population and baseline characteristics

Sixty athletes completed the protocol and were included in the final complete-case analysis. No missing IKDC or KOOS values were present across the four time points. Baseline characteristics are summarized in Table 1. Mean age was 42.6 years (SD 8.2) and mean BMI was 24.0 kg/m² (SD 3.2). Thirty-six participants (60.0%) were women and 24 (40.0%) were men. By Tegner activity level, 10 participants (16.7%) were classified as professional [9,10], 38 (63.3%) as amateur [7,8], and 12 (20.0%) as recreational [5,6]. Twenty-two participants (36.7%) had a baseline BMI of ≥25 kg/m².

**Table 1:** Baseline demographic and clinical characteristics.

Characteristic	*n*	%	Mean (SD)
Age, years	60	—	42.6 (8.2)
BMI, kg/m²	60	—	24.0 (3.2)
Sex, female	36	60.0	—
Sex, male	24	40.0	—
Athlete type, professional (Tegner 9–10)	10	16.7	—
Athlete type, amateur (Tegner 7–8)	38	63.3	—
Athlete type, recreational (Tegner 5–6)	12	20.0	—
BMI subgroup, < 25 kg/m²	38	63.3	—
BMI subgroup, ≥ 25 kg/m²	22	36.7	—

### Longitudinal IKDC and KOOS scores

On average, IKDC and KOOS scores increased progressively during follow-up (Table 2, Fig 2). Most of the score gain occurred by 3 months (IKDC ~+16.2, KOOS ~+15.8), with smaller additional increases from 3 to 6 months (IKDC ~+2.3, KOOS ~+3.4). Linear mixed model (LMM) analysis confirmed this pattern: compared with baseline, estimated mean IKDC changes were +9.11 (95% CI 7.33–10.89) at 15 days, +16.21 (14.44–17.99) at 3 months, and +18.58 (16.81–20.36) at 6 months; corresponding KOOS changes were +9.60 (7.84–11.36), +15.85 (14.09–17.61), and +19.28 (17.52–21.04) (all *p* < 0.001; overall time effect *p* < 10^−52^ for both outcomes). Individual trajectories varied, but the overall group trend was consistent.

**Table 2:** IKDC and KOOS scores over time and change from baseline (linear mixed model).

Assessment time	IKDC Mean (SD)	IKDC change from baseline (95% CI)	KOOS Mean (SD)	KOOS change from baseline (95% CI)
Baseline	64.7 (10.7)	—	68.4 (10.7)	—
15 days	73.8 (11.1)	+9.11 (7.33 to 10.89)	78.0 (8.3)	+9.60 (7.84 to 11.36)
3 months	80.9 (9.0)	+16.21 (14.44 to 17.99)	84.2 (8.9)	+15.85 (14.09 to 17.61)
6 months	83.2 (9.0)	+18.58 (16.81 to 20.36)	87.6 (7.7)	+19.28 (17.52 to 21.04)

**Fig 2:**
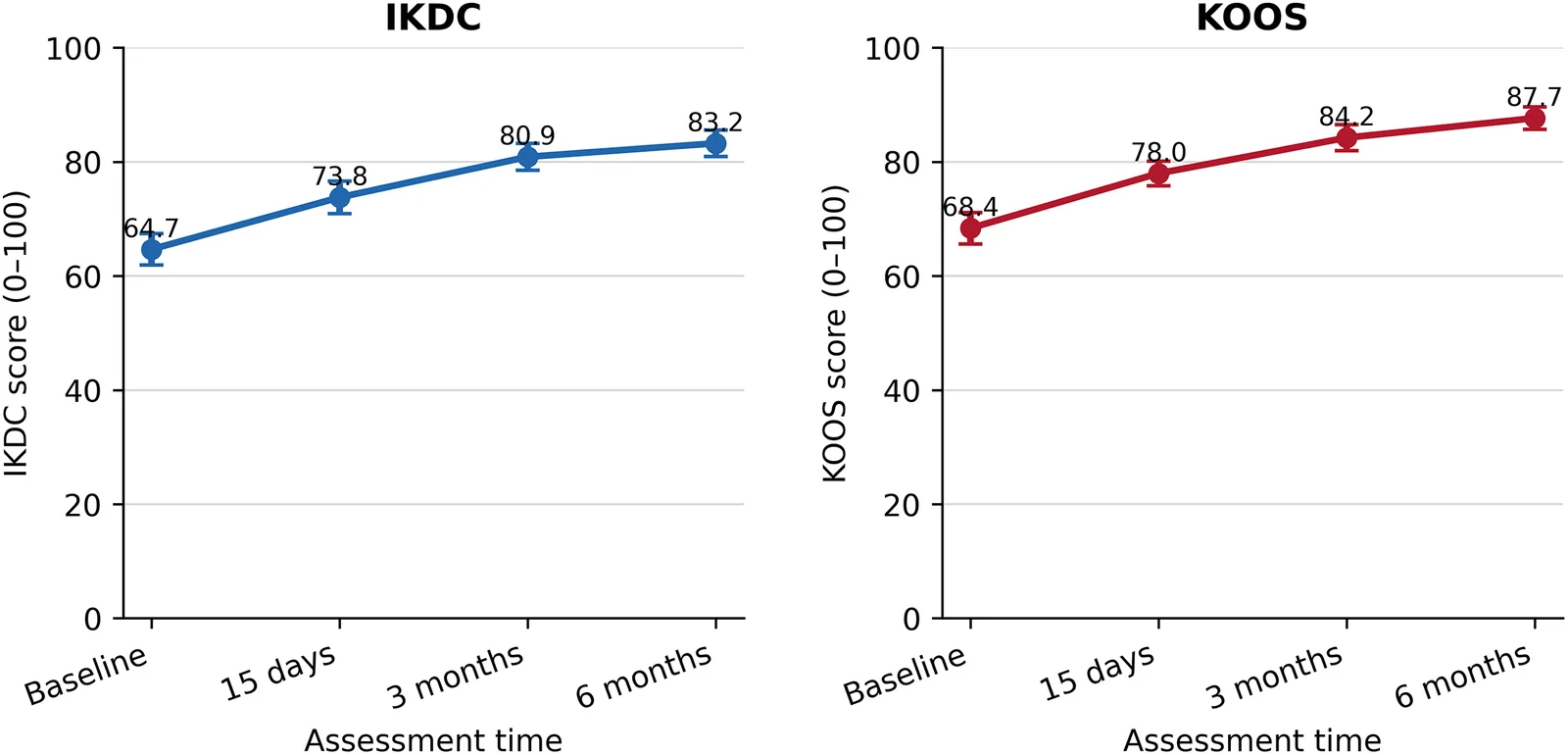
Longitudinal IKDC and KOOS scores during 6 months of follow-up. Mean scores and 95% confidence intervals are shown at baseline, 15 days, 3 months, and 6 months for the 60 athletes. The figure describes within-participant longitudinal changes observed after hyaluronic acid–sorbitol injection and does not establish a causal treatment effect.

### Paired changes from baseline

Within-participant paired changes from baseline to 6 months were 18.58 points for IKDC (95% CI 16.34–20.82, Cohen’s dz = 2.14) and 19.28 points for KOOS (95% CI 16.95–21.61, dz = 2.14) (Table 3); dz is reported as a descriptive effect size and is not a measure of treatment causality in this single-arm design. GEE sensitivity analysis yielded equivalent estimates.

**Table 3:** Paired changes from baseline.

Outcome	Comparison	n	Mean change (SD)	95% CI	t	p	Cohen’s dz
IKDC	Baseline → 15 days	60	9.11 (5.93)	7.57–10.64	11.89	<0.001	1.53
IKDC	Baseline → 6 months	60	18.58 (8.67)	16.34–20.82	16.61	<0.001	2.14
KOOS	Baseline → 15 days	60	9.60 (5.66)	8.14–11.06	13.13	<0.001	1.70
KOOS	Baseline → 6 months	60	19.28 (9.02)	16.95–21.61	16.57	<0.001	2.14

### Clinically meaningful improvement

At 6 months, 45 of 60 participants (75.0%, 95% CI 62.1–85.3) reached the predefined IKDC threshold and 50 of 60 (83.3%, 71.5–91.7) reached the KOOS threshold (Table 4).

**Table 4:** Clinically meaningful improvement at 6 months.

Outcome	MCID threshold	Responders, n/ N	Responder rate, % (95% CI)
IKDC	≥11.5	45/ 60	75.0 (62.1–85.3)
KOOS	≥10.0	50/ 60	83.3 (71.5–91.7)

### Individual post-injection improvement slopes

The mean post-injection improvement rate, summarized as an approximate linear trend across the 0.5-, 3-, and 6-month intervals, was 1.69 IKDC points/month (SD 1.46, 95% CI 1.32–2.07) and 1.74 KOOS points/month (SD 1.11, 95% CI 1.45–2.03).

### Association between baseline score and post-injection improvement rate

Lower baseline functional scores were associated with greater subsequent improvement rates. Baseline IKDC was inversely associated with IKDC slope (β = −0.065, 95% CI −0.097 to −0.034, p < 0.001), and baseline KOOS with KOOS slope (β = −0.047, −0.071 to −0.023, *p* < 0.001); this association does not establish a causal relationship (Fig 3).

**Fig 3:**
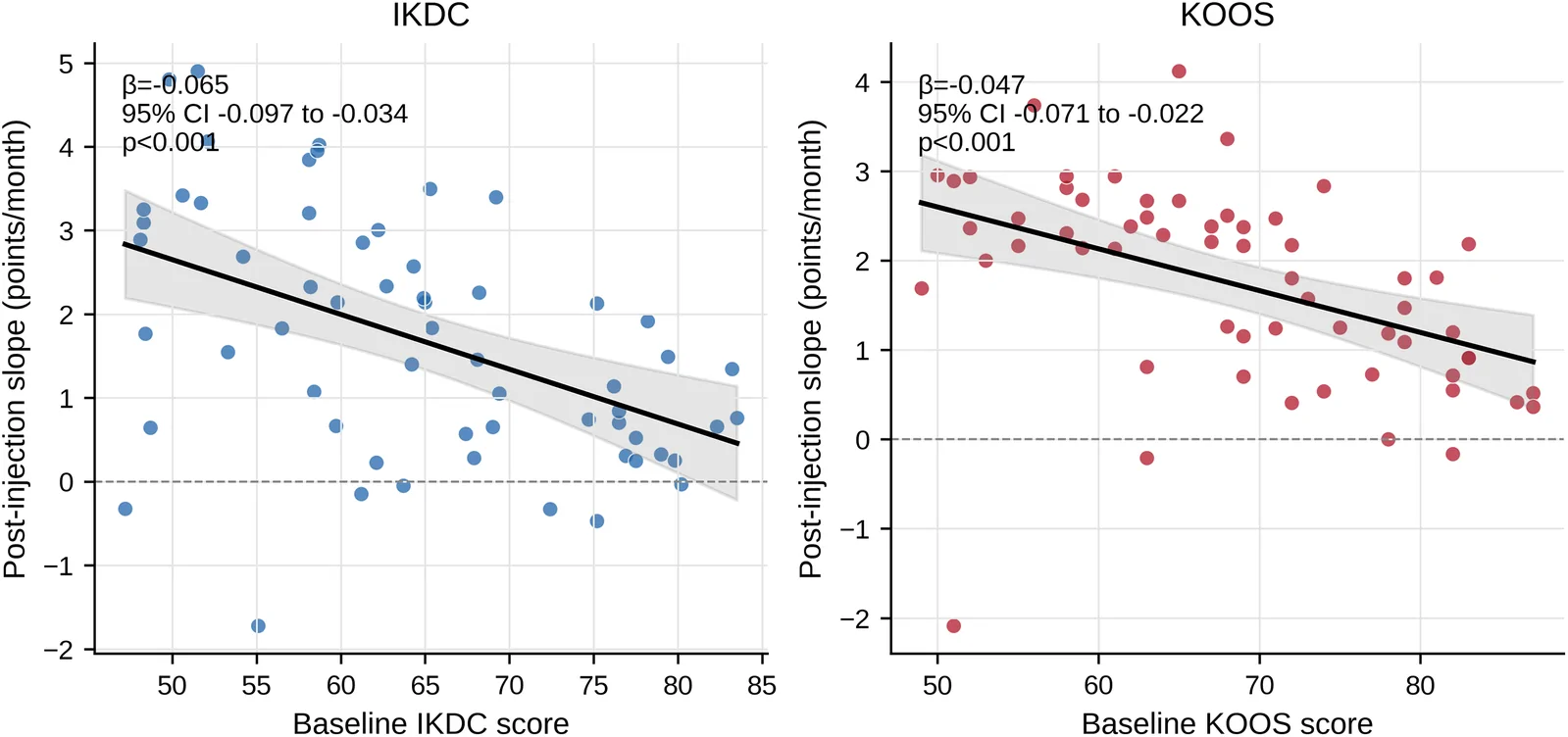
Association between baseline functional score and individual post-injection improvement rate. Each point represents one participant. Individual post-injection slopes were estimated by linear regression across the 15-day, 3-month, and 6-month assessments, corresponding to 0.5–6 months after injection. Lines represent simple linear regression estimates, with β coefficients, 95% confidence intervals, and two-sided p-values shown in each panel. Associations between baseline score and subsequent slope should not be interpreted as causal effects.

### BMI and post-injection improvement rate

In models adjusted for baseline score, BMI was not independently associated with improvement rate (IKDC β = 0.043, 95% CI −0.063 to 0.150, *p* = 0.417; KOOS β = −0.001, −0.084 to 0.082, *p* = 0.983); adding BMI did not improve model fit over the baseline-only model (F-test *p* = 0.42 IKDC, *p* = 0.98 KOOS; Table 5). Regression diagnostics identified evidence of heteroscedasticity for the IKDC model (Breusch–Pagan p = 0.022) and non-normal residuals for the KOOS model (Shapiro–Wilk *p* < 0.001); no evidence of multicollinearity or influential observations was found. For KOOS, non-normal residuals were noted; given the modest sample size, this departure is interpreted alongside the linearity assumption already discussed as a limitation of the slope-based approach (Fig 4).

**Table 5:** Associations between baseline functional score and post-injection improvement rate.

***Panel A. Regression models***				
**Outcome**	**Model**	**Predictor**	**β (95% CI), *p***	
IKDC	Simple	Baseline IKDC	−0.065 (−0.097 to −0.034), p < 0.001	
IKDC	Multiple	Baseline IKDC/ BMI	−0.065 (−0.097 to −0.034), p < 0.001/ 0.043 (−0.063 to 0.150), p = 0.417	
KOOS	Simple	Baseline KOOS	−0.047 (−0.071 to −0.023), p < 0.001	
KOOS	Multiple	Baseline KOOS/ BMI	−0.047 (−0.071 to −0.023), p < 0.001/ −0.001 (−0.084 to 0.082), p = 0.983	
***Panel B. Regression diagnostics***				
**Outcome**	**Shapiro–Wilk (residuals)**	**Breusch–Pagan**	**VIF**	**Cook’s D, max**
IKDC	*p*=0.22 (normal)	*p* = 0.022	~1.0	<0.18
KOOS	*p* < 0.001 (non-normal residuals)	*p* = 0.22	~1.0	<0.18

**Fig 4:**
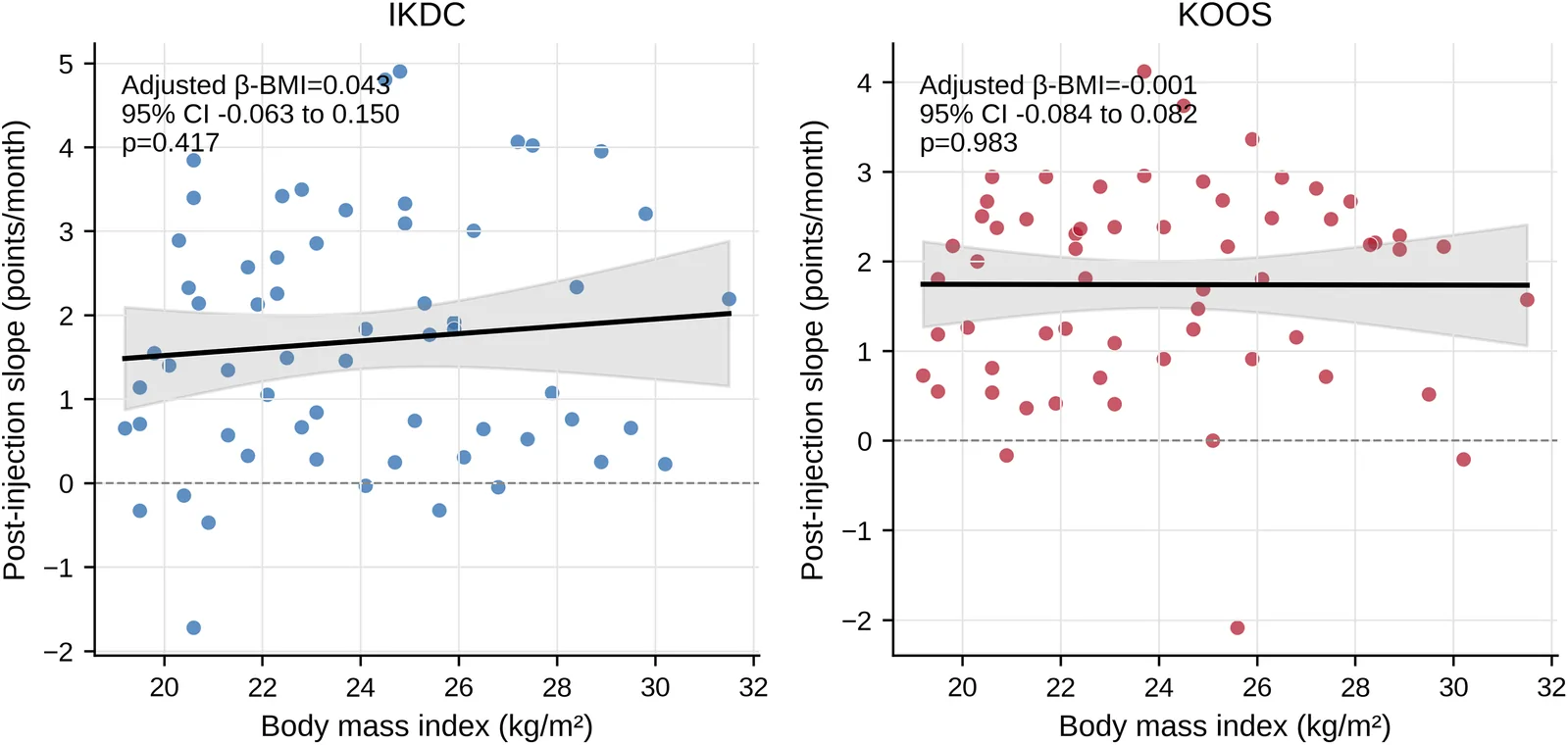
Association between baseline body mass index and individual post-injection improvement rate after adjustment for baseline functional score. Each point represents one participant. Individual post-injection slopes were estimated from the 15-day, 3-month, and 6-month assessments. Multiple linear regression models included baseline IKDC or KOOS score and baseline body mass index as continuous predictors. β-BMI denotes the adjusted regression coefficient for body mass index; results are exploratory.

### Exploratory BMI subgroup analysis

Because BMI was not independently associated with improvement rate in the adjusted models, subgroup comparisons by BMI category are exploratory. Both subgroups showed clinically relevant within-group improvement. Between-group differences were numerically lower for BMI ≥ 25 kg/m^2^ but did not reach statistical significance for IKDC (3.48 points, 95% CI −1.03 to 7.99, *p* = 0.127) or KOOS (4.47 points, −0.20 to 9.13, *p* = 0.060); the KOOS comparison approached significance and should be read as inconclusive given the smaller subgroup size (*n* = 22) (Table 6, Fig 5).

**Table 6:** Exploratory BMI subgroup analysis.

Outcome	BMI subgroup	*n*	Mean change (SD)	95% CI	Responders, *n* (%)	Difference, <25 minus ≥25 (95% CI)	Post-hoc power, %
IKDC	<25 kg/m²	38	19.86 (8.84)	16.96–22.77	31 (81.6)	3.48 (−1.03 to 7.99), *p* = 0.127 (means); *p* = 0.137 (responders)	32
IKDC	≥25 kg/m²	22	16.38 (8.09)	12.79–19.96	14 (63.6)	—	—
KOOS	<25 kg/m²	38	20.92 (9.05)	17.95–23.90	34 (89.5)	4.47 (−0.20 to 9.13), *p* = 0.060 (means); *p* = 0.149 (responders)	46
KOOS	≥25 kg/m²	22	16.45 (8.42)	12.72–20.19	16 (72.7)	—	—

**Fig 5:**
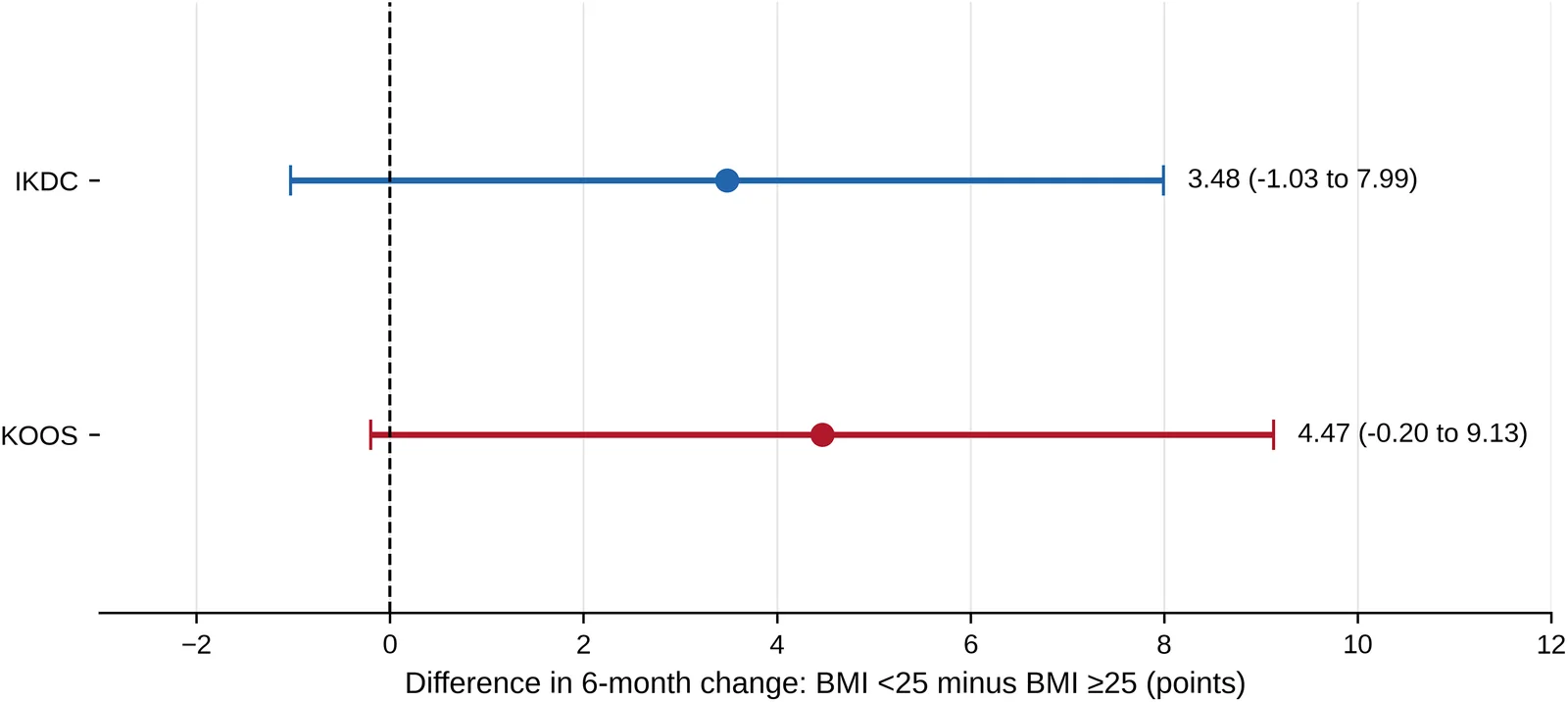
Exploratory differences in 6-month change in IKDC and KOOS scores by baseline body mass index subgroup. Points represent the mean difference in change from baseline to 6 months between participants with BMI < 25 kg/m^2^ and those with BMI ≥ 25 kg/m^2^. Positive values indicate greater mean improvement in the BMI < 25 kg/m^2^ subgroup. Horizontal lines represent 95% confidence intervals calculated using Welch’s t-test. These subgroup comparisons were exploratory and should be interpreted in view of the limited sample size in the BMI ≥ 25 kg/m^2^ subgroup.

### Safety and adverse events

Minor local adverse events occurred in 4 of 60 participants (6.7%): transient injection-site pain, swelling, or erythema, all resolved with local cryotherapy for 1–2 days. No pharmacological treatment was needed, no participant withdrew due to an adverse event, and no serious adverse events occurred.

## Discussion

### Principal findings

In this prospective single-arm study of athletes with PFP, IKDC and KOOS scores increased progressively during 6 months of follow-up after HA–sorbitol injection when analyzed with an appropriate repeated-measures model (LMM/GEE). LMM estimated mean improvements vs baseline of +18.58 points for IKDC and +19.28 for KOOS at 6 months (both *p* < 0.001), with most gain by 3 months. Using predefined a priori approximations, a substantial proportion reached clinically meaningful thresholds at 6 months (75.0% for IKDC, 83.3% for KOOS). These within-participant improvements were observed consistently across both instruments and exceeded the predefined thresholds used to characterize clinically meaningful change. In keeping with the single-arm design, these time trends are described as improvements observed during follow-up after treatment, not as demonstrated treatment efficacy.

### Interpretation of longitudinal improvement and TREND bias considerations

The temporal profile, early gains at 15 days, larger gains by 3 months, then stabilization, may reflect short-term changes in pain perception and joint tolerance followed by gradual functional recovery, potentially facilitating return to activity and confidence in sport-specific movements. However, without a placebo/sham, rehabilitation only, or no-treatment comparator, we cannot attribute improvements solely to the injection. As TREND emphasizes for non-randomized interventional studies, observed changes may include placebo/expectation effects, regression to the mean (higher scores on repeat testing among those with initially lower scores), natural symptom fluctuation, and unsupervised load modification. PFP is known for a fluctuating, often self-limiting course, so regression to the mean is a particularly relevant alternative explanation. The consistency of the LMM and GEE sensitivity analyses, together with the observed inter-time correlations, supports the robustness of the longitudinal pattern, while causal inference remains precluded by the single-arm design.

### Baseline function and improvement rate

Lower baseline IKDC/KOOS scores were associated with greater post-injection improvement rates, likely reflecting greater room for measurable change among those with worse baseline status and possibly more noticeable functional gains among more symptomatic athletes. Baseline functional status was the strongest predictor of post-injection slope in the regression models. This may inform future stratification strategies and interpretation of expected recovery patterns in active patients but should not be interpreted as a causal effect of baseline status on treatment response.

### Influence of elevated BMI

A secondary aim was to explore whether elevated BMI was associated with different longitudinal trajectories. Participants with BMI ≥ 25 kg/m^2^ showed clinically relevant within-group improvements, but mean changes and responder proportions were numerically lower than in BMI < 25 kg/m^2^. However, exploratory between-group differences, especially KOOS 4.47 points, 95% CI −0.20 to 9.13, p = 0.060, should be viewed as a non-significant trend with substantial uncertainty. With n = 38 vs 22, post-hoc power for observed IKDC (d = 0.41) and KOOS (d = 0.51) differences was only 32% and 46%, and a Type II error cannot be excluded; 62–97 participants per group would be needed for 80% power to detect differences of this magnitude, whereas power was > 98% to detect the full MCID of 10–11.5 points. Given this limited power, the appropriate interpretation is that this exploratory analysis did not detect a statistically significant BMI association, rather than that BMI has no effect on treatment response.

This is clinically relevant because BMI is often considered a negative prognostic factor via mechanical load and metabolic inflammation. Yet in athletic populations BMI is an imprecise proxy for adiposity: elevated BMI may reflect greater muscle mass rather than excess fat [39,40]. Ferreira et al. have shown that body fat and skeletal muscle mass, but not BMI alone, relate to pain mechanisms and functional capacity in young adults with PFP [15,39,40]. Future studies should therefore include direct body composition measures (fat mass %, waist circumference, imaging) to clarify whether adiposity, muscle mass, or loading patterns modify outcomes.

### Biological plausibility and potential mechanisms

The rationale for HA–sorbitol combines HA’s viscoelastic/lubricating properties with sorbitol’s proposed antioxidant action via reactive oxygen species scavenging, relevant in mechanically stressed joints and in elevated BMI states where low-grade inflammation may heighten vulnerability [29–32]. This is plausible for athletes with repetitive patellofemoral loading and high training demands. The longitudinal improvements observed are consistent with this rationale, but without synovial biomarkers, imaging, or biomechanical endpoints, mechanisms remain speculative and require direct evaluation in future studies.

### Psychological and functional considerations

PFP is increasingly recognized as multifactorial, involving local mechanical/biological factors and psychological/behavioral influences — pain self-efficacy, fear of movement, symptom persistence, training modification, and confidence during activity. Glaviano et al. reported associations between pain self-efficacy and physical activity in PFP [41], Pazzinatto et al. noted that kinesiophobia may emerge as symptoms persist [42], and a critically appraised topic found hip/knee strength changes were not consistently associated with PFP improvement despite functional gains [43].

In this context, the randomized trial by Bagheri and Naderi et al. offers a relevant comparison [44], where adding an 8-week mindfulness program to exercise therapy improved pain, knee function, fear of movement, pain catastrophizing, and coping versus exercise alone in recreational female runners with PFP. That trial illustrates that behavioral/psychological adjuncts can influence the same IKDC/KOOS domains we measured. Although we did not assess psychological variables, strength, or return-to-sport confidence, our IKDC/KOOS gains may reflect changes in pain, function, confidence, activity tolerance, or their combination. Future comprehensive assessment in athletes with PFP should integrate patient-reported outcomes, physical performance, training load, psychological factors, and return-to-sport metrics.

### Safety and tolerability

The single HA–sorbitol injection was well tolerated over 6 months. The 6.7% rate of minor, self-limited local reactions, managed with cryotherapy and without pharmacologic treatment or discontinuation, supports feasibility in active individuals, while larger studies are needed to detect uncommon adverse events. The absence of systemic or serious events is reassuring for athletic populations where downtime and delayed return to sport are key considerations.

### Limitations

First, the single-arm design without a placebo, sham injection, rehabilitation-only, or untreated comparator precludes causal inference; observed improvements are longitudinal within-participant changes after treatment and may reflect placebo, regression to the mean, natural fluctuation, expectation, and unsupervised load modification. Second, follow-up was limited to 6 months, useful for short- to intermediate-term description but insufficient for durability and recurrence during continued athletic participation; ≥12 months is needed. Third, the sample was modest, particularly for BMI subgroups (*n* = 22 with BMI ≥ 25), limiting power to detect clinically relevant differences (KOOS p = 0.060 trend; post-hoc power 32–46% for observed differences; possible Type II error). Fourth, BMI is an imprecise adiposity measure in athletes and may reflect lean mass; future work should include body composition as noted. Fifth, the single standardized preventive education session (injury-prevention, not therapeutic PFP rehabilitation) was delivered to reduce secondary exclusions, but any therapeutic co-intervention, bracing/taping, or unsupervised activity modification was not controlled and remains a potential confounder separable only with a randomized design. Sixth, we did not include objective biomechanical, imaging, return-to-sport, analgesic-use, rehabilitation-adherence, strength, psychological, or inflammatory/oxidative biomarker endpoints. Seventh, post-injection slopes are simplified linear summaries across 0.5, 3, and 6 months and assume approximate linearity; true recovery may be non-linear, as suggested by larger early mean gains. Finally, MCID thresholds were a priori approximations extrapolated from subscale and surgical (tibial tubercle osteotomy) populations [37] and may overestimate responder proportions in athletes with mild–moderate PFP.

### Future directions

Randomized controlled trials are needed to confirm these descriptive findings and isolate the contribution of HA–sorbitol beyond natural history, placebo, and standard rehabilitation. Comparator groups should include sham injections, structured exercise-based rehabilitation, HA without sorbitol, and other injectables, with ≥12-month follow-up. Larger samples would support robust evaluation of BMI, body composition, sex, sport type, baseline severity, training load, psychological factors, strength, biomechanics, and return-to-sport as modifiers, reflecting PFP’s multifactorial nature and helping identify athletes most likely to benefit from intra-articular therapies.

## Conclusion

Athletes with patellofemoral pain, including those with elevated body mass index, showed consistent improvement in knee function and symptoms over the six months following hyaluronic acid–sorbitol injection. These findings support intra-articular hyaluronic acid–sorbitol as a feasible and well-tolerated option for athletes with patellofemoral pain, regardless of body weight. As a single-arm study, this work should be followed by controlled trials to confirm the treatment’s contribution to these outcomes.

## Supporting information

S1 Dataset Anonymized patient dataset. De-identified patient-level IKDC/KOOS scores across all time points, alongside demographic and intervention variables.
(XLSX)

S1 Protocol Research protocol. (DOCX)

S1 Checklist TREND statement checklist. (DOCX)
