# Supplementary material for: Clinical outcomes of hyaluronic acid–sorbitol injection in athletes with patellofemoral pain: Evaluation of the potential influence of body mass index
Source: PLoS One. 2026 Sep 30;21(9):e0346269. doi: 10.1371/journal.pone.0346269 (PMC13626342; doi:10.1371/journal.pone.0346269)
Supplement: Supplementary file 1 [file pone.0346269.s002.docx]

RESEARCH PROTOCOL

Study title: Safety and effectiveness of hyaluronic acid and sorbitol with overuse knee pain in athletes and those with elevated body mass index.

Protocol Reference ethics commitee number: CEI/0504/PI./2022

Principal Researcher: Md. Julio Carlos Vélez de Lachica, Orthopedic surgeon, Orthopedics department head in the Regional Hopsital of Tlalnepantla, Mexico.

Adress: Av. Paseo del Ferrocarril # 88. Los Reyes Ixtacala. CP: 54055, Tlalnepantla de Baz, Estado de México.

Contact infromation: (52) 55 2626 9200 ext.: 2100 and ext.: 2101

email.: [activeknee@gmail.com](mailto:activeknee@gmail.com)

[aBSTRACT](#_Toc101176613)

INTRODUCTION

KNEE OVERUSE KNEE SYNDROME

ETIOLOGY

DIAGNOSIS

IMAGE DIAGNOSIS

CLASSIFICATIONS

OVERUSE KNEE SYNDROME TREATMENT

NON SURGICAL

ANTIINFLAMATORY NON STEROIDEAL TRATMENT

INFILTRATIONS

PHYSIOTHERAPY

HIALURONIC ACID TYPES:

HYALURONIC ACID PLUS SORBITOL

EVALUATION

RESEARCH QUESTION

PROTOCOL JUSTIFICATION

HYPOTHESYS

HYPOTHESIS

NULE HYPOTHESIS

OBJECTIVES

MAIN OBJECTIVE

SPECIFICT OBJETIVES

MATERIALS

STUDY DESIGN

SELECTION CRITERIA

INCLUSION CRITERIA

EXCLUSION CRITERIA

ELIMINATION CRITERIA

VARIABLES

POPULATION UNIVERSE

POPULATION SIZE

STUDY DESIGN

STATISTICAL ANALYSIS

ETHICAL IMPLICATION

ORGANIZATION

APPENDIX 1

APPENDIX 2

APPENDIX 3

REFERENCES

Abstract:

Introduction: The presence of overuse syndrome has been found with increasing frequency in patients who perform impact sports activities. Repetitive micro-trauma as a result of intense sport will cause the onset of joint degeneration that will generate pain, functional disability and in some cases limitation to continue carrying out sports activities.

Aims: the aims of this prospective and longitudinal intervention cohort study is to evaluate the safety and efficacy of hyaluronic acid plus sorbitol through clinical and functional analysis of sports patients undergoing overuse knee syndrome.

Patients and Methods:

According to the inclusion criteria, the participants who agree to part of the study will be enrolled, two dose of hyaluronic acid plus sorbitol will be applied in each knee, then strategies techniques for continue with sports activities. The KOOS and IKDC scores will be evaluated at 15 days 3 and 6 months. Once we obtain all data results a statistical analysis will be made.

Introduction:

It is increasingly common to find patients who refer knee pain after any kind of sports, overuse syndrome of the knee has been the most common cause. This entity can be conditioned by repetition microtrauma which are in direct relation with the repenting rise of the intensity, duration and volume in sports activity, and also we could find incorrect training techniques or also do not count with appropriate equipment during training.

Knee Overuse Syndrome

Musculoskeletal conditions are common during competitive practices and correspond to 80% of sports injuries. Of these, joint pathologies, especially of the knee, have increased significantly due to the large number of people who practice activities, both professionally and recreationally. Athletes who push their physical capacity to the limit, will present a type of joint injury that has been classified as excessive use of a joint, representing up to 42% of all sport-related musculoskeletal conditions. An overuse injury could be defined as one of gradual origin, caused by repetitive micro-traumas without an identifiable injury mechanism that triggered it. Therefore, the overuse syndrome is related to chronic repetitive injuries caused by sports activities, where one of its characteristics is that they occur when there is not enough time for small injuries to heal properly, these being the result of microtraumas associated with physical activity and exercise that exceed the tissue tolerance of the affected structure. There are distinct types of overuse injuries, which depend on the affected anatomical area: tendinitis, bursitis, stress fractures, osteoarthritis among others. The presence of these micro-traumas related to the increased intensity and frequency in the use of a joint Will eventually cause inflammation and wear of the cartilage, resulting in an overuse injury. The lower extremity is most affected, due to the overload imposed in sports involving running and jumping, in which the force of the knees can reach up to 10 times body weight.

Etiology

It has been observed that between 35 and 45% of all injuries related to sports activity are due to people with overuse syndrome and that approximately one third of these are related to the knee joint. The overall prevalence of knee overuse injuries varies between sports activities: For example, patellofemoral pain syndrome constitutes 25% of knee injuries in runners, patellar tendinopathy has a prevalence of 40 % to 50% in elite volleyball players and 2.8 to 4.8 in runners. It is common for patients with a history of knee overuse and who practice professional sports to develop degenerative cartilage injuries, causing knee osteoarthritis. It has been observed that knee osteoarthritis affects more frequently patients who practice sports activities on a regular basis. Despite the great benefits obtained from sports practice, an increase in pain and joint damage caused during training has been observed. This Will later lead to functional limitation, conditioning a decrease in physical performance or even premature withdrawal from the sport.

Diagnosis

The diagnosis of overuse injuries and early osteoarthritis in sports patients is carried out through physical examination, obtaining information related to the type of sport, weekly practice time and time spent practicing it, this can offer us a clear idea of what whether it is an injury caused by micro-traumas, in addition, to the fact that there is already an evidence limitation to complete the sports activity or even withdraw from it, can confirm the diagnosis. Adequate inspection for the identification of biomechanical factors that predispose to specific conditions are femoral anteversion, valgus or varus of the knee, patellar malposition, excessive lateral insertion of the patellar tendon, foot pronation, decreased muscle mass vastus medialis and abnormal trajectory of the patella. This i show the clinical recurrence of pain, discomfort, intermittent periods of inflammation or edema followed by long periods with minimal symptoms and anterior knee pain caused during the squat, Will lead us to the clinical diagnosis of overuse. As for radiographic studies, by ultrasound, magnetic resonance, or arthroscopy, these will allow us to stage the degrees of joint injury.

Patellofemoral knee pain in Athletes with Elevated BMI

In athletes with an elevated body mass index, this phenomenon is exacerbated due to the increased axial load on the knee joint, which accelerates cartilage degeneration processes, synovial inflammation, and biomechanical alterations. The combination of joint overuse and increased body weight not only intensifies and prolongs knee pain but also leads to greater functional limitation, negatively affecting sports performance and the continuity of physical activity, as well as increasing the risk of developing early-stage osteoarthritis.

Radiologic diagnosis:

- Simple X rays help to detect causes of knee pain, such as bipartite patella, osteoarthritis, loose bodies, occult fractures, and tumors. Several risk factors have even been identified, such as patella alta or lateralization of the patella in the femoral Groove. A radiographic study is required that allows us to identify specific injuries where degrees of joint injury in the knee can be stratified. Therefore, the diagnosis could be confirmed, by visualizing osteophytes, joint space narrowing, subcondral sclerosis, bone cysts, and grading these changes according to the Kellgren and Lawrence II to IV scale.
- MRI can provide information on degenerative changes, such as fissures or thinning of cartilage, subchondral bone marrow edema and cysts.
- Musculoskeletal ultrasound can be a useful tool to identify focal changes in tendon and subtle changes in vascularity (by mean of color / Doppler), one of its benefits, although it’s low cost, Will be the absence of radiation and patient comfort, compared with another image modalities.

CLASSIFICACION

Overuse injuries of the knee in adults include patello femoral Paint syndrome, quadriceps-patellar tendinopathy, and iliotibial band syndrome. As an addition to the classification criterion described for osteoarthritis, a classification has been proposed to identify the subpopulation of patients in the early stage who have a high risk of presenting Frank osteoarthritis of the knee. This type of classification for early osteoarthritis, a classification has been proposed to identify the subpopulation of patients in the early stage who have an elevated risk of presenting Frank osteoarthritis of the knee. This type of classification for early osteoarthritis is focused not as an individual diagnostic method but allows comparison of groups or subgroups of populations with similar characteristics, allowing standardization in clinical studies. Classifying osteoarthritis in initial stages is complex since conventional imaging criterion as well as signs and symptoms appear more sporadically, manifesting when there is heavy physical activity or repetitive sports. Therefore, a patient can be classified as having early osteoarthritis based on clinical and radiographic findings that must be integrated into the following 3 criteria:

1.- Knee pain (at least 2 episodes in les tan 10 days)

2.- Simple X-rays with Kellgren-Lawernce grade 0 or I or II osteoarthritis (osteophytes only)

3.- At least one for the following criterion:

- Arthroscopic findings of condral injury

- Findings of joint or meniscal degeneration by magnetic resonance imaging

The U-s National Athletic Injury Reporting System (NAIRS) divides overuse injuries into the following categories:

a) Undetectable – where the athlete does not stop their athletic activities,
b) Minor leaving1-7 days of sports activity
c) Moderate: from 8 to 21 days of abandonment of activities and
d) Major: more than 21 days of loss of activities and serious injuries, resulting in permanent disability.

TREATMENT OF KNEE OVERUSE SYNDROME:

There is no specific treatment guideline for most conditions caused by knee overuse. It is essential to have an adequate diagnosis of osteoarthritis at an early stage to incorporate the most appropriate treatment. The most recommended treatment for early osteoarthritis, includes weight loss, physical therapy, non-steroidal anti-inflammatory drugs, nutraceutical supplements, topical treatments, and steroid injections. In knee overuse syndrome, starting with a decrease in overload, correcting gait abnormalities, improving training techniques, adding strengthening and stretching exercises, as well as adapting the use of footwear and adding physical therapies with ice, have resulted effective methods as first line treatment.

NON-SURGICAL

The first line of treatment in early osteoarthritis of the knee caused by overuse in sports should be given conservatively and without surgery. The main objective of this treatment is to delay arthroplasty through a protocol that includes non-steroidal anti-inflammatory drugs, joint injections, as well as general measures of weight loss following a series of low-impact exercises. Physical therapy has an important role in the treatment of early osteoarthritis in athletes, stretching the extensor and abductor muscles of the knee can reduce pain and improve function. Cold therapy can also decrease the level of prostaglandins in synovial tissue resulting in decreased inflammation, reducing atherogenic muscle inhibition and facilitating vastus medialis motor neuron activity. Cryotherapy may allow more effective strengthening of the quadriceps in patients with knee joint abnormality; therefore, despite the current lack of clear research evidence, cryotherapy is recommended as a mainstay of conservative treatment in physiotherapy.

NON-STEROID ANTI-INFLAMMATORY AGENTS

Drug treatments commonly include the use of pain relievers such as aspirin or acetaminophen, and nonsteroidal anti-inflammatory drugs (NSAIDs); The use of NSAIDs is controversial due to the absence of histological inflammatory response in knee overuse syndrome. There is also concern that NSAIDs may interfere with the normal healing response of muscles and tendons.

INFILTRATIONS

- Platelet Rich Plasma (PRP) injections have been studied for the treatment of tendinopathy and osteoarthritis, obtaining Good medium-term results in the treatment of recalcitrant patellar tendinopathy by applying multiple injections. The use of PRP has been qualified by the Clinical Practice Guidelines of the American Academy of Orthopedic Surgeons (AAOS) for the treatment of knee osteoarthritis, however their decision was that it’s use cannot be recommended for or against treatment of knee osteoarthritis.

INFILTRATION WITH HIALURONIC ACID

Visco-supplementation with hyaluronic acid is one of the most recommended treatments for the non-surgical management of osteoarthritis, it can be recommended as first-line therapy, or when corticosteroids and non-steroidal anti-inflammatory drugs are inefficient, contraindicated or poorly tolerated; viscosupplementation is a safe procedure, without any systemic or local side effects, excluding possible injection pain and a feeling of heaviness for a few hours or days after treatment. Hyaluronic acid is one of the most used methods to preserve the cartilage and intra-articular structures of the knee. It protects the chondrocytes and lubricates the joint to stabilize the cartilage and the synovial membrane, benefits have also been described in its analgesic and intra-articular structures of the knee. It protects the chondrocytes and lubricates the joint to stabilize the cartilage and the synovial membrane, benefits have also been described in its analgesic and anti-inflammatory effects. Viscosupplementation significantly reduces knee pain during the first months, after which the effect is maintained for another 12 to 18 months. During this time joint function can improve and therefore patients can quickly return to sports activities.

Physiotherapy

A fundamental part of the overuse syndrome treatment is to know that mistakes in techniques of training could be avoided by guidelines of different kind of exercises. Physical therapy should focus on stretching inflexible muscles and tendons (for example: iliotibial band and hip external rotators in ITBS, hamstrings in patellar tendinopathy) and strengthening weak / core muscles (quadriceps and hip external rotators in PFPS/patella tendinopathy), in addition to the eccentric muscles. Specific training should be instituted for some conditions, such as quadriceps and patella tendinopathies. It has been found that physical therapy as a treatment for osteoarthritis in early stages has symptomatic benefits, and in sports patients it Will allow them to adapt a specific toning program that limits joint impact.

TYPES OF HYALURONIC ACID

Native HA. Has a molecular weight of 4-10 million Daltons and is present in joint fluids at a concentration of approximately 0.35 g/100 ml; Hyaluronic Acid is a glycosaminoglycan, linear polysaccharide, composed of repeating units (D-glucuronic acid and N-acetyl-glucosamine), is one of the main components of the extracellular matrix. It is part of the connective, epithelial and neuronal tissues. Infiltrations with hyaluronic acid are a viable option for athletes diagnosed with early osteoarthritis by reducing the absorption of loads in the knee, improving lubrication as well as having a certain degree of chondroprotection. Its principal biological effects are the regulation of anti-inflammatory mechanisms with antioxidants, reduction in the induction of cytokines, anabolic effect on cartilage and blockade of joint nociceptors that reduce pain.

Hyaluronic Acid with Sorbitol

Trade Name: Synolis VA

Approved chemical or generic name: hyaluronic acid (NaHa), sorbitol (C3H8O3), sodium chloride (NaCl), phosphate salts (Na2HPO4 + NaH2PO4) and sterile water for injection (SWFI).

Synolis VA is a viscoelastic, sterile, non-pyrogenic, isotonic, buffered solution of 2% hyaluronic acid. The hyaluronic acid used in synolis VA is obtained from bacterial fermentation and has a high molecular weight of 2 Mdaltons, it has a neutral pH of 6.8 - 7.4 like synovial fluid. High concentration and high molecular weight of hyaluronic acid combined with sorbitol limits its degradation and confers the capacity of viscoelastic solution to restore joint lubrication and shock absorption properties. Synolis VA work by restoring physiological and viscoelastic properties of synovial fluid that have been progressively lost during the development of osteoarthritis. Synolis VA therefore reduces pain and discomfort caused by symptomatic OA and improves joint mobility.

Composition: Each 1 ml contains: hyaluronic acid 20 mg, Sorbitol 40 mg, phosphate buffered solution 1ml. Synolis VA contains a 2 ml siringe of viscoelastic solution.

Contraindications.

Synolis VA should not be used in:

- Patients known to be hypersensitive to hyaluronic acid or sorbitol.
- Pregnant lactating patients
- Minors
- Skin disease or infection at the level of the application site.

CLINICAL EVALUATION

The knee injury and Osteoarthritis Outcome Score (KOOS) is a new, well-designed, and simple self-administered instrument developed to assess short, and long-term symptoms and function in patients with knee injuries and osteoarthritis. It has good evidence of reliability, validity, and responsiveness in different populations with different pathologies, injury duration, ages, and activity levels. The KOOS scale has been shown to be very effective in Young and active patients; it is intended to be used in short and long-time intervals; to assess week-to-week changes induced by treatment (medication, surgery, physical therapy). All items were scored from 0 to 4, and each of the five scores was calculated as the sum of the included items; scores are then converted to a scale of 0-100, where zero represented extreme knee problems and 100 represented no knee injury, this can be represented as a percentage.

The INTERNATIONAL KNEE DOCUMENTATION COMMITTEE IKDC, is a patient. oriented score to qualify symptoms and functions related to the activity of daily living; evaluates knee injuries and categorizes them into subjective variables, symptoms, ranges of motion and ligamentous exploration, which is subcategorized into normal, almost normal, abnormal and severely abnormal, the objective of this scale is to provide the physician with information on patient satisfaction and their knee. Point son the *Subjective Knee Assessment Form* are calculated by adding the points for the individual items, and then transforming the points on a scale from 0 to 100. The transformed points are interpreted as a measure of the ability to function, with higher points representing higher levels of functioning and lower levels of symptoms. One hundred points are interpreted to mean that there is no limitation in activities of daily living or sports activities, and the absence of symptoms.

Justification and Statement of the Problem

Currently there is a major influence to promote sport in the population, however, despite its great benefits, the development of early osteoarthritis secondary to micro-trauma due to overuse must be taken into consideration. In fact, the presence of early osteoarthritis following a prolonged or intense period of physical activity, or in those who practice impact sports regularly, is well described. Athletes who have initial stages of osteoarthritis require a treatment that improves their clinical symptoms and improves functional functions for as long as possible. Another objective of treatment is for their daily sports activities; sports activities; intraarticular HA injections are more effective in young patients, especially in the long term, while the results are les promising in athletes with advanced osteoarthritis. Reducing the viscoelasticity of synovial fluid, the transmission of mechanical force to the cartilage increases susceptibility to mechanical damage. Therefore the restoration of normal joint homeostasis is the reason for the administration of hyaluronic acid in the joints , in addition to seeking a physiological healing process and, if possible, complete; secondly, to shorten the recovery time, especially in athletes who are not going to limit their activity. The currently evidence that exists regarding the efficacy and safety of viscosupplementation in athletes is limited, hence the present investigation arises from the understanding that there are no protocols that demonstrate the benefits of knee joint infiltration using hyaluronic acid plus sorbitol in athlete patients, so in order to determine the safety and efficacy of this treatment, the following research question is proposed:

RESEARCH QUESTION

Is there a clinical and functional difference in the short and medium term in patients with knee pain due to overuse associated with sports treated by infiltration with hyaluronic acid with sorbitol?

Hypothesis

Athletes with sports-related overuse knee pain have Good clinical and functional results in the short and medium term through joint infiltration of hyaluronic acid with sorbitol.

Null Hypothesis

Athletes with knee pain due to sports-related overuse do not have Good clinical and functional results in the short and medium term through joint infiltration of hyaluronic acid with sorbitol.

Objectives

General Objective

To evaluate safety and efficacy of hyaluronic acid with sorbitol in athletes with overuse knee pain by comparing functional outcomes at short, medium and long term after treatment.

Specific Objectives

- To assess the safety and efficacy of hyaluronic acid with sorbitol in patients with sports-associated knee pain.
- To compare the clinical and functional results of patients with sport-associated knee pain after before and after treatment.
- To evaluate the clinical efficacy** of intra‑articular hyaluronic acid with sorbitol infiltration in athletes with elevated body mass index and knee pain due to overuse syndrome, using the KOOS and IKDC scores.

METHODS

Study Design

This research will be a cohort study, prospective and unidirectional direction.

Selection Criteria

- Patients with knee pain for more than 3 months
- Patients between 30 and 55 years old
- Patients without a history of previous knee joint surgery.
- Patients without chronic-degenerative diseases such as diabetes, hypertension, rheumatoid arthritis or other autoimmune diseases.
- Patients who perform impact social sports at least 3 times a week for more than 1 hour in the last 3 months prior to treatment.
- Patients without a history of allergies to non-steroidal anti-inflammatory drugs.
- Patients with no history of infection in or around the knee.

EXCLUSION CRITERIA

- Patients who cannot be categorized as social athletes.
- Patients with an associated sports injury that limits their sports practice.
- Patients who have a treatment associated with knee pain independent of that established in the study protocol.

ELIMITATION CRITERIA

- Patients who suffer from an injury or illness during the study that may limit their sports activity or who require an analgesic or anti-inflammatory treatment other than that established in the protocol.
- Patients who personally want to leave the study.
- Patients who did not have complete follow-up during the study.
- Patients who present an allergic or infectious reaction related to hyaluronic acid or prolotherapy or sepsis and antisepsis methods for infiltration.
- Patient with an allergic reaction to non-steroidal anti-inflammatory drugs.

VARIABLES

| Variable | Operational Definition | Dimension | Variable type | Instrument | Indicator |
| --- | --- | --- | --- | --- | --- |
| Age | Time of life of any person | Without categories | Quantitative  Discrete | Interview | Years |
| Gender | Biological and physiological characteristics that differentiate a man from a woman | Female  Male | Qualitative  Nominal | Interview | Gender |
| Body Mass Index | Score calculated by weight and height of a person. | Normal  Overweight  Obesity | Quantitative  Discrete | Measuring tape  Weighing machine | Weight in kg / Height in square meters |
| Affected Knee | Anatomic Knee side | Left  Right | Qualitative  Nominal | Physical Exploration | Left  Right |
| Athlete | Person who practice any type of sport | Social  Amateur  Professional | Qualitative  Nominal | Interview | Have a sports activity for more than 3 months, 3 times a week over 60 minutes per session |
| Score KOOS | Scores that evaluate functional and osteoarthrosis around the knee | Symptoms  Joint Stiffness  Pain  Regular activities  Sports activities  Quality of life | Quantitative  Discrete | Interview  Physical Exam | Percentage 0-100% |
| Score IKDC | International Knee Documentation Committee | Symptoms  Regular Activities  Sports Activities | Quantitative Discrete | Interview  Physical exam | Percentage  0-100% |
| Visco supplementation | Intraarticular joint injection of high weight molecular hyaluronic acid with sorbitol | Mono dose  Multi dose | Qualitative  Nominal | Hyaluronic acid 2ml plus sorbitol 2 dose per knee | Yes = Knee joint infiltration  No= Did not apply |
| Training sports technique | Coaching for adequate techniques of certain sports activities | Manual technique  Instrumented | Qualitative  Nominal | Personalized coaching techniques  Or  Information brochure | Achievement survey |

Research Universe

General Population who does sports activities and have knee overuse syndrome between June of 2022 and October 2022.

Sample Size

It will be a non-probability sampling where will be chosen participants who meet selection criteria over the enrolling time which will be for 5 months. The lower limit will be 60 participants and the higher limit will be 120 participants.

Sample size formula: n(sample), z (confidence level), p (probability to occur the studied event), q (probability to didn’t occur the studied event) and e (maximum accepted estimated error)

        Z2 * p*q

n = —————

             e2

Study Design

Research enrolling and diffusion will be initiated after authorization of the ethics and investigation committee. All participants with knee pain related to sports will be accepted for a physical evaluation. All participants will be interviewed about physical activities, sports, injuries, and knee pain. Once they have been categorized as athletes with knee overuse pain syndrome and have met the selection criteria, then will be invited to the research. For this, the participants must be agreed and sign the informed consent Appendix 1 and 2.

Before treatment Group

To participants who initiate the study research will be applied the KOOS and IKDC scores, the results will be recorder in the collection data sheet. Appendix 2. Participants will be explored by physical exam focus on knee pathology, and also an echography knee scan looking for non-overuse pain injuries.  Coaching for sports activities will be explain for each patient in order to improve its own training techniques adding stretching exercises for previous and after training.

After treatment Group

After KOOS and IKDC scores were recorder two dose of Hyaluronic Acid plus sorbitol will applied. Technique: On the examination table, the patient will lie down at supine position and the area of the knees to be infiltrated will be discovered.  Asepsis of the knee region will be performed using an iodine and alcohol solution, the remaining synovial fluid in the joint will be extracted with a 10cc syringe then 4 milliliters of 80 mg of hyaluronic acid plus 160 mg of sorbitol will be applied (Synolis VA Aptissen S. A.) at the sub quadricipital level with a 22G x 32mm sterile needle.  At the end of the infiltration, the needle will be removed, and the injection site will be covered with a small sticky-band, which can be thrown away after 20 minutes. It will be recommended do not made any kind of sports activity following the next 72 hours and applied ice therapy 3 times per day for over 20 minutes. Scores recorder data will be made at 15 days, then at 3 and 6 months.

Once the enrolling phase of the trial be concluded, statistical analysis will be made with Excel Office software for Windows, IBM SPSS and Prisma.

Statistical analysis

Recorder data of the functional scores will be evaluated before and after treatment, also at 15 days, 3 and 6 months after hyaluronic acid supplementation.

Data analysis will be made by means standard deviation for numeric variables, also t-test for parametric and Wilcoxon Mann Whitney for non-parametric variables, a standard error of 95% is allowed. Differences in time will be calculated and group compared by Anova test. Variable correlation and association measures with relative risk and attributable risk with Fisher’s test will be made with an confidence interval of 95%.

Ethical Implications

This research project will be developed after approvement of the ethics and research Institutional committee.

This research study will be according to principles of ethics by the Helsinki Declaration (2000) following three principles of ethics: beneficence, justice and respect for people.

All the declared principles will be followed in relation to the guide of good clinical practice.

This study research will follow the ethical codes established by the 18ª worldwide medical assembly, protection principle of the participants in the Nuremberg Code (1947), Belmont inform (1979), OMS guidelines and CIOMS (2002) guides for human biomedical research. Also following the NOM 012 of biomedical research health department.

Organization

Human Resources: Principal Research, co-author, medical assistant. Statesian

Economic burden: Noneconomic profit will be obtained by the principal author and coauthor. It will be no economic benefit will be for any participant.

Physical Resources:

Medical office: In this area medical assistant will collect the score records, physical examination, sonographic evaluation, coaching sports hygiene techniques, and stretching exercises.

# References

[1.	Patel DR, Villalobos A. Evaluation and management of knee pain in young athletes: overuse injuries of the knee. Transl Pediatr [Internet]. 2017 Jul;6(3):190–8. Available from: https://pubmed.ncbi.nlm.nih.gov/28795010](1. Patel DR, Villalobos A. Evaluation and management of knee pain in young athletes: overuse injuries of the knee. Transl Pediatr [Internet]. 2017 Jul;6(3):190–8. Available from: https://pubmed.ncbi.nlm.nih.gov/287950102. Nicolini AP, de Carvalho RT, Matsuda MM, Filho JS, Cohen M. Common injuries in athletes' knee: Experience of a specialized center. Acta Ortop Bras. 2014;22(3):127–31. 3. Tiu T, Craig Van Dien MD. Knee overuse disorders. 4. Fuller CW, Ekstrand J, Junge A, Andersen TE, Bahr R, Dvorak J, et al. Consensus statement on injury definitions and data collection procedures in studies  of football (soccer) injuries. Br J Sports Med. 2006 Mar;40(3):193–201. 5. Majewski M, Susanne H, Klaus S. Epidemiology of athletic knee injuries: A 10-year study. Knee. 2006 Jun;13(3):184–8. 6. Wilder RP, Sethi S. Overuse injuries: tendinopathies, stress fractures, compartment syndrome, and shin  splints. Clin Sports Med. 2004 Jan;23(1):55–81, vi. 7. Aicale R, Tarantino D, Maffulli N. Overuse injuries in sport: a comprehensive overview. J Orthop Surg Res. 2018 Dec;13(1):309. 8. Yang J, Tibbetts AS, Covassin T, Cheng G, Nayar S, Heiden E. Epidemiology of overuse and acute injuries among competitive collegiate athletes. J Athl Train. 2012;47(2):198–204. 9. Bernetti A, Mangone M, Paoloni M, Di Sante L, Murgia M, Santilli V. Corticosteroid and hyaluronic acid injection therapy in tennis elbow (lateral epicondylalgia). Med Sport. 2014;67(2):289–95. 10. Iolascon G, Gimigliano F, Moretti A, De Sire A, Migliore A, Brandi ML, et al. Early osteoarthritis: How to define, diagnose, and manage. A systematic review. Eur Geriatr Med. 2017;8(5–6):383–96. 11. Drawer S, Fuller CW. Propensity for osteoarthritis and lower limb joint pain in retired professional soccer players. Br J Sports Med. 2001;35(6):402–8. 12. Vannini F, Spalding T, Andriolo L, Berruto M, Denti M, Espregueira-Mendes J, et al. Sport and early osteoarthritis: the role of sport in aetiology, progression and treatment of knee osteoarthritis. Knee Surgery, Sport Traumatol Arthrosc. 2016;24(6):1786–96. 13. Fröhlich S, Pazeller S, Cherati AS, Müller E, Frey WO, Spörri J. Overuse injuries in the knee, back and hip of top elite female alpine skiers during  the off-season preparation period: prevalence, severity and their association with traumatic preinjuries and training load. BMJ open Sport Exerc Med. 2020;6(1):e000892. 14. Rincon GA, Vyas D, Zhou J, Fu FH, Oiestad BE, Holm I, et al. \“ Knee function and prevalence of knee osteoarthritis after anterior cruciate ligament reconstruction: a prospective study with 10 to 15 years of follow-up\”. Letter to the editor. Am J Sports Med. 2011;39(4):NP3-author. 15. Kellgren JH, Lawrence J. Radiological assessment of osteo-arthrosis. Ann Rheum Dis. 1957;16(4):494. 16. Altman R, Asch E, Bloch D, Bole G, Borenstein D, Brandt K, et al. Development of criteria for the classification and reporting of osteoarthritis: classification of osteoarthritis of the knee. Arthritis Rheum Off J Am Coll Rheumatol. 1986;29(8):1039–49. 17. Luyten FP, Denti M, Filardo G, Kon E, Engebretsen L. Definition and classification of early osteoarthritis of the knee. Knee Surgery, Sport Traumatol Arthrosc. 2012;20(3):401–6. 18. Hoemann C, Kandel R, Roberts S, Saris DBF, Creemers L, Mainil-Varlet P, et al. International Cartilage Repair Society (ICRS) Recommended Guidelines for  Histological Endpoints for Cartilage Repair Studies in Animal Models and Clinical Trials. Cartilage. 2011 Apr;2(2):153–72. 19. Hunter DJ, Lo GH, Gale D, Grainger AJ, Guermazi A, Conaghan PG. The reliability of a new scoring system for knee osteoarthritis MRI and the validity of bone marrow lesion assessment: BLOKS (Boston–Leeds Osteoarthritis Knee Score). Ann Rheum Dis. 2008;67(2):206–11. 20. Peterfy CG, Guermazi A, Zaim S, Tirman PFJ, Miaux Y, White D, et al. Whole-organ magnetic resonance imaging score (WORMS) of the knee in osteoarthritis. Osteoarthr Cartil. 2004;12(3):177–90. 21. Demange MK, Sisto M, Rodeo S. Future trends for unicompartmental arthritis of the knee: injectables & stem cells. Clin Sports Med. 2014;33(1):161–74. 22. Leslie M. Knee osteoarthritis management therapies. Pain Manag Nurs. 2000;1(2):51–7. 23. Paoloni M, Bernetti A, Belelli A, Brignoli O, Buoso S, Caputi AP, et al. Appropriateness of clinical and organizational criteria for intra-articular injection therapies in osteoarthritis: A Delphi method consensus initiative among experts in Italy. Ann Ist Super Sanita. 2015;51:131–8. 24. Sandmark H, Hogstedt C, Lewold S, Vingård E. Osteoarthrosis of the knee in men and women in association with overweight, smoking, and hormone therapy. Ann Rheum Dis. 1999;58(3):151–5. 25. Christensen BV, Iuhl IU, Vilbek H, Bülow H, Dreijer NC, Rasmussen HF. Acupuncture treatment of severe knee osteoarthrosis. A long‐term study. Acta Anaesthesiol Scand. 1992;36(6):519–25. 26. de Sire A, Agostini F, Lippi L, Mangone M, Marchese S, Cisari C, et al. Oxygen-Ozone Therapy in the Rehabilitation Field: State of the Art on Mechanisms of  Action, Safety and Effectiveness in Patients with Musculoskeletal Disorders. Biomolecules. 2021 Feb;11(3). 27. Fransen M, McCONNELL S. Land-based exercise for osteoarthritis of the knee: a metaanalysis of randomized controlled trials. J Rheumatol. 2009;36(6):1109–17. 28. Hiemstra LA, Kerslake S, Irving C. Anterior knee pain in the athlete. Clin Sports Med. 2014;33(3):437–59. 29. Filardo G, Kon E, Di Matteo B, Pelotti P, Di Martino A, Marcacci M. Platelet-rich plasma for the treatment of patellar tendinopathy: clinical and imaging findings at medium-term follow-up. Int Orthop [Internet]. 2013;37(8):1583–9. Available from: https://doi.org/10.1007/s00264-013-1972-830. Trojian TH, Concoff AL, Joy SM, Hatzenbuehler JR, Saulsberry WJ, Coleman CI. AMSSM Scientific Statement Concerning Viscosupplementation Injections for Knee  Osteoarthritis: Importance for Individual Patient Outcomes. Clin J Sport Med  Off J Can Acad  Sport Med. 2016 Jan;26(1):1–11. 31. Ammar TY, Pereira TAP, Mistura SLL, Kuhn A, Saggin JI, Lopes OV. Viscosupplementation for treating knee osteoarthrosis: review of the literature. Rev Bras Ortop. 2015;50:489–94. 32. Salini V, Pantalone A, Vanni D, Abate M. Viscosupplementation in Athletes. In: Arthroscopy and Sport Injuries. Springer; 2016. p. 433–7. 33. Erdem Y, Gul D, Akpancar S. Comparison of Intraarticular Injections of Hyaluronic Acid versus Dextrose Applied with Periarticular Prolotherapy in the Treatment of Recreational Athletes with Knee Osteoarthritis. Turkish J Sport Med. 2020;55(1):6–13. 34. Kalkhoven J, Coutts AJ, Impellizzeri FM. 'Training load error' is not a more accurate term than 'overuse' injury. Br J Sports Med [Internet]. 2020 Aug 1;54(15):934 LP – 935. Available from: http://bjsm.bmj.com/content/54/15/934.abstract35. Odole AC, Agbomeji OT, Onyeso OKK, Ojo JO, Odunaiya NA. Perspectives of Nigerian Athletes About Physiotherapy Services in Sports Injury Management: Implications for Rehabilitation. J Sport Rehabil. 2021;1(aop):1–8. 36. Hendrika W, Reswari A. The effect of physiotherapy on pain improvement in patients with early knee osteoarthritis at RSU UKI. Int J Med Heal Res. 2021;7(6):52–9. 37. MacKay C, Hawker GA, Jaglal SB. How Do Physical Therapists Approach Management of People With Early Knee Osteoarthritis? A Qualitative Study. Phys Ther [Internet]. 2019;100(2):295–306. Available from: https://doi.org/10.1093/ptj/pzz16438. Hrazdira L, Pilecki Z, Pilecki G. Ultrasound guided application of HA in the soft tissues of the locomotor system in athletes–First experience. Krátká sdělení Botek, M, Stejskal, P, Klimešová, I Vliv normobarické hypoxie na Akt Auton Nerv systému Pilot Stud 191 Drbošalová, V, Stejskal, P, Klugar, M, Krejčíř, V, Hynková, O Charakt klientů komerčn. :205. 39. Ricci M, Micheloni GM, Berti M, Perusi F, Sambugaro E, Vecchini E, et al. Clinical comparison of oral administration and viscosupplementation of hyaluronic acid (HA) in early knee osteoarthritis. Musculoskelet Surg. 2017;101(1):45–9. 40. Wang C-T, Lin Y-T, Chiang B-L, Lin Y-H, Hou S-M. High molecular weight hyaluronic acid down-regulates the gene expression of osteoarthritis-associated cytokines and enzymes in fibroblast-like synoviocytes from patients with early osteoarthritis. Osteoarthr Cartil. 2006;14(12):1237–47. 41. Irrgang JJ, Anderson AF. Development and validation of health-related quality of life measures for the knee. Clin Orthop Relat Res. 2002;402:95–109. 42. Roos EM, Roos HP, Lohmander LS, Ekdahl C, Beynnon BD. Knee Injury and Osteoarthritis Outcome Score (KOOS)—development of a self-administered outcome measure. J Orthop Sport Phys Ther. 1998;28(2):88–96. 43. Perruccio A V, Canizares M, Hawker GA, Roos EM, Maillefert JF, Lohmander SL. Comparative Validity and Responsiveness of the HOOS-PS and KOOS-PS to the WOMAC Physical Function Subscale in Total Joint Replacement for Osteoarthritis. 44. Salavati M, Mazaheri M, Negahban H, Sohani SM, Ebrahimian MR, Ebrahimi I, et al. Validation of a Persian-version of Knee injury and Osteoarthritis Outcome Score (KOOS) in Iranians with knee injuries. Osteoarthr Cartil. 2008;16(10):1178–82. 45. Higgins LD, Taylor MK, Park D, Ghodadra N, Marchant M, Pietrobon R, et al. Reliability and validity of the International Knee Documentation Committee (IKDC) subjective knee form. Jt Bone Spine. 2007;74(6):594–9. 46. Kujala UM, Kaprio J, Sarno S. Osteoarthritis of weight bearing joints of lower limbs in former elite male athletes. Bmj. 1994;308(6923):231–4. 47. Salini V, Pantalone A, Vanni D. Arthroscopy and Sport Injuries. Arthrosc Sport Inj. 2016;433–7. 48. Broglio K. Randomization in clinical trials: Permuted blocks and stratification. JAMA - J Am Med Assoc. 2018;319(21):2223–4. 49. Emanuel EJ, Wendler D, Grady C. What makes clinical research ethical? JAMA. 2000 May;283(20):2701–11. )

[2.	Nicolini AP, de Carvalho RT, Matsuda MM, Filho JS, Cohen M. Common injuries in athletes’ knee: Experience of a specialized center. Acta Ortop Bras. 2014;22(3):127–31.](1. Patel DR, Villalobos A. Evaluation and management of knee pain in young athletes: overuse injuries of the knee. Transl Pediatr [Internet]. 2017 Jul;6(3):190–8. Available from: https://pubmed.ncbi.nlm.nih.gov/287950102. Nicolini AP, de Carvalho RT, Matsuda MM, Filho JS, Cohen M. Common injuries in athletes' knee: Experience of a specialized center. Acta Ortop Bras. 2014;22(3):127–31. 3. Tiu T, Craig Van Dien MD. Knee overuse disorders. 4. Fuller CW, Ekstrand J, Junge A, Andersen TE, Bahr R, Dvorak J, et al. Consensus statement on injury definitions and data collection procedures in studies  of football (soccer) injuries. Br J Sports Med. 2006 Mar;40(3):193–201. 5. Majewski M, Susanne H, Klaus S. Epidemiology of athletic knee injuries: A 10-year study. Knee. 2006 Jun;13(3):184–8. 6. Wilder RP, Sethi S. Overuse injuries: tendinopathies, stress fractures, compartment syndrome, and shin  splints. Clin Sports Med. 2004 Jan;23(1):55–81, vi. 7. Aicale R, Tarantino D, Maffulli N. Overuse injuries in sport: a comprehensive overview. J Orthop Surg Res. 2018 Dec;13(1):309. 8. Yang J, Tibbetts AS, Covassin T, Cheng G, Nayar S, Heiden E. Epidemiology of overuse and acute injuries among competitive collegiate athletes. J Athl Train. 2012;47(2):198–204. 9. Bernetti A, Mangone M, Paoloni M, Di Sante L, Murgia M, Santilli V. Corticosteroid and hyaluronic acid injection therapy in tennis elbow (lateral epicondylalgia). Med Sport. 2014;67(2):289–95. 10. Iolascon G, Gimigliano F, Moretti A, De Sire A, Migliore A, Brandi ML, et al. Early osteoarthritis: How to define, diagnose, and manage. A systematic review. Eur Geriatr Med. 2017;8(5–6):383–96. 11. Drawer S, Fuller CW. Propensity for osteoarthritis and lower limb joint pain in retired professional soccer players. Br J Sports Med. 2001;35(6):402–8. 12. Vannini F, Spalding T, Andriolo L, Berruto M, Denti M, Espregueira-Mendes J, et al. Sport and early osteoarthritis: the role of sport in aetiology, progression and treatment of knee osteoarthritis. Knee Surgery, Sport Traumatol Arthrosc. 2016;24(6):1786–96. 13. Fröhlich S, Pazeller S, Cherati AS, Müller E, Frey WO, Spörri J. Overuse injuries in the knee, back and hip of top elite female alpine skiers during  the off-season preparation period: prevalence, severity and their association with traumatic preinjuries and training load. BMJ open Sport Exerc Med. 2020;6(1):e000892. 14. Rincon GA, Vyas D, Zhou J, Fu FH, Oiestad BE, Holm I, et al. \“ Knee function and prevalence of knee osteoarthritis after anterior cruciate ligament reconstruction: a prospective study with 10 to 15 years of follow-up\”. Letter to the editor. Am J Sports Med. 2011;39(4):NP3-author. 15. Kellgren JH, Lawrence J. Radiological assessment of osteo-arthrosis. Ann Rheum Dis. 1957;16(4):494. 16. Altman R, Asch E, Bloch D, Bole G, Borenstein D, Brandt K, et al. Development of criteria for the classification and reporting of osteoarthritis: classification of osteoarthritis of the knee. Arthritis Rheum Off J Am Coll Rheumatol. 1986;29(8):1039–49. 17. Luyten FP, Denti M, Filardo G, Kon E, Engebretsen L. Definition and classification of early osteoarthritis of the knee. Knee Surgery, Sport Traumatol Arthrosc. 2012;20(3):401–6. 18. Hoemann C, Kandel R, Roberts S, Saris DBF, Creemers L, Mainil-Varlet P, et al. International Cartilage Repair Society (ICRS) Recommended Guidelines for  Histological Endpoints for Cartilage Repair Studies in Animal Models and Clinical Trials. Cartilage. 2011 Apr;2(2):153–72. 19. Hunter DJ, Lo GH, Gale D, Grainger AJ, Guermazi A, Conaghan PG. The reliability of a new scoring system for knee osteoarthritis MRI and the validity of bone marrow lesion assessment: BLOKS (Boston–Leeds Osteoarthritis Knee Score). Ann Rheum Dis. 2008;67(2):206–11. 20. Peterfy CG, Guermazi A, Zaim S, Tirman PFJ, Miaux Y, White D, et al. Whole-organ magnetic resonance imaging score (WORMS) of the knee in osteoarthritis. Osteoarthr Cartil. 2004;12(3):177–90. 21. Demange MK, Sisto M, Rodeo S. Future trends for unicompartmental arthritis of the knee: injectables & stem cells. Clin Sports Med. 2014;33(1):161–74. 22. Leslie M. Knee osteoarthritis management therapies. Pain Manag Nurs. 2000;1(2):51–7. 23. Paoloni M, Bernetti A, Belelli A, Brignoli O, Buoso S, Caputi AP, et al. Appropriateness of clinical and organizational criteria for intra-articular injection therapies in osteoarthritis: A Delphi method consensus initiative among experts in Italy. Ann Ist Super Sanita. 2015;51:131–8. 24. Sandmark H, Hogstedt C, Lewold S, Vingård E. Osteoarthrosis of the knee in men and women in association with overweight, smoking, and hormone therapy. Ann Rheum Dis. 1999;58(3):151–5. 25. Christensen BV, Iuhl IU, Vilbek H, Bülow H, Dreijer NC, Rasmussen HF. Acupuncture treatment of severe knee osteoarthrosis. A long‐term study. Acta Anaesthesiol Scand. 1992;36(6):519–25. 26. de Sire A, Agostini F, Lippi L, Mangone M, Marchese S, Cisari C, et al. Oxygen-Ozone Therapy in the Rehabilitation Field: State of the Art on Mechanisms of  Action, Safety and Effectiveness in Patients with Musculoskeletal Disorders. Biomolecules. 2021 Feb;11(3). 27. Fransen M, McCONNELL S. Land-based exercise for osteoarthritis of the knee: a metaanalysis of randomized controlled trials. J Rheumatol. 2009;36(6):1109–17. 28. Hiemstra LA, Kerslake S, Irving C. Anterior knee pain in the athlete. Clin Sports Med. 2014;33(3):437–59. 29. Filardo G, Kon E, Di Matteo B, Pelotti P, Di Martino A, Marcacci M. Platelet-rich plasma for the treatment of patellar tendinopathy: clinical and imaging findings at medium-term follow-up. Int Orthop [Internet]. 2013;37(8):1583–9. Available from: https://doi.org/10.1007/s00264-013-1972-830. Trojian TH, Concoff AL, Joy SM, Hatzenbuehler JR, Saulsberry WJ, Coleman CI. AMSSM Scientific Statement Concerning Viscosupplementation Injections for Knee  Osteoarthritis: Importance for Individual Patient Outcomes. Clin J Sport Med  Off J Can Acad  Sport Med. 2016 Jan;26(1):1–11. 31. Ammar TY, Pereira TAP, Mistura SLL, Kuhn A, Saggin JI, Lopes OV. Viscosupplementation for treating knee osteoarthrosis: review of the literature. Rev Bras Ortop. 2015;50:489–94. 32. Salini V, Pantalone A, Vanni D, Abate M. Viscosupplementation in Athletes. In: Arthroscopy and Sport Injuries. Springer; 2016. p. 433–7. 33. Erdem Y, Gul D, Akpancar S. Comparison of Intraarticular Injections of Hyaluronic Acid versus Dextrose Applied with Periarticular Prolotherapy in the Treatment of Recreational Athletes with Knee Osteoarthritis. Turkish J Sport Med. 2020;55(1):6–13. 34. Kalkhoven J, Coutts AJ, Impellizzeri FM. 'Training load error' is not a more accurate term than 'overuse' injury. Br J Sports Med [Internet]. 2020 Aug 1;54(15):934 LP – 935. Available from: http://bjsm.bmj.com/content/54/15/934.abstract35. Odole AC, Agbomeji OT, Onyeso OKK, Ojo JO, Odunaiya NA. Perspectives of Nigerian Athletes About Physiotherapy Services in Sports Injury Management: Implications for Rehabilitation. J Sport Rehabil. 2021;1(aop):1–8. 36. Hendrika W, Reswari A. The effect of physiotherapy on pain improvement in patients with early knee osteoarthritis at RSU UKI. Int J Med Heal Res. 2021;7(6):52–9. 37. MacKay C, Hawker GA, Jaglal SB. How Do Physical Therapists Approach Management of People With Early Knee Osteoarthritis? A Qualitative Study. Phys Ther [Internet]. 2019;100(2):295–306. Available from: https://doi.org/10.1093/ptj/pzz16438. Hrazdira L, Pilecki Z, Pilecki G. Ultrasound guided application of HA in the soft tissues of the locomotor system in athletes–First experience. Krátká sdělení Botek, M, Stejskal, P, Klimešová, I Vliv normobarické hypoxie na Akt Auton Nerv systému Pilot Stud 191 Drbošalová, V, Stejskal, P, Klugar, M, Krejčíř, V, Hynková, O Charakt klientů komerčn. :205. 39. Ricci M, Micheloni GM, Berti M, Perusi F, Sambugaro E, Vecchini E, et al. Clinical comparison of oral administration and viscosupplementation of hyaluronic acid (HA) in early knee osteoarthritis. Musculoskelet Surg. 2017;101(1):45–9. 40. Wang C-T, Lin Y-T, Chiang B-L, Lin Y-H, Hou S-M. High molecular weight hyaluronic acid down-regulates the gene expression of osteoarthritis-associated cytokines and enzymes in fibroblast-like synoviocytes from patients with early osteoarthritis. Osteoarthr Cartil. 2006;14(12):1237–47. 41. Irrgang JJ, Anderson AF. Development and validation of health-related quality of life measures for the knee. Clin Orthop Relat Res. 2002;402:95–109. 42. Roos EM, Roos HP, Lohmander LS, Ekdahl C, Beynnon BD. Knee Injury and Osteoarthritis Outcome Score (KOOS)—development of a self-administered outcome measure. J Orthop Sport Phys Ther. 1998;28(2):88–96. 43. Perruccio A V, Canizares M, Hawker GA, Roos EM, Maillefert JF, Lohmander SL. Comparative Validity and Responsiveness of the HOOS-PS and KOOS-PS to the WOMAC Physical Function Subscale in Total Joint Replacement for Osteoarthritis. 44. Salavati M, Mazaheri M, Negahban H, Sohani SM, Ebrahimian MR, Ebrahimi I, et al. Validation of a Persian-version of Knee injury and Osteoarthritis Outcome Score (KOOS) in Iranians with knee injuries. Osteoarthr Cartil. 2008;16(10):1178–82. 45. Higgins LD, Taylor MK, Park D, Ghodadra N, Marchant M, Pietrobon R, et al. Reliability and validity of the International Knee Documentation Committee (IKDC) subjective knee form. Jt Bone Spine. 2007;74(6):594–9. 46. Kujala UM, Kaprio J, Sarno S. Osteoarthritis of weight bearing joints of lower limbs in former elite male athletes. Bmj. 1994;308(6923):231–4. 47. Salini V, Pantalone A, Vanni D. Arthroscopy and Sport Injuries. Arthrosc Sport Inj. 2016;433–7. 48. Broglio K. Randomization in clinical trials: Permuted blocks and stratification. JAMA - J Am Med Assoc. 2018;319(21):2223–4. 49. Emanuel EJ, Wendler D, Grady C. What makes clinical research ethical? JAMA. 2000 May;283(20):2701–11. )

[3.	Tiu T, Craig Van Dien MD. Knee overuse disorders.](1. Patel DR, Villalobos A. Evaluation and management of knee pain in young athletes: overuse injuries of the knee. Transl Pediatr [Internet]. 2017 Jul;6(3):190–8. Available from: https://pubmed.ncbi.nlm.nih.gov/287950102. Nicolini AP, de Carvalho RT, Matsuda MM, Filho JS, Cohen M. Common injuries in athletes' knee: Experience of a specialized center. Acta Ortop Bras. 2014;22(3):127–31. 3. Tiu T, Craig Van Dien MD. Knee overuse disorders. 4. Fuller CW, Ekstrand J, Junge A, Andersen TE, Bahr R, Dvorak J, et al. Consensus statement on injury definitions and data collection procedures in studies  of football (soccer) injuries. Br J Sports Med. 2006 Mar;40(3):193–201. 5. Majewski M, Susanne H, Klaus S. Epidemiology of athletic knee injuries: A 10-year study. Knee. 2006 Jun;13(3):184–8. 6. Wilder RP, Sethi S. Overuse injuries: tendinopathies, stress fractures, compartment syndrome, and shin  splints. Clin Sports Med. 2004 Jan;23(1):55–81, vi. 7. Aicale R, Tarantino D, Maffulli N. Overuse injuries in sport: a comprehensive overview. J Orthop Surg Res. 2018 Dec;13(1):309. 8. Yang J, Tibbetts AS, Covassin T, Cheng G, Nayar S, Heiden E. Epidemiology of overuse and acute injuries among competitive collegiate athletes. J Athl Train. 2012;47(2):198–204. 9. Bernetti A, Mangone M, Paoloni M, Di Sante L, Murgia M, Santilli V. Corticosteroid and hyaluronic acid injection therapy in tennis elbow (lateral epicondylalgia). Med Sport. 2014;67(2):289–95. 10. Iolascon G, Gimigliano F, Moretti A, De Sire A, Migliore A, Brandi ML, et al. Early osteoarthritis: How to define, diagnose, and manage. A systematic review. Eur Geriatr Med. 2017;8(5–6):383–96. 11. Drawer S, Fuller CW. Propensity for osteoarthritis and lower limb joint pain in retired professional soccer players. Br J Sports Med. 2001;35(6):402–8. 12. Vannini F, Spalding T, Andriolo L, Berruto M, Denti M, Espregueira-Mendes J, et al. Sport and early osteoarthritis: the role of sport in aetiology, progression and treatment of knee osteoarthritis. Knee Surgery, Sport Traumatol Arthrosc. 2016;24(6):1786–96. 13. Fröhlich S, Pazeller S, Cherati AS, Müller E, Frey WO, Spörri J. Overuse injuries in the knee, back and hip of top elite female alpine skiers during  the off-season preparation period: prevalence, severity and their association with traumatic preinjuries and training load. BMJ open Sport Exerc Med. 2020;6(1):e000892. 14. Rincon GA, Vyas D, Zhou J, Fu FH, Oiestad BE, Holm I, et al. \“ Knee function and prevalence of knee osteoarthritis after anterior cruciate ligament reconstruction: a prospective study with 10 to 15 years of follow-up\”. Letter to the editor. Am J Sports Med. 2011;39(4):NP3-author. 15. Kellgren JH, Lawrence J. Radiological assessment of osteo-arthrosis. Ann Rheum Dis. 1957;16(4):494. 16. Altman R, Asch E, Bloch D, Bole G, Borenstein D, Brandt K, et al. Development of criteria for the classification and reporting of osteoarthritis: classification of osteoarthritis of the knee. Arthritis Rheum Off J Am Coll Rheumatol. 1986;29(8):1039–49. 17. Luyten FP, Denti M, Filardo G, Kon E, Engebretsen L. Definition and classification of early osteoarthritis of the knee. Knee Surgery, Sport Traumatol Arthrosc. 2012;20(3):401–6. 18. Hoemann C, Kandel R, Roberts S, Saris DBF, Creemers L, Mainil-Varlet P, et al. International Cartilage Repair Society (ICRS) Recommended Guidelines for  Histological Endpoints for Cartilage Repair Studies in Animal Models and Clinical Trials. Cartilage. 2011 Apr;2(2):153–72. 19. Hunter DJ, Lo GH, Gale D, Grainger AJ, Guermazi A, Conaghan PG. The reliability of a new scoring system for knee osteoarthritis MRI and the validity of bone marrow lesion assessment: BLOKS (Boston–Leeds Osteoarthritis Knee Score). Ann Rheum Dis. 2008;67(2):206–11. 20. Peterfy CG, Guermazi A, Zaim S, Tirman PFJ, Miaux Y, White D, et al. Whole-organ magnetic resonance imaging score (WORMS) of the knee in osteoarthritis. Osteoarthr Cartil. 2004;12(3):177–90. 21. Demange MK, Sisto M, Rodeo S. Future trends for unicompartmental arthritis of the knee: injectables & stem cells. Clin Sports Med. 2014;33(1):161–74. 22. Leslie M. Knee osteoarthritis management therapies. Pain Manag Nurs. 2000;1(2):51–7. 23. Paoloni M, Bernetti A, Belelli A, Brignoli O, Buoso S, Caputi AP, et al. Appropriateness of clinical and organizational criteria for intra-articular injection therapies in osteoarthritis: A Delphi method consensus initiative among experts in Italy. Ann Ist Super Sanita. 2015;51:131–8. 24. Sandmark H, Hogstedt C, Lewold S, Vingård E. Osteoarthrosis of the knee in men and women in association with overweight, smoking, and hormone therapy. Ann Rheum Dis. 1999;58(3):151–5. 25. Christensen BV, Iuhl IU, Vilbek H, Bülow H, Dreijer NC, Rasmussen HF. Acupuncture treatment of severe knee osteoarthrosis. A long‐term study. Acta Anaesthesiol Scand. 1992;36(6):519–25. 26. de Sire A, Agostini F, Lippi L, Mangone M, Marchese S, Cisari C, et al. Oxygen-Ozone Therapy in the Rehabilitation Field: State of the Art on Mechanisms of  Action, Safety and Effectiveness in Patients with Musculoskeletal Disorders. Biomolecules. 2021 Feb;11(3). 27. Fransen M, McCONNELL S. Land-based exercise for osteoarthritis of the knee: a metaanalysis of randomized controlled trials. J Rheumatol. 2009;36(6):1109–17. 28. Hiemstra LA, Kerslake S, Irving C. Anterior knee pain in the athlete. Clin Sports Med. 2014;33(3):437–59. 29. Filardo G, Kon E, Di Matteo B, Pelotti P, Di Martino A, Marcacci M. Platelet-rich plasma for the treatment of patellar tendinopathy: clinical and imaging findings at medium-term follow-up. Int Orthop [Internet]. 2013;37(8):1583–9. Available from: https://doi.org/10.1007/s00264-013-1972-830. Trojian TH, Concoff AL, Joy SM, Hatzenbuehler JR, Saulsberry WJ, Coleman CI. AMSSM Scientific Statement Concerning Viscosupplementation Injections for Knee  Osteoarthritis: Importance for Individual Patient Outcomes. Clin J Sport Med  Off J Can Acad  Sport Med. 2016 Jan;26(1):1–11. 31. Ammar TY, Pereira TAP, Mistura SLL, Kuhn A, Saggin JI, Lopes OV. Viscosupplementation for treating knee osteoarthrosis: review of the literature. Rev Bras Ortop. 2015;50:489–94. 32. Salini V, Pantalone A, Vanni D, Abate M. Viscosupplementation in Athletes. In: Arthroscopy and Sport Injuries. Springer; 2016. p. 433–7. 33. Erdem Y, Gul D, Akpancar S. Comparison of Intraarticular Injections of Hyaluronic Acid versus Dextrose Applied with Periarticular Prolotherapy in the Treatment of Recreational Athletes with Knee Osteoarthritis. Turkish J Sport Med. 2020;55(1):6–13. 34. Kalkhoven J, Coutts AJ, Impellizzeri FM. 'Training load error' is not a more accurate term than 'overuse' injury. Br J Sports Med [Internet]. 2020 Aug 1;54(15):934 LP – 935. Available from: http://bjsm.bmj.com/content/54/15/934.abstract35. Odole AC, Agbomeji OT, Onyeso OKK, Ojo JO, Odunaiya NA. Perspectives of Nigerian Athletes About Physiotherapy Services in Sports Injury Management: Implications for Rehabilitation. J Sport Rehabil. 2021;1(aop):1–8. 36. Hendrika W, Reswari A. The effect of physiotherapy on pain improvement in patients with early knee osteoarthritis at RSU UKI. Int J Med Heal Res. 2021;7(6):52–9. 37. MacKay C, Hawker GA, Jaglal SB. How Do Physical Therapists Approach Management of People With Early Knee Osteoarthritis? A Qualitative Study. Phys Ther [Internet]. 2019;100(2):295–306. Available from: https://doi.org/10.1093/ptj/pzz16438. Hrazdira L, Pilecki Z, Pilecki G. Ultrasound guided application of HA in the soft tissues of the locomotor system in athletes–First experience. Krátká sdělení Botek, M, Stejskal, P, Klimešová, I Vliv normobarické hypoxie na Akt Auton Nerv systému Pilot Stud 191 Drbošalová, V, Stejskal, P, Klugar, M, Krejčíř, V, Hynková, O Charakt klientů komerčn. :205. 39. Ricci M, Micheloni GM, Berti M, Perusi F, Sambugaro E, Vecchini E, et al. Clinical comparison of oral administration and viscosupplementation of hyaluronic acid (HA) in early knee osteoarthritis. Musculoskelet Surg. 2017;101(1):45–9. 40. Wang C-T, Lin Y-T, Chiang B-L, Lin Y-H, Hou S-M. High molecular weight hyaluronic acid down-regulates the gene expression of osteoarthritis-associated cytokines and enzymes in fibroblast-like synoviocytes from patients with early osteoarthritis. Osteoarthr Cartil. 2006;14(12):1237–47. 41. Irrgang JJ, Anderson AF. Development and validation of health-related quality of life measures for the knee. Clin Orthop Relat Res. 2002;402:95–109. 42. Roos EM, Roos HP, Lohmander LS, Ekdahl C, Beynnon BD. Knee Injury and Osteoarthritis Outcome Score (KOOS)—development of a self-administered outcome measure. J Orthop Sport Phys Ther. 1998;28(2):88–96. 43. Perruccio A V, Canizares M, Hawker GA, Roos EM, Maillefert JF, Lohmander SL. Comparative Validity and Responsiveness of the HOOS-PS and KOOS-PS to the WOMAC Physical Function Subscale in Total Joint Replacement for Osteoarthritis. 44. Salavati M, Mazaheri M, Negahban H, Sohani SM, Ebrahimian MR, Ebrahimi I, et al. Validation of a Persian-version of Knee injury and Osteoarthritis Outcome Score (KOOS) in Iranians with knee injuries. Osteoarthr Cartil. 2008;16(10):1178–82. 45. Higgins LD, Taylor MK, Park D, Ghodadra N, Marchant M, Pietrobon R, et al. Reliability and validity of the International Knee Documentation Committee (IKDC) subjective knee form. Jt Bone Spine. 2007;74(6):594–9. 46. Kujala UM, Kaprio J, Sarno S. Osteoarthritis of weight bearing joints of lower limbs in former elite male athletes. Bmj. 1994;308(6923):231–4. 47. Salini V, Pantalone A, Vanni D. Arthroscopy and Sport Injuries. Arthrosc Sport Inj. 2016;433–7. 48. Broglio K. Randomization in clinical trials: Permuted blocks and stratification. JAMA - J Am Med Assoc. 2018;319(21):2223–4. 49. Emanuel EJ, Wendler D, Grady C. What makes clinical research ethical? JAMA. 2000 May;283(20):2701–11. )

[4.	Fuller CW, Ekstrand J, Junge A, Andersen TE, Bahr R, Dvorak J, et al. Consensus statement on injury definitions and data collection procedures in studies of football (soccer) injuries. Br J Sports Med. 2006 Mar;40(3):193–201.](1. Patel DR, Villalobos A. Evaluation and management of knee pain in young athletes: overuse injuries of the knee. Transl Pediatr [Internet]. 2017 Jul;6(3):190–8. Available from: https://pubmed.ncbi.nlm.nih.gov/287950102. Nicolini AP, de Carvalho RT, Matsuda MM, Filho JS, Cohen M. Common injuries in athletes' knee: Experience of a specialized center. Acta Ortop Bras. 2014;22(3):127–31. 3. Tiu T, Craig Van Dien MD. Knee overuse disorders. 4. Fuller CW, Ekstrand J, Junge A, Andersen TE, Bahr R, Dvorak J, et al. Consensus statement on injury definitions and data collection procedures in studies  of football (soccer) injuries. Br J Sports Med. 2006 Mar;40(3):193–201. 5. Majewski M, Susanne H, Klaus S. Epidemiology of athletic knee injuries: A 10-year study. Knee. 2006 Jun;13(3):184–8. 6. Wilder RP, Sethi S. Overuse injuries: tendinopathies, stress fractures, compartment syndrome, and shin  splints. Clin Sports Med. 2004 Jan;23(1):55–81, vi. 7. Aicale R, Tarantino D, Maffulli N. Overuse injuries in sport: a comprehensive overview. J Orthop Surg Res. 2018 Dec;13(1):309. 8. Yang J, Tibbetts AS, Covassin T, Cheng G, Nayar S, Heiden E. Epidemiology of overuse and acute injuries among competitive collegiate athletes. J Athl Train. 2012;47(2):198–204. 9. Bernetti A, Mangone M, Paoloni M, Di Sante L, Murgia M, Santilli V. Corticosteroid and hyaluronic acid injection therapy in tennis elbow (lateral epicondylalgia). Med Sport. 2014;67(2):289–95. 10. Iolascon G, Gimigliano F, Moretti A, De Sire A, Migliore A, Brandi ML, et al. Early osteoarthritis: How to define, diagnose, and manage. A systematic review. Eur Geriatr Med. 2017;8(5–6):383–96. 11. Drawer S, Fuller CW. Propensity for osteoarthritis and lower limb joint pain in retired professional soccer players. Br J Sports Med. 2001;35(6):402–8. 12. Vannini F, Spalding T, Andriolo L, Berruto M, Denti M, Espregueira-Mendes J, et al. Sport and early osteoarthritis: the role of sport in aetiology, progression and treatment of knee osteoarthritis. Knee Surgery, Sport Traumatol Arthrosc. 2016;24(6):1786–96. 13. Fröhlich S, Pazeller S, Cherati AS, Müller E, Frey WO, Spörri J. Overuse injuries in the knee, back and hip of top elite female alpine skiers during  the off-season preparation period: prevalence, severity and their association with traumatic preinjuries and training load. BMJ open Sport Exerc Med. 2020;6(1):e000892. 14. Rincon GA, Vyas D, Zhou J, Fu FH, Oiestad BE, Holm I, et al. \“ Knee function and prevalence of knee osteoarthritis after anterior cruciate ligament reconstruction: a prospective study with 10 to 15 years of follow-up\”. Letter to the editor. Am J Sports Med. 2011;39(4):NP3-author. 15. Kellgren JH, Lawrence J. Radiological assessment of osteo-arthrosis. Ann Rheum Dis. 1957;16(4):494. 16. Altman R, Asch E, Bloch D, Bole G, Borenstein D, Brandt K, et al. Development of criteria for the classification and reporting of osteoarthritis: classification of osteoarthritis of the knee. Arthritis Rheum Off J Am Coll Rheumatol. 1986;29(8):1039–49. 17. Luyten FP, Denti M, Filardo G, Kon E, Engebretsen L. Definition and classification of early osteoarthritis of the knee. Knee Surgery, Sport Traumatol Arthrosc. 2012;20(3):401–6. 18. Hoemann C, Kandel R, Roberts S, Saris DBF, Creemers L, Mainil-Varlet P, et al. International Cartilage Repair Society (ICRS) Recommended Guidelines for  Histological Endpoints for Cartilage Repair Studies in Animal Models and Clinical Trials. Cartilage. 2011 Apr;2(2):153–72. 19. Hunter DJ, Lo GH, Gale D, Grainger AJ, Guermazi A, Conaghan PG. The reliability of a new scoring system for knee osteoarthritis MRI and the validity of bone marrow lesion assessment: BLOKS (Boston–Leeds Osteoarthritis Knee Score). Ann Rheum Dis. 2008;67(2):206–11. 20. Peterfy CG, Guermazi A, Zaim S, Tirman PFJ, Miaux Y, White D, et al. Whole-organ magnetic resonance imaging score (WORMS) of the knee in osteoarthritis. Osteoarthr Cartil. 2004;12(3):177–90. 21. Demange MK, Sisto M, Rodeo S. Future trends for unicompartmental arthritis of the knee: injectables & stem cells. Clin Sports Med. 2014;33(1):161–74. 22. Leslie M. Knee osteoarthritis management therapies. Pain Manag Nurs. 2000;1(2):51–7. 23. Paoloni M, Bernetti A, Belelli A, Brignoli O, Buoso S, Caputi AP, et al. Appropriateness of clinical and organizational criteria for intra-articular injection therapies in osteoarthritis: A Delphi method consensus initiative among experts in Italy. Ann Ist Super Sanita. 2015;51:131–8. 24. Sandmark H, Hogstedt C, Lewold S, Vingård E. Osteoarthrosis of the knee in men and women in association with overweight, smoking, and hormone therapy. Ann Rheum Dis. 1999;58(3):151–5. 25. Christensen BV, Iuhl IU, Vilbek H, Bülow H, Dreijer NC, Rasmussen HF. Acupuncture treatment of severe knee osteoarthrosis. A long‐term study. Acta Anaesthesiol Scand. 1992;36(6):519–25. 26. de Sire A, Agostini F, Lippi L, Mangone M, Marchese S, Cisari C, et al. Oxygen-Ozone Therapy in the Rehabilitation Field: State of the Art on Mechanisms of  Action, Safety and Effectiveness in Patients with Musculoskeletal Disorders. Biomolecules. 2021 Feb;11(3). 27. Fransen M, McCONNELL S. Land-based exercise for osteoarthritis of the knee: a metaanalysis of randomized controlled trials. J Rheumatol. 2009;36(6):1109–17. 28. Hiemstra LA, Kerslake S, Irving C. Anterior knee pain in the athlete. Clin Sports Med. 2014;33(3):437–59. 29. Filardo G, Kon E, Di Matteo B, Pelotti P, Di Martino A, Marcacci M. Platelet-rich plasma for the treatment of patellar tendinopathy: clinical and imaging findings at medium-term follow-up. Int Orthop [Internet]. 2013;37(8):1583–9. Available from: https://doi.org/10.1007/s00264-013-1972-830. Trojian TH, Concoff AL, Joy SM, Hatzenbuehler JR, Saulsberry WJ, Coleman CI. AMSSM Scientific Statement Concerning Viscosupplementation Injections for Knee  Osteoarthritis: Importance for Individual Patient Outcomes. Clin J Sport Med  Off J Can Acad  Sport Med. 2016 Jan;26(1):1–11. 31. Ammar TY, Pereira TAP, Mistura SLL, Kuhn A, Saggin JI, Lopes OV. Viscosupplementation for treating knee osteoarthrosis: review of the literature. Rev Bras Ortop. 2015;50:489–94. 32. Salini V, Pantalone A, Vanni D, Abate M. Viscosupplementation in Athletes. In: Arthroscopy and Sport Injuries. Springer; 2016. p. 433–7. 33. Erdem Y, Gul D, Akpancar S. Comparison of Intraarticular Injections of Hyaluronic Acid versus Dextrose Applied with Periarticular Prolotherapy in the Treatment of Recreational Athletes with Knee Osteoarthritis. Turkish J Sport Med. 2020;55(1):6–13. 34. Kalkhoven J, Coutts AJ, Impellizzeri FM. 'Training load error' is not a more accurate term than 'overuse' injury. Br J Sports Med [Internet]. 2020 Aug 1;54(15):934 LP – 935. Available from: http://bjsm.bmj.com/content/54/15/934.abstract35. Odole AC, Agbomeji OT, Onyeso OKK, Ojo JO, Odunaiya NA. Perspectives of Nigerian Athletes About Physiotherapy Services in Sports Injury Management: Implications for Rehabilitation. J Sport Rehabil. 2021;1(aop):1–8. 36. Hendrika W, Reswari A. The effect of physiotherapy on pain improvement in patients with early knee osteoarthritis at RSU UKI. Int J Med Heal Res. 2021;7(6):52–9. 37. MacKay C, Hawker GA, Jaglal SB. How Do Physical Therapists Approach Management of People With Early Knee Osteoarthritis? A Qualitative Study. Phys Ther [Internet]. 2019;100(2):295–306. Available from: https://doi.org/10.1093/ptj/pzz16438. Hrazdira L, Pilecki Z, Pilecki G. Ultrasound guided application of HA in the soft tissues of the locomotor system in athletes–First experience. Krátká sdělení Botek, M, Stejskal, P, Klimešová, I Vliv normobarické hypoxie na Akt Auton Nerv systému Pilot Stud 191 Drbošalová, V, Stejskal, P, Klugar, M, Krejčíř, V, Hynková, O Charakt klientů komerčn. :205. 39. Ricci M, Micheloni GM, Berti M, Perusi F, Sambugaro E, Vecchini E, et al. Clinical comparison of oral administration and viscosupplementation of hyaluronic acid (HA) in early knee osteoarthritis. Musculoskelet Surg. 2017;101(1):45–9. 40. Wang C-T, Lin Y-T, Chiang B-L, Lin Y-H, Hou S-M. High molecular weight hyaluronic acid down-regulates the gene expression of osteoarthritis-associated cytokines and enzymes in fibroblast-like synoviocytes from patients with early osteoarthritis. Osteoarthr Cartil. 2006;14(12):1237–47. 41. Irrgang JJ, Anderson AF. Development and validation of health-related quality of life measures for the knee. Clin Orthop Relat Res. 2002;402:95–109. 42. Roos EM, Roos HP, Lohmander LS, Ekdahl C, Beynnon BD. Knee Injury and Osteoarthritis Outcome Score (KOOS)—development of a self-administered outcome measure. J Orthop Sport Phys Ther. 1998;28(2):88–96. 43. Perruccio A V, Canizares M, Hawker GA, Roos EM, Maillefert JF, Lohmander SL. Comparative Validity and Responsiveness of the HOOS-PS and KOOS-PS to the WOMAC Physical Function Subscale in Total Joint Replacement for Osteoarthritis. 44. Salavati M, Mazaheri M, Negahban H, Sohani SM, Ebrahimian MR, Ebrahimi I, et al. Validation of a Persian-version of Knee injury and Osteoarthritis Outcome Score (KOOS) in Iranians with knee injuries. Osteoarthr Cartil. 2008;16(10):1178–82. 45. Higgins LD, Taylor MK, Park D, Ghodadra N, Marchant M, Pietrobon R, et al. Reliability and validity of the International Knee Documentation Committee (IKDC) subjective knee form. Jt Bone Spine. 2007;74(6):594–9. 46. Kujala UM, Kaprio J, Sarno S. Osteoarthritis of weight bearing joints of lower limbs in former elite male athletes. Bmj. 1994;308(6923):231–4. 47. Salini V, Pantalone A, Vanni D. Arthroscopy and Sport Injuries. Arthrosc Sport Inj. 2016;433–7. 48. Broglio K. Randomization in clinical trials: Permuted blocks and stratification. JAMA - J Am Med Assoc. 2018;319(21):2223–4. 49. Emanuel EJ, Wendler D, Grady C. What makes clinical research ethical? JAMA. 2000 May;283(20):2701–11. )

[5.	Majewski M, Susanne H, Klaus S. Epidemiology of athletic knee injuries: A 10-year study. Knee. 2006 Jun;13(3):184–8.](1. Patel DR, Villalobos A. Evaluation and management of knee pain in young athletes: overuse injuries of the knee. Transl Pediatr [Internet]. 2017 Jul;6(3):190–8. Available from: https://pubmed.ncbi.nlm.nih.gov/287950102. Nicolini AP, de Carvalho RT, Matsuda MM, Filho JS, Cohen M. Common injuries in athletes' knee: Experience of a specialized center. Acta Ortop Bras. 2014;22(3):127–31. 3. Tiu T, Craig Van Dien MD. Knee overuse disorders. 4. Fuller CW, Ekstrand J, Junge A, Andersen TE, Bahr R, Dvorak J, et al. Consensus statement on injury definitions and data collection procedures in studies  of football (soccer) injuries. Br J Sports Med. 2006 Mar;40(3):193–201. 5. Majewski M, Susanne H, Klaus S. Epidemiology of athletic knee injuries: A 10-year study. Knee. 2006 Jun;13(3):184–8. 6. Wilder RP, Sethi S. Overuse injuries: tendinopathies, stress fractures, compartment syndrome, and shin  splints. Clin Sports Med. 2004 Jan;23(1):55–81, vi. 7. Aicale R, Tarantino D, Maffulli N. Overuse injuries in sport: a comprehensive overview. J Orthop Surg Res. 2018 Dec;13(1):309. 8. Yang J, Tibbetts AS, Covassin T, Cheng G, Nayar S, Heiden E. Epidemiology of overuse and acute injuries among competitive collegiate athletes. J Athl Train. 2012;47(2):198–204. 9. Bernetti A, Mangone M, Paoloni M, Di Sante L, Murgia M, Santilli V. Corticosteroid and hyaluronic acid injection therapy in tennis elbow (lateral epicondylalgia). Med Sport. 2014;67(2):289–95. 10. Iolascon G, Gimigliano F, Moretti A, De Sire A, Migliore A, Brandi ML, et al. Early osteoarthritis: How to define, diagnose, and manage. A systematic review. Eur Geriatr Med. 2017;8(5–6):383–96. 11. Drawer S, Fuller CW. Propensity for osteoarthritis and lower limb joint pain in retired professional soccer players. Br J Sports Med. 2001;35(6):402–8. 12. Vannini F, Spalding T, Andriolo L, Berruto M, Denti M, Espregueira-Mendes J, et al. Sport and early osteoarthritis: the role of sport in aetiology, progression and treatment of knee osteoarthritis. Knee Surgery, Sport Traumatol Arthrosc. 2016;24(6):1786–96. 13. Fröhlich S, Pazeller S, Cherati AS, Müller E, Frey WO, Spörri J. Overuse injuries in the knee, back and hip of top elite female alpine skiers during  the off-season preparation period: prevalence, severity and their association with traumatic preinjuries and training load. BMJ open Sport Exerc Med. 2020;6(1):e000892. 14. Rincon GA, Vyas D, Zhou J, Fu FH, Oiestad BE, Holm I, et al. \“ Knee function and prevalence of knee osteoarthritis after anterior cruciate ligament reconstruction: a prospective study with 10 to 15 years of follow-up\”. Letter to the editor. Am J Sports Med. 2011;39(4):NP3-author. 15. Kellgren JH, Lawrence J. Radiological assessment of osteo-arthrosis. Ann Rheum Dis. 1957;16(4):494. 16. Altman R, Asch E, Bloch D, Bole G, Borenstein D, Brandt K, et al. Development of criteria for the classification and reporting of osteoarthritis: classification of osteoarthritis of the knee. Arthritis Rheum Off J Am Coll Rheumatol. 1986;29(8):1039–49. 17. Luyten FP, Denti M, Filardo G, Kon E, Engebretsen L. Definition and classification of early osteoarthritis of the knee. Knee Surgery, Sport Traumatol Arthrosc. 2012;20(3):401–6. 18. Hoemann C, Kandel R, Roberts S, Saris DBF, Creemers L, Mainil-Varlet P, et al. International Cartilage Repair Society (ICRS) Recommended Guidelines for  Histological Endpoints for Cartilage Repair Studies in Animal Models and Clinical Trials. Cartilage. 2011 Apr;2(2):153–72. 19. Hunter DJ, Lo GH, Gale D, Grainger AJ, Guermazi A, Conaghan PG. The reliability of a new scoring system for knee osteoarthritis MRI and the validity of bone marrow lesion assessment: BLOKS (Boston–Leeds Osteoarthritis Knee Score). Ann Rheum Dis. 2008;67(2):206–11. 20. Peterfy CG, Guermazi A, Zaim S, Tirman PFJ, Miaux Y, White D, et al. Whole-organ magnetic resonance imaging score (WORMS) of the knee in osteoarthritis. Osteoarthr Cartil. 2004;12(3):177–90. 21. Demange MK, Sisto M, Rodeo S. Future trends for unicompartmental arthritis of the knee: injectables & stem cells. Clin Sports Med. 2014;33(1):161–74. 22. Leslie M. Knee osteoarthritis management therapies. Pain Manag Nurs. 2000;1(2):51–7. 23. Paoloni M, Bernetti A, Belelli A, Brignoli O, Buoso S, Caputi AP, et al. Appropriateness of clinical and organizational criteria for intra-articular injection therapies in osteoarthritis: A Delphi method consensus initiative among experts in Italy. Ann Ist Super Sanita. 2015;51:131–8. 24. Sandmark H, Hogstedt C, Lewold S, Vingård E. Osteoarthrosis of the knee in men and women in association with overweight, smoking, and hormone therapy. Ann Rheum Dis. 1999;58(3):151–5. 25. Christensen BV, Iuhl IU, Vilbek H, Bülow H, Dreijer NC, Rasmussen HF. Acupuncture treatment of severe knee osteoarthrosis. A long‐term study. Acta Anaesthesiol Scand. 1992;36(6):519–25. 26. de Sire A, Agostini F, Lippi L, Mangone M, Marchese S, Cisari C, et al. Oxygen-Ozone Therapy in the Rehabilitation Field: State of the Art on Mechanisms of  Action, Safety and Effectiveness in Patients with Musculoskeletal Disorders. Biomolecules. 2021 Feb;11(3). 27. Fransen M, McCONNELL S. Land-based exercise for osteoarthritis of the knee: a metaanalysis of randomized controlled trials. J Rheumatol. 2009;36(6):1109–17. 28. Hiemstra LA, Kerslake S, Irving C. Anterior knee pain in the athlete. Clin Sports Med. 2014;33(3):437–59. 29. Filardo G, Kon E, Di Matteo B, Pelotti P, Di Martino A, Marcacci M. Platelet-rich plasma for the treatment of patellar tendinopathy: clinical and imaging findings at medium-term follow-up. Int Orthop [Internet]. 2013;37(8):1583–9. Available from: https://doi.org/10.1007/s00264-013-1972-830. Trojian TH, Concoff AL, Joy SM, Hatzenbuehler JR, Saulsberry WJ, Coleman CI. AMSSM Scientific Statement Concerning Viscosupplementation Injections for Knee  Osteoarthritis: Importance for Individual Patient Outcomes. Clin J Sport Med  Off J Can Acad  Sport Med. 2016 Jan;26(1):1–11. 31. Ammar TY, Pereira TAP, Mistura SLL, Kuhn A, Saggin JI, Lopes OV. Viscosupplementation for treating knee osteoarthrosis: review of the literature. Rev Bras Ortop. 2015;50:489–94. 32. Salini V, Pantalone A, Vanni D, Abate M. Viscosupplementation in Athletes. In: Arthroscopy and Sport Injuries. Springer; 2016. p. 433–7. 33. Erdem Y, Gul D, Akpancar S. Comparison of Intraarticular Injections of Hyaluronic Acid versus Dextrose Applied with Periarticular Prolotherapy in the Treatment of Recreational Athletes with Knee Osteoarthritis. Turkish J Sport Med. 2020;55(1):6–13. 34. Kalkhoven J, Coutts AJ, Impellizzeri FM. 'Training load error' is not a more accurate term than 'overuse' injury. Br J Sports Med [Internet]. 2020 Aug 1;54(15):934 LP – 935. Available from: http://bjsm.bmj.com/content/54/15/934.abstract35. Odole AC, Agbomeji OT, Onyeso OKK, Ojo JO, Odunaiya NA. Perspectives of Nigerian Athletes About Physiotherapy Services in Sports Injury Management: Implications for Rehabilitation. J Sport Rehabil. 2021;1(aop):1–8. 36. Hendrika W, Reswari A. The effect of physiotherapy on pain improvement in patients with early knee osteoarthritis at RSU UKI. Int J Med Heal Res. 2021;7(6):52–9. 37. MacKay C, Hawker GA, Jaglal SB. How Do Physical Therapists Approach Management of People With Early Knee Osteoarthritis? A Qualitative Study. Phys Ther [Internet]. 2019;100(2):295–306. Available from: https://doi.org/10.1093/ptj/pzz16438. Hrazdira L, Pilecki Z, Pilecki G. Ultrasound guided application of HA in the soft tissues of the locomotor system in athletes–First experience. Krátká sdělení Botek, M, Stejskal, P, Klimešová, I Vliv normobarické hypoxie na Akt Auton Nerv systému Pilot Stud 191 Drbošalová, V, Stejskal, P, Klugar, M, Krejčíř, V, Hynková, O Charakt klientů komerčn. :205. 39. Ricci M, Micheloni GM, Berti M, Perusi F, Sambugaro E, Vecchini E, et al. Clinical comparison of oral administration and viscosupplementation of hyaluronic acid (HA) in early knee osteoarthritis. Musculoskelet Surg. 2017;101(1):45–9. 40. Wang C-T, Lin Y-T, Chiang B-L, Lin Y-H, Hou S-M. High molecular weight hyaluronic acid down-regulates the gene expression of osteoarthritis-associated cytokines and enzymes in fibroblast-like synoviocytes from patients with early osteoarthritis. Osteoarthr Cartil. 2006;14(12):1237–47. 41. Irrgang JJ, Anderson AF. Development and validation of health-related quality of life measures for the knee. Clin Orthop Relat Res. 2002;402:95–109. 42. Roos EM, Roos HP, Lohmander LS, Ekdahl C, Beynnon BD. Knee Injury and Osteoarthritis Outcome Score (KOOS)—development of a self-administered outcome measure. J Orthop Sport Phys Ther. 1998;28(2):88–96. 43. Perruccio A V, Canizares M, Hawker GA, Roos EM, Maillefert JF, Lohmander SL. Comparative Validity and Responsiveness of the HOOS-PS and KOOS-PS to the WOMAC Physical Function Subscale in Total Joint Replacement for Osteoarthritis. 44. Salavati M, Mazaheri M, Negahban H, Sohani SM, Ebrahimian MR, Ebrahimi I, et al. Validation of a Persian-version of Knee injury and Osteoarthritis Outcome Score (KOOS) in Iranians with knee injuries. Osteoarthr Cartil. 2008;16(10):1178–82. 45. Higgins LD, Taylor MK, Park D, Ghodadra N, Marchant M, Pietrobon R, et al. Reliability and validity of the International Knee Documentation Committee (IKDC) subjective knee form. Jt Bone Spine. 2007;74(6):594–9. 46. Kujala UM, Kaprio J, Sarno S. Osteoarthritis of weight bearing joints of lower limbs in former elite male athletes. Bmj. 1994;308(6923):231–4. 47. Salini V, Pantalone A, Vanni D. Arthroscopy and Sport Injuries. Arthrosc Sport Inj. 2016;433–7. 48. Broglio K. Randomization in clinical trials: Permuted blocks and stratification. JAMA - J Am Med Assoc. 2018;319(21):2223–4. 49. Emanuel EJ, Wendler D, Grady C. What makes clinical research ethical? JAMA. 2000 May;283(20):2701–11. )

[6.	Wilder RP, Sethi S. Overuse injuries: tendinopathies, stress fractures, compartment syndrome, and shin splints. Clin Sports Med. 2004 Jan;23(1):55–81, vi.](1. Patel DR, Villalobos A. Evaluation and management of knee pain in young athletes: overuse injuries of the knee. Transl Pediatr [Internet]. 2017 Jul;6(3):190–8. Available from: https://pubmed.ncbi.nlm.nih.gov/287950102. Nicolini AP, de Carvalho RT, Matsuda MM, Filho JS, Cohen M. Common injuries in athletes' knee: Experience of a specialized center. Acta Ortop Bras. 2014;22(3):127–31. 3. Tiu T, Craig Van Dien MD. Knee overuse disorders. 4. Fuller CW, Ekstrand J, Junge A, Andersen TE, Bahr R, Dvorak J, et al. Consensus statement on injury definitions and data collection procedures in studies  of football (soccer) injuries. Br J Sports Med. 2006 Mar;40(3):193–201. 5. Majewski M, Susanne H, Klaus S. Epidemiology of athletic knee injuries: A 10-year study. Knee. 2006 Jun;13(3):184–8. 6. Wilder RP, Sethi S. Overuse injuries: tendinopathies, stress fractures, compartment syndrome, and shin  splints. Clin Sports Med. 2004 Jan;23(1):55–81, vi. 7. Aicale R, Tarantino D, Maffulli N. Overuse injuries in sport: a comprehensive overview. J Orthop Surg Res. 2018 Dec;13(1):309. 8. Yang J, Tibbetts AS, Covassin T, Cheng G, Nayar S, Heiden E. Epidemiology of overuse and acute injuries among competitive collegiate athletes. J Athl Train. 2012;47(2):198–204. 9. Bernetti A, Mangone M, Paoloni M, Di Sante L, Murgia M, Santilli V. Corticosteroid and hyaluronic acid injection therapy in tennis elbow (lateral epicondylalgia). Med Sport. 2014;67(2):289–95. 10. Iolascon G, Gimigliano F, Moretti A, De Sire A, Migliore A, Brandi ML, et al. Early osteoarthritis: How to define, diagnose, and manage. A systematic review. Eur Geriatr Med. 2017;8(5–6):383–96. 11. Drawer S, Fuller CW. Propensity for osteoarthritis and lower limb joint pain in retired professional soccer players. Br J Sports Med. 2001;35(6):402–8. 12. Vannini F, Spalding T, Andriolo L, Berruto M, Denti M, Espregueira-Mendes J, et al. Sport and early osteoarthritis: the role of sport in aetiology, progression and treatment of knee osteoarthritis. Knee Surgery, Sport Traumatol Arthrosc. 2016;24(6):1786–96. 13. Fröhlich S, Pazeller S, Cherati AS, Müller E, Frey WO, Spörri J. Overuse injuries in the knee, back and hip of top elite female alpine skiers during  the off-season preparation period: prevalence, severity and their association with traumatic preinjuries and training load. BMJ open Sport Exerc Med. 2020;6(1):e000892. 14. Rincon GA, Vyas D, Zhou J, Fu FH, Oiestad BE, Holm I, et al. \“ Knee function and prevalence of knee osteoarthritis after anterior cruciate ligament reconstruction: a prospective study with 10 to 15 years of follow-up\”. Letter to the editor. Am J Sports Med. 2011;39(4):NP3-author. 15. Kellgren JH, Lawrence J. Radiological assessment of osteo-arthrosis. Ann Rheum Dis. 1957;16(4):494. 16. Altman R, Asch E, Bloch D, Bole G, Borenstein D, Brandt K, et al. Development of criteria for the classification and reporting of osteoarthritis: classification of osteoarthritis of the knee. Arthritis Rheum Off J Am Coll Rheumatol. 1986;29(8):1039–49. 17. Luyten FP, Denti M, Filardo G, Kon E, Engebretsen L. Definition and classification of early osteoarthritis of the knee. Knee Surgery, Sport Traumatol Arthrosc. 2012;20(3):401–6. 18. Hoemann C, Kandel R, Roberts S, Saris DBF, Creemers L, Mainil-Varlet P, et al. International Cartilage Repair Society (ICRS) Recommended Guidelines for  Histological Endpoints for Cartilage Repair Studies in Animal Models and Clinical Trials. Cartilage. 2011 Apr;2(2):153–72. 19. Hunter DJ, Lo GH, Gale D, Grainger AJ, Guermazi A, Conaghan PG. The reliability of a new scoring system for knee osteoarthritis MRI and the validity of bone marrow lesion assessment: BLOKS (Boston–Leeds Osteoarthritis Knee Score). Ann Rheum Dis. 2008;67(2):206–11. 20. Peterfy CG, Guermazi A, Zaim S, Tirman PFJ, Miaux Y, White D, et al. Whole-organ magnetic resonance imaging score (WORMS) of the knee in osteoarthritis. Osteoarthr Cartil. 2004;12(3):177–90. 21. Demange MK, Sisto M, Rodeo S. Future trends for unicompartmental arthritis of the knee: injectables & stem cells. Clin Sports Med. 2014;33(1):161–74. 22. Leslie M. Knee osteoarthritis management therapies. Pain Manag Nurs. 2000;1(2):51–7. 23. Paoloni M, Bernetti A, Belelli A, Brignoli O, Buoso S, Caputi AP, et al. Appropriateness of clinical and organizational criteria for intra-articular injection therapies in osteoarthritis: A Delphi method consensus initiative among experts in Italy. Ann Ist Super Sanita. 2015;51:131–8. 24. Sandmark H, Hogstedt C, Lewold S, Vingård E. Osteoarthrosis of the knee in men and women in association with overweight, smoking, and hormone therapy. Ann Rheum Dis. 1999;58(3):151–5. 25. Christensen BV, Iuhl IU, Vilbek H, Bülow H, Dreijer NC, Rasmussen HF. Acupuncture treatment of severe knee osteoarthrosis. A long‐term study. Acta Anaesthesiol Scand. 1992;36(6):519–25. 26. de Sire A, Agostini F, Lippi L, Mangone M, Marchese S, Cisari C, et al. Oxygen-Ozone Therapy in the Rehabilitation Field: State of the Art on Mechanisms of  Action, Safety and Effectiveness in Patients with Musculoskeletal Disorders. Biomolecules. 2021 Feb;11(3). 27. Fransen M, McCONNELL S. Land-based exercise for osteoarthritis of the knee: a metaanalysis of randomized controlled trials. J Rheumatol. 2009;36(6):1109–17. 28. Hiemstra LA, Kerslake S, Irving C. Anterior knee pain in the athlete. Clin Sports Med. 2014;33(3):437–59. 29. Filardo G, Kon E, Di Matteo B, Pelotti P, Di Martino A, Marcacci M. Platelet-rich plasma for the treatment of patellar tendinopathy: clinical and imaging findings at medium-term follow-up. Int Orthop [Internet]. 2013;37(8):1583–9. Available from: https://doi.org/10.1007/s00264-013-1972-830. Trojian TH, Concoff AL, Joy SM, Hatzenbuehler JR, Saulsberry WJ, Coleman CI. AMSSM Scientific Statement Concerning Viscosupplementation Injections for Knee  Osteoarthritis: Importance for Individual Patient Outcomes. Clin J Sport Med  Off J Can Acad  Sport Med. 2016 Jan;26(1):1–11. 31. Ammar TY, Pereira TAP, Mistura SLL, Kuhn A, Saggin JI, Lopes OV. Viscosupplementation for treating knee osteoarthrosis: review of the literature. Rev Bras Ortop. 2015;50:489–94. 32. Salini V, Pantalone A, Vanni D, Abate M. Viscosupplementation in Athletes. In: Arthroscopy and Sport Injuries. Springer; 2016. p. 433–7. 33. Erdem Y, Gul D, Akpancar S. Comparison of Intraarticular Injections of Hyaluronic Acid versus Dextrose Applied with Periarticular Prolotherapy in the Treatment of Recreational Athletes with Knee Osteoarthritis. Turkish J Sport Med. 2020;55(1):6–13. 34. Kalkhoven J, Coutts AJ, Impellizzeri FM. 'Training load error' is not a more accurate term than 'overuse' injury. Br J Sports Med [Internet]. 2020 Aug 1;54(15):934 LP – 935. Available from: http://bjsm.bmj.com/content/54/15/934.abstract35. Odole AC, Agbomeji OT, Onyeso OKK, Ojo JO, Odunaiya NA. Perspectives of Nigerian Athletes About Physiotherapy Services in Sports Injury Management: Implications for Rehabilitation. J Sport Rehabil. 2021;1(aop):1–8. 36. Hendrika W, Reswari A. The effect of physiotherapy on pain improvement in patients with early knee osteoarthritis at RSU UKI. Int J Med Heal Res. 2021;7(6):52–9. 37. MacKay C, Hawker GA, Jaglal SB. How Do Physical Therapists Approach Management of People With Early Knee Osteoarthritis? A Qualitative Study. Phys Ther [Internet]. 2019;100(2):295–306. Available from: https://doi.org/10.1093/ptj/pzz16438. Hrazdira L, Pilecki Z, Pilecki G. Ultrasound guided application of HA in the soft tissues of the locomotor system in athletes–First experience. Krátká sdělení Botek, M, Stejskal, P, Klimešová, I Vliv normobarické hypoxie na Akt Auton Nerv systému Pilot Stud 191 Drbošalová, V, Stejskal, P, Klugar, M, Krejčíř, V, Hynková, O Charakt klientů komerčn. :205. 39. Ricci M, Micheloni GM, Berti M, Perusi F, Sambugaro E, Vecchini E, et al. Clinical comparison of oral administration and viscosupplementation of hyaluronic acid (HA) in early knee osteoarthritis. Musculoskelet Surg. 2017;101(1):45–9. 40. Wang C-T, Lin Y-T, Chiang B-L, Lin Y-H, Hou S-M. High molecular weight hyaluronic acid down-regulates the gene expression of osteoarthritis-associated cytokines and enzymes in fibroblast-like synoviocytes from patients with early osteoarthritis. Osteoarthr Cartil. 2006;14(12):1237–47. 41. Irrgang JJ, Anderson AF. Development and validation of health-related quality of life measures for the knee. Clin Orthop Relat Res. 2002;402:95–109. 42. Roos EM, Roos HP, Lohmander LS, Ekdahl C, Beynnon BD. Knee Injury and Osteoarthritis Outcome Score (KOOS)—development of a self-administered outcome measure. J Orthop Sport Phys Ther. 1998;28(2):88–96. 43. Perruccio A V, Canizares M, Hawker GA, Roos EM, Maillefert JF, Lohmander SL. Comparative Validity and Responsiveness of the HOOS-PS and KOOS-PS to the WOMAC Physical Function Subscale in Total Joint Replacement for Osteoarthritis. 44. Salavati M, Mazaheri M, Negahban H, Sohani SM, Ebrahimian MR, Ebrahimi I, et al. Validation of a Persian-version of Knee injury and Osteoarthritis Outcome Score (KOOS) in Iranians with knee injuries. Osteoarthr Cartil. 2008;16(10):1178–82. 45. Higgins LD, Taylor MK, Park D, Ghodadra N, Marchant M, Pietrobon R, et al. Reliability and validity of the International Knee Documentation Committee (IKDC) subjective knee form. Jt Bone Spine. 2007;74(6):594–9. 46. Kujala UM, Kaprio J, Sarno S. Osteoarthritis of weight bearing joints of lower limbs in former elite male athletes. Bmj. 1994;308(6923):231–4. 47. Salini V, Pantalone A, Vanni D. Arthroscopy and Sport Injuries. Arthrosc Sport Inj. 2016;433–7. 48. Broglio K. Randomization in clinical trials: Permuted blocks and stratification. JAMA - J Am Med Assoc. 2018;319(21):2223–4. 49. Emanuel EJ, Wendler D, Grady C. What makes clinical research ethical? JAMA. 2000 May;283(20):2701–11. )

[7.	Aicale R, Tarantino D, Maffulli N. Overuse injuries in sport: a comprehensive overview. J Orthop Surg Res. 2018 Dec;13(1):309.](1. Patel DR, Villalobos A. Evaluation and management of knee pain in young athletes: overuse injuries of the knee. Transl Pediatr [Internet]. 2017 Jul;6(3):190–8. Available from: https://pubmed.ncbi.nlm.nih.gov/287950102. Nicolini AP, de Carvalho RT, Matsuda MM, Filho JS, Cohen M. Common injuries in athletes' knee: Experience of a specialized center. Acta Ortop Bras. 2014;22(3):127–31. 3. Tiu T, Craig Van Dien MD. Knee overuse disorders. 4. Fuller CW, Ekstrand J, Junge A, Andersen TE, Bahr R, Dvorak J, et al. Consensus statement on injury definitions and data collection procedures in studies  of football (soccer) injuries. Br J Sports Med. 2006 Mar;40(3):193–201. 5. Majewski M, Susanne H, Klaus S. Epidemiology of athletic knee injuries: A 10-year study. Knee. 2006 Jun;13(3):184–8. 6. Wilder RP, Sethi S. Overuse injuries: tendinopathies, stress fractures, compartment syndrome, and shin  splints. Clin Sports Med. 2004 Jan;23(1):55–81, vi. 7. Aicale R, Tarantino D, Maffulli N. Overuse injuries in sport: a comprehensive overview. J Orthop Surg Res. 2018 Dec;13(1):309. 8. Yang J, Tibbetts AS, Covassin T, Cheng G, Nayar S, Heiden E. Epidemiology of overuse and acute injuries among competitive collegiate athletes. J Athl Train. 2012;47(2):198–204. 9. Bernetti A, Mangone M, Paoloni M, Di Sante L, Murgia M, Santilli V. Corticosteroid and hyaluronic acid injection therapy in tennis elbow (lateral epicondylalgia). Med Sport. 2014;67(2):289–95. 10. Iolascon G, Gimigliano F, Moretti A, De Sire A, Migliore A, Brandi ML, et al. Early osteoarthritis: How to define, diagnose, and manage. A systematic review. Eur Geriatr Med. 2017;8(5–6):383–96. 11. Drawer S, Fuller CW. Propensity for osteoarthritis and lower limb joint pain in retired professional soccer players. Br J Sports Med. 2001;35(6):402–8. 12. Vannini F, Spalding T, Andriolo L, Berruto M, Denti M, Espregueira-Mendes J, et al. Sport and early osteoarthritis: the role of sport in aetiology, progression and treatment of knee osteoarthritis. Knee Surgery, Sport Traumatol Arthrosc. 2016;24(6):1786–96. 13. Fröhlich S, Pazeller S, Cherati AS, Müller E, Frey WO, Spörri J. Overuse injuries in the knee, back and hip of top elite female alpine skiers during  the off-season preparation period: prevalence, severity and their association with traumatic preinjuries and training load. BMJ open Sport Exerc Med. 2020;6(1):e000892. 14. Rincon GA, Vyas D, Zhou J, Fu FH, Oiestad BE, Holm I, et al. \“ Knee function and prevalence of knee osteoarthritis after anterior cruciate ligament reconstruction: a prospective study with 10 to 15 years of follow-up\”. Letter to the editor. Am J Sports Med. 2011;39(4):NP3-author. 15. Kellgren JH, Lawrence J. Radiological assessment of osteo-arthrosis. Ann Rheum Dis. 1957;16(4):494. 16. Altman R, Asch E, Bloch D, Bole G, Borenstein D, Brandt K, et al. Development of criteria for the classification and reporting of osteoarthritis: classification of osteoarthritis of the knee. Arthritis Rheum Off J Am Coll Rheumatol. 1986;29(8):1039–49. 17. Luyten FP, Denti M, Filardo G, Kon E, Engebretsen L. Definition and classification of early osteoarthritis of the knee. Knee Surgery, Sport Traumatol Arthrosc. 2012;20(3):401–6. 18. Hoemann C, Kandel R, Roberts S, Saris DBF, Creemers L, Mainil-Varlet P, et al. International Cartilage Repair Society (ICRS) Recommended Guidelines for  Histological Endpoints for Cartilage Repair Studies in Animal Models and Clinical Trials. Cartilage. 2011 Apr;2(2):153–72. 19. Hunter DJ, Lo GH, Gale D, Grainger AJ, Guermazi A, Conaghan PG. The reliability of a new scoring system for knee osteoarthritis MRI and the validity of bone marrow lesion assessment: BLOKS (Boston–Leeds Osteoarthritis Knee Score). Ann Rheum Dis. 2008;67(2):206–11. 20. Peterfy CG, Guermazi A, Zaim S, Tirman PFJ, Miaux Y, White D, et al. Whole-organ magnetic resonance imaging score (WORMS) of the knee in osteoarthritis. Osteoarthr Cartil. 2004;12(3):177–90. 21. Demange MK, Sisto M, Rodeo S. Future trends for unicompartmental arthritis of the knee: injectables & stem cells. Clin Sports Med. 2014;33(1):161–74. 22. Leslie M. Knee osteoarthritis management therapies. Pain Manag Nurs. 2000;1(2):51–7. 23. Paoloni M, Bernetti A, Belelli A, Brignoli O, Buoso S, Caputi AP, et al. Appropriateness of clinical and organizational criteria for intra-articular injection therapies in osteoarthritis: A Delphi method consensus initiative among experts in Italy. Ann Ist Super Sanita. 2015;51:131–8. 24. Sandmark H, Hogstedt C, Lewold S, Vingård E. Osteoarthrosis of the knee in men and women in association with overweight, smoking, and hormone therapy. Ann Rheum Dis. 1999;58(3):151–5. 25. Christensen BV, Iuhl IU, Vilbek H, Bülow H, Dreijer NC, Rasmussen HF. Acupuncture treatment of severe knee osteoarthrosis. A long‐term study. Acta Anaesthesiol Scand. 1992;36(6):519–25. 26. de Sire A, Agostini F, Lippi L, Mangone M, Marchese S, Cisari C, et al. Oxygen-Ozone Therapy in the Rehabilitation Field: State of the Art on Mechanisms of  Action, Safety and Effectiveness in Patients with Musculoskeletal Disorders. Biomolecules. 2021 Feb;11(3). 27. Fransen M, McCONNELL S. Land-based exercise for osteoarthritis of the knee: a metaanalysis of randomized controlled trials. J Rheumatol. 2009;36(6):1109–17. 28. Hiemstra LA, Kerslake S, Irving C. Anterior knee pain in the athlete. Clin Sports Med. 2014;33(3):437–59. 29. Filardo G, Kon E, Di Matteo B, Pelotti P, Di Martino A, Marcacci M. Platelet-rich plasma for the treatment of patellar tendinopathy: clinical and imaging findings at medium-term follow-up. Int Orthop [Internet]. 2013;37(8):1583–9. Available from: https://doi.org/10.1007/s00264-013-1972-830. Trojian TH, Concoff AL, Joy SM, Hatzenbuehler JR, Saulsberry WJ, Coleman CI. AMSSM Scientific Statement Concerning Viscosupplementation Injections for Knee  Osteoarthritis: Importance for Individual Patient Outcomes. Clin J Sport Med  Off J Can Acad  Sport Med. 2016 Jan;26(1):1–11. 31. Ammar TY, Pereira TAP, Mistura SLL, Kuhn A, Saggin JI, Lopes OV. Viscosupplementation for treating knee osteoarthrosis: review of the literature. Rev Bras Ortop. 2015;50:489–94. 32. Salini V, Pantalone A, Vanni D, Abate M. Viscosupplementation in Athletes. In: Arthroscopy and Sport Injuries. Springer; 2016. p. 433–7. 33. Erdem Y, Gul D, Akpancar S. Comparison of Intraarticular Injections of Hyaluronic Acid versus Dextrose Applied with Periarticular Prolotherapy in the Treatment of Recreational Athletes with Knee Osteoarthritis. Turkish J Sport Med. 2020;55(1):6–13. 34. Kalkhoven J, Coutts AJ, Impellizzeri FM. 'Training load error' is not a more accurate term than 'overuse' injury. Br J Sports Med [Internet]. 2020 Aug 1;54(15):934 LP – 935. Available from: http://bjsm.bmj.com/content/54/15/934.abstract35. Odole AC, Agbomeji OT, Onyeso OKK, Ojo JO, Odunaiya NA. Perspectives of Nigerian Athletes About Physiotherapy Services in Sports Injury Management: Implications for Rehabilitation. J Sport Rehabil. 2021;1(aop):1–8. 36. Hendrika W, Reswari A. The effect of physiotherapy on pain improvement in patients with early knee osteoarthritis at RSU UKI. Int J Med Heal Res. 2021;7(6):52–9. 37. MacKay C, Hawker GA, Jaglal SB. How Do Physical Therapists Approach Management of People With Early Knee Osteoarthritis? A Qualitative Study. Phys Ther [Internet]. 2019;100(2):295–306. Available from: https://doi.org/10.1093/ptj/pzz16438. Hrazdira L, Pilecki Z, Pilecki G. Ultrasound guided application of HA in the soft tissues of the locomotor system in athletes–First experience. Krátká sdělení Botek, M, Stejskal, P, Klimešová, I Vliv normobarické hypoxie na Akt Auton Nerv systému Pilot Stud 191 Drbošalová, V, Stejskal, P, Klugar, M, Krejčíř, V, Hynková, O Charakt klientů komerčn. :205. 39. Ricci M, Micheloni GM, Berti M, Perusi F, Sambugaro E, Vecchini E, et al. Clinical comparison of oral administration and viscosupplementation of hyaluronic acid (HA) in early knee osteoarthritis. Musculoskelet Surg. 2017;101(1):45–9. 40. Wang C-T, Lin Y-T, Chiang B-L, Lin Y-H, Hou S-M. High molecular weight hyaluronic acid down-regulates the gene expression of osteoarthritis-associated cytokines and enzymes in fibroblast-like synoviocytes from patients with early osteoarthritis. Osteoarthr Cartil. 2006;14(12):1237–47. 41. Irrgang JJ, Anderson AF. Development and validation of health-related quality of life measures for the knee. Clin Orthop Relat Res. 2002;402:95–109. 42. Roos EM, Roos HP, Lohmander LS, Ekdahl C, Beynnon BD. Knee Injury and Osteoarthritis Outcome Score (KOOS)—development of a self-administered outcome measure. J Orthop Sport Phys Ther. 1998;28(2):88–96. 43. Perruccio A V, Canizares M, Hawker GA, Roos EM, Maillefert JF, Lohmander SL. Comparative Validity and Responsiveness of the HOOS-PS and KOOS-PS to the WOMAC Physical Function Subscale in Total Joint Replacement for Osteoarthritis. 44. Salavati M, Mazaheri M, Negahban H, Sohani SM, Ebrahimian MR, Ebrahimi I, et al. Validation of a Persian-version of Knee injury and Osteoarthritis Outcome Score (KOOS) in Iranians with knee injuries. Osteoarthr Cartil. 2008;16(10):1178–82. 45. Higgins LD, Taylor MK, Park D, Ghodadra N, Marchant M, Pietrobon R, et al. Reliability and validity of the International Knee Documentation Committee (IKDC) subjective knee form. Jt Bone Spine. 2007;74(6):594–9. 46. Kujala UM, Kaprio J, Sarno S. Osteoarthritis of weight bearing joints of lower limbs in former elite male athletes. Bmj. 1994;308(6923):231–4. 47. Salini V, Pantalone A, Vanni D. Arthroscopy and Sport Injuries. Arthrosc Sport Inj. 2016;433–7. 48. Broglio K. Randomization in clinical trials: Permuted blocks and stratification. JAMA - J Am Med Assoc. 2018;319(21):2223–4. 49. Emanuel EJ, Wendler D, Grady C. What makes clinical research ethical? JAMA. 2000 May;283(20):2701–11. )

[8.	Yang J, Tibbetts AS, Covassin T, Cheng G, Nayar S, Heiden E. Epidemiology of overuse and acute injuries among competitive collegiate athletes. J Athl Train. 2012;47(2):198–204.](1. Patel DR, Villalobos A. Evaluation and management of knee pain in young athletes: overuse injuries of the knee. Transl Pediatr [Internet]. 2017 Jul;6(3):190–8. Available from: https://pubmed.ncbi.nlm.nih.gov/287950102. Nicolini AP, de Carvalho RT, Matsuda MM, Filho JS, Cohen M. Common injuries in athletes' knee: Experience of a specialized center. Acta Ortop Bras. 2014;22(3):127–31. 3. Tiu T, Craig Van Dien MD. Knee overuse disorders. 4. Fuller CW, Ekstrand J, Junge A, Andersen TE, Bahr R, Dvorak J, et al. Consensus statement on injury definitions and data collection procedures in studies  of football (soccer) injuries. Br J Sports Med. 2006 Mar;40(3):193–201. 5. Majewski M, Susanne H, Klaus S. Epidemiology of athletic knee injuries: A 10-year study. Knee. 2006 Jun;13(3):184–8. 6. Wilder RP, Sethi S. Overuse injuries: tendinopathies, stress fractures, compartment syndrome, and shin  splints. Clin Sports Med. 2004 Jan;23(1):55–81, vi. 7. Aicale R, Tarantino D, Maffulli N. Overuse injuries in sport: a comprehensive overview. J Orthop Surg Res. 2018 Dec;13(1):309. 8. Yang J, Tibbetts AS, Covassin T, Cheng G, Nayar S, Heiden E. Epidemiology of overuse and acute injuries among competitive collegiate athletes. J Athl Train. 2012;47(2):198–204. 9. Bernetti A, Mangone M, Paoloni M, Di Sante L, Murgia M, Santilli V. Corticosteroid and hyaluronic acid injection therapy in tennis elbow (lateral epicondylalgia). Med Sport. 2014;67(2):289–95. 10. Iolascon G, Gimigliano F, Moretti A, De Sire A, Migliore A, Brandi ML, et al. Early osteoarthritis: How to define, diagnose, and manage. A systematic review. Eur Geriatr Med. 2017;8(5–6):383–96. 11. Drawer S, Fuller CW. Propensity for osteoarthritis and lower limb joint pain in retired professional soccer players. Br J Sports Med. 2001;35(6):402–8. 12. Vannini F, Spalding T, Andriolo L, Berruto M, Denti M, Espregueira-Mendes J, et al. Sport and early osteoarthritis: the role of sport in aetiology, progression and treatment of knee osteoarthritis. Knee Surgery, Sport Traumatol Arthrosc. 2016;24(6):1786–96. 13. Fröhlich S, Pazeller S, Cherati AS, Müller E, Frey WO, Spörri J. Overuse injuries in the knee, back and hip of top elite female alpine skiers during  the off-season preparation period: prevalence, severity and their association with traumatic preinjuries and training load. BMJ open Sport Exerc Med. 2020;6(1):e000892. 14. Rincon GA, Vyas D, Zhou J, Fu FH, Oiestad BE, Holm I, et al. \“ Knee function and prevalence of knee osteoarthritis after anterior cruciate ligament reconstruction: a prospective study with 10 to 15 years of follow-up\”. Letter to the editor. Am J Sports Med. 2011;39(4):NP3-author. 15. Kellgren JH, Lawrence J. Radiological assessment of osteo-arthrosis. Ann Rheum Dis. 1957;16(4):494. 16. Altman R, Asch E, Bloch D, Bole G, Borenstein D, Brandt K, et al. Development of criteria for the classification and reporting of osteoarthritis: classification of osteoarthritis of the knee. Arthritis Rheum Off J Am Coll Rheumatol. 1986;29(8):1039–49. 17. Luyten FP, Denti M, Filardo G, Kon E, Engebretsen L. Definition and classification of early osteoarthritis of the knee. Knee Surgery, Sport Traumatol Arthrosc. 2012;20(3):401–6. 18. Hoemann C, Kandel R, Roberts S, Saris DBF, Creemers L, Mainil-Varlet P, et al. International Cartilage Repair Society (ICRS) Recommended Guidelines for  Histological Endpoints for Cartilage Repair Studies in Animal Models and Clinical Trials. Cartilage. 2011 Apr;2(2):153–72. 19. Hunter DJ, Lo GH, Gale D, Grainger AJ, Guermazi A, Conaghan PG. The reliability of a new scoring system for knee osteoarthritis MRI and the validity of bone marrow lesion assessment: BLOKS (Boston–Leeds Osteoarthritis Knee Score). Ann Rheum Dis. 2008;67(2):206–11. 20. Peterfy CG, Guermazi A, Zaim S, Tirman PFJ, Miaux Y, White D, et al. Whole-organ magnetic resonance imaging score (WORMS) of the knee in osteoarthritis. Osteoarthr Cartil. 2004;12(3):177–90. 21. Demange MK, Sisto M, Rodeo S. Future trends for unicompartmental arthritis of the knee: injectables & stem cells. Clin Sports Med. 2014;33(1):161–74. 22. Leslie M. Knee osteoarthritis management therapies. Pain Manag Nurs. 2000;1(2):51–7. 23. Paoloni M, Bernetti A, Belelli A, Brignoli O, Buoso S, Caputi AP, et al. Appropriateness of clinical and organizational criteria for intra-articular injection therapies in osteoarthritis: A Delphi method consensus initiative among experts in Italy. Ann Ist Super Sanita. 2015;51:131–8. 24. Sandmark H, Hogstedt C, Lewold S, Vingård E. Osteoarthrosis of the knee in men and women in association with overweight, smoking, and hormone therapy. Ann Rheum Dis. 1999;58(3):151–5. 25. Christensen BV, Iuhl IU, Vilbek H, Bülow H, Dreijer NC, Rasmussen HF. Acupuncture treatment of severe knee osteoarthrosis. A long‐term study. Acta Anaesthesiol Scand. 1992;36(6):519–25. 26. de Sire A, Agostini F, Lippi L, Mangone M, Marchese S, Cisari C, et al. Oxygen-Ozone Therapy in the Rehabilitation Field: State of the Art on Mechanisms of  Action, Safety and Effectiveness in Patients with Musculoskeletal Disorders. Biomolecules. 2021 Feb;11(3). 27. Fransen M, McCONNELL S. Land-based exercise for osteoarthritis of the knee: a metaanalysis of randomized controlled trials. J Rheumatol. 2009;36(6):1109–17. 28. Hiemstra LA, Kerslake S, Irving C. Anterior knee pain in the athlete. Clin Sports Med. 2014;33(3):437–59. 29. Filardo G, Kon E, Di Matteo B, Pelotti P, Di Martino A, Marcacci M. Platelet-rich plasma for the treatment of patellar tendinopathy: clinical and imaging findings at medium-term follow-up. Int Orthop [Internet]. 2013;37(8):1583–9. Available from: https://doi.org/10.1007/s00264-013-1972-830. Trojian TH, Concoff AL, Joy SM, Hatzenbuehler JR, Saulsberry WJ, Coleman CI. AMSSM Scientific Statement Concerning Viscosupplementation Injections for Knee  Osteoarthritis: Importance for Individual Patient Outcomes. Clin J Sport Med  Off J Can Acad  Sport Med. 2016 Jan;26(1):1–11. 31. Ammar TY, Pereira TAP, Mistura SLL, Kuhn A, Saggin JI, Lopes OV. Viscosupplementation for treating knee osteoarthrosis: review of the literature. Rev Bras Ortop. 2015;50:489–94. 32. Salini V, Pantalone A, Vanni D, Abate M. Viscosupplementation in Athletes. In: Arthroscopy and Sport Injuries. Springer; 2016. p. 433–7. 33. Erdem Y, Gul D, Akpancar S. Comparison of Intraarticular Injections of Hyaluronic Acid versus Dextrose Applied with Periarticular Prolotherapy in the Treatment of Recreational Athletes with Knee Osteoarthritis. Turkish J Sport Med. 2020;55(1):6–13. 34. Kalkhoven J, Coutts AJ, Impellizzeri FM. 'Training load error' is not a more accurate term than 'overuse' injury. Br J Sports Med [Internet]. 2020 Aug 1;54(15):934 LP – 935. Available from: http://bjsm.bmj.com/content/54/15/934.abstract35. Odole AC, Agbomeji OT, Onyeso OKK, Ojo JO, Odunaiya NA. Perspectives of Nigerian Athletes About Physiotherapy Services in Sports Injury Management: Implications for Rehabilitation. J Sport Rehabil. 2021;1(aop):1–8. 36. Hendrika W, Reswari A. The effect of physiotherapy on pain improvement in patients with early knee osteoarthritis at RSU UKI. Int J Med Heal Res. 2021;7(6):52–9. 37. MacKay C, Hawker GA, Jaglal SB. How Do Physical Therapists Approach Management of People With Early Knee Osteoarthritis? A Qualitative Study. Phys Ther [Internet]. 2019;100(2):295–306. Available from: https://doi.org/10.1093/ptj/pzz16438. Hrazdira L, Pilecki Z, Pilecki G. Ultrasound guided application of HA in the soft tissues of the locomotor system in athletes–First experience. Krátká sdělení Botek, M, Stejskal, P, Klimešová, I Vliv normobarické hypoxie na Akt Auton Nerv systému Pilot Stud 191 Drbošalová, V, Stejskal, P, Klugar, M, Krejčíř, V, Hynková, O Charakt klientů komerčn. :205. 39. Ricci M, Micheloni GM, Berti M, Perusi F, Sambugaro E, Vecchini E, et al. Clinical comparison of oral administration and viscosupplementation of hyaluronic acid (HA) in early knee osteoarthritis. Musculoskelet Surg. 2017;101(1):45–9. 40. Wang C-T, Lin Y-T, Chiang B-L, Lin Y-H, Hou S-M. High molecular weight hyaluronic acid down-regulates the gene expression of osteoarthritis-associated cytokines and enzymes in fibroblast-like synoviocytes from patients with early osteoarthritis. Osteoarthr Cartil. 2006;14(12):1237–47. 41. Irrgang JJ, Anderson AF. Development and validation of health-related quality of life measures for the knee. Clin Orthop Relat Res. 2002;402:95–109. 42. Roos EM, Roos HP, Lohmander LS, Ekdahl C, Beynnon BD. Knee Injury and Osteoarthritis Outcome Score (KOOS)—development of a self-administered outcome measure. J Orthop Sport Phys Ther. 1998;28(2):88–96. 43. Perruccio A V, Canizares M, Hawker GA, Roos EM, Maillefert JF, Lohmander SL. Comparative Validity and Responsiveness of the HOOS-PS and KOOS-PS to the WOMAC Physical Function Subscale in Total Joint Replacement for Osteoarthritis. 44. Salavati M, Mazaheri M, Negahban H, Sohani SM, Ebrahimian MR, Ebrahimi I, et al. Validation of a Persian-version of Knee injury and Osteoarthritis Outcome Score (KOOS) in Iranians with knee injuries. Osteoarthr Cartil. 2008;16(10):1178–82. 45. Higgins LD, Taylor MK, Park D, Ghodadra N, Marchant M, Pietrobon R, et al. Reliability and validity of the International Knee Documentation Committee (IKDC) subjective knee form. Jt Bone Spine. 2007;74(6):594–9. 46. Kujala UM, Kaprio J, Sarno S. Osteoarthritis of weight bearing joints of lower limbs in former elite male athletes. Bmj. 1994;308(6923):231–4. 47. Salini V, Pantalone A, Vanni D. Arthroscopy and Sport Injuries. Arthrosc Sport Inj. 2016;433–7. 48. Broglio K. Randomization in clinical trials: Permuted blocks and stratification. JAMA - J Am Med Assoc. 2018;319(21):2223–4. 49. Emanuel EJ, Wendler D, Grady C. What makes clinical research ethical? JAMA. 2000 May;283(20):2701–11. )

[9.	Bernetti A, Mangone M, Paoloni M, Di Sante L, Murgia M, Santilli V. Corticosteroid and hyaluronic acid injection therapy in tennis elbow (lateral epicondylalgia). Med Sport. 2014;67(2):289–95.](1. Patel DR, Villalobos A. Evaluation and management of knee pain in young athletes: overuse injuries of the knee. Transl Pediatr [Internet]. 2017 Jul;6(3):190–8. Available from: https://pubmed.ncbi.nlm.nih.gov/287950102. Nicolini AP, de Carvalho RT, Matsuda MM, Filho JS, Cohen M. Common injuries in athletes' knee: Experience of a specialized center. Acta Ortop Bras. 2014;22(3):127–31. 3. Tiu T, Craig Van Dien MD. Knee overuse disorders. 4. Fuller CW, Ekstrand J, Junge A, Andersen TE, Bahr R, Dvorak J, et al. Consensus statement on injury definitions and data collection procedures in studies  of football (soccer) injuries. Br J Sports Med. 2006 Mar;40(3):193–201. 5. Majewski M, Susanne H, Klaus S. Epidemiology of athletic knee injuries: A 10-year study. Knee. 2006 Jun;13(3):184–8. 6. Wilder RP, Sethi S. Overuse injuries: tendinopathies, stress fractures, compartment syndrome, and shin  splints. Clin Sports Med. 2004 Jan;23(1):55–81, vi. 7. Aicale R, Tarantino D, Maffulli N. Overuse injuries in sport: a comprehensive overview. J Orthop Surg Res. 2018 Dec;13(1):309. 8. Yang J, Tibbetts AS, Covassin T, Cheng G, Nayar S, Heiden E. Epidemiology of overuse and acute injuries among competitive collegiate athletes. J Athl Train. 2012;47(2):198–204. 9. Bernetti A, Mangone M, Paoloni M, Di Sante L, Murgia M, Santilli V. Corticosteroid and hyaluronic acid injection therapy in tennis elbow (lateral epicondylalgia). Med Sport. 2014;67(2):289–95. 10. Iolascon G, Gimigliano F, Moretti A, De Sire A, Migliore A, Brandi ML, et al. Early osteoarthritis: How to define, diagnose, and manage. A systematic review. Eur Geriatr Med. 2017;8(5–6):383–96. 11. Drawer S, Fuller CW. Propensity for osteoarthritis and lower limb joint pain in retired professional soccer players. Br J Sports Med. 2001;35(6):402–8. 12. Vannini F, Spalding T, Andriolo L, Berruto M, Denti M, Espregueira-Mendes J, et al. Sport and early osteoarthritis: the role of sport in aetiology, progression and treatment of knee osteoarthritis. Knee Surgery, Sport Traumatol Arthrosc. 2016;24(6):1786–96. 13. Fröhlich S, Pazeller S, Cherati AS, Müller E, Frey WO, Spörri J. Overuse injuries in the knee, back and hip of top elite female alpine skiers during  the off-season preparation period: prevalence, severity and their association with traumatic preinjuries and training load. BMJ open Sport Exerc Med. 2020;6(1):e000892. 14. Rincon GA, Vyas D, Zhou J, Fu FH, Oiestad BE, Holm I, et al. \“ Knee function and prevalence of knee osteoarthritis after anterior cruciate ligament reconstruction: a prospective study with 10 to 15 years of follow-up\”. Letter to the editor. Am J Sports Med. 2011;39(4):NP3-author. 15. Kellgren JH, Lawrence J. Radiological assessment of osteo-arthrosis. Ann Rheum Dis. 1957;16(4):494. 16. Altman R, Asch E, Bloch D, Bole G, Borenstein D, Brandt K, et al. Development of criteria for the classification and reporting of osteoarthritis: classification of osteoarthritis of the knee. Arthritis Rheum Off J Am Coll Rheumatol. 1986;29(8):1039–49. 17. Luyten FP, Denti M, Filardo G, Kon E, Engebretsen L. Definition and classification of early osteoarthritis of the knee. Knee Surgery, Sport Traumatol Arthrosc. 2012;20(3):401–6. 18. Hoemann C, Kandel R, Roberts S, Saris DBF, Creemers L, Mainil-Varlet P, et al. International Cartilage Repair Society (ICRS) Recommended Guidelines for  Histological Endpoints for Cartilage Repair Studies in Animal Models and Clinical Trials. Cartilage. 2011 Apr;2(2):153–72. 19. Hunter DJ, Lo GH, Gale D, Grainger AJ, Guermazi A, Conaghan PG. The reliability of a new scoring system for knee osteoarthritis MRI and the validity of bone marrow lesion assessment: BLOKS (Boston–Leeds Osteoarthritis Knee Score). Ann Rheum Dis. 2008;67(2):206–11. 20. Peterfy CG, Guermazi A, Zaim S, Tirman PFJ, Miaux Y, White D, et al. Whole-organ magnetic resonance imaging score (WORMS) of the knee in osteoarthritis. Osteoarthr Cartil. 2004;12(3):177–90. 21. Demange MK, Sisto M, Rodeo S. Future trends for unicompartmental arthritis of the knee: injectables & stem cells. Clin Sports Med. 2014;33(1):161–74. 22. Leslie M. Knee osteoarthritis management therapies. Pain Manag Nurs. 2000;1(2):51–7. 23. Paoloni M, Bernetti A, Belelli A, Brignoli O, Buoso S, Caputi AP, et al. Appropriateness of clinical and organizational criteria for intra-articular injection therapies in osteoarthritis: A Delphi method consensus initiative among experts in Italy. Ann Ist Super Sanita. 2015;51:131–8. 24. Sandmark H, Hogstedt C, Lewold S, Vingård E. Osteoarthrosis of the knee in men and women in association with overweight, smoking, and hormone therapy. Ann Rheum Dis. 1999;58(3):151–5. 25. Christensen BV, Iuhl IU, Vilbek H, Bülow H, Dreijer NC, Rasmussen HF. Acupuncture treatment of severe knee osteoarthrosis. A long‐term study. Acta Anaesthesiol Scand. 1992;36(6):519–25. 26. de Sire A, Agostini F, Lippi L, Mangone M, Marchese S, Cisari C, et al. Oxygen-Ozone Therapy in the Rehabilitation Field: State of the Art on Mechanisms of  Action, Safety and Effectiveness in Patients with Musculoskeletal Disorders. Biomolecules. 2021 Feb;11(3). 27. Fransen M, McCONNELL S. Land-based exercise for osteoarthritis of the knee: a metaanalysis of randomized controlled trials. J Rheumatol. 2009;36(6):1109–17. 28. Hiemstra LA, Kerslake S, Irving C. Anterior knee pain in the athlete. Clin Sports Med. 2014;33(3):437–59. 29. Filardo G, Kon E, Di Matteo B, Pelotti P, Di Martino A, Marcacci M. Platelet-rich plasma for the treatment of patellar tendinopathy: clinical and imaging findings at medium-term follow-up. Int Orthop [Internet]. 2013;37(8):1583–9. Available from: https://doi.org/10.1007/s00264-013-1972-830. Trojian TH, Concoff AL, Joy SM, Hatzenbuehler JR, Saulsberry WJ, Coleman CI. AMSSM Scientific Statement Concerning Viscosupplementation Injections for Knee  Osteoarthritis: Importance for Individual Patient Outcomes. Clin J Sport Med  Off J Can Acad  Sport Med. 2016 Jan;26(1):1–11. 31. Ammar TY, Pereira TAP, Mistura SLL, Kuhn A, Saggin JI, Lopes OV. Viscosupplementation for treating knee osteoarthrosis: review of the literature. Rev Bras Ortop. 2015;50:489–94. 32. Salini V, Pantalone A, Vanni D, Abate M. Viscosupplementation in Athletes. In: Arthroscopy and Sport Injuries. Springer; 2016. p. 433–7. 33. Erdem Y, Gul D, Akpancar S. Comparison of Intraarticular Injections of Hyaluronic Acid versus Dextrose Applied with Periarticular Prolotherapy in the Treatment of Recreational Athletes with Knee Osteoarthritis. Turkish J Sport Med. 2020;55(1):6–13. 34. Kalkhoven J, Coutts AJ, Impellizzeri FM. 'Training load error' is not a more accurate term than 'overuse' injury. Br J Sports Med [Internet]. 2020 Aug 1;54(15):934 LP – 935. Available from: http://bjsm.bmj.com/content/54/15/934.abstract35. Odole AC, Agbomeji OT, Onyeso OKK, Ojo JO, Odunaiya NA. Perspectives of Nigerian Athletes About Physiotherapy Services in Sports Injury Management: Implications for Rehabilitation. J Sport Rehabil. 2021;1(aop):1–8. 36. Hendrika W, Reswari A. The effect of physiotherapy on pain improvement in patients with early knee osteoarthritis at RSU UKI. Int J Med Heal Res. 2021;7(6):52–9. 37. MacKay C, Hawker GA, Jaglal SB. How Do Physical Therapists Approach Management of People With Early Knee Osteoarthritis? A Qualitative Study. Phys Ther [Internet]. 2019;100(2):295–306. Available from: https://doi.org/10.1093/ptj/pzz16438. Hrazdira L, Pilecki Z, Pilecki G. Ultrasound guided application of HA in the soft tissues of the locomotor system in athletes–First experience. Krátká sdělení Botek, M, Stejskal, P, Klimešová, I Vliv normobarické hypoxie na Akt Auton Nerv systému Pilot Stud 191 Drbošalová, V, Stejskal, P, Klugar, M, Krejčíř, V, Hynková, O Charakt klientů komerčn. :205. 39. Ricci M, Micheloni GM, Berti M, Perusi F, Sambugaro E, Vecchini E, et al. Clinical comparison of oral administration and viscosupplementation of hyaluronic acid (HA) in early knee osteoarthritis. Musculoskelet Surg. 2017;101(1):45–9. 40. Wang C-T, Lin Y-T, Chiang B-L, Lin Y-H, Hou S-M. High molecular weight hyaluronic acid down-regulates the gene expression of osteoarthritis-associated cytokines and enzymes in fibroblast-like synoviocytes from patients with early osteoarthritis. Osteoarthr Cartil. 2006;14(12):1237–47. 41. Irrgang JJ, Anderson AF. Development and validation of health-related quality of life measures for the knee. Clin Orthop Relat Res. 2002;402:95–109. 42. Roos EM, Roos HP, Lohmander LS, Ekdahl C, Beynnon BD. Knee Injury and Osteoarthritis Outcome Score (KOOS)—development of a self-administered outcome measure. J Orthop Sport Phys Ther. 1998;28(2):88–96. 43. Perruccio A V, Canizares M, Hawker GA, Roos EM, Maillefert JF, Lohmander SL. Comparative Validity and Responsiveness of the HOOS-PS and KOOS-PS to the WOMAC Physical Function Subscale in Total Joint Replacement for Osteoarthritis. 44. Salavati M, Mazaheri M, Negahban H, Sohani SM, Ebrahimian MR, Ebrahimi I, et al. Validation of a Persian-version of Knee injury and Osteoarthritis Outcome Score (KOOS) in Iranians with knee injuries. Osteoarthr Cartil. 2008;16(10):1178–82. 45. Higgins LD, Taylor MK, Park D, Ghodadra N, Marchant M, Pietrobon R, et al. Reliability and validity of the International Knee Documentation Committee (IKDC) subjective knee form. Jt Bone Spine. 2007;74(6):594–9. 46. Kujala UM, Kaprio J, Sarno S. Osteoarthritis of weight bearing joints of lower limbs in former elite male athletes. Bmj. 1994;308(6923):231–4. 47. Salini V, Pantalone A, Vanni D. Arthroscopy and Sport Injuries. Arthrosc Sport Inj. 2016;433–7. 48. Broglio K. Randomization in clinical trials: Permuted blocks and stratification. JAMA - J Am Med Assoc. 2018;319(21):2223–4. 49. Emanuel EJ, Wendler D, Grady C. What makes clinical research ethical? JAMA. 2000 May;283(20):2701–11. )

[10.	Iolascon G, Gimigliano F, Moretti A, De Sire A, Migliore A, Brandi ML, et al. Early osteoarthritis: How to define, diagnose, and manage. A systematic review. Eur Geriatr Med. 2017;8(5–6):383–96.](1. Patel DR, Villalobos A. Evaluation and management of knee pain in young athletes: overuse injuries of the knee. Transl Pediatr [Internet]. 2017 Jul;6(3):190–8. Available from: https://pubmed.ncbi.nlm.nih.gov/287950102. Nicolini AP, de Carvalho RT, Matsuda MM, Filho JS, Cohen M. Common injuries in athletes' knee: Experience of a specialized center. Acta Ortop Bras. 2014;22(3):127–31. 3. Tiu T, Craig Van Dien MD. Knee overuse disorders. 4. Fuller CW, Ekstrand J, Junge A, Andersen TE, Bahr R, Dvorak J, et al. Consensus statement on injury definitions and data collection procedures in studies  of football (soccer) injuries. Br J Sports Med. 2006 Mar;40(3):193–201. 5. Majewski M, Susanne H, Klaus S. Epidemiology of athletic knee injuries: A 10-year study. Knee. 2006 Jun;13(3):184–8. 6. Wilder RP, Sethi S. Overuse injuries: tendinopathies, stress fractures, compartment syndrome, and shin  splints. Clin Sports Med. 2004 Jan;23(1):55–81, vi. 7. Aicale R, Tarantino D, Maffulli N. Overuse injuries in sport: a comprehensive overview. J Orthop Surg Res. 2018 Dec;13(1):309. 8. Yang J, Tibbetts AS, Covassin T, Cheng G, Nayar S, Heiden E. Epidemiology of overuse and acute injuries among competitive collegiate athletes. J Athl Train. 2012;47(2):198–204. 9. Bernetti A, Mangone M, Paoloni M, Di Sante L, Murgia M, Santilli V. Corticosteroid and hyaluronic acid injection therapy in tennis elbow (lateral epicondylalgia). Med Sport. 2014;67(2):289–95. 10. Iolascon G, Gimigliano F, Moretti A, De Sire A, Migliore A, Brandi ML, et al. Early osteoarthritis: How to define, diagnose, and manage. A systematic review. Eur Geriatr Med. 2017;8(5–6):383–96. 11. Drawer S, Fuller CW. Propensity for osteoarthritis and lower limb joint pain in retired professional soccer players. Br J Sports Med. 2001;35(6):402–8. 12. Vannini F, Spalding T, Andriolo L, Berruto M, Denti M, Espregueira-Mendes J, et al. Sport and early osteoarthritis: the role of sport in aetiology, progression and treatment of knee osteoarthritis. Knee Surgery, Sport Traumatol Arthrosc. 2016;24(6):1786–96. 13. Fröhlich S, Pazeller S, Cherati AS, Müller E, Frey WO, Spörri J. Overuse injuries in the knee, back and hip of top elite female alpine skiers during  the off-season preparation period: prevalence, severity and their association with traumatic preinjuries and training load. BMJ open Sport Exerc Med. 2020;6(1):e000892. 14. Rincon GA, Vyas D, Zhou J, Fu FH, Oiestad BE, Holm I, et al. \“ Knee function and prevalence of knee osteoarthritis after anterior cruciate ligament reconstruction: a prospective study with 10 to 15 years of follow-up\”. Letter to the editor. Am J Sports Med. 2011;39(4):NP3-author. 15. Kellgren JH, Lawrence J. Radiological assessment of osteo-arthrosis. Ann Rheum Dis. 1957;16(4):494. 16. Altman R, Asch E, Bloch D, Bole G, Borenstein D, Brandt K, et al. Development of criteria for the classification and reporting of osteoarthritis: classification of osteoarthritis of the knee. Arthritis Rheum Off J Am Coll Rheumatol. 1986;29(8):1039–49. 17. Luyten FP, Denti M, Filardo G, Kon E, Engebretsen L. Definition and classification of early osteoarthritis of the knee. Knee Surgery, Sport Traumatol Arthrosc. 2012;20(3):401–6. 18. Hoemann C, Kandel R, Roberts S, Saris DBF, Creemers L, Mainil-Varlet P, et al. International Cartilage Repair Society (ICRS) Recommended Guidelines for  Histological Endpoints for Cartilage Repair Studies in Animal Models and Clinical Trials. Cartilage. 2011 Apr;2(2):153–72. 19. Hunter DJ, Lo GH, Gale D, Grainger AJ, Guermazi A, Conaghan PG. The reliability of a new scoring system for knee osteoarthritis MRI and the validity of bone marrow lesion assessment: BLOKS (Boston–Leeds Osteoarthritis Knee Score). Ann Rheum Dis. 2008;67(2):206–11. 20. Peterfy CG, Guermazi A, Zaim S, Tirman PFJ, Miaux Y, White D, et al. Whole-organ magnetic resonance imaging score (WORMS) of the knee in osteoarthritis. Osteoarthr Cartil. 2004;12(3):177–90. 21. Demange MK, Sisto M, Rodeo S. Future trends for unicompartmental arthritis of the knee: injectables & stem cells. Clin Sports Med. 2014;33(1):161–74. 22. Leslie M. Knee osteoarthritis management therapies. Pain Manag Nurs. 2000;1(2):51–7. 23. Paoloni M, Bernetti A, Belelli A, Brignoli O, Buoso S, Caputi AP, et al. Appropriateness of clinical and organizational criteria for intra-articular injection therapies in osteoarthritis: A Delphi method consensus initiative among experts in Italy. Ann Ist Super Sanita. 2015;51:131–8. 24. Sandmark H, Hogstedt C, Lewold S, Vingård E. Osteoarthrosis of the knee in men and women in association with overweight, smoking, and hormone therapy. Ann Rheum Dis. 1999;58(3):151–5. 25. Christensen BV, Iuhl IU, Vilbek H, Bülow H, Dreijer NC, Rasmussen HF. Acupuncture treatment of severe knee osteoarthrosis. A long‐term study. Acta Anaesthesiol Scand. 1992;36(6):519–25. 26. de Sire A, Agostini F, Lippi L, Mangone M, Marchese S, Cisari C, et al. Oxygen-Ozone Therapy in the Rehabilitation Field: State of the Art on Mechanisms of  Action, Safety and Effectiveness in Patients with Musculoskeletal Disorders. Biomolecules. 2021 Feb;11(3). 27. Fransen M, McCONNELL S. Land-based exercise for osteoarthritis of the knee: a metaanalysis of randomized controlled trials. J Rheumatol. 2009;36(6):1109–17. 28. Hiemstra LA, Kerslake S, Irving C. Anterior knee pain in the athlete. Clin Sports Med. 2014;33(3):437–59. 29. Filardo G, Kon E, Di Matteo B, Pelotti P, Di Martino A, Marcacci M. Platelet-rich plasma for the treatment of patellar tendinopathy: clinical and imaging findings at medium-term follow-up. Int Orthop [Internet]. 2013;37(8):1583–9. Available from: https://doi.org/10.1007/s00264-013-1972-830. Trojian TH, Concoff AL, Joy SM, Hatzenbuehler JR, Saulsberry WJ, Coleman CI. AMSSM Scientific Statement Concerning Viscosupplementation Injections for Knee  Osteoarthritis: Importance for Individual Patient Outcomes. Clin J Sport Med  Off J Can Acad  Sport Med. 2016 Jan;26(1):1–11. 31. Ammar TY, Pereira TAP, Mistura SLL, Kuhn A, Saggin JI, Lopes OV. Viscosupplementation for treating knee osteoarthrosis: review of the literature. Rev Bras Ortop. 2015;50:489–94. 32. Salini V, Pantalone A, Vanni D, Abate M. Viscosupplementation in Athletes. In: Arthroscopy and Sport Injuries. Springer; 2016. p. 433–7. 33. Erdem Y, Gul D, Akpancar S. Comparison of Intraarticular Injections of Hyaluronic Acid versus Dextrose Applied with Periarticular Prolotherapy in the Treatment of Recreational Athletes with Knee Osteoarthritis. Turkish J Sport Med. 2020;55(1):6–13. 34. Kalkhoven J, Coutts AJ, Impellizzeri FM. 'Training load error' is not a more accurate term than 'overuse' injury. Br J Sports Med [Internet]. 2020 Aug 1;54(15):934 LP – 935. Available from: http://bjsm.bmj.com/content/54/15/934.abstract35. Odole AC, Agbomeji OT, Onyeso OKK, Ojo JO, Odunaiya NA. Perspectives of Nigerian Athletes About Physiotherapy Services in Sports Injury Management: Implications for Rehabilitation. J Sport Rehabil. 2021;1(aop):1–8. 36. Hendrika W, Reswari A. The effect of physiotherapy on pain improvement in patients with early knee osteoarthritis at RSU UKI. Int J Med Heal Res. 2021;7(6):52–9. 37. MacKay C, Hawker GA, Jaglal SB. How Do Physical Therapists Approach Management of People With Early Knee Osteoarthritis? A Qualitative Study. Phys Ther [Internet]. 2019;100(2):295–306. Available from: https://doi.org/10.1093/ptj/pzz16438. Hrazdira L, Pilecki Z, Pilecki G. Ultrasound guided application of HA in the soft tissues of the locomotor system in athletes–First experience. Krátká sdělení Botek, M, Stejskal, P, Klimešová, I Vliv normobarické hypoxie na Akt Auton Nerv systému Pilot Stud 191 Drbošalová, V, Stejskal, P, Klugar, M, Krejčíř, V, Hynková, O Charakt klientů komerčn. :205. 39. Ricci M, Micheloni GM, Berti M, Perusi F, Sambugaro E, Vecchini E, et al. Clinical comparison of oral administration and viscosupplementation of hyaluronic acid (HA) in early knee osteoarthritis. Musculoskelet Surg. 2017;101(1):45–9. 40. Wang C-T, Lin Y-T, Chiang B-L, Lin Y-H, Hou S-M. High molecular weight hyaluronic acid down-regulates the gene expression of osteoarthritis-associated cytokines and enzymes in fibroblast-like synoviocytes from patients with early osteoarthritis. Osteoarthr Cartil. 2006;14(12):1237–47. 41. Irrgang JJ, Anderson AF. Development and validation of health-related quality of life measures for the knee. Clin Orthop Relat Res. 2002;402:95–109. 42. Roos EM, Roos HP, Lohmander LS, Ekdahl C, Beynnon BD. Knee Injury and Osteoarthritis Outcome Score (KOOS)—development of a self-administered outcome measure. J Orthop Sport Phys Ther. 1998;28(2):88–96. 43. Perruccio A V, Canizares M, Hawker GA, Roos EM, Maillefert JF, Lohmander SL. Comparative Validity and Responsiveness of the HOOS-PS and KOOS-PS to the WOMAC Physical Function Subscale in Total Joint Replacement for Osteoarthritis. 44. Salavati M, Mazaheri M, Negahban H, Sohani SM, Ebrahimian MR, Ebrahimi I, et al. Validation of a Persian-version of Knee injury and Osteoarthritis Outcome Score (KOOS) in Iranians with knee injuries. Osteoarthr Cartil. 2008;16(10):1178–82. 45. Higgins LD, Taylor MK, Park D, Ghodadra N, Marchant M, Pietrobon R, et al. Reliability and validity of the International Knee Documentation Committee (IKDC) subjective knee form. Jt Bone Spine. 2007;74(6):594–9. 46. Kujala UM, Kaprio J, Sarno S. Osteoarthritis of weight bearing joints of lower limbs in former elite male athletes. Bmj. 1994;308(6923):231–4. 47. Salini V, Pantalone A, Vanni D. Arthroscopy and Sport Injuries. Arthrosc Sport Inj. 2016;433–7. 48. Broglio K. Randomization in clinical trials: Permuted blocks and stratification. JAMA - J Am Med Assoc. 2018;319(21):2223–4. 49. Emanuel EJ, Wendler D, Grady C. What makes clinical research ethical? JAMA. 2000 May;283(20):2701–11. )

[11.	Drawer S, Fuller CW. Propensity for osteoarthritis and lower limb joint pain in retired professional soccer players. Br J Sports Med. 2001;35(6):402–8.](1. Patel DR, Villalobos A. Evaluation and management of knee pain in young athletes: overuse injuries of the knee. Transl Pediatr [Internet]. 2017 Jul;6(3):190–8. Available from: https://pubmed.ncbi.nlm.nih.gov/287950102. Nicolini AP, de Carvalho RT, Matsuda MM, Filho JS, Cohen M. Common injuries in athletes' knee: Experience of a specialized center. Acta Ortop Bras. 2014;22(3):127–31. 3. Tiu T, Craig Van Dien MD. Knee overuse disorders. 4. Fuller CW, Ekstrand J, Junge A, Andersen TE, Bahr R, Dvorak J, et al. Consensus statement on injury definitions and data collection procedures in studies  of football (soccer) injuries. Br J Sports Med. 2006 Mar;40(3):193–201. 5. Majewski M, Susanne H, Klaus S. Epidemiology of athletic knee injuries: A 10-year study. Knee. 2006 Jun;13(3):184–8. 6. Wilder RP, Sethi S. Overuse injuries: tendinopathies, stress fractures, compartment syndrome, and shin  splints. Clin Sports Med. 2004 Jan;23(1):55–81, vi. 7. Aicale R, Tarantino D, Maffulli N. Overuse injuries in sport: a comprehensive overview. J Orthop Surg Res. 2018 Dec;13(1):309. 8. Yang J, Tibbetts AS, Covassin T, Cheng G, Nayar S, Heiden E. Epidemiology of overuse and acute injuries among competitive collegiate athletes. J Athl Train. 2012;47(2):198–204. 9. Bernetti A, Mangone M, Paoloni M, Di Sante L, Murgia M, Santilli V. Corticosteroid and hyaluronic acid injection therapy in tennis elbow (lateral epicondylalgia). Med Sport. 2014;67(2):289–95. 10. Iolascon G, Gimigliano F, Moretti A, De Sire A, Migliore A, Brandi ML, et al. Early osteoarthritis: How to define, diagnose, and manage. A systematic review. Eur Geriatr Med. 2017;8(5–6):383–96. 11. Drawer S, Fuller CW. Propensity for osteoarthritis and lower limb joint pain in retired professional soccer players. Br J Sports Med. 2001;35(6):402–8. 12. Vannini F, Spalding T, Andriolo L, Berruto M, Denti M, Espregueira-Mendes J, et al. Sport and early osteoarthritis: the role of sport in aetiology, progression and treatment of knee osteoarthritis. Knee Surgery, Sport Traumatol Arthrosc. 2016;24(6):1786–96. 13. Fröhlich S, Pazeller S, Cherati AS, Müller E, Frey WO, Spörri J. Overuse injuries in the knee, back and hip of top elite female alpine skiers during  the off-season preparation period: prevalence, severity and their association with traumatic preinjuries and training load. BMJ open Sport Exerc Med. 2020;6(1):e000892. 14. Rincon GA, Vyas D, Zhou J, Fu FH, Oiestad BE, Holm I, et al. \“ Knee function and prevalence of knee osteoarthritis after anterior cruciate ligament reconstruction: a prospective study with 10 to 15 years of follow-up\”. Letter to the editor. Am J Sports Med. 2011;39(4):NP3-author. 15. Kellgren JH, Lawrence J. Radiological assessment of osteo-arthrosis. Ann Rheum Dis. 1957;16(4):494. 16. Altman R, Asch E, Bloch D, Bole G, Borenstein D, Brandt K, et al. Development of criteria for the classification and reporting of osteoarthritis: classification of osteoarthritis of the knee. Arthritis Rheum Off J Am Coll Rheumatol. 1986;29(8):1039–49. 17. Luyten FP, Denti M, Filardo G, Kon E, Engebretsen L. Definition and classification of early osteoarthritis of the knee. Knee Surgery, Sport Traumatol Arthrosc. 2012;20(3):401–6. 18. Hoemann C, Kandel R, Roberts S, Saris DBF, Creemers L, Mainil-Varlet P, et al. International Cartilage Repair Society (ICRS) Recommended Guidelines for  Histological Endpoints for Cartilage Repair Studies in Animal Models and Clinical Trials. Cartilage. 2011 Apr;2(2):153–72. 19. Hunter DJ, Lo GH, Gale D, Grainger AJ, Guermazi A, Conaghan PG. The reliability of a new scoring system for knee osteoarthritis MRI and the validity of bone marrow lesion assessment: BLOKS (Boston–Leeds Osteoarthritis Knee Score). Ann Rheum Dis. 2008;67(2):206–11. 20. Peterfy CG, Guermazi A, Zaim S, Tirman PFJ, Miaux Y, White D, et al. Whole-organ magnetic resonance imaging score (WORMS) of the knee in osteoarthritis. Osteoarthr Cartil. 2004;12(3):177–90. 21. Demange MK, Sisto M, Rodeo S. Future trends for unicompartmental arthritis of the knee: injectables & stem cells. Clin Sports Med. 2014;33(1):161–74. 22. Leslie M. Knee osteoarthritis management therapies. Pain Manag Nurs. 2000;1(2):51–7. 23. Paoloni M, Bernetti A, Belelli A, Brignoli O, Buoso S, Caputi AP, et al. Appropriateness of clinical and organizational criteria for intra-articular injection therapies in osteoarthritis: A Delphi method consensus initiative among experts in Italy. Ann Ist Super Sanita. 2015;51:131–8. 24. Sandmark H, Hogstedt C, Lewold S, Vingård E. Osteoarthrosis of the knee in men and women in association with overweight, smoking, and hormone therapy. Ann Rheum Dis. 1999;58(3):151–5. 25. Christensen BV, Iuhl IU, Vilbek H, Bülow H, Dreijer NC, Rasmussen HF. Acupuncture treatment of severe knee osteoarthrosis. A long‐term study. Acta Anaesthesiol Scand. 1992;36(6):519–25. 26. de Sire A, Agostini F, Lippi L, Mangone M, Marchese S, Cisari C, et al. Oxygen-Ozone Therapy in the Rehabilitation Field: State of the Art on Mechanisms of  Action, Safety and Effectiveness in Patients with Musculoskeletal Disorders. Biomolecules. 2021 Feb;11(3). 27. Fransen M, McCONNELL S. Land-based exercise for osteoarthritis of the knee: a metaanalysis of randomized controlled trials. J Rheumatol. 2009;36(6):1109–17. 28. Hiemstra LA, Kerslake S, Irving C. Anterior knee pain in the athlete. Clin Sports Med. 2014;33(3):437–59. 29. Filardo G, Kon E, Di Matteo B, Pelotti P, Di Martino A, Marcacci M. Platelet-rich plasma for the treatment of patellar tendinopathy: clinical and imaging findings at medium-term follow-up. Int Orthop [Internet]. 2013;37(8):1583–9. Available from: https://doi.org/10.1007/s00264-013-1972-830. Trojian TH, Concoff AL, Joy SM, Hatzenbuehler JR, Saulsberry WJ, Coleman CI. AMSSM Scientific Statement Concerning Viscosupplementation Injections for Knee  Osteoarthritis: Importance for Individual Patient Outcomes. Clin J Sport Med  Off J Can Acad  Sport Med. 2016 Jan;26(1):1–11. 31. Ammar TY, Pereira TAP, Mistura SLL, Kuhn A, Saggin JI, Lopes OV. Viscosupplementation for treating knee osteoarthrosis: review of the literature. Rev Bras Ortop. 2015;50:489–94. 32. Salini V, Pantalone A, Vanni D, Abate M. Viscosupplementation in Athletes. In: Arthroscopy and Sport Injuries. Springer; 2016. p. 433–7. 33. Erdem Y, Gul D, Akpancar S. Comparison of Intraarticular Injections of Hyaluronic Acid versus Dextrose Applied with Periarticular Prolotherapy in the Treatment of Recreational Athletes with Knee Osteoarthritis. Turkish J Sport Med. 2020;55(1):6–13. 34. Kalkhoven J, Coutts AJ, Impellizzeri FM. 'Training load error' is not a more accurate term than 'overuse' injury. Br J Sports Med [Internet]. 2020 Aug 1;54(15):934 LP – 935. Available from: http://bjsm.bmj.com/content/54/15/934.abstract35. Odole AC, Agbomeji OT, Onyeso OKK, Ojo JO, Odunaiya NA. Perspectives of Nigerian Athletes About Physiotherapy Services in Sports Injury Management: Implications for Rehabilitation. J Sport Rehabil. 2021;1(aop):1–8. 36. Hendrika W, Reswari A. The effect of physiotherapy on pain improvement in patients with early knee osteoarthritis at RSU UKI. Int J Med Heal Res. 2021;7(6):52–9. 37. MacKay C, Hawker GA, Jaglal SB. How Do Physical Therapists Approach Management of People With Early Knee Osteoarthritis? A Qualitative Study. Phys Ther [Internet]. 2019;100(2):295–306. Available from: https://doi.org/10.1093/ptj/pzz16438. Hrazdira L, Pilecki Z, Pilecki G. Ultrasound guided application of HA in the soft tissues of the locomotor system in athletes–First experience. Krátká sdělení Botek, M, Stejskal, P, Klimešová, I Vliv normobarické hypoxie na Akt Auton Nerv systému Pilot Stud 191 Drbošalová, V, Stejskal, P, Klugar, M, Krejčíř, V, Hynková, O Charakt klientů komerčn. :205. 39. Ricci M, Micheloni GM, Berti M, Perusi F, Sambugaro E, Vecchini E, et al. Clinical comparison of oral administration and viscosupplementation of hyaluronic acid (HA) in early knee osteoarthritis. Musculoskelet Surg. 2017;101(1):45–9. 40. Wang C-T, Lin Y-T, Chiang B-L, Lin Y-H, Hou S-M. High molecular weight hyaluronic acid down-regulates the gene expression of osteoarthritis-associated cytokines and enzymes in fibroblast-like synoviocytes from patients with early osteoarthritis. Osteoarthr Cartil. 2006;14(12):1237–47. 41. Irrgang JJ, Anderson AF. Development and validation of health-related quality of life measures for the knee. Clin Orthop Relat Res. 2002;402:95–109. 42. Roos EM, Roos HP, Lohmander LS, Ekdahl C, Beynnon BD. Knee Injury and Osteoarthritis Outcome Score (KOOS)—development of a self-administered outcome measure. J Orthop Sport Phys Ther. 1998;28(2):88–96. 43. Perruccio A V, Canizares M, Hawker GA, Roos EM, Maillefert JF, Lohmander SL. Comparative Validity and Responsiveness of the HOOS-PS and KOOS-PS to the WOMAC Physical Function Subscale in Total Joint Replacement for Osteoarthritis. 44. Salavati M, Mazaheri M, Negahban H, Sohani SM, Ebrahimian MR, Ebrahimi I, et al. Validation of a Persian-version of Knee injury and Osteoarthritis Outcome Score (KOOS) in Iranians with knee injuries. Osteoarthr Cartil. 2008;16(10):1178–82. 45. Higgins LD, Taylor MK, Park D, Ghodadra N, Marchant M, Pietrobon R, et al. Reliability and validity of the International Knee Documentation Committee (IKDC) subjective knee form. Jt Bone Spine. 2007;74(6):594–9. 46. Kujala UM, Kaprio J, Sarno S. Osteoarthritis of weight bearing joints of lower limbs in former elite male athletes. Bmj. 1994;308(6923):231–4. 47. Salini V, Pantalone A, Vanni D. Arthroscopy and Sport Injuries. Arthrosc Sport Inj. 2016;433–7. 48. Broglio K. Randomization in clinical trials: Permuted blocks and stratification. JAMA - J Am Med Assoc. 2018;319(21):2223–4. 49. Emanuel EJ, Wendler D, Grady C. What makes clinical research ethical? JAMA. 2000 May;283(20):2701–11. )

[12.	Vannini F, Spalding T, Andriolo L, Berruto M, Denti M, Espregueira-Mendes J, et al. Sport and early osteoarthritis: the role of sport in aetiology, progression and treatment of knee osteoarthritis. Knee Surgery, Sport Traumatol Arthrosc. 2016;24(6):1786–96.](1. Patel DR, Villalobos A. Evaluation and management of knee pain in young athletes: overuse injuries of the knee. Transl Pediatr [Internet]. 2017 Jul;6(3):190–8. Available from: https://pubmed.ncbi.nlm.nih.gov/287950102. Nicolini AP, de Carvalho RT, Matsuda MM, Filho JS, Cohen M. Common injuries in athletes' knee: Experience of a specialized center. Acta Ortop Bras. 2014;22(3):127–31. 3. Tiu T, Craig Van Dien MD. Knee overuse disorders. 4. Fuller CW, Ekstrand J, Junge A, Andersen TE, Bahr R, Dvorak J, et al. Consensus statement on injury definitions and data collection procedures in studies  of football (soccer) injuries. Br J Sports Med. 2006 Mar;40(3):193–201. 5. Majewski M, Susanne H, Klaus S. Epidemiology of athletic knee injuries: A 10-year study. Knee. 2006 Jun;13(3):184–8. 6. Wilder RP, Sethi S. Overuse injuries: tendinopathies, stress fractures, compartment syndrome, and shin  splints. Clin Sports Med. 2004 Jan;23(1):55–81, vi. 7. Aicale R, Tarantino D, Maffulli N. Overuse injuries in sport: a comprehensive overview. J Orthop Surg Res. 2018 Dec;13(1):309. 8. Yang J, Tibbetts AS, Covassin T, Cheng G, Nayar S, Heiden E. Epidemiology of overuse and acute injuries among competitive collegiate athletes. J Athl Train. 2012;47(2):198–204. 9. Bernetti A, Mangone M, Paoloni M, Di Sante L, Murgia M, Santilli V. Corticosteroid and hyaluronic acid injection therapy in tennis elbow (lateral epicondylalgia). Med Sport. 2014;67(2):289–95. 10. Iolascon G, Gimigliano F, Moretti A, De Sire A, Migliore A, Brandi ML, et al. Early osteoarthritis: How to define, diagnose, and manage. A systematic review. Eur Geriatr Med. 2017;8(5–6):383–96. 11. Drawer S, Fuller CW. Propensity for osteoarthritis and lower limb joint pain in retired professional soccer players. Br J Sports Med. 2001;35(6):402–8. 12. Vannini F, Spalding T, Andriolo L, Berruto M, Denti M, Espregueira-Mendes J, et al. Sport and early osteoarthritis: the role of sport in aetiology, progression and treatment of knee osteoarthritis. Knee Surgery, Sport Traumatol Arthrosc. 2016;24(6):1786–96. 13. Fröhlich S, Pazeller S, Cherati AS, Müller E, Frey WO, Spörri J. Overuse injuries in the knee, back and hip of top elite female alpine skiers during  the off-season preparation period: prevalence, severity and their association with traumatic preinjuries and training load. BMJ open Sport Exerc Med. 2020;6(1):e000892. 14. Rincon GA, Vyas D, Zhou J, Fu FH, Oiestad BE, Holm I, et al. \“ Knee function and prevalence of knee osteoarthritis after anterior cruciate ligament reconstruction: a prospective study with 10 to 15 years of follow-up\”. Letter to the editor. Am J Sports Med. 2011;39(4):NP3-author. 15. Kellgren JH, Lawrence J. Radiological assessment of osteo-arthrosis. Ann Rheum Dis. 1957;16(4):494. 16. Altman R, Asch E, Bloch D, Bole G, Borenstein D, Brandt K, et al. Development of criteria for the classification and reporting of osteoarthritis: classification of osteoarthritis of the knee. Arthritis Rheum Off J Am Coll Rheumatol. 1986;29(8):1039–49. 17. Luyten FP, Denti M, Filardo G, Kon E, Engebretsen L. Definition and classification of early osteoarthritis of the knee. Knee Surgery, Sport Traumatol Arthrosc. 2012;20(3):401–6. 18. Hoemann C, Kandel R, Roberts S, Saris DBF, Creemers L, Mainil-Varlet P, et al. International Cartilage Repair Society (ICRS) Recommended Guidelines for  Histological Endpoints for Cartilage Repair Studies in Animal Models and Clinical Trials. Cartilage. 2011 Apr;2(2):153–72. 19. Hunter DJ, Lo GH, Gale D, Grainger AJ, Guermazi A, Conaghan PG. The reliability of a new scoring system for knee osteoarthritis MRI and the validity of bone marrow lesion assessment: BLOKS (Boston–Leeds Osteoarthritis Knee Score). Ann Rheum Dis. 2008;67(2):206–11. 20. Peterfy CG, Guermazi A, Zaim S, Tirman PFJ, Miaux Y, White D, et al. Whole-organ magnetic resonance imaging score (WORMS) of the knee in osteoarthritis. Osteoarthr Cartil. 2004;12(3):177–90. 21. Demange MK, Sisto M, Rodeo S. Future trends for unicompartmental arthritis of the knee: injectables & stem cells. Clin Sports Med. 2014;33(1):161–74. 22. Leslie M. Knee osteoarthritis management therapies. Pain Manag Nurs. 2000;1(2):51–7. 23. Paoloni M, Bernetti A, Belelli A, Brignoli O, Buoso S, Caputi AP, et al. Appropriateness of clinical and organizational criteria for intra-articular injection therapies in osteoarthritis: A Delphi method consensus initiative among experts in Italy. Ann Ist Super Sanita. 2015;51:131–8. 24. Sandmark H, Hogstedt C, Lewold S, Vingård E. Osteoarthrosis of the knee in men and women in association with overweight, smoking, and hormone therapy. Ann Rheum Dis. 1999;58(3):151–5. 25. Christensen BV, Iuhl IU, Vilbek H, Bülow H, Dreijer NC, Rasmussen HF. Acupuncture treatment of severe knee osteoarthrosis. A long‐term study. Acta Anaesthesiol Scand. 1992;36(6):519–25. 26. de Sire A, Agostini F, Lippi L, Mangone M, Marchese S, Cisari C, et al. Oxygen-Ozone Therapy in the Rehabilitation Field: State of the Art on Mechanisms of  Action, Safety and Effectiveness in Patients with Musculoskeletal Disorders. Biomolecules. 2021 Feb;11(3). 27. Fransen M, McCONNELL S. Land-based exercise for osteoarthritis of the knee: a metaanalysis of randomized controlled trials. J Rheumatol. 2009;36(6):1109–17. 28. Hiemstra LA, Kerslake S, Irving C. Anterior knee pain in the athlete. Clin Sports Med. 2014;33(3):437–59. 29. Filardo G, Kon E, Di Matteo B, Pelotti P, Di Martino A, Marcacci M. Platelet-rich plasma for the treatment of patellar tendinopathy: clinical and imaging findings at medium-term follow-up. Int Orthop [Internet]. 2013;37(8):1583–9. Available from: https://doi.org/10.1007/s00264-013-1972-830. Trojian TH, Concoff AL, Joy SM, Hatzenbuehler JR, Saulsberry WJ, Coleman CI. AMSSM Scientific Statement Concerning Viscosupplementation Injections for Knee  Osteoarthritis: Importance for Individual Patient Outcomes. Clin J Sport Med  Off J Can Acad  Sport Med. 2016 Jan;26(1):1–11. 31. Ammar TY, Pereira TAP, Mistura SLL, Kuhn A, Saggin JI, Lopes OV. Viscosupplementation for treating knee osteoarthrosis: review of the literature. Rev Bras Ortop. 2015;50:489–94. 32. Salini V, Pantalone A, Vanni D, Abate M. Viscosupplementation in Athletes. In: Arthroscopy and Sport Injuries. Springer; 2016. p. 433–7. 33. Erdem Y, Gul D, Akpancar S. Comparison of Intraarticular Injections of Hyaluronic Acid versus Dextrose Applied with Periarticular Prolotherapy in the Treatment of Recreational Athletes with Knee Osteoarthritis. Turkish J Sport Med. 2020;55(1):6–13. 34. Kalkhoven J, Coutts AJ, Impellizzeri FM. 'Training load error' is not a more accurate term than 'overuse' injury. Br J Sports Med [Internet]. 2020 Aug 1;54(15):934 LP – 935. Available from: http://bjsm.bmj.com/content/54/15/934.abstract35. Odole AC, Agbomeji OT, Onyeso OKK, Ojo JO, Odunaiya NA. Perspectives of Nigerian Athletes About Physiotherapy Services in Sports Injury Management: Implications for Rehabilitation. J Sport Rehabil. 2021;1(aop):1–8. 36. Hendrika W, Reswari A. The effect of physiotherapy on pain improvement in patients with early knee osteoarthritis at RSU UKI. Int J Med Heal Res. 2021;7(6):52–9. 37. MacKay C, Hawker GA, Jaglal SB. How Do Physical Therapists Approach Management of People With Early Knee Osteoarthritis? A Qualitative Study. Phys Ther [Internet]. 2019;100(2):295–306. Available from: https://doi.org/10.1093/ptj/pzz16438. Hrazdira L, Pilecki Z, Pilecki G. Ultrasound guided application of HA in the soft tissues of the locomotor system in athletes–First experience. Krátká sdělení Botek, M, Stejskal, P, Klimešová, I Vliv normobarické hypoxie na Akt Auton Nerv systému Pilot Stud 191 Drbošalová, V, Stejskal, P, Klugar, M, Krejčíř, V, Hynková, O Charakt klientů komerčn. :205. 39. Ricci M, Micheloni GM, Berti M, Perusi F, Sambugaro E, Vecchini E, et al. Clinical comparison of oral administration and viscosupplementation of hyaluronic acid (HA) in early knee osteoarthritis. Musculoskelet Surg. 2017;101(1):45–9. 40. Wang C-T, Lin Y-T, Chiang B-L, Lin Y-H, Hou S-M. High molecular weight hyaluronic acid down-regulates the gene expression of osteoarthritis-associated cytokines and enzymes in fibroblast-like synoviocytes from patients with early osteoarthritis. Osteoarthr Cartil. 2006;14(12):1237–47. 41. Irrgang JJ, Anderson AF. Development and validation of health-related quality of life measures for the knee. Clin Orthop Relat Res. 2002;402:95–109. 42. Roos EM, Roos HP, Lohmander LS, Ekdahl C, Beynnon BD. Knee Injury and Osteoarthritis Outcome Score (KOOS)—development of a self-administered outcome measure. J Orthop Sport Phys Ther. 1998;28(2):88–96. 43. Perruccio A V, Canizares M, Hawker GA, Roos EM, Maillefert JF, Lohmander SL. Comparative Validity and Responsiveness of the HOOS-PS and KOOS-PS to the WOMAC Physical Function Subscale in Total Joint Replacement for Osteoarthritis. 44. Salavati M, Mazaheri M, Negahban H, Sohani SM, Ebrahimian MR, Ebrahimi I, et al. Validation of a Persian-version of Knee injury and Osteoarthritis Outcome Score (KOOS) in Iranians with knee injuries. Osteoarthr Cartil. 2008;16(10):1178–82. 45. Higgins LD, Taylor MK, Park D, Ghodadra N, Marchant M, Pietrobon R, et al. Reliability and validity of the International Knee Documentation Committee (IKDC) subjective knee form. Jt Bone Spine. 2007;74(6):594–9. 46. Kujala UM, Kaprio J, Sarno S. Osteoarthritis of weight bearing joints of lower limbs in former elite male athletes. Bmj. 1994;308(6923):231–4. 47. Salini V, Pantalone A, Vanni D. Arthroscopy and Sport Injuries. Arthrosc Sport Inj. 2016;433–7. 48. Broglio K. Randomization in clinical trials: Permuted blocks and stratification. JAMA - J Am Med Assoc. 2018;319(21):2223–4. 49. Emanuel EJ, Wendler D, Grady C. What makes clinical research ethical? JAMA. 2000 May;283(20):2701–11. )

[13.	Fröhlich S, Pazeller S, Cherati AS, Müller E, Frey WO, Spörri J. Overuse injuries in the knee, back and hip of top elite female alpine skiers during the off-season preparation period: prevalence, severity and their association with traumatic preinjuries and training load. BMJ open Sport Exerc Med. 2020;6(1):e000892.](1. Patel DR, Villalobos A. Evaluation and management of knee pain in young athletes: overuse injuries of the knee. Transl Pediatr [Internet]. 2017 Jul;6(3):190–8. Available from: https://pubmed.ncbi.nlm.nih.gov/287950102. Nicolini AP, de Carvalho RT, Matsuda MM, Filho JS, Cohen M. Common injuries in athletes' knee: Experience of a specialized center. Acta Ortop Bras. 2014;22(3):127–31. 3. Tiu T, Craig Van Dien MD. Knee overuse disorders. 4. Fuller CW, Ekstrand J, Junge A, Andersen TE, Bahr R, Dvorak J, et al. Consensus statement on injury definitions and data collection procedures in studies  of football (soccer) injuries. Br J Sports Med. 2006 Mar;40(3):193–201. 5. Majewski M, Susanne H, Klaus S. Epidemiology of athletic knee injuries: A 10-year study. Knee. 2006 Jun;13(3):184–8. 6. Wilder RP, Sethi S. Overuse injuries: tendinopathies, stress fractures, compartment syndrome, and shin  splints. Clin Sports Med. 2004 Jan;23(1):55–81, vi. 7. Aicale R, Tarantino D, Maffulli N. Overuse injuries in sport: a comprehensive overview. J Orthop Surg Res. 2018 Dec;13(1):309. 8. Yang J, Tibbetts AS, Covassin T, Cheng G, Nayar S, Heiden E. Epidemiology of overuse and acute injuries among competitive collegiate athletes. J Athl Train. 2012;47(2):198–204. 9. Bernetti A, Mangone M, Paoloni M, Di Sante L, Murgia M, Santilli V. Corticosteroid and hyaluronic acid injection therapy in tennis elbow (lateral epicondylalgia). Med Sport. 2014;67(2):289–95. 10. Iolascon G, Gimigliano F, Moretti A, De Sire A, Migliore A, Brandi ML, et al. Early osteoarthritis: How to define, diagnose, and manage. A systematic review. Eur Geriatr Med. 2017;8(5–6):383–96. 11. Drawer S, Fuller CW. Propensity for osteoarthritis and lower limb joint pain in retired professional soccer players. Br J Sports Med. 2001;35(6):402–8. 12. Vannini F, Spalding T, Andriolo L, Berruto M, Denti M, Espregueira-Mendes J, et al. Sport and early osteoarthritis: the role of sport in aetiology, progression and treatment of knee osteoarthritis. Knee Surgery, Sport Traumatol Arthrosc. 2016;24(6):1786–96. 13. Fröhlich S, Pazeller S, Cherati AS, Müller E, Frey WO, Spörri J. Overuse injuries in the knee, back and hip of top elite female alpine skiers during  the off-season preparation period: prevalence, severity and their association with traumatic preinjuries and training load. BMJ open Sport Exerc Med. 2020;6(1):e000892. 14. Rincon GA, Vyas D, Zhou J, Fu FH, Oiestad BE, Holm I, et al. \“ Knee function and prevalence of knee osteoarthritis after anterior cruciate ligament reconstruction: a prospective study with 10 to 15 years of follow-up\”. Letter to the editor. Am J Sports Med. 2011;39(4):NP3-author. 15. Kellgren JH, Lawrence J. Radiological assessment of osteo-arthrosis. Ann Rheum Dis. 1957;16(4):494. 16. Altman R, Asch E, Bloch D, Bole G, Borenstein D, Brandt K, et al. Development of criteria for the classification and reporting of osteoarthritis: classification of osteoarthritis of the knee. Arthritis Rheum Off J Am Coll Rheumatol. 1986;29(8):1039–49. 17. Luyten FP, Denti M, Filardo G, Kon E, Engebretsen L. Definition and classification of early osteoarthritis of the knee. Knee Surgery, Sport Traumatol Arthrosc. 2012;20(3):401–6. 18. Hoemann C, Kandel R, Roberts S, Saris DBF, Creemers L, Mainil-Varlet P, et al. International Cartilage Repair Society (ICRS) Recommended Guidelines for  Histological Endpoints for Cartilage Repair Studies in Animal Models and Clinical Trials. Cartilage. 2011 Apr;2(2):153–72. 19. Hunter DJ, Lo GH, Gale D, Grainger AJ, Guermazi A, Conaghan PG. The reliability of a new scoring system for knee osteoarthritis MRI and the validity of bone marrow lesion assessment: BLOKS (Boston–Leeds Osteoarthritis Knee Score). Ann Rheum Dis. 2008;67(2):206–11. 20. Peterfy CG, Guermazi A, Zaim S, Tirman PFJ, Miaux Y, White D, et al. Whole-organ magnetic resonance imaging score (WORMS) of the knee in osteoarthritis. Osteoarthr Cartil. 2004;12(3):177–90. 21. Demange MK, Sisto M, Rodeo S. Future trends for unicompartmental arthritis of the knee: injectables & stem cells. Clin Sports Med. 2014;33(1):161–74. 22. Leslie M. Knee osteoarthritis management therapies. Pain Manag Nurs. 2000;1(2):51–7. 23. Paoloni M, Bernetti A, Belelli A, Brignoli O, Buoso S, Caputi AP, et al. Appropriateness of clinical and organizational criteria for intra-articular injection therapies in osteoarthritis: A Delphi method consensus initiative among experts in Italy. Ann Ist Super Sanita. 2015;51:131–8. 24. Sandmark H, Hogstedt C, Lewold S, Vingård E. Osteoarthrosis of the knee in men and women in association with overweight, smoking, and hormone therapy. Ann Rheum Dis. 1999;58(3):151–5. 25. Christensen BV, Iuhl IU, Vilbek H, Bülow H, Dreijer NC, Rasmussen HF. Acupuncture treatment of severe knee osteoarthrosis. A long‐term study. Acta Anaesthesiol Scand. 1992;36(6):519–25. 26. de Sire A, Agostini F, Lippi L, Mangone M, Marchese S, Cisari C, et al. Oxygen-Ozone Therapy in the Rehabilitation Field: State of the Art on Mechanisms of  Action, Safety and Effectiveness in Patients with Musculoskeletal Disorders. Biomolecules. 2021 Feb;11(3). 27. Fransen M, McCONNELL S. Land-based exercise for osteoarthritis of the knee: a metaanalysis of randomized controlled trials. J Rheumatol. 2009;36(6):1109–17. 28. Hiemstra LA, Kerslake S, Irving C. Anterior knee pain in the athlete. Clin Sports Med. 2014;33(3):437–59. 29. Filardo G, Kon E, Di Matteo B, Pelotti P, Di Martino A, Marcacci M. Platelet-rich plasma for the treatment of patellar tendinopathy: clinical and imaging findings at medium-term follow-up. Int Orthop [Internet]. 2013;37(8):1583–9. Available from: https://doi.org/10.1007/s00264-013-1972-830. Trojian TH, Concoff AL, Joy SM, Hatzenbuehler JR, Saulsberry WJ, Coleman CI. AMSSM Scientific Statement Concerning Viscosupplementation Injections for Knee  Osteoarthritis: Importance for Individual Patient Outcomes. Clin J Sport Med  Off J Can Acad  Sport Med. 2016 Jan;26(1):1–11. 31. Ammar TY, Pereira TAP, Mistura SLL, Kuhn A, Saggin JI, Lopes OV. Viscosupplementation for treating knee osteoarthrosis: review of the literature. Rev Bras Ortop. 2015;50:489–94. 32. Salini V, Pantalone A, Vanni D, Abate M. Viscosupplementation in Athletes. In: Arthroscopy and Sport Injuries. Springer; 2016. p. 433–7. 33. Erdem Y, Gul D, Akpancar S. Comparison of Intraarticular Injections of Hyaluronic Acid versus Dextrose Applied with Periarticular Prolotherapy in the Treatment of Recreational Athletes with Knee Osteoarthritis. Turkish J Sport Med. 2020;55(1):6–13. 34. Kalkhoven J, Coutts AJ, Impellizzeri FM. 'Training load error' is not a more accurate term than 'overuse' injury. Br J Sports Med [Internet]. 2020 Aug 1;54(15):934 LP – 935. Available from: http://bjsm.bmj.com/content/54/15/934.abstract35. Odole AC, Agbomeji OT, Onyeso OKK, Ojo JO, Odunaiya NA. Perspectives of Nigerian Athletes About Physiotherapy Services in Sports Injury Management: Implications for Rehabilitation. J Sport Rehabil. 2021;1(aop):1–8. 36. Hendrika W, Reswari A. The effect of physiotherapy on pain improvement in patients with early knee osteoarthritis at RSU UKI. Int J Med Heal Res. 2021;7(6):52–9. 37. MacKay C, Hawker GA, Jaglal SB. How Do Physical Therapists Approach Management of People With Early Knee Osteoarthritis? A Qualitative Study. Phys Ther [Internet]. 2019;100(2):295–306. Available from: https://doi.org/10.1093/ptj/pzz16438. Hrazdira L, Pilecki Z, Pilecki G. Ultrasound guided application of HA in the soft tissues of the locomotor system in athletes–First experience. Krátká sdělení Botek, M, Stejskal, P, Klimešová, I Vliv normobarické hypoxie na Akt Auton Nerv systému Pilot Stud 191 Drbošalová, V, Stejskal, P, Klugar, M, Krejčíř, V, Hynková, O Charakt klientů komerčn. :205. 39. Ricci M, Micheloni GM, Berti M, Perusi F, Sambugaro E, Vecchini E, et al. Clinical comparison of oral administration and viscosupplementation of hyaluronic acid (HA) in early knee osteoarthritis. Musculoskelet Surg. 2017;101(1):45–9. 40. Wang C-T, Lin Y-T, Chiang B-L, Lin Y-H, Hou S-M. High molecular weight hyaluronic acid down-regulates the gene expression of osteoarthritis-associated cytokines and enzymes in fibroblast-like synoviocytes from patients with early osteoarthritis. Osteoarthr Cartil. 2006;14(12):1237–47. 41. Irrgang JJ, Anderson AF. Development and validation of health-related quality of life measures for the knee. Clin Orthop Relat Res. 2002;402:95–109. 42. Roos EM, Roos HP, Lohmander LS, Ekdahl C, Beynnon BD. Knee Injury and Osteoarthritis Outcome Score (KOOS)—development of a self-administered outcome measure. J Orthop Sport Phys Ther. 1998;28(2):88–96. 43. Perruccio A V, Canizares M, Hawker GA, Roos EM, Maillefert JF, Lohmander SL. Comparative Validity and Responsiveness of the HOOS-PS and KOOS-PS to the WOMAC Physical Function Subscale in Total Joint Replacement for Osteoarthritis. 44. Salavati M, Mazaheri M, Negahban H, Sohani SM, Ebrahimian MR, Ebrahimi I, et al. Validation of a Persian-version of Knee injury and Osteoarthritis Outcome Score (KOOS) in Iranians with knee injuries. Osteoarthr Cartil. 2008;16(10):1178–82. 45. Higgins LD, Taylor MK, Park D, Ghodadra N, Marchant M, Pietrobon R, et al. Reliability and validity of the International Knee Documentation Committee (IKDC) subjective knee form. Jt Bone Spine. 2007;74(6):594–9. 46. Kujala UM, Kaprio J, Sarno S. Osteoarthritis of weight bearing joints of lower limbs in former elite male athletes. Bmj. 1994;308(6923):231–4. 47. Salini V, Pantalone A, Vanni D. Arthroscopy and Sport Injuries. Arthrosc Sport Inj. 2016;433–7. 48. Broglio K. Randomization in clinical trials: Permuted blocks and stratification. JAMA - J Am Med Assoc. 2018;319(21):2223–4. 49. Emanuel EJ, Wendler D, Grady C. What makes clinical research ethical? JAMA. 2000 May;283(20):2701–11. )

[14.	Rincon GA, Vyas D, Zhou J, Fu FH, Oiestad BE, Holm I, et al. “ Knee function and prevalence of knee osteoarthritis after anterior cruciate ligament reconstruction: a prospective study with 10 to 15 years of follow-up”. Letter to the editor. Am J Sports Med. 2011;39(4):NP3-author.](1. Patel DR, Villalobos A. Evaluation and management of knee pain in young athletes: overuse injuries of the knee. Transl Pediatr [Internet]. 2017 Jul;6(3):190–8. Available from: https://pubmed.ncbi.nlm.nih.gov/287950102. Nicolini AP, de Carvalho RT, Matsuda MM, Filho JS, Cohen M. Common injuries in athletes' knee: Experience of a specialized center. Acta Ortop Bras. 2014;22(3):127–31. 3. Tiu T, Craig Van Dien MD. Knee overuse disorders. 4. Fuller CW, Ekstrand J, Junge A, Andersen TE, Bahr R, Dvorak J, et al. Consensus statement on injury definitions and data collection procedures in studies  of football (soccer) injuries. Br J Sports Med. 2006 Mar;40(3):193–201. 5. Majewski M, Susanne H, Klaus S. Epidemiology of athletic knee injuries: A 10-year study. Knee. 2006 Jun;13(3):184–8. 6. Wilder RP, Sethi S. Overuse injuries: tendinopathies, stress fractures, compartment syndrome, and shin  splints. Clin Sports Med. 2004 Jan;23(1):55–81, vi. 7. Aicale R, Tarantino D, Maffulli N. Overuse injuries in sport: a comprehensive overview. J Orthop Surg Res. 2018 Dec;13(1):309. 8. Yang J, Tibbetts AS, Covassin T, Cheng G, Nayar S, Heiden E. Epidemiology of overuse and acute injuries among competitive collegiate athletes. J Athl Train. 2012;47(2):198–204. 9. Bernetti A, Mangone M, Paoloni M, Di Sante L, Murgia M, Santilli V. Corticosteroid and hyaluronic acid injection therapy in tennis elbow (lateral epicondylalgia). Med Sport. 2014;67(2):289–95. 10. Iolascon G, Gimigliano F, Moretti A, De Sire A, Migliore A, Brandi ML, et al. Early osteoarthritis: How to define, diagnose, and manage. A systematic review. Eur Geriatr Med. 2017;8(5–6):383–96. 11. Drawer S, Fuller CW. Propensity for osteoarthritis and lower limb joint pain in retired professional soccer players. Br J Sports Med. 2001;35(6):402–8. 12. Vannini F, Spalding T, Andriolo L, Berruto M, Denti M, Espregueira-Mendes J, et al. Sport and early osteoarthritis: the role of sport in aetiology, progression and treatment of knee osteoarthritis. Knee Surgery, Sport Traumatol Arthrosc. 2016;24(6):1786–96. 13. Fröhlich S, Pazeller S, Cherati AS, Müller E, Frey WO, Spörri J. Overuse injuries in the knee, back and hip of top elite female alpine skiers during  the off-season preparation period: prevalence, severity and their association with traumatic preinjuries and training load. BMJ open Sport Exerc Med. 2020;6(1):e000892. 14. Rincon GA, Vyas D, Zhou J, Fu FH, Oiestad BE, Holm I, et al. \“ Knee function and prevalence of knee osteoarthritis after anterior cruciate ligament reconstruction: a prospective study with 10 to 15 years of follow-up\”. Letter to the editor. Am J Sports Med. 2011;39(4):NP3-author. 15. Kellgren JH, Lawrence J. Radiological assessment of osteo-arthrosis. Ann Rheum Dis. 1957;16(4):494. 16. Altman R, Asch E, Bloch D, Bole G, Borenstein D, Brandt K, et al. Development of criteria for the classification and reporting of osteoarthritis: classification of osteoarthritis of the knee. Arthritis Rheum Off J Am Coll Rheumatol. 1986;29(8):1039–49. 17. Luyten FP, Denti M, Filardo G, Kon E, Engebretsen L. Definition and classification of early osteoarthritis of the knee. Knee Surgery, Sport Traumatol Arthrosc. 2012;20(3):401–6. 18. Hoemann C, Kandel R, Roberts S, Saris DBF, Creemers L, Mainil-Varlet P, et al. International Cartilage Repair Society (ICRS) Recommended Guidelines for  Histological Endpoints for Cartilage Repair Studies in Animal Models and Clinical Trials. Cartilage. 2011 Apr;2(2):153–72. 19. Hunter DJ, Lo GH, Gale D, Grainger AJ, Guermazi A, Conaghan PG. The reliability of a new scoring system for knee osteoarthritis MRI and the validity of bone marrow lesion assessment: BLOKS (Boston–Leeds Osteoarthritis Knee Score). Ann Rheum Dis. 2008;67(2):206–11. 20. Peterfy CG, Guermazi A, Zaim S, Tirman PFJ, Miaux Y, White D, et al. Whole-organ magnetic resonance imaging score (WORMS) of the knee in osteoarthritis. Osteoarthr Cartil. 2004;12(3):177–90. 21. Demange MK, Sisto M, Rodeo S. Future trends for unicompartmental arthritis of the knee: injectables & stem cells. Clin Sports Med. 2014;33(1):161–74. 22. Leslie M. Knee osteoarthritis management therapies. Pain Manag Nurs. 2000;1(2):51–7. 23. Paoloni M, Bernetti A, Belelli A, Brignoli O, Buoso S, Caputi AP, et al. Appropriateness of clinical and organizational criteria for intra-articular injection therapies in osteoarthritis: A Delphi method consensus initiative among experts in Italy. Ann Ist Super Sanita. 2015;51:131–8. 24. Sandmark H, Hogstedt C, Lewold S, Vingård E. Osteoarthrosis of the knee in men and women in association with overweight, smoking, and hormone therapy. Ann Rheum Dis. 1999;58(3):151–5. 25. Christensen BV, Iuhl IU, Vilbek H, Bülow H, Dreijer NC, Rasmussen HF. Acupuncture treatment of severe knee osteoarthrosis. A long‐term study. Acta Anaesthesiol Scand. 1992;36(6):519–25. 26. de Sire A, Agostini F, Lippi L, Mangone M, Marchese S, Cisari C, et al. Oxygen-Ozone Therapy in the Rehabilitation Field: State of the Art on Mechanisms of  Action, Safety and Effectiveness in Patients with Musculoskeletal Disorders. Biomolecules. 2021 Feb;11(3). 27. Fransen M, McCONNELL S. Land-based exercise for osteoarthritis of the knee: a metaanalysis of randomized controlled trials. J Rheumatol. 2009;36(6):1109–17. 28. Hiemstra LA, Kerslake S, Irving C. Anterior knee pain in the athlete. Clin Sports Med. 2014;33(3):437–59. 29. Filardo G, Kon E, Di Matteo B, Pelotti P, Di Martino A, Marcacci M. Platelet-rich plasma for the treatment of patellar tendinopathy: clinical and imaging findings at medium-term follow-up. Int Orthop [Internet]. 2013;37(8):1583–9. Available from: https://doi.org/10.1007/s00264-013-1972-830. Trojian TH, Concoff AL, Joy SM, Hatzenbuehler JR, Saulsberry WJ, Coleman CI. AMSSM Scientific Statement Concerning Viscosupplementation Injections for Knee  Osteoarthritis: Importance for Individual Patient Outcomes. Clin J Sport Med  Off J Can Acad  Sport Med. 2016 Jan;26(1):1–11. 31. Ammar TY, Pereira TAP, Mistura SLL, Kuhn A, Saggin JI, Lopes OV. Viscosupplementation for treating knee osteoarthrosis: review of the literature. Rev Bras Ortop. 2015;50:489–94. 32. Salini V, Pantalone A, Vanni D, Abate M. Viscosupplementation in Athletes. In: Arthroscopy and Sport Injuries. Springer; 2016. p. 433–7. 33. Erdem Y, Gul D, Akpancar S. Comparison of Intraarticular Injections of Hyaluronic Acid versus Dextrose Applied with Periarticular Prolotherapy in the Treatment of Recreational Athletes with Knee Osteoarthritis. Turkish J Sport Med. 2020;55(1):6–13. 34. Kalkhoven J, Coutts AJ, Impellizzeri FM. 'Training load error' is not a more accurate term than 'overuse' injury. Br J Sports Med [Internet]. 2020 Aug 1;54(15):934 LP – 935. Available from: http://bjsm.bmj.com/content/54/15/934.abstract35. Odole AC, Agbomeji OT, Onyeso OKK, Ojo JO, Odunaiya NA. Perspectives of Nigerian Athletes About Physiotherapy Services in Sports Injury Management: Implications for Rehabilitation. J Sport Rehabil. 2021;1(aop):1–8. 36. Hendrika W, Reswari A. The effect of physiotherapy on pain improvement in patients with early knee osteoarthritis at RSU UKI. Int J Med Heal Res. 2021;7(6):52–9. 37. MacKay C, Hawker GA, Jaglal SB. How Do Physical Therapists Approach Management of People With Early Knee Osteoarthritis? A Qualitative Study. Phys Ther [Internet]. 2019;100(2):295–306. Available from: https://doi.org/10.1093/ptj/pzz16438. Hrazdira L, Pilecki Z, Pilecki G. Ultrasound guided application of HA in the soft tissues of the locomotor system in athletes–First experience. Krátká sdělení Botek, M, Stejskal, P, Klimešová, I Vliv normobarické hypoxie na Akt Auton Nerv systému Pilot Stud 191 Drbošalová, V, Stejskal, P, Klugar, M, Krejčíř, V, Hynková, O Charakt klientů komerčn. :205. 39. Ricci M, Micheloni GM, Berti M, Perusi F, Sambugaro E, Vecchini E, et al. Clinical comparison of oral administration and viscosupplementation of hyaluronic acid (HA) in early knee osteoarthritis. Musculoskelet Surg. 2017;101(1):45–9. 40. Wang C-T, Lin Y-T, Chiang B-L, Lin Y-H, Hou S-M. High molecular weight hyaluronic acid down-regulates the gene expression of osteoarthritis-associated cytokines and enzymes in fibroblast-like synoviocytes from patients with early osteoarthritis. Osteoarthr Cartil. 2006;14(12):1237–47. 41. Irrgang JJ, Anderson AF. Development and validation of health-related quality of life measures for the knee. Clin Orthop Relat Res. 2002;402:95–109. 42. Roos EM, Roos HP, Lohmander LS, Ekdahl C, Beynnon BD. Knee Injury and Osteoarthritis Outcome Score (KOOS)—development of a self-administered outcome measure. J Orthop Sport Phys Ther. 1998;28(2):88–96. 43. Perruccio A V, Canizares M, Hawker GA, Roos EM, Maillefert JF, Lohmander SL. Comparative Validity and Responsiveness of the HOOS-PS and KOOS-PS to the WOMAC Physical Function Subscale in Total Joint Replacement for Osteoarthritis. 44. Salavati M, Mazaheri M, Negahban H, Sohani SM, Ebrahimian MR, Ebrahimi I, et al. Validation of a Persian-version of Knee injury and Osteoarthritis Outcome Score (KOOS) in Iranians with knee injuries. Osteoarthr Cartil. 2008;16(10):1178–82. 45. Higgins LD, Taylor MK, Park D, Ghodadra N, Marchant M, Pietrobon R, et al. Reliability and validity of the International Knee Documentation Committee (IKDC) subjective knee form. Jt Bone Spine. 2007;74(6):594–9. 46. Kujala UM, Kaprio J, Sarno S. Osteoarthritis of weight bearing joints of lower limbs in former elite male athletes. Bmj. 1994;308(6923):231–4. 47. Salini V, Pantalone A, Vanni D. Arthroscopy and Sport Injuries. Arthrosc Sport Inj. 2016;433–7. 48. Broglio K. Randomization in clinical trials: Permuted blocks and stratification. JAMA - J Am Med Assoc. 2018;319(21):2223–4. 49. Emanuel EJ, Wendler D, Grady C. What makes clinical research ethical? JAMA. 2000 May;283(20):2701–11. )

[15.	Kellgren JH, Lawrence J. Radiological assessment of osteo-arthrosis. Ann Rheum Dis. 1957;16(4):494.](1. Patel DR, Villalobos A. Evaluation and management of knee pain in young athletes: overuse injuries of the knee. Transl Pediatr [Internet]. 2017 Jul;6(3):190–8. Available from: https://pubmed.ncbi.nlm.nih.gov/287950102. Nicolini AP, de Carvalho RT, Matsuda MM, Filho JS, Cohen M. Common injuries in athletes' knee: Experience of a specialized center. Acta Ortop Bras. 2014;22(3):127–31. 3. Tiu T, Craig Van Dien MD. Knee overuse disorders. 4. Fuller CW, Ekstrand J, Junge A, Andersen TE, Bahr R, Dvorak J, et al. Consensus statement on injury definitions and data collection procedures in studies  of football (soccer) injuries. Br J Sports Med. 2006 Mar;40(3):193–201. 5. Majewski M, Susanne H, Klaus S. Epidemiology of athletic knee injuries: A 10-year study. Knee. 2006 Jun;13(3):184–8. 6. Wilder RP, Sethi S. Overuse injuries: tendinopathies, stress fractures, compartment syndrome, and shin  splints. Clin Sports Med. 2004 Jan;23(1):55–81, vi. 7. Aicale R, Tarantino D, Maffulli N. Overuse injuries in sport: a comprehensive overview. J Orthop Surg Res. 2018 Dec;13(1):309. 8. Yang J, Tibbetts AS, Covassin T, Cheng G, Nayar S, Heiden E. Epidemiology of overuse and acute injuries among competitive collegiate athletes. J Athl Train. 2012;47(2):198–204. 9. Bernetti A, Mangone M, Paoloni M, Di Sante L, Murgia M, Santilli V. Corticosteroid and hyaluronic acid injection therapy in tennis elbow (lateral epicondylalgia). Med Sport. 2014;67(2):289–95. 10. Iolascon G, Gimigliano F, Moretti A, De Sire A, Migliore A, Brandi ML, et al. Early osteoarthritis: How to define, diagnose, and manage. A systematic review. Eur Geriatr Med. 2017;8(5–6):383–96. 11. Drawer S, Fuller CW. Propensity for osteoarthritis and lower limb joint pain in retired professional soccer players. Br J Sports Med. 2001;35(6):402–8. 12. Vannini F, Spalding T, Andriolo L, Berruto M, Denti M, Espregueira-Mendes J, et al. Sport and early osteoarthritis: the role of sport in aetiology, progression and treatment of knee osteoarthritis. Knee Surgery, Sport Traumatol Arthrosc. 2016;24(6):1786–96. 13. Fröhlich S, Pazeller S, Cherati AS, Müller E, Frey WO, Spörri J. Overuse injuries in the knee, back and hip of top elite female alpine skiers during  the off-season preparation period: prevalence, severity and their association with traumatic preinjuries and training load. BMJ open Sport Exerc Med. 2020;6(1):e000892. 14. Rincon GA, Vyas D, Zhou J, Fu FH, Oiestad BE, Holm I, et al. \“ Knee function and prevalence of knee osteoarthritis after anterior cruciate ligament reconstruction: a prospective study with 10 to 15 years of follow-up\”. Letter to the editor. Am J Sports Med. 2011;39(4):NP3-author. 15. Kellgren JH, Lawrence J. Radiological assessment of osteo-arthrosis. Ann Rheum Dis. 1957;16(4):494. 16. Altman R, Asch E, Bloch D, Bole G, Borenstein D, Brandt K, et al. Development of criteria for the classification and reporting of osteoarthritis: classification of osteoarthritis of the knee. Arthritis Rheum Off J Am Coll Rheumatol. 1986;29(8):1039–49. 17. Luyten FP, Denti M, Filardo G, Kon E, Engebretsen L. Definition and classification of early osteoarthritis of the knee. Knee Surgery, Sport Traumatol Arthrosc. 2012;20(3):401–6. 18. Hoemann C, Kandel R, Roberts S, Saris DBF, Creemers L, Mainil-Varlet P, et al. International Cartilage Repair Society (ICRS) Recommended Guidelines for  Histological Endpoints for Cartilage Repair Studies in Animal Models and Clinical Trials. Cartilage. 2011 Apr;2(2):153–72. 19. Hunter DJ, Lo GH, Gale D, Grainger AJ, Guermazi A, Conaghan PG. The reliability of a new scoring system for knee osteoarthritis MRI and the validity of bone marrow lesion assessment: BLOKS (Boston–Leeds Osteoarthritis Knee Score). Ann Rheum Dis. 2008;67(2):206–11. 20. Peterfy CG, Guermazi A, Zaim S, Tirman PFJ, Miaux Y, White D, et al. Whole-organ magnetic resonance imaging score (WORMS) of the knee in osteoarthritis. Osteoarthr Cartil. 2004;12(3):177–90. 21. Demange MK, Sisto M, Rodeo S. Future trends for unicompartmental arthritis of the knee: injectables & stem cells. Clin Sports Med. 2014;33(1):161–74. 22. Leslie M. Knee osteoarthritis management therapies. Pain Manag Nurs. 2000;1(2):51–7. 23. Paoloni M, Bernetti A, Belelli A, Brignoli O, Buoso S, Caputi AP, et al. Appropriateness of clinical and organizational criteria for intra-articular injection therapies in osteoarthritis: A Delphi method consensus initiative among experts in Italy. Ann Ist Super Sanita. 2015;51:131–8. 24. Sandmark H, Hogstedt C, Lewold S, Vingård E. Osteoarthrosis of the knee in men and women in association with overweight, smoking, and hormone therapy. Ann Rheum Dis. 1999;58(3):151–5. 25. Christensen BV, Iuhl IU, Vilbek H, Bülow H, Dreijer NC, Rasmussen HF. Acupuncture treatment of severe knee osteoarthrosis. A long‐term study. Acta Anaesthesiol Scand. 1992;36(6):519–25. 26. de Sire A, Agostini F, Lippi L, Mangone M, Marchese S, Cisari C, et al. Oxygen-Ozone Therapy in the Rehabilitation Field: State of the Art on Mechanisms of  Action, Safety and Effectiveness in Patients with Musculoskeletal Disorders. Biomolecules. 2021 Feb;11(3). 27. Fransen M, McCONNELL S. Land-based exercise for osteoarthritis of the knee: a metaanalysis of randomized controlled trials. J Rheumatol. 2009;36(6):1109–17. 28. Hiemstra LA, Kerslake S, Irving C. Anterior knee pain in the athlete. Clin Sports Med. 2014;33(3):437–59. 29. Filardo G, Kon E, Di Matteo B, Pelotti P, Di Martino A, Marcacci M. Platelet-rich plasma for the treatment of patellar tendinopathy: clinical and imaging findings at medium-term follow-up. Int Orthop [Internet]. 2013;37(8):1583–9. Available from: https://doi.org/10.1007/s00264-013-1972-830. Trojian TH, Concoff AL, Joy SM, Hatzenbuehler JR, Saulsberry WJ, Coleman CI. AMSSM Scientific Statement Concerning Viscosupplementation Injections for Knee  Osteoarthritis: Importance for Individual Patient Outcomes. Clin J Sport Med  Off J Can Acad  Sport Med. 2016 Jan;26(1):1–11. 31. Ammar TY, Pereira TAP, Mistura SLL, Kuhn A, Saggin JI, Lopes OV. Viscosupplementation for treating knee osteoarthrosis: review of the literature. Rev Bras Ortop. 2015;50:489–94. 32. Salini V, Pantalone A, Vanni D, Abate M. Viscosupplementation in Athletes. In: Arthroscopy and Sport Injuries. Springer; 2016. p. 433–7. 33. Erdem Y, Gul D, Akpancar S. Comparison of Intraarticular Injections of Hyaluronic Acid versus Dextrose Applied with Periarticular Prolotherapy in the Treatment of Recreational Athletes with Knee Osteoarthritis. Turkish J Sport Med. 2020;55(1):6–13. 34. Kalkhoven J, Coutts AJ, Impellizzeri FM. 'Training load error' is not a more accurate term than 'overuse' injury. Br J Sports Med [Internet]. 2020 Aug 1;54(15):934 LP – 935. Available from: http://bjsm.bmj.com/content/54/15/934.abstract35. Odole AC, Agbomeji OT, Onyeso OKK, Ojo JO, Odunaiya NA. Perspectives of Nigerian Athletes About Physiotherapy Services in Sports Injury Management: Implications for Rehabilitation. J Sport Rehabil. 2021;1(aop):1–8. 36. Hendrika W, Reswari A. The effect of physiotherapy on pain improvement in patients with early knee osteoarthritis at RSU UKI. Int J Med Heal Res. 2021;7(6):52–9. 37. MacKay C, Hawker GA, Jaglal SB. How Do Physical Therapists Approach Management of People With Early Knee Osteoarthritis? A Qualitative Study. Phys Ther [Internet]. 2019;100(2):295–306. Available from: https://doi.org/10.1093/ptj/pzz16438. Hrazdira L, Pilecki Z, Pilecki G. Ultrasound guided application of HA in the soft tissues of the locomotor system in athletes–First experience. Krátká sdělení Botek, M, Stejskal, P, Klimešová, I Vliv normobarické hypoxie na Akt Auton Nerv systému Pilot Stud 191 Drbošalová, V, Stejskal, P, Klugar, M, Krejčíř, V, Hynková, O Charakt klientů komerčn. :205. 39. Ricci M, Micheloni GM, Berti M, Perusi F, Sambugaro E, Vecchini E, et al. Clinical comparison of oral administration and viscosupplementation of hyaluronic acid (HA) in early knee osteoarthritis. Musculoskelet Surg. 2017;101(1):45–9. 40. Wang C-T, Lin Y-T, Chiang B-L, Lin Y-H, Hou S-M. High molecular weight hyaluronic acid down-regulates the gene expression of osteoarthritis-associated cytokines and enzymes in fibroblast-like synoviocytes from patients with early osteoarthritis. Osteoarthr Cartil. 2006;14(12):1237–47. 41. Irrgang JJ, Anderson AF. Development and validation of health-related quality of life measures for the knee. Clin Orthop Relat Res. 2002;402:95–109. 42. Roos EM, Roos HP, Lohmander LS, Ekdahl C, Beynnon BD. Knee Injury and Osteoarthritis Outcome Score (KOOS)—development of a self-administered outcome measure. J Orthop Sport Phys Ther. 1998;28(2):88–96. 43. Perruccio A V, Canizares M, Hawker GA, Roos EM, Maillefert JF, Lohmander SL. Comparative Validity and Responsiveness of the HOOS-PS and KOOS-PS to the WOMAC Physical Function Subscale in Total Joint Replacement for Osteoarthritis. 44. Salavati M, Mazaheri M, Negahban H, Sohani SM, Ebrahimian MR, Ebrahimi I, et al. Validation of a Persian-version of Knee injury and Osteoarthritis Outcome Score (KOOS) in Iranians with knee injuries. Osteoarthr Cartil. 2008;16(10):1178–82. 45. Higgins LD, Taylor MK, Park D, Ghodadra N, Marchant M, Pietrobon R, et al. Reliability and validity of the International Knee Documentation Committee (IKDC) subjective knee form. Jt Bone Spine. 2007;74(6):594–9. 46. Kujala UM, Kaprio J, Sarno S. Osteoarthritis of weight bearing joints of lower limbs in former elite male athletes. Bmj. 1994;308(6923):231–4. 47. Salini V, Pantalone A, Vanni D. Arthroscopy and Sport Injuries. Arthrosc Sport Inj. 2016;433–7. 48. Broglio K. Randomization in clinical trials: Permuted blocks and stratification. JAMA - J Am Med Assoc. 2018;319(21):2223–4. 49. Emanuel EJ, Wendler D, Grady C. What makes clinical research ethical? JAMA. 2000 May;283(20):2701–11. )

[16.	Altman R, Asch E, Bloch D, Bole G, Borenstein D, Brandt K, et al. Development of criteria for the classification and reporting of osteoarthritis: classification of osteoarthritis of the knee. Arthritis Rheum Off J Am Coll Rheumatol. 1986;29(8):1039–49.](1. Patel DR, Villalobos A. Evaluation and management of knee pain in young athletes: overuse injuries of the knee. Transl Pediatr [Internet]. 2017 Jul;6(3):190–8. Available from: https://pubmed.ncbi.nlm.nih.gov/287950102. Nicolini AP, de Carvalho RT, Matsuda MM, Filho JS, Cohen M. Common injuries in athletes' knee: Experience of a specialized center. Acta Ortop Bras. 2014;22(3):127–31. 3. Tiu T, Craig Van Dien MD. Knee overuse disorders. 4. Fuller CW, Ekstrand J, Junge A, Andersen TE, Bahr R, Dvorak J, et al. Consensus statement on injury definitions and data collection procedures in studies  of football (soccer) injuries. Br J Sports Med. 2006 Mar;40(3):193–201. 5. Majewski M, Susanne H, Klaus S. Epidemiology of athletic knee injuries: A 10-year study. Knee. 2006 Jun;13(3):184–8. 6. Wilder RP, Sethi S. Overuse injuries: tendinopathies, stress fractures, compartment syndrome, and shin  splints. Clin Sports Med. 2004 Jan;23(1):55–81, vi. 7. Aicale R, Tarantino D, Maffulli N. Overuse injuries in sport: a comprehensive overview. J Orthop Surg Res. 2018 Dec;13(1):309. 8. Yang J, Tibbetts AS, Covassin T, Cheng G, Nayar S, Heiden E. Epidemiology of overuse and acute injuries among competitive collegiate athletes. J Athl Train. 2012;47(2):198–204. 9. Bernetti A, Mangone M, Paoloni M, Di Sante L, Murgia M, Santilli V. Corticosteroid and hyaluronic acid injection therapy in tennis elbow (lateral epicondylalgia). Med Sport. 2014;67(2):289–95. 10. Iolascon G, Gimigliano F, Moretti A, De Sire A, Migliore A, Brandi ML, et al. Early osteoarthritis: How to define, diagnose, and manage. A systematic review. Eur Geriatr Med. 2017;8(5–6):383–96. 11. Drawer S, Fuller CW. Propensity for osteoarthritis and lower limb joint pain in retired professional soccer players. Br J Sports Med. 2001;35(6):402–8. 12. Vannini F, Spalding T, Andriolo L, Berruto M, Denti M, Espregueira-Mendes J, et al. Sport and early osteoarthritis: the role of sport in aetiology, progression and treatment of knee osteoarthritis. Knee Surgery, Sport Traumatol Arthrosc. 2016;24(6):1786–96. 13. Fröhlich S, Pazeller S, Cherati AS, Müller E, Frey WO, Spörri J. Overuse injuries in the knee, back and hip of top elite female alpine skiers during  the off-season preparation period: prevalence, severity and their association with traumatic preinjuries and training load. BMJ open Sport Exerc Med. 2020;6(1):e000892. 14. Rincon GA, Vyas D, Zhou J, Fu FH, Oiestad BE, Holm I, et al. \“ Knee function and prevalence of knee osteoarthritis after anterior cruciate ligament reconstruction: a prospective study with 10 to 15 years of follow-up\”. Letter to the editor. Am J Sports Med. 2011;39(4):NP3-author. 15. Kellgren JH, Lawrence J. Radiological assessment of osteo-arthrosis. Ann Rheum Dis. 1957;16(4):494. 16. Altman R, Asch E, Bloch D, Bole G, Borenstein D, Brandt K, et al. Development of criteria for the classification and reporting of osteoarthritis: classification of osteoarthritis of the knee. Arthritis Rheum Off J Am Coll Rheumatol. 1986;29(8):1039–49. 17. Luyten FP, Denti M, Filardo G, Kon E, Engebretsen L. Definition and classification of early osteoarthritis of the knee. Knee Surgery, Sport Traumatol Arthrosc. 2012;20(3):401–6. 18. Hoemann C, Kandel R, Roberts S, Saris DBF, Creemers L, Mainil-Varlet P, et al. International Cartilage Repair Society (ICRS) Recommended Guidelines for  Histological Endpoints for Cartilage Repair Studies in Animal Models and Clinical Trials. Cartilage. 2011 Apr;2(2):153–72. 19. Hunter DJ, Lo GH, Gale D, Grainger AJ, Guermazi A, Conaghan PG. The reliability of a new scoring system for knee osteoarthritis MRI and the validity of bone marrow lesion assessment: BLOKS (Boston–Leeds Osteoarthritis Knee Score). Ann Rheum Dis. 2008;67(2):206–11. 20. Peterfy CG, Guermazi A, Zaim S, Tirman PFJ, Miaux Y, White D, et al. Whole-organ magnetic resonance imaging score (WORMS) of the knee in osteoarthritis. Osteoarthr Cartil. 2004;12(3):177–90. 21. Demange MK, Sisto M, Rodeo S. Future trends for unicompartmental arthritis of the knee: injectables & stem cells. Clin Sports Med. 2014;33(1):161–74. 22. Leslie M. Knee osteoarthritis management therapies. Pain Manag Nurs. 2000;1(2):51–7. 23. Paoloni M, Bernetti A, Belelli A, Brignoli O, Buoso S, Caputi AP, et al. Appropriateness of clinical and organizational criteria for intra-articular injection therapies in osteoarthritis: A Delphi method consensus initiative among experts in Italy. Ann Ist Super Sanita. 2015;51:131–8. 24. Sandmark H, Hogstedt C, Lewold S, Vingård E. Osteoarthrosis of the knee in men and women in association with overweight, smoking, and hormone therapy. Ann Rheum Dis. 1999;58(3):151–5. 25. Christensen BV, Iuhl IU, Vilbek H, Bülow H, Dreijer NC, Rasmussen HF. Acupuncture treatment of severe knee osteoarthrosis. A long‐term study. Acta Anaesthesiol Scand. 1992;36(6):519–25. 26. de Sire A, Agostini F, Lippi L, Mangone M, Marchese S, Cisari C, et al. Oxygen-Ozone Therapy in the Rehabilitation Field: State of the Art on Mechanisms of  Action, Safety and Effectiveness in Patients with Musculoskeletal Disorders. Biomolecules. 2021 Feb;11(3). 27. Fransen M, McCONNELL S. Land-based exercise for osteoarthritis of the knee: a metaanalysis of randomized controlled trials. J Rheumatol. 2009;36(6):1109–17. 28. Hiemstra LA, Kerslake S, Irving C. Anterior knee pain in the athlete. Clin Sports Med. 2014;33(3):437–59. 29. Filardo G, Kon E, Di Matteo B, Pelotti P, Di Martino A, Marcacci M. Platelet-rich plasma for the treatment of patellar tendinopathy: clinical and imaging findings at medium-term follow-up. Int Orthop [Internet]. 2013;37(8):1583–9. Available from: https://doi.org/10.1007/s00264-013-1972-830. Trojian TH, Concoff AL, Joy SM, Hatzenbuehler JR, Saulsberry WJ, Coleman CI. AMSSM Scientific Statement Concerning Viscosupplementation Injections for Knee  Osteoarthritis: Importance for Individual Patient Outcomes. Clin J Sport Med  Off J Can Acad  Sport Med. 2016 Jan;26(1):1–11. 31. Ammar TY, Pereira TAP, Mistura SLL, Kuhn A, Saggin JI, Lopes OV. Viscosupplementation for treating knee osteoarthrosis: review of the literature. Rev Bras Ortop. 2015;50:489–94. 32. Salini V, Pantalone A, Vanni D, Abate M. Viscosupplementation in Athletes. In: Arthroscopy and Sport Injuries. Springer; 2016. p. 433–7. 33. Erdem Y, Gul D, Akpancar S. Comparison of Intraarticular Injections of Hyaluronic Acid versus Dextrose Applied with Periarticular Prolotherapy in the Treatment of Recreational Athletes with Knee Osteoarthritis. Turkish J Sport Med. 2020;55(1):6–13. 34. Kalkhoven J, Coutts AJ, Impellizzeri FM. 'Training load error' is not a more accurate term than 'overuse' injury. Br J Sports Med [Internet]. 2020 Aug 1;54(15):934 LP – 935. Available from: http://bjsm.bmj.com/content/54/15/934.abstract35. Odole AC, Agbomeji OT, Onyeso OKK, Ojo JO, Odunaiya NA. Perspectives of Nigerian Athletes About Physiotherapy Services in Sports Injury Management: Implications for Rehabilitation. J Sport Rehabil. 2021;1(aop):1–8. 36. Hendrika W, Reswari A. The effect of physiotherapy on pain improvement in patients with early knee osteoarthritis at RSU UKI. Int J Med Heal Res. 2021;7(6):52–9. 37. MacKay C, Hawker GA, Jaglal SB. How Do Physical Therapists Approach Management of People With Early Knee Osteoarthritis? A Qualitative Study. Phys Ther [Internet]. 2019;100(2):295–306. Available from: https://doi.org/10.1093/ptj/pzz16438. Hrazdira L, Pilecki Z, Pilecki G. Ultrasound guided application of HA in the soft tissues of the locomotor system in athletes–First experience. Krátká sdělení Botek, M, Stejskal, P, Klimešová, I Vliv normobarické hypoxie na Akt Auton Nerv systému Pilot Stud 191 Drbošalová, V, Stejskal, P, Klugar, M, Krejčíř, V, Hynková, O Charakt klientů komerčn. :205. 39. Ricci M, Micheloni GM, Berti M, Perusi F, Sambugaro E, Vecchini E, et al. Clinical comparison of oral administration and viscosupplementation of hyaluronic acid (HA) in early knee osteoarthritis. Musculoskelet Surg. 2017;101(1):45–9. 40. Wang C-T, Lin Y-T, Chiang B-L, Lin Y-H, Hou S-M. High molecular weight hyaluronic acid down-regulates the gene expression of osteoarthritis-associated cytokines and enzymes in fibroblast-like synoviocytes from patients with early osteoarthritis. Osteoarthr Cartil. 2006;14(12):1237–47. 41. Irrgang JJ, Anderson AF. Development and validation of health-related quality of life measures for the knee. Clin Orthop Relat Res. 2002;402:95–109. 42. Roos EM, Roos HP, Lohmander LS, Ekdahl C, Beynnon BD. Knee Injury and Osteoarthritis Outcome Score (KOOS)—development of a self-administered outcome measure. J Orthop Sport Phys Ther. 1998;28(2):88–96. 43. Perruccio A V, Canizares M, Hawker GA, Roos EM, Maillefert JF, Lohmander SL. Comparative Validity and Responsiveness of the HOOS-PS and KOOS-PS to the WOMAC Physical Function Subscale in Total Joint Replacement for Osteoarthritis. 44. Salavati M, Mazaheri M, Negahban H, Sohani SM, Ebrahimian MR, Ebrahimi I, et al. Validation of a Persian-version of Knee injury and Osteoarthritis Outcome Score (KOOS) in Iranians with knee injuries. Osteoarthr Cartil. 2008;16(10):1178–82. 45. Higgins LD, Taylor MK, Park D, Ghodadra N, Marchant M, Pietrobon R, et al. Reliability and validity of the International Knee Documentation Committee (IKDC) subjective knee form. Jt Bone Spine. 2007;74(6):594–9. 46. Kujala UM, Kaprio J, Sarno S. Osteoarthritis of weight bearing joints of lower limbs in former elite male athletes. Bmj. 1994;308(6923):231–4. 47. Salini V, Pantalone A, Vanni D. Arthroscopy and Sport Injuries. Arthrosc Sport Inj. 2016;433–7. 48. Broglio K. Randomization in clinical trials: Permuted blocks and stratification. JAMA - J Am Med Assoc. 2018;319(21):2223–4. 49. Emanuel EJ, Wendler D, Grady C. What makes clinical research ethical? JAMA. 2000 May;283(20):2701–11. )

[17.	Luyten FP, Denti M, Filardo G, Kon E, Engebretsen L. Definition and classification of early osteoarthritis of the knee. Knee Surgery, Sport Traumatol Arthrosc. 2012;20(3):401–6.](1. Patel DR, Villalobos A. Evaluation and management of knee pain in young athletes: overuse injuries of the knee. Transl Pediatr [Internet]. 2017 Jul;6(3):190–8. Available from: https://pubmed.ncbi.nlm.nih.gov/287950102. Nicolini AP, de Carvalho RT, Matsuda MM, Filho JS, Cohen M. Common injuries in athletes' knee: Experience of a specialized center. Acta Ortop Bras. 2014;22(3):127–31. 3. Tiu T, Craig Van Dien MD. Knee overuse disorders. 4. Fuller CW, Ekstrand J, Junge A, Andersen TE, Bahr R, Dvorak J, et al. Consensus statement on injury definitions and data collection procedures in studies  of football (soccer) injuries. Br J Sports Med. 2006 Mar;40(3):193–201. 5. Majewski M, Susanne H, Klaus S. Epidemiology of athletic knee injuries: A 10-year study. Knee. 2006 Jun;13(3):184–8. 6. Wilder RP, Sethi S. Overuse injuries: tendinopathies, stress fractures, compartment syndrome, and shin  splints. Clin Sports Med. 2004 Jan;23(1):55–81, vi. 7. Aicale R, Tarantino D, Maffulli N. Overuse injuries in sport: a comprehensive overview. J Orthop Surg Res. 2018 Dec;13(1):309. 8. Yang J, Tibbetts AS, Covassin T, Cheng G, Nayar S, Heiden E. Epidemiology of overuse and acute injuries among competitive collegiate athletes. J Athl Train. 2012;47(2):198–204. 9. Bernetti A, Mangone M, Paoloni M, Di Sante L, Murgia M, Santilli V. Corticosteroid and hyaluronic acid injection therapy in tennis elbow (lateral epicondylalgia). Med Sport. 2014;67(2):289–95. 10. Iolascon G, Gimigliano F, Moretti A, De Sire A, Migliore A, Brandi ML, et al. Early osteoarthritis: How to define, diagnose, and manage. A systematic review. Eur Geriatr Med. 2017;8(5–6):383–96. 11. Drawer S, Fuller CW. Propensity for osteoarthritis and lower limb joint pain in retired professional soccer players. Br J Sports Med. 2001;35(6):402–8. 12. Vannini F, Spalding T, Andriolo L, Berruto M, Denti M, Espregueira-Mendes J, et al. Sport and early osteoarthritis: the role of sport in aetiology, progression and treatment of knee osteoarthritis. Knee Surgery, Sport Traumatol Arthrosc. 2016;24(6):1786–96. 13. Fröhlich S, Pazeller S, Cherati AS, Müller E, Frey WO, Spörri J. Overuse injuries in the knee, back and hip of top elite female alpine skiers during  the off-season preparation period: prevalence, severity and their association with traumatic preinjuries and training load. BMJ open Sport Exerc Med. 2020;6(1):e000892. 14. Rincon GA, Vyas D, Zhou J, Fu FH, Oiestad BE, Holm I, et al. \“ Knee function and prevalence of knee osteoarthritis after anterior cruciate ligament reconstruction: a prospective study with 10 to 15 years of follow-up\”. Letter to the editor. Am J Sports Med. 2011;39(4):NP3-author. 15. Kellgren JH, Lawrence J. Radiological assessment of osteo-arthrosis. Ann Rheum Dis. 1957;16(4):494. 16. Altman R, Asch E, Bloch D, Bole G, Borenstein D, Brandt K, et al. Development of criteria for the classification and reporting of osteoarthritis: classification of osteoarthritis of the knee. Arthritis Rheum Off J Am Coll Rheumatol. 1986;29(8):1039–49. 17. Luyten FP, Denti M, Filardo G, Kon E, Engebretsen L. Definition and classification of early osteoarthritis of the knee. Knee Surgery, Sport Traumatol Arthrosc. 2012;20(3):401–6. 18. Hoemann C, Kandel R, Roberts S, Saris DBF, Creemers L, Mainil-Varlet P, et al. International Cartilage Repair Society (ICRS) Recommended Guidelines for  Histological Endpoints for Cartilage Repair Studies in Animal Models and Clinical Trials. Cartilage. 2011 Apr;2(2):153–72. 19. Hunter DJ, Lo GH, Gale D, Grainger AJ, Guermazi A, Conaghan PG. The reliability of a new scoring system for knee osteoarthritis MRI and the validity of bone marrow lesion assessment: BLOKS (Boston–Leeds Osteoarthritis Knee Score). Ann Rheum Dis. 2008;67(2):206–11. 20. Peterfy CG, Guermazi A, Zaim S, Tirman PFJ, Miaux Y, White D, et al. Whole-organ magnetic resonance imaging score (WORMS) of the knee in osteoarthritis. Osteoarthr Cartil. 2004;12(3):177–90. 21. Demange MK, Sisto M, Rodeo S. Future trends for unicompartmental arthritis of the knee: injectables & stem cells. Clin Sports Med. 2014;33(1):161–74. 22. Leslie M. Knee osteoarthritis management therapies. Pain Manag Nurs. 2000;1(2):51–7. 23. Paoloni M, Bernetti A, Belelli A, Brignoli O, Buoso S, Caputi AP, et al. Appropriateness of clinical and organizational criteria for intra-articular injection therapies in osteoarthritis: A Delphi method consensus initiative among experts in Italy. Ann Ist Super Sanita. 2015;51:131–8. 24. Sandmark H, Hogstedt C, Lewold S, Vingård E. Osteoarthrosis of the knee in men and women in association with overweight, smoking, and hormone therapy. Ann Rheum Dis. 1999;58(3):151–5. 25. Christensen BV, Iuhl IU, Vilbek H, Bülow H, Dreijer NC, Rasmussen HF. Acupuncture treatment of severe knee osteoarthrosis. A long‐term study. Acta Anaesthesiol Scand. 1992;36(6):519–25. 26. de Sire A, Agostini F, Lippi L, Mangone M, Marchese S, Cisari C, et al. Oxygen-Ozone Therapy in the Rehabilitation Field: State of the Art on Mechanisms of  Action, Safety and Effectiveness in Patients with Musculoskeletal Disorders. Biomolecules. 2021 Feb;11(3). 27. Fransen M, McCONNELL S. Land-based exercise for osteoarthritis of the knee: a metaanalysis of randomized controlled trials. J Rheumatol. 2009;36(6):1109–17. 28. Hiemstra LA, Kerslake S, Irving C. Anterior knee pain in the athlete. Clin Sports Med. 2014;33(3):437–59. 29. Filardo G, Kon E, Di Matteo B, Pelotti P, Di Martino A, Marcacci M. Platelet-rich plasma for the treatment of patellar tendinopathy: clinical and imaging findings at medium-term follow-up. Int Orthop [Internet]. 2013;37(8):1583–9. Available from: https://doi.org/10.1007/s00264-013-1972-830. Trojian TH, Concoff AL, Joy SM, Hatzenbuehler JR, Saulsberry WJ, Coleman CI. AMSSM Scientific Statement Concerning Viscosupplementation Injections for Knee  Osteoarthritis: Importance for Individual Patient Outcomes. Clin J Sport Med  Off J Can Acad  Sport Med. 2016 Jan;26(1):1–11. 31. Ammar TY, Pereira TAP, Mistura SLL, Kuhn A, Saggin JI, Lopes OV. Viscosupplementation for treating knee osteoarthrosis: review of the literature. Rev Bras Ortop. 2015;50:489–94. 32. Salini V, Pantalone A, Vanni D, Abate M. Viscosupplementation in Athletes. In: Arthroscopy and Sport Injuries. Springer; 2016. p. 433–7. 33. Erdem Y, Gul D, Akpancar S. Comparison of Intraarticular Injections of Hyaluronic Acid versus Dextrose Applied with Periarticular Prolotherapy in the Treatment of Recreational Athletes with Knee Osteoarthritis. Turkish J Sport Med. 2020;55(1):6–13. 34. Kalkhoven J, Coutts AJ, Impellizzeri FM. 'Training load error' is not a more accurate term than 'overuse' injury. Br J Sports Med [Internet]. 2020 Aug 1;54(15):934 LP – 935. Available from: http://bjsm.bmj.com/content/54/15/934.abstract35. Odole AC, Agbomeji OT, Onyeso OKK, Ojo JO, Odunaiya NA. Perspectives of Nigerian Athletes About Physiotherapy Services in Sports Injury Management: Implications for Rehabilitation. J Sport Rehabil. 2021;1(aop):1–8. 36. Hendrika W, Reswari A. The effect of physiotherapy on pain improvement in patients with early knee osteoarthritis at RSU UKI. Int J Med Heal Res. 2021;7(6):52–9. 37. MacKay C, Hawker GA, Jaglal SB. How Do Physical Therapists Approach Management of People With Early Knee Osteoarthritis? A Qualitative Study. Phys Ther [Internet]. 2019;100(2):295–306. Available from: https://doi.org/10.1093/ptj/pzz16438. Hrazdira L, Pilecki Z, Pilecki G. Ultrasound guided application of HA in the soft tissues of the locomotor system in athletes–First experience. Krátká sdělení Botek, M, Stejskal, P, Klimešová, I Vliv normobarické hypoxie na Akt Auton Nerv systému Pilot Stud 191 Drbošalová, V, Stejskal, P, Klugar, M, Krejčíř, V, Hynková, O Charakt klientů komerčn. :205. 39. Ricci M, Micheloni GM, Berti M, Perusi F, Sambugaro E, Vecchini E, et al. Clinical comparison of oral administration and viscosupplementation of hyaluronic acid (HA) in early knee osteoarthritis. Musculoskelet Surg. 2017;101(1):45–9. 40. Wang C-T, Lin Y-T, Chiang B-L, Lin Y-H, Hou S-M. High molecular weight hyaluronic acid down-regulates the gene expression of osteoarthritis-associated cytokines and enzymes in fibroblast-like synoviocytes from patients with early osteoarthritis. Osteoarthr Cartil. 2006;14(12):1237–47. 41. Irrgang JJ, Anderson AF. Development and validation of health-related quality of life measures for the knee. Clin Orthop Relat Res. 2002;402:95–109. 42. Roos EM, Roos HP, Lohmander LS, Ekdahl C, Beynnon BD. Knee Injury and Osteoarthritis Outcome Score (KOOS)—development of a self-administered outcome measure. J Orthop Sport Phys Ther. 1998;28(2):88–96. 43. Perruccio A V, Canizares M, Hawker GA, Roos EM, Maillefert JF, Lohmander SL. Comparative Validity and Responsiveness of the HOOS-PS and KOOS-PS to the WOMAC Physical Function Subscale in Total Joint Replacement for Osteoarthritis. 44. Salavati M, Mazaheri M, Negahban H, Sohani SM, Ebrahimian MR, Ebrahimi I, et al. Validation of a Persian-version of Knee injury and Osteoarthritis Outcome Score (KOOS) in Iranians with knee injuries. Osteoarthr Cartil. 2008;16(10):1178–82. 45. Higgins LD, Taylor MK, Park D, Ghodadra N, Marchant M, Pietrobon R, et al. Reliability and validity of the International Knee Documentation Committee (IKDC) subjective knee form. Jt Bone Spine. 2007;74(6):594–9. 46. Kujala UM, Kaprio J, Sarno S. Osteoarthritis of weight bearing joints of lower limbs in former elite male athletes. Bmj. 1994;308(6923):231–4. 47. Salini V, Pantalone A, Vanni D. Arthroscopy and Sport Injuries. Arthrosc Sport Inj. 2016;433–7. 48. Broglio K. Randomization in clinical trials: Permuted blocks and stratification. JAMA - J Am Med Assoc. 2018;319(21):2223–4. 49. Emanuel EJ, Wendler D, Grady C. What makes clinical research ethical? JAMA. 2000 May;283(20):2701–11. )

[18.	Hoemann C, Kandel R, Roberts S, Saris DBF, Creemers L, Mainil-Varlet P, et al. International Cartilage Repair Society (ICRS) Recommended Guidelines for Histological Endpoints for Cartilage Repair Studies in Animal Models and Clinical Trials. Cartilage. 2011 Apr;2(2):153–72.](1. Patel DR, Villalobos A. Evaluation and management of knee pain in young athletes: overuse injuries of the knee. Transl Pediatr [Internet]. 2017 Jul;6(3):190–8. Available from: https://pubmed.ncbi.nlm.nih.gov/287950102. Nicolini AP, de Carvalho RT, Matsuda MM, Filho JS, Cohen M. Common injuries in athletes' knee: Experience of a specialized center. Acta Ortop Bras. 2014;22(3):127–31. 3. Tiu T, Craig Van Dien MD. Knee overuse disorders. 4. Fuller CW, Ekstrand J, Junge A, Andersen TE, Bahr R, Dvorak J, et al. Consensus statement on injury definitions and data collection procedures in studies  of football (soccer) injuries. Br J Sports Med. 2006 Mar;40(3):193–201. 5. Majewski M, Susanne H, Klaus S. Epidemiology of athletic knee injuries: A 10-year study. Knee. 2006 Jun;13(3):184–8. 6. Wilder RP, Sethi S. Overuse injuries: tendinopathies, stress fractures, compartment syndrome, and shin  splints. Clin Sports Med. 2004 Jan;23(1):55–81, vi. 7. Aicale R, Tarantino D, Maffulli N. Overuse injuries in sport: a comprehensive overview. J Orthop Surg Res. 2018 Dec;13(1):309. 8. Yang J, Tibbetts AS, Covassin T, Cheng G, Nayar S, Heiden E. Epidemiology of overuse and acute injuries among competitive collegiate athletes. J Athl Train. 2012;47(2):198–204. 9. Bernetti A, Mangone M, Paoloni M, Di Sante L, Murgia M, Santilli V. Corticosteroid and hyaluronic acid injection therapy in tennis elbow (lateral epicondylalgia). Med Sport. 2014;67(2):289–95. 10. Iolascon G, Gimigliano F, Moretti A, De Sire A, Migliore A, Brandi ML, et al. Early osteoarthritis: How to define, diagnose, and manage. A systematic review. Eur Geriatr Med. 2017;8(5–6):383–96. 11. Drawer S, Fuller CW. Propensity for osteoarthritis and lower limb joint pain in retired professional soccer players. Br J Sports Med. 2001;35(6):402–8. 12. Vannini F, Spalding T, Andriolo L, Berruto M, Denti M, Espregueira-Mendes J, et al. Sport and early osteoarthritis: the role of sport in aetiology, progression and treatment of knee osteoarthritis. Knee Surgery, Sport Traumatol Arthrosc. 2016;24(6):1786–96. 13. Fröhlich S, Pazeller S, Cherati AS, Müller E, Frey WO, Spörri J. Overuse injuries in the knee, back and hip of top elite female alpine skiers during  the off-season preparation period: prevalence, severity and their association with traumatic preinjuries and training load. BMJ open Sport Exerc Med. 2020;6(1):e000892. 14. Rincon GA, Vyas D, Zhou J, Fu FH, Oiestad BE, Holm I, et al. \“ Knee function and prevalence of knee osteoarthritis after anterior cruciate ligament reconstruction: a prospective study with 10 to 15 years of follow-up\”. Letter to the editor. Am J Sports Med. 2011;39(4):NP3-author. 15. Kellgren JH, Lawrence J. Radiological assessment of osteo-arthrosis. Ann Rheum Dis. 1957;16(4):494. 16. Altman R, Asch E, Bloch D, Bole G, Borenstein D, Brandt K, et al. Development of criteria for the classification and reporting of osteoarthritis: classification of osteoarthritis of the knee. Arthritis Rheum Off J Am Coll Rheumatol. 1986;29(8):1039–49. 17. Luyten FP, Denti M, Filardo G, Kon E, Engebretsen L. Definition and classification of early osteoarthritis of the knee. Knee Surgery, Sport Traumatol Arthrosc. 2012;20(3):401–6. 18. Hoemann C, Kandel R, Roberts S, Saris DBF, Creemers L, Mainil-Varlet P, et al. International Cartilage Repair Society (ICRS) Recommended Guidelines for  Histological Endpoints for Cartilage Repair Studies in Animal Models and Clinical Trials. Cartilage. 2011 Apr;2(2):153–72. 19. Hunter DJ, Lo GH, Gale D, Grainger AJ, Guermazi A, Conaghan PG. The reliability of a new scoring system for knee osteoarthritis MRI and the validity of bone marrow lesion assessment: BLOKS (Boston–Leeds Osteoarthritis Knee Score). Ann Rheum Dis. 2008;67(2):206–11. 20. Peterfy CG, Guermazi A, Zaim S, Tirman PFJ, Miaux Y, White D, et al. Whole-organ magnetic resonance imaging score (WORMS) of the knee in osteoarthritis. Osteoarthr Cartil. 2004;12(3):177–90. 21. Demange MK, Sisto M, Rodeo S. Future trends for unicompartmental arthritis of the knee: injectables & stem cells. Clin Sports Med. 2014;33(1):161–74. 22. Leslie M. Knee osteoarthritis management therapies. Pain Manag Nurs. 2000;1(2):51–7. 23. Paoloni M, Bernetti A, Belelli A, Brignoli O, Buoso S, Caputi AP, et al. Appropriateness of clinical and organizational criteria for intra-articular injection therapies in osteoarthritis: A Delphi method consensus initiative among experts in Italy. Ann Ist Super Sanita. 2015;51:131–8. 24. Sandmark H, Hogstedt C, Lewold S, Vingård E. Osteoarthrosis of the knee in men and women in association with overweight, smoking, and hormone therapy. Ann Rheum Dis. 1999;58(3):151–5. 25. Christensen BV, Iuhl IU, Vilbek H, Bülow H, Dreijer NC, Rasmussen HF. Acupuncture treatment of severe knee osteoarthrosis. A long‐term study. Acta Anaesthesiol Scand. 1992;36(6):519–25. 26. de Sire A, Agostini F, Lippi L, Mangone M, Marchese S, Cisari C, et al. Oxygen-Ozone Therapy in the Rehabilitation Field: State of the Art on Mechanisms of  Action, Safety and Effectiveness in Patients with Musculoskeletal Disorders. Biomolecules. 2021 Feb;11(3). 27. Fransen M, McCONNELL S. Land-based exercise for osteoarthritis of the knee: a metaanalysis of randomized controlled trials. J Rheumatol. 2009;36(6):1109–17. 28. Hiemstra LA, Kerslake S, Irving C. Anterior knee pain in the athlete. Clin Sports Med. 2014;33(3):437–59. 29. Filardo G, Kon E, Di Matteo B, Pelotti P, Di Martino A, Marcacci M. Platelet-rich plasma for the treatment of patellar tendinopathy: clinical and imaging findings at medium-term follow-up. Int Orthop [Internet]. 2013;37(8):1583–9. Available from: https://doi.org/10.1007/s00264-013-1972-830. Trojian TH, Concoff AL, Joy SM, Hatzenbuehler JR, Saulsberry WJ, Coleman CI. AMSSM Scientific Statement Concerning Viscosupplementation Injections for Knee  Osteoarthritis: Importance for Individual Patient Outcomes. Clin J Sport Med  Off J Can Acad  Sport Med. 2016 Jan;26(1):1–11. 31. Ammar TY, Pereira TAP, Mistura SLL, Kuhn A, Saggin JI, Lopes OV. Viscosupplementation for treating knee osteoarthrosis: review of the literature. Rev Bras Ortop. 2015;50:489–94. 32. Salini V, Pantalone A, Vanni D, Abate M. Viscosupplementation in Athletes. In: Arthroscopy and Sport Injuries. Springer; 2016. p. 433–7. 33. Erdem Y, Gul D, Akpancar S. Comparison of Intraarticular Injections of Hyaluronic Acid versus Dextrose Applied with Periarticular Prolotherapy in the Treatment of Recreational Athletes with Knee Osteoarthritis. Turkish J Sport Med. 2020;55(1):6–13. 34. Kalkhoven J, Coutts AJ, Impellizzeri FM. 'Training load error' is not a more accurate term than 'overuse' injury. Br J Sports Med [Internet]. 2020 Aug 1;54(15):934 LP – 935. Available from: http://bjsm.bmj.com/content/54/15/934.abstract35. Odole AC, Agbomeji OT, Onyeso OKK, Ojo JO, Odunaiya NA. Perspectives of Nigerian Athletes About Physiotherapy Services in Sports Injury Management: Implications for Rehabilitation. J Sport Rehabil. 2021;1(aop):1–8. 36. Hendrika W, Reswari A. The effect of physiotherapy on pain improvement in patients with early knee osteoarthritis at RSU UKI. Int J Med Heal Res. 2021;7(6):52–9. 37. MacKay C, Hawker GA, Jaglal SB. How Do Physical Therapists Approach Management of People With Early Knee Osteoarthritis? A Qualitative Study. Phys Ther [Internet]. 2019;100(2):295–306. Available from: https://doi.org/10.1093/ptj/pzz16438. Hrazdira L, Pilecki Z, Pilecki G. Ultrasound guided application of HA in the soft tissues of the locomotor system in athletes–First experience. Krátká sdělení Botek, M, Stejskal, P, Klimešová, I Vliv normobarické hypoxie na Akt Auton Nerv systému Pilot Stud 191 Drbošalová, V, Stejskal, P, Klugar, M, Krejčíř, V, Hynková, O Charakt klientů komerčn. :205. 39. Ricci M, Micheloni GM, Berti M, Perusi F, Sambugaro E, Vecchini E, et al. Clinical comparison of oral administration and viscosupplementation of hyaluronic acid (HA) in early knee osteoarthritis. Musculoskelet Surg. 2017;101(1):45–9. 40. Wang C-T, Lin Y-T, Chiang B-L, Lin Y-H, Hou S-M. High molecular weight hyaluronic acid down-regulates the gene expression of osteoarthritis-associated cytokines and enzymes in fibroblast-like synoviocytes from patients with early osteoarthritis. Osteoarthr Cartil. 2006;14(12):1237–47. 41. Irrgang JJ, Anderson AF. Development and validation of health-related quality of life measures for the knee. Clin Orthop Relat Res. 2002;402:95–109. 42. Roos EM, Roos HP, Lohmander LS, Ekdahl C, Beynnon BD. Knee Injury and Osteoarthritis Outcome Score (KOOS)—development of a self-administered outcome measure. J Orthop Sport Phys Ther. 1998;28(2):88–96. 43. Perruccio A V, Canizares M, Hawker GA, Roos EM, Maillefert JF, Lohmander SL. Comparative Validity and Responsiveness of the HOOS-PS and KOOS-PS to the WOMAC Physical Function Subscale in Total Joint Replacement for Osteoarthritis. 44. Salavati M, Mazaheri M, Negahban H, Sohani SM, Ebrahimian MR, Ebrahimi I, et al. Validation of a Persian-version of Knee injury and Osteoarthritis Outcome Score (KOOS) in Iranians with knee injuries. Osteoarthr Cartil. 2008;16(10):1178–82. 45. Higgins LD, Taylor MK, Park D, Ghodadra N, Marchant M, Pietrobon R, et al. Reliability and validity of the International Knee Documentation Committee (IKDC) subjective knee form. Jt Bone Spine. 2007;74(6):594–9. 46. Kujala UM, Kaprio J, Sarno S. Osteoarthritis of weight bearing joints of lower limbs in former elite male athletes. Bmj. 1994;308(6923):231–4. 47. Salini V, Pantalone A, Vanni D. Arthroscopy and Sport Injuries. Arthrosc Sport Inj. 2016;433–7. 48. Broglio K. Randomization in clinical trials: Permuted blocks and stratification. JAMA - J Am Med Assoc. 2018;319(21):2223–4. 49. Emanuel EJ, Wendler D, Grady C. What makes clinical research ethical? JAMA. 2000 May;283(20):2701–11. )

[19.	Hunter DJ, Lo GH, Gale D, Grainger AJ, Guermazi A, Conaghan PG. The reliability of a new scoring system for knee osteoarthritis MRI and the validity of bone marrow lesion assessment: BLOKS (Boston–Leeds Osteoarthritis Knee Score). Ann Rheum Dis. 2008;67(2):206–11.](1. Patel DR, Villalobos A. Evaluation and management of knee pain in young athletes: overuse injuries of the knee. Transl Pediatr [Internet]. 2017 Jul;6(3):190–8. Available from: https://pubmed.ncbi.nlm.nih.gov/287950102. Nicolini AP, de Carvalho RT, Matsuda MM, Filho JS, Cohen M. Common injuries in athletes' knee: Experience of a specialized center. Acta Ortop Bras. 2014;22(3):127–31. 3. Tiu T, Craig Van Dien MD. Knee overuse disorders. 4. Fuller CW, Ekstrand J, Junge A, Andersen TE, Bahr R, Dvorak J, et al. Consensus statement on injury definitions and data collection procedures in studies  of football (soccer) injuries. Br J Sports Med. 2006 Mar;40(3):193–201. 5. Majewski M, Susanne H, Klaus S. Epidemiology of athletic knee injuries: A 10-year study. Knee. 2006 Jun;13(3):184–8. 6. Wilder RP, Sethi S. Overuse injuries: tendinopathies, stress fractures, compartment syndrome, and shin  splints. Clin Sports Med. 2004 Jan;23(1):55–81, vi. 7. Aicale R, Tarantino D, Maffulli N. Overuse injuries in sport: a comprehensive overview. J Orthop Surg Res. 2018 Dec;13(1):309. 8. Yang J, Tibbetts AS, Covassin T, Cheng G, Nayar S, Heiden E. Epidemiology of overuse and acute injuries among competitive collegiate athletes. J Athl Train. 2012;47(2):198–204. 9. Bernetti A, Mangone M, Paoloni M, Di Sante L, Murgia M, Santilli V. Corticosteroid and hyaluronic acid injection therapy in tennis elbow (lateral epicondylalgia). Med Sport. 2014;67(2):289–95. 10. Iolascon G, Gimigliano F, Moretti A, De Sire A, Migliore A, Brandi ML, et al. Early osteoarthritis: How to define, diagnose, and manage. A systematic review. Eur Geriatr Med. 2017;8(5–6):383–96. 11. Drawer S, Fuller CW. Propensity for osteoarthritis and lower limb joint pain in retired professional soccer players. Br J Sports Med. 2001;35(6):402–8. 12. Vannini F, Spalding T, Andriolo L, Berruto M, Denti M, Espregueira-Mendes J, et al. Sport and early osteoarthritis: the role of sport in aetiology, progression and treatment of knee osteoarthritis. Knee Surgery, Sport Traumatol Arthrosc. 2016;24(6):1786–96. 13. Fröhlich S, Pazeller S, Cherati AS, Müller E, Frey WO, Spörri J. Overuse injuries in the knee, back and hip of top elite female alpine skiers during  the off-season preparation period: prevalence, severity and their association with traumatic preinjuries and training load. BMJ open Sport Exerc Med. 2020;6(1):e000892. 14. Rincon GA, Vyas D, Zhou J, Fu FH, Oiestad BE, Holm I, et al. \“ Knee function and prevalence of knee osteoarthritis after anterior cruciate ligament reconstruction: a prospective study with 10 to 15 years of follow-up\”. Letter to the editor. Am J Sports Med. 2011;39(4):NP3-author. 15. Kellgren JH, Lawrence J. Radiological assessment of osteo-arthrosis. Ann Rheum Dis. 1957;16(4):494. 16. Altman R, Asch E, Bloch D, Bole G, Borenstein D, Brandt K, et al. Development of criteria for the classification and reporting of osteoarthritis: classification of osteoarthritis of the knee. Arthritis Rheum Off J Am Coll Rheumatol. 1986;29(8):1039–49. 17. Luyten FP, Denti M, Filardo G, Kon E, Engebretsen L. Definition and classification of early osteoarthritis of the knee. Knee Surgery, Sport Traumatol Arthrosc. 2012;20(3):401–6. 18. Hoemann C, Kandel R, Roberts S, Saris DBF, Creemers L, Mainil-Varlet P, et al. International Cartilage Repair Society (ICRS) Recommended Guidelines for  Histological Endpoints for Cartilage Repair Studies in Animal Models and Clinical Trials. Cartilage. 2011 Apr;2(2):153–72. 19. Hunter DJ, Lo GH, Gale D, Grainger AJ, Guermazi A, Conaghan PG. The reliability of a new scoring system for knee osteoarthritis MRI and the validity of bone marrow lesion assessment: BLOKS (Boston–Leeds Osteoarthritis Knee Score). Ann Rheum Dis. 2008;67(2):206–11. 20. Peterfy CG, Guermazi A, Zaim S, Tirman PFJ, Miaux Y, White D, et al. Whole-organ magnetic resonance imaging score (WORMS) of the knee in osteoarthritis. Osteoarthr Cartil. 2004;12(3):177–90. 21. Demange MK, Sisto M, Rodeo S. Future trends for unicompartmental arthritis of the knee: injectables & stem cells. Clin Sports Med. 2014;33(1):161–74. 22. Leslie M. Knee osteoarthritis management therapies. Pain Manag Nurs. 2000;1(2):51–7. 23. Paoloni M, Bernetti A, Belelli A, Brignoli O, Buoso S, Caputi AP, et al. Appropriateness of clinical and organizational criteria for intra-articular injection therapies in osteoarthritis: A Delphi method consensus initiative among experts in Italy. Ann Ist Super Sanita. 2015;51:131–8. 24. Sandmark H, Hogstedt C, Lewold S, Vingård E. Osteoarthrosis of the knee in men and women in association with overweight, smoking, and hormone therapy. Ann Rheum Dis. 1999;58(3):151–5. 25. Christensen BV, Iuhl IU, Vilbek H, Bülow H, Dreijer NC, Rasmussen HF. Acupuncture treatment of severe knee osteoarthrosis. A long‐term study. Acta Anaesthesiol Scand. 1992;36(6):519–25. 26. de Sire A, Agostini F, Lippi L, Mangone M, Marchese S, Cisari C, et al. Oxygen-Ozone Therapy in the Rehabilitation Field: State of the Art on Mechanisms of  Action, Safety and Effectiveness in Patients with Musculoskeletal Disorders. Biomolecules. 2021 Feb;11(3). 27. Fransen M, McCONNELL S. Land-based exercise for osteoarthritis of the knee: a metaanalysis of randomized controlled trials. J Rheumatol. 2009;36(6):1109–17. 28. Hiemstra LA, Kerslake S, Irving C. Anterior knee pain in the athlete. Clin Sports Med. 2014;33(3):437–59. 29. Filardo G, Kon E, Di Matteo B, Pelotti P, Di Martino A, Marcacci M. Platelet-rich plasma for the treatment of patellar tendinopathy: clinical and imaging findings at medium-term follow-up. Int Orthop [Internet]. 2013;37(8):1583–9. Available from: https://doi.org/10.1007/s00264-013-1972-830. Trojian TH, Concoff AL, Joy SM, Hatzenbuehler JR, Saulsberry WJ, Coleman CI. AMSSM Scientific Statement Concerning Viscosupplementation Injections for Knee  Osteoarthritis: Importance for Individual Patient Outcomes. Clin J Sport Med  Off J Can Acad  Sport Med. 2016 Jan;26(1):1–11. 31. Ammar TY, Pereira TAP, Mistura SLL, Kuhn A, Saggin JI, Lopes OV. Viscosupplementation for treating knee osteoarthrosis: review of the literature. Rev Bras Ortop. 2015;50:489–94. 32. Salini V, Pantalone A, Vanni D, Abate M. Viscosupplementation in Athletes. In: Arthroscopy and Sport Injuries. Springer; 2016. p. 433–7. 33. Erdem Y, Gul D, Akpancar S. Comparison of Intraarticular Injections of Hyaluronic Acid versus Dextrose Applied with Periarticular Prolotherapy in the Treatment of Recreational Athletes with Knee Osteoarthritis. Turkish J Sport Med. 2020;55(1):6–13. 34. Kalkhoven J, Coutts AJ, Impellizzeri FM. 'Training load error' is not a more accurate term than 'overuse' injury. Br J Sports Med [Internet]. 2020 Aug 1;54(15):934 LP – 935. Available from: http://bjsm.bmj.com/content/54/15/934.abstract35. Odole AC, Agbomeji OT, Onyeso OKK, Ojo JO, Odunaiya NA. Perspectives of Nigerian Athletes About Physiotherapy Services in Sports Injury Management: Implications for Rehabilitation. J Sport Rehabil. 2021;1(aop):1–8. 36. Hendrika W, Reswari A. The effect of physiotherapy on pain improvement in patients with early knee osteoarthritis at RSU UKI. Int J Med Heal Res. 2021;7(6):52–9. 37. MacKay C, Hawker GA, Jaglal SB. How Do Physical Therapists Approach Management of People With Early Knee Osteoarthritis? A Qualitative Study. Phys Ther [Internet]. 2019;100(2):295–306. Available from: https://doi.org/10.1093/ptj/pzz16438. Hrazdira L, Pilecki Z, Pilecki G. Ultrasound guided application of HA in the soft tissues of the locomotor system in athletes–First experience. Krátká sdělení Botek, M, Stejskal, P, Klimešová, I Vliv normobarické hypoxie na Akt Auton Nerv systému Pilot Stud 191 Drbošalová, V, Stejskal, P, Klugar, M, Krejčíř, V, Hynková, O Charakt klientů komerčn. :205. 39. Ricci M, Micheloni GM, Berti M, Perusi F, Sambugaro E, Vecchini E, et al. Clinical comparison of oral administration and viscosupplementation of hyaluronic acid (HA) in early knee osteoarthritis. Musculoskelet Surg. 2017;101(1):45–9. 40. Wang C-T, Lin Y-T, Chiang B-L, Lin Y-H, Hou S-M. High molecular weight hyaluronic acid down-regulates the gene expression of osteoarthritis-associated cytokines and enzymes in fibroblast-like synoviocytes from patients with early osteoarthritis. Osteoarthr Cartil. 2006;14(12):1237–47. 41. Irrgang JJ, Anderson AF. Development and validation of health-related quality of life measures for the knee. Clin Orthop Relat Res. 2002;402:95–109. 42. Roos EM, Roos HP, Lohmander LS, Ekdahl C, Beynnon BD. Knee Injury and Osteoarthritis Outcome Score (KOOS)—development of a self-administered outcome measure. J Orthop Sport Phys Ther. 1998;28(2):88–96. 43. Perruccio A V, Canizares M, Hawker GA, Roos EM, Maillefert JF, Lohmander SL. Comparative Validity and Responsiveness of the HOOS-PS and KOOS-PS to the WOMAC Physical Function Subscale in Total Joint Replacement for Osteoarthritis. 44. Salavati M, Mazaheri M, Negahban H, Sohani SM, Ebrahimian MR, Ebrahimi I, et al. Validation of a Persian-version of Knee injury and Osteoarthritis Outcome Score (KOOS) in Iranians with knee injuries. Osteoarthr Cartil. 2008;16(10):1178–82. 45. Higgins LD, Taylor MK, Park D, Ghodadra N, Marchant M, Pietrobon R, et al. Reliability and validity of the International Knee Documentation Committee (IKDC) subjective knee form. Jt Bone Spine. 2007;74(6):594–9. 46. Kujala UM, Kaprio J, Sarno S. Osteoarthritis of weight bearing joints of lower limbs in former elite male athletes. Bmj. 1994;308(6923):231–4. 47. Salini V, Pantalone A, Vanni D. Arthroscopy and Sport Injuries. Arthrosc Sport Inj. 2016;433–7. 48. Broglio K. Randomization in clinical trials: Permuted blocks and stratification. JAMA - J Am Med Assoc. 2018;319(21):2223–4. 49. Emanuel EJ, Wendler D, Grady C. What makes clinical research ethical? JAMA. 2000 May;283(20):2701–11. )

[20.	Peterfy CG, Guermazi A, Zaim S, Tirman PFJ, Miaux Y, White D, et al. Whole-organ magnetic resonance imaging score (WORMS) of the knee in osteoarthritis. Osteoarthr Cartil. 2004;12(3):177–90.](1. Patel DR, Villalobos A. Evaluation and management of knee pain in young athletes: overuse injuries of the knee. Transl Pediatr [Internet]. 2017 Jul;6(3):190–8. Available from: https://pubmed.ncbi.nlm.nih.gov/287950102. Nicolini AP, de Carvalho RT, Matsuda MM, Filho JS, Cohen M. Common injuries in athletes' knee: Experience of a specialized center. Acta Ortop Bras. 2014;22(3):127–31. 3. Tiu T, Craig Van Dien MD. Knee overuse disorders. 4. Fuller CW, Ekstrand J, Junge A, Andersen TE, Bahr R, Dvorak J, et al. Consensus statement on injury definitions and data collection procedures in studies  of football (soccer) injuries. Br J Sports Med. 2006 Mar;40(3):193–201. 5. Majewski M, Susanne H, Klaus S. Epidemiology of athletic knee injuries: A 10-year study. Knee. 2006 Jun;13(3):184–8. 6. Wilder RP, Sethi S. Overuse injuries: tendinopathies, stress fractures, compartment syndrome, and shin  splints. Clin Sports Med. 2004 Jan;23(1):55–81, vi. 7. Aicale R, Tarantino D, Maffulli N. Overuse injuries in sport: a comprehensive overview. J Orthop Surg Res. 2018 Dec;13(1):309. 8. Yang J, Tibbetts AS, Covassin T, Cheng G, Nayar S, Heiden E. Epidemiology of overuse and acute injuries among competitive collegiate athletes. J Athl Train. 2012;47(2):198–204. 9. Bernetti A, Mangone M, Paoloni M, Di Sante L, Murgia M, Santilli V. Corticosteroid and hyaluronic acid injection therapy in tennis elbow (lateral epicondylalgia). Med Sport. 2014;67(2):289–95. 10. Iolascon G, Gimigliano F, Moretti A, De Sire A, Migliore A, Brandi ML, et al. Early osteoarthritis: How to define, diagnose, and manage. A systematic review. Eur Geriatr Med. 2017;8(5–6):383–96. 11. Drawer S, Fuller CW. Propensity for osteoarthritis and lower limb joint pain in retired professional soccer players. Br J Sports Med. 2001;35(6):402–8. 12. Vannini F, Spalding T, Andriolo L, Berruto M, Denti M, Espregueira-Mendes J, et al. Sport and early osteoarthritis: the role of sport in aetiology, progression and treatment of knee osteoarthritis. Knee Surgery, Sport Traumatol Arthrosc. 2016;24(6):1786–96. 13. Fröhlich S, Pazeller S, Cherati AS, Müller E, Frey WO, Spörri J. Overuse injuries in the knee, back and hip of top elite female alpine skiers during  the off-season preparation period: prevalence, severity and their association with traumatic preinjuries and training load. BMJ open Sport Exerc Med. 2020;6(1):e000892. 14. Rincon GA, Vyas D, Zhou J, Fu FH, Oiestad BE, Holm I, et al. \“ Knee function and prevalence of knee osteoarthritis after anterior cruciate ligament reconstruction: a prospective study with 10 to 15 years of follow-up\”. Letter to the editor. Am J Sports Med. 2011;39(4):NP3-author. 15. Kellgren JH, Lawrence J. Radiological assessment of osteo-arthrosis. Ann Rheum Dis. 1957;16(4):494. 16. Altman R, Asch E, Bloch D, Bole G, Borenstein D, Brandt K, et al. Development of criteria for the classification and reporting of osteoarthritis: classification of osteoarthritis of the knee. Arthritis Rheum Off J Am Coll Rheumatol. 1986;29(8):1039–49. 17. Luyten FP, Denti M, Filardo G, Kon E, Engebretsen L. Definition and classification of early osteoarthritis of the knee. Knee Surgery, Sport Traumatol Arthrosc. 2012;20(3):401–6. 18. Hoemann C, Kandel R, Roberts S, Saris DBF, Creemers L, Mainil-Varlet P, et al. International Cartilage Repair Society (ICRS) Recommended Guidelines for  Histological Endpoints for Cartilage Repair Studies in Animal Models and Clinical Trials. Cartilage. 2011 Apr;2(2):153–72. 19. Hunter DJ, Lo GH, Gale D, Grainger AJ, Guermazi A, Conaghan PG. The reliability of a new scoring system for knee osteoarthritis MRI and the validity of bone marrow lesion assessment: BLOKS (Boston–Leeds Osteoarthritis Knee Score). Ann Rheum Dis. 2008;67(2):206–11. 20. Peterfy CG, Guermazi A, Zaim S, Tirman PFJ, Miaux Y, White D, et al. Whole-organ magnetic resonance imaging score (WORMS) of the knee in osteoarthritis. Osteoarthr Cartil. 2004;12(3):177–90. 21. Demange MK, Sisto M, Rodeo S. Future trends for unicompartmental arthritis of the knee: injectables & stem cells. Clin Sports Med. 2014;33(1):161–74. 22. Leslie M. Knee osteoarthritis management therapies. Pain Manag Nurs. 2000;1(2):51–7. 23. Paoloni M, Bernetti A, Belelli A, Brignoli O, Buoso S, Caputi AP, et al. Appropriateness of clinical and organizational criteria for intra-articular injection therapies in osteoarthritis: A Delphi method consensus initiative among experts in Italy. Ann Ist Super Sanita. 2015;51:131–8. 24. Sandmark H, Hogstedt C, Lewold S, Vingård E. Osteoarthrosis of the knee in men and women in association with overweight, smoking, and hormone therapy. Ann Rheum Dis. 1999;58(3):151–5. 25. Christensen BV, Iuhl IU, Vilbek H, Bülow H, Dreijer NC, Rasmussen HF. Acupuncture treatment of severe knee osteoarthrosis. A long‐term study. Acta Anaesthesiol Scand. 1992;36(6):519–25. 26. de Sire A, Agostini F, Lippi L, Mangone M, Marchese S, Cisari C, et al. Oxygen-Ozone Therapy in the Rehabilitation Field: State of the Art on Mechanisms of  Action, Safety and Effectiveness in Patients with Musculoskeletal Disorders. Biomolecules. 2021 Feb;11(3). 27. Fransen M, McCONNELL S. Land-based exercise for osteoarthritis of the knee: a metaanalysis of randomized controlled trials. J Rheumatol. 2009;36(6):1109–17. 28. Hiemstra LA, Kerslake S, Irving C. Anterior knee pain in the athlete. Clin Sports Med. 2014;33(3):437–59. 29. Filardo G, Kon E, Di Matteo B, Pelotti P, Di Martino A, Marcacci M. Platelet-rich plasma for the treatment of patellar tendinopathy: clinical and imaging findings at medium-term follow-up. Int Orthop [Internet]. 2013;37(8):1583–9. Available from: https://doi.org/10.1007/s00264-013-1972-830. Trojian TH, Concoff AL, Joy SM, Hatzenbuehler JR, Saulsberry WJ, Coleman CI. AMSSM Scientific Statement Concerning Viscosupplementation Injections for Knee  Osteoarthritis: Importance for Individual Patient Outcomes. Clin J Sport Med  Off J Can Acad  Sport Med. 2016 Jan;26(1):1–11. 31. Ammar TY, Pereira TAP, Mistura SLL, Kuhn A, Saggin JI, Lopes OV. Viscosupplementation for treating knee osteoarthrosis: review of the literature. Rev Bras Ortop. 2015;50:489–94. 32. Salini V, Pantalone A, Vanni D, Abate M. Viscosupplementation in Athletes. In: Arthroscopy and Sport Injuries. Springer; 2016. p. 433–7. 33. Erdem Y, Gul D, Akpancar S. Comparison of Intraarticular Injections of Hyaluronic Acid versus Dextrose Applied with Periarticular Prolotherapy in the Treatment of Recreational Athletes with Knee Osteoarthritis. Turkish J Sport Med. 2020;55(1):6–13. 34. Kalkhoven J, Coutts AJ, Impellizzeri FM. 'Training load error' is not a more accurate term than 'overuse' injury. Br J Sports Med [Internet]. 2020 Aug 1;54(15):934 LP – 935. Available from: http://bjsm.bmj.com/content/54/15/934.abstract35. Odole AC, Agbomeji OT, Onyeso OKK, Ojo JO, Odunaiya NA. Perspectives of Nigerian Athletes About Physiotherapy Services in Sports Injury Management: Implications for Rehabilitation. J Sport Rehabil. 2021;1(aop):1–8. 36. Hendrika W, Reswari A. The effect of physiotherapy on pain improvement in patients with early knee osteoarthritis at RSU UKI. Int J Med Heal Res. 2021;7(6):52–9. 37. MacKay C, Hawker GA, Jaglal SB. How Do Physical Therapists Approach Management of People With Early Knee Osteoarthritis? A Qualitative Study. Phys Ther [Internet]. 2019;100(2):295–306. Available from: https://doi.org/10.1093/ptj/pzz16438. Hrazdira L, Pilecki Z, Pilecki G. Ultrasound guided application of HA in the soft tissues of the locomotor system in athletes–First experience. Krátká sdělení Botek, M, Stejskal, P, Klimešová, I Vliv normobarické hypoxie na Akt Auton Nerv systému Pilot Stud 191 Drbošalová, V, Stejskal, P, Klugar, M, Krejčíř, V, Hynková, O Charakt klientů komerčn. :205. 39. Ricci M, Micheloni GM, Berti M, Perusi F, Sambugaro E, Vecchini E, et al. Clinical comparison of oral administration and viscosupplementation of hyaluronic acid (HA) in early knee osteoarthritis. Musculoskelet Surg. 2017;101(1):45–9. 40. Wang C-T, Lin Y-T, Chiang B-L, Lin Y-H, Hou S-M. High molecular weight hyaluronic acid down-regulates the gene expression of osteoarthritis-associated cytokines and enzymes in fibroblast-like synoviocytes from patients with early osteoarthritis. Osteoarthr Cartil. 2006;14(12):1237–47. 41. Irrgang JJ, Anderson AF. Development and validation of health-related quality of life measures for the knee. Clin Orthop Relat Res. 2002;402:95–109. 42. Roos EM, Roos HP, Lohmander LS, Ekdahl C, Beynnon BD. Knee Injury and Osteoarthritis Outcome Score (KOOS)—development of a self-administered outcome measure. J Orthop Sport Phys Ther. 1998;28(2):88–96. 43. Perruccio A V, Canizares M, Hawker GA, Roos EM, Maillefert JF, Lohmander SL. Comparative Validity and Responsiveness of the HOOS-PS and KOOS-PS to the WOMAC Physical Function Subscale in Total Joint Replacement for Osteoarthritis. 44. Salavati M, Mazaheri M, Negahban H, Sohani SM, Ebrahimian MR, Ebrahimi I, et al. Validation of a Persian-version of Knee injury and Osteoarthritis Outcome Score (KOOS) in Iranians with knee injuries. Osteoarthr Cartil. 2008;16(10):1178–82. 45. Higgins LD, Taylor MK, Park D, Ghodadra N, Marchant M, Pietrobon R, et al. Reliability and validity of the International Knee Documentation Committee (IKDC) subjective knee form. Jt Bone Spine. 2007;74(6):594–9. 46. Kujala UM, Kaprio J, Sarno S. Osteoarthritis of weight bearing joints of lower limbs in former elite male athletes. Bmj. 1994;308(6923):231–4. 47. Salini V, Pantalone A, Vanni D. Arthroscopy and Sport Injuries. Arthrosc Sport Inj. 2016;433–7. 48. Broglio K. Randomization in clinical trials: Permuted blocks and stratification. JAMA - J Am Med Assoc. 2018;319(21):2223–4. 49. Emanuel EJ, Wendler D, Grady C. What makes clinical research ethical? JAMA. 2000 May;283(20):2701–11. )

[21.	Demange MK, Sisto M, Rodeo S. Future trends for unicompartmental arthritis of the knee: injectables & stem cells. Clin Sports Med. 2014;33(1):161–74.](1. Patel DR, Villalobos A. Evaluation and management of knee pain in young athletes: overuse injuries of the knee. Transl Pediatr [Internet]. 2017 Jul;6(3):190–8. Available from: https://pubmed.ncbi.nlm.nih.gov/287950102. Nicolini AP, de Carvalho RT, Matsuda MM, Filho JS, Cohen M. Common injuries in athletes' knee: Experience of a specialized center. Acta Ortop Bras. 2014;22(3):127–31. 3. Tiu T, Craig Van Dien MD. Knee overuse disorders. 4. Fuller CW, Ekstrand J, Junge A, Andersen TE, Bahr R, Dvorak J, et al. Consensus statement on injury definitions and data collection procedures in studies  of football (soccer) injuries. Br J Sports Med. 2006 Mar;40(3):193–201. 5. Majewski M, Susanne H, Klaus S. Epidemiology of athletic knee injuries: A 10-year study. Knee. 2006 Jun;13(3):184–8. 6. Wilder RP, Sethi S. Overuse injuries: tendinopathies, stress fractures, compartment syndrome, and shin  splints. Clin Sports Med. 2004 Jan;23(1):55–81, vi. 7. Aicale R, Tarantino D, Maffulli N. Overuse injuries in sport: a comprehensive overview. J Orthop Surg Res. 2018 Dec;13(1):309. 8. Yang J, Tibbetts AS, Covassin T, Cheng G, Nayar S, Heiden E. Epidemiology of overuse and acute injuries among competitive collegiate athletes. J Athl Train. 2012;47(2):198–204. 9. Bernetti A, Mangone M, Paoloni M, Di Sante L, Murgia M, Santilli V. Corticosteroid and hyaluronic acid injection therapy in tennis elbow (lateral epicondylalgia). Med Sport. 2014;67(2):289–95. 10. Iolascon G, Gimigliano F, Moretti A, De Sire A, Migliore A, Brandi ML, et al. Early osteoarthritis: How to define, diagnose, and manage. A systematic review. Eur Geriatr Med. 2017;8(5–6):383–96. 11. Drawer S, Fuller CW. Propensity for osteoarthritis and lower limb joint pain in retired professional soccer players. Br J Sports Med. 2001;35(6):402–8. 12. Vannini F, Spalding T, Andriolo L, Berruto M, Denti M, Espregueira-Mendes J, et al. Sport and early osteoarthritis: the role of sport in aetiology, progression and treatment of knee osteoarthritis. Knee Surgery, Sport Traumatol Arthrosc. 2016;24(6):1786–96. 13. Fröhlich S, Pazeller S, Cherati AS, Müller E, Frey WO, Spörri J. Overuse injuries in the knee, back and hip of top elite female alpine skiers during  the off-season preparation period: prevalence, severity and their association with traumatic preinjuries and training load. BMJ open Sport Exerc Med. 2020;6(1):e000892. 14. Rincon GA, Vyas D, Zhou J, Fu FH, Oiestad BE, Holm I, et al. \“ Knee function and prevalence of knee osteoarthritis after anterior cruciate ligament reconstruction: a prospective study with 10 to 15 years of follow-up\”. Letter to the editor. Am J Sports Med. 2011;39(4):NP3-author. 15. Kellgren JH, Lawrence J. Radiological assessment of osteo-arthrosis. Ann Rheum Dis. 1957;16(4):494. 16. Altman R, Asch E, Bloch D, Bole G, Borenstein D, Brandt K, et al. Development of criteria for the classification and reporting of osteoarthritis: classification of osteoarthritis of the knee. Arthritis Rheum Off J Am Coll Rheumatol. 1986;29(8):1039–49. 17. Luyten FP, Denti M, Filardo G, Kon E, Engebretsen L. Definition and classification of early osteoarthritis of the knee. Knee Surgery, Sport Traumatol Arthrosc. 2012;20(3):401–6. 18. Hoemann C, Kandel R, Roberts S, Saris DBF, Creemers L, Mainil-Varlet P, et al. International Cartilage Repair Society (ICRS) Recommended Guidelines for  Histological Endpoints for Cartilage Repair Studies in Animal Models and Clinical Trials. Cartilage. 2011 Apr;2(2):153–72. 19. Hunter DJ, Lo GH, Gale D, Grainger AJ, Guermazi A, Conaghan PG. The reliability of a new scoring system for knee osteoarthritis MRI and the validity of bone marrow lesion assessment: BLOKS (Boston–Leeds Osteoarthritis Knee Score). Ann Rheum Dis. 2008;67(2):206–11. 20. Peterfy CG, Guermazi A, Zaim S, Tirman PFJ, Miaux Y, White D, et al. Whole-organ magnetic resonance imaging score (WORMS) of the knee in osteoarthritis. Osteoarthr Cartil. 2004;12(3):177–90. 21. Demange MK, Sisto M, Rodeo S. Future trends for unicompartmental arthritis of the knee: injectables & stem cells. Clin Sports Med. 2014;33(1):161–74. 22. Leslie M. Knee osteoarthritis management therapies. Pain Manag Nurs. 2000;1(2):51–7. 23. Paoloni M, Bernetti A, Belelli A, Brignoli O, Buoso S, Caputi AP, et al. Appropriateness of clinical and organizational criteria for intra-articular injection therapies in osteoarthritis: A Delphi method consensus initiative among experts in Italy. Ann Ist Super Sanita. 2015;51:131–8. 24. Sandmark H, Hogstedt C, Lewold S, Vingård E. Osteoarthrosis of the knee in men and women in association with overweight, smoking, and hormone therapy. Ann Rheum Dis. 1999;58(3):151–5. 25. Christensen BV, Iuhl IU, Vilbek H, Bülow H, Dreijer NC, Rasmussen HF. Acupuncture treatment of severe knee osteoarthrosis. A long‐term study. Acta Anaesthesiol Scand. 1992;36(6):519–25. 26. de Sire A, Agostini F, Lippi L, Mangone M, Marchese S, Cisari C, et al. Oxygen-Ozone Therapy in the Rehabilitation Field: State of the Art on Mechanisms of  Action, Safety and Effectiveness in Patients with Musculoskeletal Disorders. Biomolecules. 2021 Feb;11(3). 27. Fransen M, McCONNELL S. Land-based exercise for osteoarthritis of the knee: a metaanalysis of randomized controlled trials. J Rheumatol. 2009;36(6):1109–17. 28. Hiemstra LA, Kerslake S, Irving C. Anterior knee pain in the athlete. Clin Sports Med. 2014;33(3):437–59. 29. Filardo G, Kon E, Di Matteo B, Pelotti P, Di Martino A, Marcacci M. Platelet-rich plasma for the treatment of patellar tendinopathy: clinical and imaging findings at medium-term follow-up. Int Orthop [Internet]. 2013;37(8):1583–9. Available from: https://doi.org/10.1007/s00264-013-1972-830. Trojian TH, Concoff AL, Joy SM, Hatzenbuehler JR, Saulsberry WJ, Coleman CI. AMSSM Scientific Statement Concerning Viscosupplementation Injections for Knee  Osteoarthritis: Importance for Individual Patient Outcomes. Clin J Sport Med  Off J Can Acad  Sport Med. 2016 Jan;26(1):1–11. 31. Ammar TY, Pereira TAP, Mistura SLL, Kuhn A, Saggin JI, Lopes OV. Viscosupplementation for treating knee osteoarthrosis: review of the literature. Rev Bras Ortop. 2015;50:489–94. 32. Salini V, Pantalone A, Vanni D, Abate M. Viscosupplementation in Athletes. In: Arthroscopy and Sport Injuries. Springer; 2016. p. 433–7. 33. Erdem Y, Gul D, Akpancar S. Comparison of Intraarticular Injections of Hyaluronic Acid versus Dextrose Applied with Periarticular Prolotherapy in the Treatment of Recreational Athletes with Knee Osteoarthritis. Turkish J Sport Med. 2020;55(1):6–13. 34. Kalkhoven J, Coutts AJ, Impellizzeri FM. 'Training load error' is not a more accurate term than 'overuse' injury. Br J Sports Med [Internet]. 2020 Aug 1;54(15):934 LP – 935. Available from: http://bjsm.bmj.com/content/54/15/934.abstract35. Odole AC, Agbomeji OT, Onyeso OKK, Ojo JO, Odunaiya NA. Perspectives of Nigerian Athletes About Physiotherapy Services in Sports Injury Management: Implications for Rehabilitation. J Sport Rehabil. 2021;1(aop):1–8. 36. Hendrika W, Reswari A. The effect of physiotherapy on pain improvement in patients with early knee osteoarthritis at RSU UKI. Int J Med Heal Res. 2021;7(6):52–9. 37. MacKay C, Hawker GA, Jaglal SB. How Do Physical Therapists Approach Management of People With Early Knee Osteoarthritis? A Qualitative Study. Phys Ther [Internet]. 2019;100(2):295–306. Available from: https://doi.org/10.1093/ptj/pzz16438. Hrazdira L, Pilecki Z, Pilecki G. Ultrasound guided application of HA in the soft tissues of the locomotor system in athletes–First experience. Krátká sdělení Botek, M, Stejskal, P, Klimešová, I Vliv normobarické hypoxie na Akt Auton Nerv systému Pilot Stud 191 Drbošalová, V, Stejskal, P, Klugar, M, Krejčíř, V, Hynková, O Charakt klientů komerčn. :205. 39. Ricci M, Micheloni GM, Berti M, Perusi F, Sambugaro E, Vecchini E, et al. Clinical comparison of oral administration and viscosupplementation of hyaluronic acid (HA) in early knee osteoarthritis. Musculoskelet Surg. 2017;101(1):45–9. 40. Wang C-T, Lin Y-T, Chiang B-L, Lin Y-H, Hou S-M. High molecular weight hyaluronic acid down-regulates the gene expression of osteoarthritis-associated cytokines and enzymes in fibroblast-like synoviocytes from patients with early osteoarthritis. Osteoarthr Cartil. 2006;14(12):1237–47. 41. Irrgang JJ, Anderson AF. Development and validation of health-related quality of life measures for the knee. Clin Orthop Relat Res. 2002;402:95–109. 42. Roos EM, Roos HP, Lohmander LS, Ekdahl C, Beynnon BD. Knee Injury and Osteoarthritis Outcome Score (KOOS)—development of a self-administered outcome measure. J Orthop Sport Phys Ther. 1998;28(2):88–96. 43. Perruccio A V, Canizares M, Hawker GA, Roos EM, Maillefert JF, Lohmander SL. Comparative Validity and Responsiveness of the HOOS-PS and KOOS-PS to the WOMAC Physical Function Subscale in Total Joint Replacement for Osteoarthritis. 44. Salavati M, Mazaheri M, Negahban H, Sohani SM, Ebrahimian MR, Ebrahimi I, et al. Validation of a Persian-version of Knee injury and Osteoarthritis Outcome Score (KOOS) in Iranians with knee injuries. Osteoarthr Cartil. 2008;16(10):1178–82. 45. Higgins LD, Taylor MK, Park D, Ghodadra N, Marchant M, Pietrobon R, et al. Reliability and validity of the International Knee Documentation Committee (IKDC) subjective knee form. Jt Bone Spine. 2007;74(6):594–9. 46. Kujala UM, Kaprio J, Sarno S. Osteoarthritis of weight bearing joints of lower limbs in former elite male athletes. Bmj. 1994;308(6923):231–4. 47. Salini V, Pantalone A, Vanni D. Arthroscopy and Sport Injuries. Arthrosc Sport Inj. 2016;433–7. 48. Broglio K. Randomization in clinical trials: Permuted blocks and stratification. JAMA - J Am Med Assoc. 2018;319(21):2223–4. 49. Emanuel EJ, Wendler D, Grady C. What makes clinical research ethical? JAMA. 2000 May;283(20):2701–11. )

[22.	Leslie M. Knee osteoarthritis management therapies. Pain Manag Nurs. 2000;1(2):51–7.](1. Patel DR, Villalobos A. Evaluation and management of knee pain in young athletes: overuse injuries of the knee. Transl Pediatr [Internet]. 2017 Jul;6(3):190–8. Available from: https://pubmed.ncbi.nlm.nih.gov/287950102. Nicolini AP, de Carvalho RT, Matsuda MM, Filho JS, Cohen M. Common injuries in athletes' knee: Experience of a specialized center. Acta Ortop Bras. 2014;22(3):127–31. 3. Tiu T, Craig Van Dien MD. Knee overuse disorders. 4. Fuller CW, Ekstrand J, Junge A, Andersen TE, Bahr R, Dvorak J, et al. Consensus statement on injury definitions and data collection procedures in studies  of football (soccer) injuries. Br J Sports Med. 2006 Mar;40(3):193–201. 5. Majewski M, Susanne H, Klaus S. Epidemiology of athletic knee injuries: A 10-year study. Knee. 2006 Jun;13(3):184–8. 6. Wilder RP, Sethi S. Overuse injuries: tendinopathies, stress fractures, compartment syndrome, and shin  splints. Clin Sports Med. 2004 Jan;23(1):55–81, vi. 7. Aicale R, Tarantino D, Maffulli N. Overuse injuries in sport: a comprehensive overview. J Orthop Surg Res. 2018 Dec;13(1):309. 8. Yang J, Tibbetts AS, Covassin T, Cheng G, Nayar S, Heiden E. Epidemiology of overuse and acute injuries among competitive collegiate athletes. J Athl Train. 2012;47(2):198–204. 9. Bernetti A, Mangone M, Paoloni M, Di Sante L, Murgia M, Santilli V. Corticosteroid and hyaluronic acid injection therapy in tennis elbow (lateral epicondylalgia). Med Sport. 2014;67(2):289–95. 10. Iolascon G, Gimigliano F, Moretti A, De Sire A, Migliore A, Brandi ML, et al. Early osteoarthritis: How to define, diagnose, and manage. A systematic review. Eur Geriatr Med. 2017;8(5–6):383–96. 11. Drawer S, Fuller CW. Propensity for osteoarthritis and lower limb joint pain in retired professional soccer players. Br J Sports Med. 2001;35(6):402–8. 12. Vannini F, Spalding T, Andriolo L, Berruto M, Denti M, Espregueira-Mendes J, et al. Sport and early osteoarthritis: the role of sport in aetiology, progression and treatment of knee osteoarthritis. Knee Surgery, Sport Traumatol Arthrosc. 2016;24(6):1786–96. 13. Fröhlich S, Pazeller S, Cherati AS, Müller E, Frey WO, Spörri J. Overuse injuries in the knee, back and hip of top elite female alpine skiers during  the off-season preparation period: prevalence, severity and their association with traumatic preinjuries and training load. BMJ open Sport Exerc Med. 2020;6(1):e000892. 14. Rincon GA, Vyas D, Zhou J, Fu FH, Oiestad BE, Holm I, et al. \“ Knee function and prevalence of knee osteoarthritis after anterior cruciate ligament reconstruction: a prospective study with 10 to 15 years of follow-up\”. Letter to the editor. Am J Sports Med. 2011;39(4):NP3-author. 15. Kellgren JH, Lawrence J. Radiological assessment of osteo-arthrosis. Ann Rheum Dis. 1957;16(4):494. 16. Altman R, Asch E, Bloch D, Bole G, Borenstein D, Brandt K, et al. Development of criteria for the classification and reporting of osteoarthritis: classification of osteoarthritis of the knee. Arthritis Rheum Off J Am Coll Rheumatol. 1986;29(8):1039–49. 17. Luyten FP, Denti M, Filardo G, Kon E, Engebretsen L. Definition and classification of early osteoarthritis of the knee. Knee Surgery, Sport Traumatol Arthrosc. 2012;20(3):401–6. 18. Hoemann C, Kandel R, Roberts S, Saris DBF, Creemers L, Mainil-Varlet P, et al. International Cartilage Repair Society (ICRS) Recommended Guidelines for  Histological Endpoints for Cartilage Repair Studies in Animal Models and Clinical Trials. Cartilage. 2011 Apr;2(2):153–72. 19. Hunter DJ, Lo GH, Gale D, Grainger AJ, Guermazi A, Conaghan PG. The reliability of a new scoring system for knee osteoarthritis MRI and the validity of bone marrow lesion assessment: BLOKS (Boston–Leeds Osteoarthritis Knee Score). Ann Rheum Dis. 2008;67(2):206–11. 20. Peterfy CG, Guermazi A, Zaim S, Tirman PFJ, Miaux Y, White D, et al. Whole-organ magnetic resonance imaging score (WORMS) of the knee in osteoarthritis. Osteoarthr Cartil. 2004;12(3):177–90. 21. Demange MK, Sisto M, Rodeo S. Future trends for unicompartmental arthritis of the knee: injectables & stem cells. Clin Sports Med. 2014;33(1):161–74. 22. Leslie M. Knee osteoarthritis management therapies. Pain Manag Nurs. 2000;1(2):51–7. 23. Paoloni M, Bernetti A, Belelli A, Brignoli O, Buoso S, Caputi AP, et al. Appropriateness of clinical and organizational criteria for intra-articular injection therapies in osteoarthritis: A Delphi method consensus initiative among experts in Italy. Ann Ist Super Sanita. 2015;51:131–8. 24. Sandmark H, Hogstedt C, Lewold S, Vingård E. Osteoarthrosis of the knee in men and women in association with overweight, smoking, and hormone therapy. Ann Rheum Dis. 1999;58(3):151–5. 25. Christensen BV, Iuhl IU, Vilbek H, Bülow H, Dreijer NC, Rasmussen HF. Acupuncture treatment of severe knee osteoarthrosis. A long‐term study. Acta Anaesthesiol Scand. 1992;36(6):519–25. 26. de Sire A, Agostini F, Lippi L, Mangone M, Marchese S, Cisari C, et al. Oxygen-Ozone Therapy in the Rehabilitation Field: State of the Art on Mechanisms of  Action, Safety and Effectiveness in Patients with Musculoskeletal Disorders. Biomolecules. 2021 Feb;11(3). 27. Fransen M, McCONNELL S. Land-based exercise for osteoarthritis of the knee: a metaanalysis of randomized controlled trials. J Rheumatol. 2009;36(6):1109–17. 28. Hiemstra LA, Kerslake S, Irving C. Anterior knee pain in the athlete. Clin Sports Med. 2014;33(3):437–59. 29. Filardo G, Kon E, Di Matteo B, Pelotti P, Di Martino A, Marcacci M. Platelet-rich plasma for the treatment of patellar tendinopathy: clinical and imaging findings at medium-term follow-up. Int Orthop [Internet]. 2013;37(8):1583–9. Available from: https://doi.org/10.1007/s00264-013-1972-830. Trojian TH, Concoff AL, Joy SM, Hatzenbuehler JR, Saulsberry WJ, Coleman CI. AMSSM Scientific Statement Concerning Viscosupplementation Injections for Knee  Osteoarthritis: Importance for Individual Patient Outcomes. Clin J Sport Med  Off J Can Acad  Sport Med. 2016 Jan;26(1):1–11. 31. Ammar TY, Pereira TAP, Mistura SLL, Kuhn A, Saggin JI, Lopes OV. Viscosupplementation for treating knee osteoarthrosis: review of the literature. Rev Bras Ortop. 2015;50:489–94. 32. Salini V, Pantalone A, Vanni D, Abate M. Viscosupplementation in Athletes. In: Arthroscopy and Sport Injuries. Springer; 2016. p. 433–7. 33. Erdem Y, Gul D, Akpancar S. Comparison of Intraarticular Injections of Hyaluronic Acid versus Dextrose Applied with Periarticular Prolotherapy in the Treatment of Recreational Athletes with Knee Osteoarthritis. Turkish J Sport Med. 2020;55(1):6–13. 34. Kalkhoven J, Coutts AJ, Impellizzeri FM. 'Training load error' is not a more accurate term than 'overuse' injury. Br J Sports Med [Internet]. 2020 Aug 1;54(15):934 LP – 935. Available from: http://bjsm.bmj.com/content/54/15/934.abstract35. Odole AC, Agbomeji OT, Onyeso OKK, Ojo JO, Odunaiya NA. Perspectives of Nigerian Athletes About Physiotherapy Services in Sports Injury Management: Implications for Rehabilitation. J Sport Rehabil. 2021;1(aop):1–8. 36. Hendrika W, Reswari A. The effect of physiotherapy on pain improvement in patients with early knee osteoarthritis at RSU UKI. Int J Med Heal Res. 2021;7(6):52–9. 37. MacKay C, Hawker GA, Jaglal SB. How Do Physical Therapists Approach Management of People With Early Knee Osteoarthritis? A Qualitative Study. Phys Ther [Internet]. 2019;100(2):295–306. Available from: https://doi.org/10.1093/ptj/pzz16438. Hrazdira L, Pilecki Z, Pilecki G. Ultrasound guided application of HA in the soft tissues of the locomotor system in athletes–First experience. Krátká sdělení Botek, M, Stejskal, P, Klimešová, I Vliv normobarické hypoxie na Akt Auton Nerv systému Pilot Stud 191 Drbošalová, V, Stejskal, P, Klugar, M, Krejčíř, V, Hynková, O Charakt klientů komerčn. :205. 39. Ricci M, Micheloni GM, Berti M, Perusi F, Sambugaro E, Vecchini E, et al. Clinical comparison of oral administration and viscosupplementation of hyaluronic acid (HA) in early knee osteoarthritis. Musculoskelet Surg. 2017;101(1):45–9. 40. Wang C-T, Lin Y-T, Chiang B-L, Lin Y-H, Hou S-M. High molecular weight hyaluronic acid down-regulates the gene expression of osteoarthritis-associated cytokines and enzymes in fibroblast-like synoviocytes from patients with early osteoarthritis. Osteoarthr Cartil. 2006;14(12):1237–47. 41. Irrgang JJ, Anderson AF. Development and validation of health-related quality of life measures for the knee. Clin Orthop Relat Res. 2002;402:95–109. 42. Roos EM, Roos HP, Lohmander LS, Ekdahl C, Beynnon BD. Knee Injury and Osteoarthritis Outcome Score (KOOS)—development of a self-administered outcome measure. J Orthop Sport Phys Ther. 1998;28(2):88–96. 43. Perruccio A V, Canizares M, Hawker GA, Roos EM, Maillefert JF, Lohmander SL. Comparative Validity and Responsiveness of the HOOS-PS and KOOS-PS to the WOMAC Physical Function Subscale in Total Joint Replacement for Osteoarthritis. 44. Salavati M, Mazaheri M, Negahban H, Sohani SM, Ebrahimian MR, Ebrahimi I, et al. Validation of a Persian-version of Knee injury and Osteoarthritis Outcome Score (KOOS) in Iranians with knee injuries. Osteoarthr Cartil. 2008;16(10):1178–82. 45. Higgins LD, Taylor MK, Park D, Ghodadra N, Marchant M, Pietrobon R, et al. Reliability and validity of the International Knee Documentation Committee (IKDC) subjective knee form. Jt Bone Spine. 2007;74(6):594–9. 46. Kujala UM, Kaprio J, Sarno S. Osteoarthritis of weight bearing joints of lower limbs in former elite male athletes. Bmj. 1994;308(6923):231–4. 47. Salini V, Pantalone A, Vanni D. Arthroscopy and Sport Injuries. Arthrosc Sport Inj. 2016;433–7. 48. Broglio K. Randomization in clinical trials: Permuted blocks and stratification. JAMA - J Am Med Assoc. 2018;319(21):2223–4. 49. Emanuel EJ, Wendler D, Grady C. What makes clinical research ethical? JAMA. 2000 May;283(20):2701–11. )

[23.	Paoloni M, Bernetti A, Belelli A, Brignoli O, Buoso S, Caputi AP, et al. Appropriateness of clinical and organizational criteria for intra-articular injection therapies in osteoarthritis: A Delphi method consensus initiative among experts in Italy. Ann Ist Super Sanita. 2015;51:131–8.](1. Patel DR, Villalobos A. Evaluation and management of knee pain in young athletes: overuse injuries of the knee. Transl Pediatr [Internet]. 2017 Jul;6(3):190–8. Available from: https://pubmed.ncbi.nlm.nih.gov/287950102. Nicolini AP, de Carvalho RT, Matsuda MM, Filho JS, Cohen M. Common injuries in athletes' knee: Experience of a specialized center. Acta Ortop Bras. 2014;22(3):127–31. 3. Tiu T, Craig Van Dien MD. Knee overuse disorders. 4. Fuller CW, Ekstrand J, Junge A, Andersen TE, Bahr R, Dvorak J, et al. Consensus statement on injury definitions and data collection procedures in studies  of football (soccer) injuries. Br J Sports Med. 2006 Mar;40(3):193–201. 5. Majewski M, Susanne H, Klaus S. Epidemiology of athletic knee injuries: A 10-year study. Knee. 2006 Jun;13(3):184–8. 6. Wilder RP, Sethi S. Overuse injuries: tendinopathies, stress fractures, compartment syndrome, and shin  splints. Clin Sports Med. 2004 Jan;23(1):55–81, vi. 7. Aicale R, Tarantino D, Maffulli N. Overuse injuries in sport: a comprehensive overview. J Orthop Surg Res. 2018 Dec;13(1):309. 8. Yang J, Tibbetts AS, Covassin T, Cheng G, Nayar S, Heiden E. Epidemiology of overuse and acute injuries among competitive collegiate athletes. J Athl Train. 2012;47(2):198–204. 9. Bernetti A, Mangone M, Paoloni M, Di Sante L, Murgia M, Santilli V. Corticosteroid and hyaluronic acid injection therapy in tennis elbow (lateral epicondylalgia). Med Sport. 2014;67(2):289–95. 10. Iolascon G, Gimigliano F, Moretti A, De Sire A, Migliore A, Brandi ML, et al. Early osteoarthritis: How to define, diagnose, and manage. A systematic review. Eur Geriatr Med. 2017;8(5–6):383–96. 11. Drawer S, Fuller CW. Propensity for osteoarthritis and lower limb joint pain in retired professional soccer players. Br J Sports Med. 2001;35(6):402–8. 12. Vannini F, Spalding T, Andriolo L, Berruto M, Denti M, Espregueira-Mendes J, et al. Sport and early osteoarthritis: the role of sport in aetiology, progression and treatment of knee osteoarthritis. Knee Surgery, Sport Traumatol Arthrosc. 2016;24(6):1786–96. 13. Fröhlich S, Pazeller S, Cherati AS, Müller E, Frey WO, Spörri J. Overuse injuries in the knee, back and hip of top elite female alpine skiers during  the off-season preparation period: prevalence, severity and their association with traumatic preinjuries and training load. BMJ open Sport Exerc Med. 2020;6(1):e000892. 14. Rincon GA, Vyas D, Zhou J, Fu FH, Oiestad BE, Holm I, et al. \“ Knee function and prevalence of knee osteoarthritis after anterior cruciate ligament reconstruction: a prospective study with 10 to 15 years of follow-up\”. Letter to the editor. Am J Sports Med. 2011;39(4):NP3-author. 15. Kellgren JH, Lawrence J. Radiological assessment of osteo-arthrosis. Ann Rheum Dis. 1957;16(4):494. 16. Altman R, Asch E, Bloch D, Bole G, Borenstein D, Brandt K, et al. Development of criteria for the classification and reporting of osteoarthritis: classification of osteoarthritis of the knee. Arthritis Rheum Off J Am Coll Rheumatol. 1986;29(8):1039–49. 17. Luyten FP, Denti M, Filardo G, Kon E, Engebretsen L. Definition and classification of early osteoarthritis of the knee. Knee Surgery, Sport Traumatol Arthrosc. 2012;20(3):401–6. 18. Hoemann C, Kandel R, Roberts S, Saris DBF, Creemers L, Mainil-Varlet P, et al. International Cartilage Repair Society (ICRS) Recommended Guidelines for  Histological Endpoints for Cartilage Repair Studies in Animal Models and Clinical Trials. Cartilage. 2011 Apr;2(2):153–72. 19. Hunter DJ, Lo GH, Gale D, Grainger AJ, Guermazi A, Conaghan PG. The reliability of a new scoring system for knee osteoarthritis MRI and the validity of bone marrow lesion assessment: BLOKS (Boston–Leeds Osteoarthritis Knee Score). Ann Rheum Dis. 2008;67(2):206–11. 20. Peterfy CG, Guermazi A, Zaim S, Tirman PFJ, Miaux Y, White D, et al. Whole-organ magnetic resonance imaging score (WORMS) of the knee in osteoarthritis. Osteoarthr Cartil. 2004;12(3):177–90. 21. Demange MK, Sisto M, Rodeo S. Future trends for unicompartmental arthritis of the knee: injectables & stem cells. Clin Sports Med. 2014;33(1):161–74. 22. Leslie M. Knee osteoarthritis management therapies. Pain Manag Nurs. 2000;1(2):51–7. 23. Paoloni M, Bernetti A, Belelli A, Brignoli O, Buoso S, Caputi AP, et al. Appropriateness of clinical and organizational criteria for intra-articular injection therapies in osteoarthritis: A Delphi method consensus initiative among experts in Italy. Ann Ist Super Sanita. 2015;51:131–8. 24. Sandmark H, Hogstedt C, Lewold S, Vingård E. Osteoarthrosis of the knee in men and women in association with overweight, smoking, and hormone therapy. Ann Rheum Dis. 1999;58(3):151–5. 25. Christensen BV, Iuhl IU, Vilbek H, Bülow H, Dreijer NC, Rasmussen HF. Acupuncture treatment of severe knee osteoarthrosis. A long‐term study. Acta Anaesthesiol Scand. 1992;36(6):519–25. 26. de Sire A, Agostini F, Lippi L, Mangone M, Marchese S, Cisari C, et al. Oxygen-Ozone Therapy in the Rehabilitation Field: State of the Art on Mechanisms of  Action, Safety and Effectiveness in Patients with Musculoskeletal Disorders. Biomolecules. 2021 Feb;11(3). 27. Fransen M, McCONNELL S. Land-based exercise for osteoarthritis of the knee: a metaanalysis of randomized controlled trials. J Rheumatol. 2009;36(6):1109–17. 28. Hiemstra LA, Kerslake S, Irving C. Anterior knee pain in the athlete. Clin Sports Med. 2014;33(3):437–59. 29. Filardo G, Kon E, Di Matteo B, Pelotti P, Di Martino A, Marcacci M. Platelet-rich plasma for the treatment of patellar tendinopathy: clinical and imaging findings at medium-term follow-up. Int Orthop [Internet]. 2013;37(8):1583–9. Available from: https://doi.org/10.1007/s00264-013-1972-830. Trojian TH, Concoff AL, Joy SM, Hatzenbuehler JR, Saulsberry WJ, Coleman CI. AMSSM Scientific Statement Concerning Viscosupplementation Injections for Knee  Osteoarthritis: Importance for Individual Patient Outcomes. Clin J Sport Med  Off J Can Acad  Sport Med. 2016 Jan;26(1):1–11. 31. Ammar TY, Pereira TAP, Mistura SLL, Kuhn A, Saggin JI, Lopes OV. Viscosupplementation for treating knee osteoarthrosis: review of the literature. Rev Bras Ortop. 2015;50:489–94. 32. Salini V, Pantalone A, Vanni D, Abate M. Viscosupplementation in Athletes. In: Arthroscopy and Sport Injuries. Springer; 2016. p. 433–7. 33. Erdem Y, Gul D, Akpancar S. Comparison of Intraarticular Injections of Hyaluronic Acid versus Dextrose Applied with Periarticular Prolotherapy in the Treatment of Recreational Athletes with Knee Osteoarthritis. Turkish J Sport Med. 2020;55(1):6–13. 34. Kalkhoven J, Coutts AJ, Impellizzeri FM. 'Training load error' is not a more accurate term than 'overuse' injury. Br J Sports Med [Internet]. 2020 Aug 1;54(15):934 LP – 935. Available from: http://bjsm.bmj.com/content/54/15/934.abstract35. Odole AC, Agbomeji OT, Onyeso OKK, Ojo JO, Odunaiya NA. Perspectives of Nigerian Athletes About Physiotherapy Services in Sports Injury Management: Implications for Rehabilitation. J Sport Rehabil. 2021;1(aop):1–8. 36. Hendrika W, Reswari A. The effect of physiotherapy on pain improvement in patients with early knee osteoarthritis at RSU UKI. Int J Med Heal Res. 2021;7(6):52–9. 37. MacKay C, Hawker GA, Jaglal SB. How Do Physical Therapists Approach Management of People With Early Knee Osteoarthritis? A Qualitative Study. Phys Ther [Internet]. 2019;100(2):295–306. Available from: https://doi.org/10.1093/ptj/pzz16438. Hrazdira L, Pilecki Z, Pilecki G. Ultrasound guided application of HA in the soft tissues of the locomotor system in athletes–First experience. Krátká sdělení Botek, M, Stejskal, P, Klimešová, I Vliv normobarické hypoxie na Akt Auton Nerv systému Pilot Stud 191 Drbošalová, V, Stejskal, P, Klugar, M, Krejčíř, V, Hynková, O Charakt klientů komerčn. :205. 39. Ricci M, Micheloni GM, Berti M, Perusi F, Sambugaro E, Vecchini E, et al. Clinical comparison of oral administration and viscosupplementation of hyaluronic acid (HA) in early knee osteoarthritis. Musculoskelet Surg. 2017;101(1):45–9. 40. Wang C-T, Lin Y-T, Chiang B-L, Lin Y-H, Hou S-M. High molecular weight hyaluronic acid down-regulates the gene expression of osteoarthritis-associated cytokines and enzymes in fibroblast-like synoviocytes from patients with early osteoarthritis. Osteoarthr Cartil. 2006;14(12):1237–47. 41. Irrgang JJ, Anderson AF. Development and validation of health-related quality of life measures for the knee. Clin Orthop Relat Res. 2002;402:95–109. 42. Roos EM, Roos HP, Lohmander LS, Ekdahl C, Beynnon BD. Knee Injury and Osteoarthritis Outcome Score (KOOS)—development of a self-administered outcome measure. J Orthop Sport Phys Ther. 1998;28(2):88–96. 43. Perruccio A V, Canizares M, Hawker GA, Roos EM, Maillefert JF, Lohmander SL. Comparative Validity and Responsiveness of the HOOS-PS and KOOS-PS to the WOMAC Physical Function Subscale in Total Joint Replacement for Osteoarthritis. 44. Salavati M, Mazaheri M, Negahban H, Sohani SM, Ebrahimian MR, Ebrahimi I, et al. Validation of a Persian-version of Knee injury and Osteoarthritis Outcome Score (KOOS) in Iranians with knee injuries. Osteoarthr Cartil. 2008;16(10):1178–82. 45. Higgins LD, Taylor MK, Park D, Ghodadra N, Marchant M, Pietrobon R, et al. Reliability and validity of the International Knee Documentation Committee (IKDC) subjective knee form. Jt Bone Spine. 2007;74(6):594–9. 46. Kujala UM, Kaprio J, Sarno S. Osteoarthritis of weight bearing joints of lower limbs in former elite male athletes. Bmj. 1994;308(6923):231–4. 47. Salini V, Pantalone A, Vanni D. Arthroscopy and Sport Injuries. Arthrosc Sport Inj. 2016;433–7. 48. Broglio K. Randomization in clinical trials: Permuted blocks and stratification. JAMA - J Am Med Assoc. 2018;319(21):2223–4. 49. Emanuel EJ, Wendler D, Grady C. What makes clinical research ethical? JAMA. 2000 May;283(20):2701–11. )

[24.	Sandmark H, Hogstedt C, Lewold S, Vingård E. Osteoarthrosis of the knee in men and women in association with overweight, smoking, and hormone therapy. Ann Rheum Dis. 1999;58(3):151–5.](1. Patel DR, Villalobos A. Evaluation and management of knee pain in young athletes: overuse injuries of the knee. Transl Pediatr [Internet]. 2017 Jul;6(3):190–8. Available from: https://pubmed.ncbi.nlm.nih.gov/287950102. Nicolini AP, de Carvalho RT, Matsuda MM, Filho JS, Cohen M. Common injuries in athletes' knee: Experience of a specialized center. Acta Ortop Bras. 2014;22(3):127–31. 3. Tiu T, Craig Van Dien MD. Knee overuse disorders. 4. Fuller CW, Ekstrand J, Junge A, Andersen TE, Bahr R, Dvorak J, et al. Consensus statement on injury definitions and data collection procedures in studies  of football (soccer) injuries. Br J Sports Med. 2006 Mar;40(3):193–201. 5. Majewski M, Susanne H, Klaus S. Epidemiology of athletic knee injuries: A 10-year study. Knee. 2006 Jun;13(3):184–8. 6. Wilder RP, Sethi S. Overuse injuries: tendinopathies, stress fractures, compartment syndrome, and shin  splints. Clin Sports Med. 2004 Jan;23(1):55–81, vi. 7. Aicale R, Tarantino D, Maffulli N. Overuse injuries in sport: a comprehensive overview. J Orthop Surg Res. 2018 Dec;13(1):309. 8. Yang J, Tibbetts AS, Covassin T, Cheng G, Nayar S, Heiden E. Epidemiology of overuse and acute injuries among competitive collegiate athletes. J Athl Train. 2012;47(2):198–204. 9. Bernetti A, Mangone M, Paoloni M, Di Sante L, Murgia M, Santilli V. Corticosteroid and hyaluronic acid injection therapy in tennis elbow (lateral epicondylalgia). Med Sport. 2014;67(2):289–95. 10. Iolascon G, Gimigliano F, Moretti A, De Sire A, Migliore A, Brandi ML, et al. Early osteoarthritis: How to define, diagnose, and manage. A systematic review. Eur Geriatr Med. 2017;8(5–6):383–96. 11. Drawer S, Fuller CW. Propensity for osteoarthritis and lower limb joint pain in retired professional soccer players. Br J Sports Med. 2001;35(6):402–8. 12. Vannini F, Spalding T, Andriolo L, Berruto M, Denti M, Espregueira-Mendes J, et al. Sport and early osteoarthritis: the role of sport in aetiology, progression and treatment of knee osteoarthritis. Knee Surgery, Sport Traumatol Arthrosc. 2016;24(6):1786–96. 13. Fröhlich S, Pazeller S, Cherati AS, Müller E, Frey WO, Spörri J. Overuse injuries in the knee, back and hip of top elite female alpine skiers during  the off-season preparation period: prevalence, severity and their association with traumatic preinjuries and training load. BMJ open Sport Exerc Med. 2020;6(1):e000892. 14. Rincon GA, Vyas D, Zhou J, Fu FH, Oiestad BE, Holm I, et al. \“ Knee function and prevalence of knee osteoarthritis after anterior cruciate ligament reconstruction: a prospective study with 10 to 15 years of follow-up\”. Letter to the editor. Am J Sports Med. 2011;39(4):NP3-author. 15. Kellgren JH, Lawrence J. Radiological assessment of osteo-arthrosis. Ann Rheum Dis. 1957;16(4):494. 16. Altman R, Asch E, Bloch D, Bole G, Borenstein D, Brandt K, et al. Development of criteria for the classification and reporting of osteoarthritis: classification of osteoarthritis of the knee. Arthritis Rheum Off J Am Coll Rheumatol. 1986;29(8):1039–49. 17. Luyten FP, Denti M, Filardo G, Kon E, Engebretsen L. Definition and classification of early osteoarthritis of the knee. Knee Surgery, Sport Traumatol Arthrosc. 2012;20(3):401–6. 18. Hoemann C, Kandel R, Roberts S, Saris DBF, Creemers L, Mainil-Varlet P, et al. International Cartilage Repair Society (ICRS) Recommended Guidelines for  Histological Endpoints for Cartilage Repair Studies in Animal Models and Clinical Trials. Cartilage. 2011 Apr;2(2):153–72. 19. Hunter DJ, Lo GH, Gale D, Grainger AJ, Guermazi A, Conaghan PG. The reliability of a new scoring system for knee osteoarthritis MRI and the validity of bone marrow lesion assessment: BLOKS (Boston–Leeds Osteoarthritis Knee Score). Ann Rheum Dis. 2008;67(2):206–11. 20. Peterfy CG, Guermazi A, Zaim S, Tirman PFJ, Miaux Y, White D, et al. Whole-organ magnetic resonance imaging score (WORMS) of the knee in osteoarthritis. Osteoarthr Cartil. 2004;12(3):177–90. 21. Demange MK, Sisto M, Rodeo S. Future trends for unicompartmental arthritis of the knee: injectables & stem cells. Clin Sports Med. 2014;33(1):161–74. 22. Leslie M. Knee osteoarthritis management therapies. Pain Manag Nurs. 2000;1(2):51–7. 23. Paoloni M, Bernetti A, Belelli A, Brignoli O, Buoso S, Caputi AP, et al. Appropriateness of clinical and organizational criteria for intra-articular injection therapies in osteoarthritis: A Delphi method consensus initiative among experts in Italy. Ann Ist Super Sanita. 2015;51:131–8. 24. Sandmark H, Hogstedt C, Lewold S, Vingård E. Osteoarthrosis of the knee in men and women in association with overweight, smoking, and hormone therapy. Ann Rheum Dis. 1999;58(3):151–5. 25. Christensen BV, Iuhl IU, Vilbek H, Bülow H, Dreijer NC, Rasmussen HF. Acupuncture treatment of severe knee osteoarthrosis. A long‐term study. Acta Anaesthesiol Scand. 1992;36(6):519–25. 26. de Sire A, Agostini F, Lippi L, Mangone M, Marchese S, Cisari C, et al. Oxygen-Ozone Therapy in the Rehabilitation Field: State of the Art on Mechanisms of  Action, Safety and Effectiveness in Patients with Musculoskeletal Disorders. Biomolecules. 2021 Feb;11(3). 27. Fransen M, McCONNELL S. Land-based exercise for osteoarthritis of the knee: a metaanalysis of randomized controlled trials. J Rheumatol. 2009;36(6):1109–17. 28. Hiemstra LA, Kerslake S, Irving C. Anterior knee pain in the athlete. Clin Sports Med. 2014;33(3):437–59. 29. Filardo G, Kon E, Di Matteo B, Pelotti P, Di Martino A, Marcacci M. Platelet-rich plasma for the treatment of patellar tendinopathy: clinical and imaging findings at medium-term follow-up. Int Orthop [Internet]. 2013;37(8):1583–9. Available from: https://doi.org/10.1007/s00264-013-1972-830. Trojian TH, Concoff AL, Joy SM, Hatzenbuehler JR, Saulsberry WJ, Coleman CI. AMSSM Scientific Statement Concerning Viscosupplementation Injections for Knee  Osteoarthritis: Importance for Individual Patient Outcomes. Clin J Sport Med  Off J Can Acad  Sport Med. 2016 Jan;26(1):1–11. 31. Ammar TY, Pereira TAP, Mistura SLL, Kuhn A, Saggin JI, Lopes OV. Viscosupplementation for treating knee osteoarthrosis: review of the literature. Rev Bras Ortop. 2015;50:489–94. 32. Salini V, Pantalone A, Vanni D, Abate M. Viscosupplementation in Athletes. In: Arthroscopy and Sport Injuries. Springer; 2016. p. 433–7. 33. Erdem Y, Gul D, Akpancar S. Comparison of Intraarticular Injections of Hyaluronic Acid versus Dextrose Applied with Periarticular Prolotherapy in the Treatment of Recreational Athletes with Knee Osteoarthritis. Turkish J Sport Med. 2020;55(1):6–13. 34. Kalkhoven J, Coutts AJ, Impellizzeri FM. 'Training load error' is not a more accurate term than 'overuse' injury. Br J Sports Med [Internet]. 2020 Aug 1;54(15):934 LP – 935. Available from: http://bjsm.bmj.com/content/54/15/934.abstract35. Odole AC, Agbomeji OT, Onyeso OKK, Ojo JO, Odunaiya NA. Perspectives of Nigerian Athletes About Physiotherapy Services in Sports Injury Management: Implications for Rehabilitation. J Sport Rehabil. 2021;1(aop):1–8. 36. Hendrika W, Reswari A. The effect of physiotherapy on pain improvement in patients with early knee osteoarthritis at RSU UKI. Int J Med Heal Res. 2021;7(6):52–9. 37. MacKay C, Hawker GA, Jaglal SB. How Do Physical Therapists Approach Management of People With Early Knee Osteoarthritis? A Qualitative Study. Phys Ther [Internet]. 2019;100(2):295–306. Available from: https://doi.org/10.1093/ptj/pzz16438. Hrazdira L, Pilecki Z, Pilecki G. Ultrasound guided application of HA in the soft tissues of the locomotor system in athletes–First experience. Krátká sdělení Botek, M, Stejskal, P, Klimešová, I Vliv normobarické hypoxie na Akt Auton Nerv systému Pilot Stud 191 Drbošalová, V, Stejskal, P, Klugar, M, Krejčíř, V, Hynková, O Charakt klientů komerčn. :205. 39. Ricci M, Micheloni GM, Berti M, Perusi F, Sambugaro E, Vecchini E, et al. Clinical comparison of oral administration and viscosupplementation of hyaluronic acid (HA) in early knee osteoarthritis. Musculoskelet Surg. 2017;101(1):45–9. 40. Wang C-T, Lin Y-T, Chiang B-L, Lin Y-H, Hou S-M. High molecular weight hyaluronic acid down-regulates the gene expression of osteoarthritis-associated cytokines and enzymes in fibroblast-like synoviocytes from patients with early osteoarthritis. Osteoarthr Cartil. 2006;14(12):1237–47. 41. Irrgang JJ, Anderson AF. Development and validation of health-related quality of life measures for the knee. Clin Orthop Relat Res. 2002;402:95–109. 42. Roos EM, Roos HP, Lohmander LS, Ekdahl C, Beynnon BD. Knee Injury and Osteoarthritis Outcome Score (KOOS)—development of a self-administered outcome measure. J Orthop Sport Phys Ther. 1998;28(2):88–96. 43. Perruccio A V, Canizares M, Hawker GA, Roos EM, Maillefert JF, Lohmander SL. Comparative Validity and Responsiveness of the HOOS-PS and KOOS-PS to the WOMAC Physical Function Subscale in Total Joint Replacement for Osteoarthritis. 44. Salavati M, Mazaheri M, Negahban H, Sohani SM, Ebrahimian MR, Ebrahimi I, et al. Validation of a Persian-version of Knee injury and Osteoarthritis Outcome Score (KOOS) in Iranians with knee injuries. Osteoarthr Cartil. 2008;16(10):1178–82. 45. Higgins LD, Taylor MK, Park D, Ghodadra N, Marchant M, Pietrobon R, et al. Reliability and validity of the International Knee Documentation Committee (IKDC) subjective knee form. Jt Bone Spine. 2007;74(6):594–9. 46. Kujala UM, Kaprio J, Sarno S. Osteoarthritis of weight bearing joints of lower limbs in former elite male athletes. Bmj. 1994;308(6923):231–4. 47. Salini V, Pantalone A, Vanni D. Arthroscopy and Sport Injuries. Arthrosc Sport Inj. 2016;433–7. 48. Broglio K. Randomization in clinical trials: Permuted blocks and stratification. JAMA - J Am Med Assoc. 2018;319(21):2223–4. 49. Emanuel EJ, Wendler D, Grady C. What makes clinical research ethical? JAMA. 2000 May;283(20):2701–11. )

[25.	Christensen BV, Iuhl IU, Vilbek H, Bülow H, Dreijer NC, Rasmussen HF. Acupuncture treatment of severe knee osteoarthrosis. A long‐term study. Acta Anaesthesiol Scand. 1992;36(6):519–25.](1. Patel DR, Villalobos A. Evaluation and management of knee pain in young athletes: overuse injuries of the knee. Transl Pediatr [Internet]. 2017 Jul;6(3):190–8. Available from: https://pubmed.ncbi.nlm.nih.gov/287950102. Nicolini AP, de Carvalho RT, Matsuda MM, Filho JS, Cohen M. Common injuries in athletes' knee: Experience of a specialized center. Acta Ortop Bras. 2014;22(3):127–31. 3. Tiu T, Craig Van Dien MD. Knee overuse disorders. 4. Fuller CW, Ekstrand J, Junge A, Andersen TE, Bahr R, Dvorak J, et al. Consensus statement on injury definitions and data collection procedures in studies  of football (soccer) injuries. Br J Sports Med. 2006 Mar;40(3):193–201. 5. Majewski M, Susanne H, Klaus S. Epidemiology of athletic knee injuries: A 10-year study. Knee. 2006 Jun;13(3):184–8. 6. Wilder RP, Sethi S. Overuse injuries: tendinopathies, stress fractures, compartment syndrome, and shin  splints. Clin Sports Med. 2004 Jan;23(1):55–81, vi. 7. Aicale R, Tarantino D, Maffulli N. Overuse injuries in sport: a comprehensive overview. J Orthop Surg Res. 2018 Dec;13(1):309. 8. Yang J, Tibbetts AS, Covassin T, Cheng G, Nayar S, Heiden E. Epidemiology of overuse and acute injuries among competitive collegiate athletes. J Athl Train. 2012;47(2):198–204. 9. Bernetti A, Mangone M, Paoloni M, Di Sante L, Murgia M, Santilli V. Corticosteroid and hyaluronic acid injection therapy in tennis elbow (lateral epicondylalgia). Med Sport. 2014;67(2):289–95. 10. Iolascon G, Gimigliano F, Moretti A, De Sire A, Migliore A, Brandi ML, et al. Early osteoarthritis: How to define, diagnose, and manage. A systematic review. Eur Geriatr Med. 2017;8(5–6):383–96. 11. Drawer S, Fuller CW. Propensity for osteoarthritis and lower limb joint pain in retired professional soccer players. Br J Sports Med. 2001;35(6):402–8. 12. Vannini F, Spalding T, Andriolo L, Berruto M, Denti M, Espregueira-Mendes J, et al. Sport and early osteoarthritis: the role of sport in aetiology, progression and treatment of knee osteoarthritis. Knee Surgery, Sport Traumatol Arthrosc. 2016;24(6):1786–96. 13. Fröhlich S, Pazeller S, Cherati AS, Müller E, Frey WO, Spörri J. Overuse injuries in the knee, back and hip of top elite female alpine skiers during  the off-season preparation period: prevalence, severity and their association with traumatic preinjuries and training load. BMJ open Sport Exerc Med. 2020;6(1):e000892. 14. Rincon GA, Vyas D, Zhou J, Fu FH, Oiestad BE, Holm I, et al. \“ Knee function and prevalence of knee osteoarthritis after anterior cruciate ligament reconstruction: a prospective study with 10 to 15 years of follow-up\”. Letter to the editor. Am J Sports Med. 2011;39(4):NP3-author. 15. Kellgren JH, Lawrence J. Radiological assessment of osteo-arthrosis. Ann Rheum Dis. 1957;16(4):494. 16. Altman R, Asch E, Bloch D, Bole G, Borenstein D, Brandt K, et al. Development of criteria for the classification and reporting of osteoarthritis: classification of osteoarthritis of the knee. Arthritis Rheum Off J Am Coll Rheumatol. 1986;29(8):1039–49. 17. Luyten FP, Denti M, Filardo G, Kon E, Engebretsen L. Definition and classification of early osteoarthritis of the knee. Knee Surgery, Sport Traumatol Arthrosc. 2012;20(3):401–6. 18. Hoemann C, Kandel R, Roberts S, Saris DBF, Creemers L, Mainil-Varlet P, et al. International Cartilage Repair Society (ICRS) Recommended Guidelines for  Histological Endpoints for Cartilage Repair Studies in Animal Models and Clinical Trials. Cartilage. 2011 Apr;2(2):153–72. 19. Hunter DJ, Lo GH, Gale D, Grainger AJ, Guermazi A, Conaghan PG. The reliability of a new scoring system for knee osteoarthritis MRI and the validity of bone marrow lesion assessment: BLOKS (Boston–Leeds Osteoarthritis Knee Score). Ann Rheum Dis. 2008;67(2):206–11. 20. Peterfy CG, Guermazi A, Zaim S, Tirman PFJ, Miaux Y, White D, et al. Whole-organ magnetic resonance imaging score (WORMS) of the knee in osteoarthritis. Osteoarthr Cartil. 2004;12(3):177–90. 21. Demange MK, Sisto M, Rodeo S. Future trends for unicompartmental arthritis of the knee: injectables & stem cells. Clin Sports Med. 2014;33(1):161–74. 22. Leslie M. Knee osteoarthritis management therapies. Pain Manag Nurs. 2000;1(2):51–7. 23. Paoloni M, Bernetti A, Belelli A, Brignoli O, Buoso S, Caputi AP, et al. Appropriateness of clinical and organizational criteria for intra-articular injection therapies in osteoarthritis: A Delphi method consensus initiative among experts in Italy. Ann Ist Super Sanita. 2015;51:131–8. 24. Sandmark H, Hogstedt C, Lewold S, Vingård E. Osteoarthrosis of the knee in men and women in association with overweight, smoking, and hormone therapy. Ann Rheum Dis. 1999;58(3):151–5. 25. Christensen BV, Iuhl IU, Vilbek H, Bülow H, Dreijer NC, Rasmussen HF. Acupuncture treatment of severe knee osteoarthrosis. A long‐term study. Acta Anaesthesiol Scand. 1992;36(6):519–25. 26. de Sire A, Agostini F, Lippi L, Mangone M, Marchese S, Cisari C, et al. Oxygen-Ozone Therapy in the Rehabilitation Field: State of the Art on Mechanisms of  Action, Safety and Effectiveness in Patients with Musculoskeletal Disorders. Biomolecules. 2021 Feb;11(3). 27. Fransen M, McCONNELL S. Land-based exercise for osteoarthritis of the knee: a metaanalysis of randomized controlled trials. J Rheumatol. 2009;36(6):1109–17. 28. Hiemstra LA, Kerslake S, Irving C. Anterior knee pain in the athlete. Clin Sports Med. 2014;33(3):437–59. 29. Filardo G, Kon E, Di Matteo B, Pelotti P, Di Martino A, Marcacci M. Platelet-rich plasma for the treatment of patellar tendinopathy: clinical and imaging findings at medium-term follow-up. Int Orthop [Internet]. 2013;37(8):1583–9. Available from: https://doi.org/10.1007/s00264-013-1972-830. Trojian TH, Concoff AL, Joy SM, Hatzenbuehler JR, Saulsberry WJ, Coleman CI. AMSSM Scientific Statement Concerning Viscosupplementation Injections for Knee  Osteoarthritis: Importance for Individual Patient Outcomes. Clin J Sport Med  Off J Can Acad  Sport Med. 2016 Jan;26(1):1–11. 31. Ammar TY, Pereira TAP, Mistura SLL, Kuhn A, Saggin JI, Lopes OV. Viscosupplementation for treating knee osteoarthrosis: review of the literature. Rev Bras Ortop. 2015;50:489–94. 32. Salini V, Pantalone A, Vanni D, Abate M. Viscosupplementation in Athletes. In: Arthroscopy and Sport Injuries. Springer; 2016. p. 433–7. 33. Erdem Y, Gul D, Akpancar S. Comparison of Intraarticular Injections of Hyaluronic Acid versus Dextrose Applied with Periarticular Prolotherapy in the Treatment of Recreational Athletes with Knee Osteoarthritis. Turkish J Sport Med. 2020;55(1):6–13. 34. Kalkhoven J, Coutts AJ, Impellizzeri FM. 'Training load error' is not a more accurate term than 'overuse' injury. Br J Sports Med [Internet]. 2020 Aug 1;54(15):934 LP – 935. Available from: http://bjsm.bmj.com/content/54/15/934.abstract35. Odole AC, Agbomeji OT, Onyeso OKK, Ojo JO, Odunaiya NA. Perspectives of Nigerian Athletes About Physiotherapy Services in Sports Injury Management: Implications for Rehabilitation. J Sport Rehabil. 2021;1(aop):1–8. 36. Hendrika W, Reswari A. The effect of physiotherapy on pain improvement in patients with early knee osteoarthritis at RSU UKI. Int J Med Heal Res. 2021;7(6):52–9. 37. MacKay C, Hawker GA, Jaglal SB. How Do Physical Therapists Approach Management of People With Early Knee Osteoarthritis? A Qualitative Study. Phys Ther [Internet]. 2019;100(2):295–306. Available from: https://doi.org/10.1093/ptj/pzz16438. Hrazdira L, Pilecki Z, Pilecki G. Ultrasound guided application of HA in the soft tissues of the locomotor system in athletes–First experience. Krátká sdělení Botek, M, Stejskal, P, Klimešová, I Vliv normobarické hypoxie na Akt Auton Nerv systému Pilot Stud 191 Drbošalová, V, Stejskal, P, Klugar, M, Krejčíř, V, Hynková, O Charakt klientů komerčn. :205. 39. Ricci M, Micheloni GM, Berti M, Perusi F, Sambugaro E, Vecchini E, et al. Clinical comparison of oral administration and viscosupplementation of hyaluronic acid (HA) in early knee osteoarthritis. Musculoskelet Surg. 2017;101(1):45–9. 40. Wang C-T, Lin Y-T, Chiang B-L, Lin Y-H, Hou S-M. High molecular weight hyaluronic acid down-regulates the gene expression of osteoarthritis-associated cytokines and enzymes in fibroblast-like synoviocytes from patients with early osteoarthritis. Osteoarthr Cartil. 2006;14(12):1237–47. 41. Irrgang JJ, Anderson AF. Development and validation of health-related quality of life measures for the knee. Clin Orthop Relat Res. 2002;402:95–109. 42. Roos EM, Roos HP, Lohmander LS, Ekdahl C, Beynnon BD. Knee Injury and Osteoarthritis Outcome Score (KOOS)—development of a self-administered outcome measure. J Orthop Sport Phys Ther. 1998;28(2):88–96. 43. Perruccio A V, Canizares M, Hawker GA, Roos EM, Maillefert JF, Lohmander SL. Comparative Validity and Responsiveness of the HOOS-PS and KOOS-PS to the WOMAC Physical Function Subscale in Total Joint Replacement for Osteoarthritis. 44. Salavati M, Mazaheri M, Negahban H, Sohani SM, Ebrahimian MR, Ebrahimi I, et al. Validation of a Persian-version of Knee injury and Osteoarthritis Outcome Score (KOOS) in Iranians with knee injuries. Osteoarthr Cartil. 2008;16(10):1178–82. 45. Higgins LD, Taylor MK, Park D, Ghodadra N, Marchant M, Pietrobon R, et al. Reliability and validity of the International Knee Documentation Committee (IKDC) subjective knee form. Jt Bone Spine. 2007;74(6):594–9. 46. Kujala UM, Kaprio J, Sarno S. Osteoarthritis of weight bearing joints of lower limbs in former elite male athletes. Bmj. 1994;308(6923):231–4. 47. Salini V, Pantalone A, Vanni D. Arthroscopy and Sport Injuries. Arthrosc Sport Inj. 2016;433–7. 48. Broglio K. Randomization in clinical trials: Permuted blocks and stratification. JAMA - J Am Med Assoc. 2018;319(21):2223–4. 49. Emanuel EJ, Wendler D, Grady C. What makes clinical research ethical? JAMA. 2000 May;283(20):2701–11. )

[26.	de Sire A, Agostini F, Lippi L, Mangone M, Marchese S, Cisari C, et al. Oxygen-Ozone Therapy in the Rehabilitation Field: State of the Art on Mechanisms of Action, Safety and Effectiveness in Patients with Musculoskeletal Disorders. Biomolecules. 2021 Feb;11(3).](1. Patel DR, Villalobos A. Evaluation and management of knee pain in young athletes: overuse injuries of the knee. Transl Pediatr [Internet]. 2017 Jul;6(3):190–8. Available from: https://pubmed.ncbi.nlm.nih.gov/287950102. Nicolini AP, de Carvalho RT, Matsuda MM, Filho JS, Cohen M. Common injuries in athletes' knee: Experience of a specialized center. Acta Ortop Bras. 2014;22(3):127–31. 3. Tiu T, Craig Van Dien MD. Knee overuse disorders. 4. Fuller CW, Ekstrand J, Junge A, Andersen TE, Bahr R, Dvorak J, et al. Consensus statement on injury definitions and data collection procedures in studies  of football (soccer) injuries. Br J Sports Med. 2006 Mar;40(3):193–201. 5. Majewski M, Susanne H, Klaus S. Epidemiology of athletic knee injuries: A 10-year study. Knee. 2006 Jun;13(3):184–8. 6. Wilder RP, Sethi S. Overuse injuries: tendinopathies, stress fractures, compartment syndrome, and shin  splints. Clin Sports Med. 2004 Jan;23(1):55–81, vi. 7. Aicale R, Tarantino D, Maffulli N. Overuse injuries in sport: a comprehensive overview. J Orthop Surg Res. 2018 Dec;13(1):309. 8. Yang J, Tibbetts AS, Covassin T, Cheng G, Nayar S, Heiden E. Epidemiology of overuse and acute injuries among competitive collegiate athletes. J Athl Train. 2012;47(2):198–204. 9. Bernetti A, Mangone M, Paoloni M, Di Sante L, Murgia M, Santilli V. Corticosteroid and hyaluronic acid injection therapy in tennis elbow (lateral epicondylalgia). Med Sport. 2014;67(2):289–95. 10. Iolascon G, Gimigliano F, Moretti A, De Sire A, Migliore A, Brandi ML, et al. Early osteoarthritis: How to define, diagnose, and manage. A systematic review. Eur Geriatr Med. 2017;8(5–6):383–96. 11. Drawer S, Fuller CW. Propensity for osteoarthritis and lower limb joint pain in retired professional soccer players. Br J Sports Med. 2001;35(6):402–8. 12. Vannini F, Spalding T, Andriolo L, Berruto M, Denti M, Espregueira-Mendes J, et al. Sport and early osteoarthritis: the role of sport in aetiology, progression and treatment of knee osteoarthritis. Knee Surgery, Sport Traumatol Arthrosc. 2016;24(6):1786–96. 13. Fröhlich S, Pazeller S, Cherati AS, Müller E, Frey WO, Spörri J. Overuse injuries in the knee, back and hip of top elite female alpine skiers during  the off-season preparation period: prevalence, severity and their association with traumatic preinjuries and training load. BMJ open Sport Exerc Med. 2020;6(1):e000892. 14. Rincon GA, Vyas D, Zhou J, Fu FH, Oiestad BE, Holm I, et al. \“ Knee function and prevalence of knee osteoarthritis after anterior cruciate ligament reconstruction: a prospective study with 10 to 15 years of follow-up\”. Letter to the editor. Am J Sports Med. 2011;39(4):NP3-author. 15. Kellgren JH, Lawrence J. Radiological assessment of osteo-arthrosis. Ann Rheum Dis. 1957;16(4):494. 16. Altman R, Asch E, Bloch D, Bole G, Borenstein D, Brandt K, et al. Development of criteria for the classification and reporting of osteoarthritis: classification of osteoarthritis of the knee. Arthritis Rheum Off J Am Coll Rheumatol. 1986;29(8):1039–49. 17. Luyten FP, Denti M, Filardo G, Kon E, Engebretsen L. Definition and classification of early osteoarthritis of the knee. Knee Surgery, Sport Traumatol Arthrosc. 2012;20(3):401–6. 18. Hoemann C, Kandel R, Roberts S, Saris DBF, Creemers L, Mainil-Varlet P, et al. International Cartilage Repair Society (ICRS) Recommended Guidelines for  Histological Endpoints for Cartilage Repair Studies in Animal Models and Clinical Trials. Cartilage. 2011 Apr;2(2):153–72. 19. Hunter DJ, Lo GH, Gale D, Grainger AJ, Guermazi A, Conaghan PG. The reliability of a new scoring system for knee osteoarthritis MRI and the validity of bone marrow lesion assessment: BLOKS (Boston–Leeds Osteoarthritis Knee Score). Ann Rheum Dis. 2008;67(2):206–11. 20. Peterfy CG, Guermazi A, Zaim S, Tirman PFJ, Miaux Y, White D, et al. Whole-organ magnetic resonance imaging score (WORMS) of the knee in osteoarthritis. Osteoarthr Cartil. 2004;12(3):177–90. 21. Demange MK, Sisto M, Rodeo S. Future trends for unicompartmental arthritis of the knee: injectables & stem cells. Clin Sports Med. 2014;33(1):161–74. 22. Leslie M. Knee osteoarthritis management therapies. Pain Manag Nurs. 2000;1(2):51–7. 23. Paoloni M, Bernetti A, Belelli A, Brignoli O, Buoso S, Caputi AP, et al. Appropriateness of clinical and organizational criteria for intra-articular injection therapies in osteoarthritis: A Delphi method consensus initiative among experts in Italy. Ann Ist Super Sanita. 2015;51:131–8. 24. Sandmark H, Hogstedt C, Lewold S, Vingård E. Osteoarthrosis of the knee in men and women in association with overweight, smoking, and hormone therapy. Ann Rheum Dis. 1999;58(3):151–5. 25. Christensen BV, Iuhl IU, Vilbek H, Bülow H, Dreijer NC, Rasmussen HF. Acupuncture treatment of severe knee osteoarthrosis. A long‐term study. Acta Anaesthesiol Scand. 1992;36(6):519–25. 26. de Sire A, Agostini F, Lippi L, Mangone M, Marchese S, Cisari C, et al. Oxygen-Ozone Therapy in the Rehabilitation Field: State of the Art on Mechanisms of  Action, Safety and Effectiveness in Patients with Musculoskeletal Disorders. Biomolecules. 2021 Feb;11(3). 27. Fransen M, McCONNELL S. Land-based exercise for osteoarthritis of the knee: a metaanalysis of randomized controlled trials. J Rheumatol. 2009;36(6):1109–17. 28. Hiemstra LA, Kerslake S, Irving C. Anterior knee pain in the athlete. Clin Sports Med. 2014;33(3):437–59. 29. Filardo G, Kon E, Di Matteo B, Pelotti P, Di Martino A, Marcacci M. Platelet-rich plasma for the treatment of patellar tendinopathy: clinical and imaging findings at medium-term follow-up. Int Orthop [Internet]. 2013;37(8):1583–9. Available from: https://doi.org/10.1007/s00264-013-1972-830. Trojian TH, Concoff AL, Joy SM, Hatzenbuehler JR, Saulsberry WJ, Coleman CI. AMSSM Scientific Statement Concerning Viscosupplementation Injections for Knee  Osteoarthritis: Importance for Individual Patient Outcomes. Clin J Sport Med  Off J Can Acad  Sport Med. 2016 Jan;26(1):1–11. 31. Ammar TY, Pereira TAP, Mistura SLL, Kuhn A, Saggin JI, Lopes OV. Viscosupplementation for treating knee osteoarthrosis: review of the literature. Rev Bras Ortop. 2015;50:489–94. 32. Salini V, Pantalone A, Vanni D, Abate M. Viscosupplementation in Athletes. In: Arthroscopy and Sport Injuries. Springer; 2016. p. 433–7. 33. Erdem Y, Gul D, Akpancar S. Comparison of Intraarticular Injections of Hyaluronic Acid versus Dextrose Applied with Periarticular Prolotherapy in the Treatment of Recreational Athletes with Knee Osteoarthritis. Turkish J Sport Med. 2020;55(1):6–13. 34. Kalkhoven J, Coutts AJ, Impellizzeri FM. 'Training load error' is not a more accurate term than 'overuse' injury. Br J Sports Med [Internet]. 2020 Aug 1;54(15):934 LP – 935. Available from: http://bjsm.bmj.com/content/54/15/934.abstract35. Odole AC, Agbomeji OT, Onyeso OKK, Ojo JO, Odunaiya NA. Perspectives of Nigerian Athletes About Physiotherapy Services in Sports Injury Management: Implications for Rehabilitation. J Sport Rehabil. 2021;1(aop):1–8. 36. Hendrika W, Reswari A. The effect of physiotherapy on pain improvement in patients with early knee osteoarthritis at RSU UKI. Int J Med Heal Res. 2021;7(6):52–9. 37. MacKay C, Hawker GA, Jaglal SB. How Do Physical Therapists Approach Management of People With Early Knee Osteoarthritis? A Qualitative Study. Phys Ther [Internet]. 2019;100(2):295–306. Available from: https://doi.org/10.1093/ptj/pzz16438. Hrazdira L, Pilecki Z, Pilecki G. Ultrasound guided application of HA in the soft tissues of the locomotor system in athletes–First experience. Krátká sdělení Botek, M, Stejskal, P, Klimešová, I Vliv normobarické hypoxie na Akt Auton Nerv systému Pilot Stud 191 Drbošalová, V, Stejskal, P, Klugar, M, Krejčíř, V, Hynková, O Charakt klientů komerčn. :205. 39. Ricci M, Micheloni GM, Berti M, Perusi F, Sambugaro E, Vecchini E, et al. Clinical comparison of oral administration and viscosupplementation of hyaluronic acid (HA) in early knee osteoarthritis. Musculoskelet Surg. 2017;101(1):45–9. 40. Wang C-T, Lin Y-T, Chiang B-L, Lin Y-H, Hou S-M. High molecular weight hyaluronic acid down-regulates the gene expression of osteoarthritis-associated cytokines and enzymes in fibroblast-like synoviocytes from patients with early osteoarthritis. Osteoarthr Cartil. 2006;14(12):1237–47. 41. Irrgang JJ, Anderson AF. Development and validation of health-related quality of life measures for the knee. Clin Orthop Relat Res. 2002;402:95–109. 42. Roos EM, Roos HP, Lohmander LS, Ekdahl C, Beynnon BD. Knee Injury and Osteoarthritis Outcome Score (KOOS)—development of a self-administered outcome measure. J Orthop Sport Phys Ther. 1998;28(2):88–96. 43. Perruccio A V, Canizares M, Hawker GA, Roos EM, Maillefert JF, Lohmander SL. Comparative Validity and Responsiveness of the HOOS-PS and KOOS-PS to the WOMAC Physical Function Subscale in Total Joint Replacement for Osteoarthritis. 44. Salavati M, Mazaheri M, Negahban H, Sohani SM, Ebrahimian MR, Ebrahimi I, et al. Validation of a Persian-version of Knee injury and Osteoarthritis Outcome Score (KOOS) in Iranians with knee injuries. Osteoarthr Cartil. 2008;16(10):1178–82. 45. Higgins LD, Taylor MK, Park D, Ghodadra N, Marchant M, Pietrobon R, et al. Reliability and validity of the International Knee Documentation Committee (IKDC) subjective knee form. Jt Bone Spine. 2007;74(6):594–9. 46. Kujala UM, Kaprio J, Sarno S. Osteoarthritis of weight bearing joints of lower limbs in former elite male athletes. Bmj. 1994;308(6923):231–4. 47. Salini V, Pantalone A, Vanni D. Arthroscopy and Sport Injuries. Arthrosc Sport Inj. 2016;433–7. 48. Broglio K. Randomization in clinical trials: Permuted blocks and stratification. JAMA - J Am Med Assoc. 2018;319(21):2223–4. 49. Emanuel EJ, Wendler D, Grady C. What makes clinical research ethical? JAMA. 2000 May;283(20):2701–11. )

[27.	Fransen M, McCONNELL S. Land-based exercise for osteoarthritis of the knee: a metaanalysis of randomized controlled trials. J Rheumatol. 2009;36(6):1109–17.](1. Patel DR, Villalobos A. Evaluation and management of knee pain in young athletes: overuse injuries of the knee. Transl Pediatr [Internet]. 2017 Jul;6(3):190–8. Available from: https://pubmed.ncbi.nlm.nih.gov/287950102. Nicolini AP, de Carvalho RT, Matsuda MM, Filho JS, Cohen M. Common injuries in athletes' knee: Experience of a specialized center. Acta Ortop Bras. 2014;22(3):127–31. 3. Tiu T, Craig Van Dien MD. Knee overuse disorders. 4. Fuller CW, Ekstrand J, Junge A, Andersen TE, Bahr R, Dvorak J, et al. Consensus statement on injury definitions and data collection procedures in studies  of football (soccer) injuries. Br J Sports Med. 2006 Mar;40(3):193–201. 5. Majewski M, Susanne H, Klaus S. Epidemiology of athletic knee injuries: A 10-year study. Knee. 2006 Jun;13(3):184–8. 6. Wilder RP, Sethi S. Overuse injuries: tendinopathies, stress fractures, compartment syndrome, and shin  splints. Clin Sports Med. 2004 Jan;23(1):55–81, vi. 7. Aicale R, Tarantino D, Maffulli N. Overuse injuries in sport: a comprehensive overview. J Orthop Surg Res. 2018 Dec;13(1):309. 8. Yang J, Tibbetts AS, Covassin T, Cheng G, Nayar S, Heiden E. Epidemiology of overuse and acute injuries among competitive collegiate athletes. J Athl Train. 2012;47(2):198–204. 9. Bernetti A, Mangone M, Paoloni M, Di Sante L, Murgia M, Santilli V. Corticosteroid and hyaluronic acid injection therapy in tennis elbow (lateral epicondylalgia). Med Sport. 2014;67(2):289–95. 10. Iolascon G, Gimigliano F, Moretti A, De Sire A, Migliore A, Brandi ML, et al. Early osteoarthritis: How to define, diagnose, and manage. A systematic review. Eur Geriatr Med. 2017;8(5–6):383–96. 11. Drawer S, Fuller CW. Propensity for osteoarthritis and lower limb joint pain in retired professional soccer players. Br J Sports Med. 2001;35(6):402–8. 12. Vannini F, Spalding T, Andriolo L, Berruto M, Denti M, Espregueira-Mendes J, et al. Sport and early osteoarthritis: the role of sport in aetiology, progression and treatment of knee osteoarthritis. Knee Surgery, Sport Traumatol Arthrosc. 2016;24(6):1786–96. 13. Fröhlich S, Pazeller S, Cherati AS, Müller E, Frey WO, Spörri J. Overuse injuries in the knee, back and hip of top elite female alpine skiers during  the off-season preparation period: prevalence, severity and their association with traumatic preinjuries and training load. BMJ open Sport Exerc Med. 2020;6(1):e000892. 14. Rincon GA, Vyas D, Zhou J, Fu FH, Oiestad BE, Holm I, et al. \“ Knee function and prevalence of knee osteoarthritis after anterior cruciate ligament reconstruction: a prospective study with 10 to 15 years of follow-up\”. Letter to the editor. Am J Sports Med. 2011;39(4):NP3-author. 15. Kellgren JH, Lawrence J. Radiological assessment of osteo-arthrosis. Ann Rheum Dis. 1957;16(4):494. 16. Altman R, Asch E, Bloch D, Bole G, Borenstein D, Brandt K, et al. Development of criteria for the classification and reporting of osteoarthritis: classification of osteoarthritis of the knee. Arthritis Rheum Off J Am Coll Rheumatol. 1986;29(8):1039–49. 17. Luyten FP, Denti M, Filardo G, Kon E, Engebretsen L. Definition and classification of early osteoarthritis of the knee. Knee Surgery, Sport Traumatol Arthrosc. 2012;20(3):401–6. 18. Hoemann C, Kandel R, Roberts S, Saris DBF, Creemers L, Mainil-Varlet P, et al. International Cartilage Repair Society (ICRS) Recommended Guidelines for  Histological Endpoints for Cartilage Repair Studies in Animal Models and Clinical Trials. Cartilage. 2011 Apr;2(2):153–72. 19. Hunter DJ, Lo GH, Gale D, Grainger AJ, Guermazi A, Conaghan PG. The reliability of a new scoring system for knee osteoarthritis MRI and the validity of bone marrow lesion assessment: BLOKS (Boston–Leeds Osteoarthritis Knee Score). Ann Rheum Dis. 2008;67(2):206–11. 20. Peterfy CG, Guermazi A, Zaim S, Tirman PFJ, Miaux Y, White D, et al. Whole-organ magnetic resonance imaging score (WORMS) of the knee in osteoarthritis. Osteoarthr Cartil. 2004;12(3):177–90. 21. Demange MK, Sisto M, Rodeo S. Future trends for unicompartmental arthritis of the knee: injectables & stem cells. Clin Sports Med. 2014;33(1):161–74. 22. Leslie M. Knee osteoarthritis management therapies. Pain Manag Nurs. 2000;1(2):51–7. 23. Paoloni M, Bernetti A, Belelli A, Brignoli O, Buoso S, Caputi AP, et al. Appropriateness of clinical and organizational criteria for intra-articular injection therapies in osteoarthritis: A Delphi method consensus initiative among experts in Italy. Ann Ist Super Sanita. 2015;51:131–8. 24. Sandmark H, Hogstedt C, Lewold S, Vingård E. Osteoarthrosis of the knee in men and women in association with overweight, smoking, and hormone therapy. Ann Rheum Dis. 1999;58(3):151–5. 25. Christensen BV, Iuhl IU, Vilbek H, Bülow H, Dreijer NC, Rasmussen HF. Acupuncture treatment of severe knee osteoarthrosis. A long‐term study. Acta Anaesthesiol Scand. 1992;36(6):519–25. 26. de Sire A, Agostini F, Lippi L, Mangone M, Marchese S, Cisari C, et al. Oxygen-Ozone Therapy in the Rehabilitation Field: State of the Art on Mechanisms of  Action, Safety and Effectiveness in Patients with Musculoskeletal Disorders. Biomolecules. 2021 Feb;11(3). 27. Fransen M, McCONNELL S. Land-based exercise for osteoarthritis of the knee: a metaanalysis of randomized controlled trials. J Rheumatol. 2009;36(6):1109–17. 28. Hiemstra LA, Kerslake S, Irving C. Anterior knee pain in the athlete. Clin Sports Med. 2014;33(3):437–59. 29. Filardo G, Kon E, Di Matteo B, Pelotti P, Di Martino A, Marcacci M. Platelet-rich plasma for the treatment of patellar tendinopathy: clinical and imaging findings at medium-term follow-up. Int Orthop [Internet]. 2013;37(8):1583–9. Available from: https://doi.org/10.1007/s00264-013-1972-830. Trojian TH, Concoff AL, Joy SM, Hatzenbuehler JR, Saulsberry WJ, Coleman CI. AMSSM Scientific Statement Concerning Viscosupplementation Injections for Knee  Osteoarthritis: Importance for Individual Patient Outcomes. Clin J Sport Med  Off J Can Acad  Sport Med. 2016 Jan;26(1):1–11. 31. Ammar TY, Pereira TAP, Mistura SLL, Kuhn A, Saggin JI, Lopes OV. Viscosupplementation for treating knee osteoarthrosis: review of the literature. Rev Bras Ortop. 2015;50:489–94. 32. Salini V, Pantalone A, Vanni D, Abate M. Viscosupplementation in Athletes. In: Arthroscopy and Sport Injuries. Springer; 2016. p. 433–7. 33. Erdem Y, Gul D, Akpancar S. Comparison of Intraarticular Injections of Hyaluronic Acid versus Dextrose Applied with Periarticular Prolotherapy in the Treatment of Recreational Athletes with Knee Osteoarthritis. Turkish J Sport Med. 2020;55(1):6–13. 34. Kalkhoven J, Coutts AJ, Impellizzeri FM. 'Training load error' is not a more accurate term than 'overuse' injury. Br J Sports Med [Internet]. 2020 Aug 1;54(15):934 LP – 935. Available from: http://bjsm.bmj.com/content/54/15/934.abstract35. Odole AC, Agbomeji OT, Onyeso OKK, Ojo JO, Odunaiya NA. Perspectives of Nigerian Athletes About Physiotherapy Services in Sports Injury Management: Implications for Rehabilitation. J Sport Rehabil. 2021;1(aop):1–8. 36. Hendrika W, Reswari A. The effect of physiotherapy on pain improvement in patients with early knee osteoarthritis at RSU UKI. Int J Med Heal Res. 2021;7(6):52–9. 37. MacKay C, Hawker GA, Jaglal SB. How Do Physical Therapists Approach Management of People With Early Knee Osteoarthritis? A Qualitative Study. Phys Ther [Internet]. 2019;100(2):295–306. Available from: https://doi.org/10.1093/ptj/pzz16438. Hrazdira L, Pilecki Z, Pilecki G. Ultrasound guided application of HA in the soft tissues of the locomotor system in athletes–First experience. Krátká sdělení Botek, M, Stejskal, P, Klimešová, I Vliv normobarické hypoxie na Akt Auton Nerv systému Pilot Stud 191 Drbošalová, V, Stejskal, P, Klugar, M, Krejčíř, V, Hynková, O Charakt klientů komerčn. :205. 39. Ricci M, Micheloni GM, Berti M, Perusi F, Sambugaro E, Vecchini E, et al. Clinical comparison of oral administration and viscosupplementation of hyaluronic acid (HA) in early knee osteoarthritis. Musculoskelet Surg. 2017;101(1):45–9. 40. Wang C-T, Lin Y-T, Chiang B-L, Lin Y-H, Hou S-M. High molecular weight hyaluronic acid down-regulates the gene expression of osteoarthritis-associated cytokines and enzymes in fibroblast-like synoviocytes from patients with early osteoarthritis. Osteoarthr Cartil. 2006;14(12):1237–47. 41. Irrgang JJ, Anderson AF. Development and validation of health-related quality of life measures for the knee. Clin Orthop Relat Res. 2002;402:95–109. 42. Roos EM, Roos HP, Lohmander LS, Ekdahl C, Beynnon BD. Knee Injury and Osteoarthritis Outcome Score (KOOS)—development of a self-administered outcome measure. J Orthop Sport Phys Ther. 1998;28(2):88–96. 43. Perruccio A V, Canizares M, Hawker GA, Roos EM, Maillefert JF, Lohmander SL. Comparative Validity and Responsiveness of the HOOS-PS and KOOS-PS to the WOMAC Physical Function Subscale in Total Joint Replacement for Osteoarthritis. 44. Salavati M, Mazaheri M, Negahban H, Sohani SM, Ebrahimian MR, Ebrahimi I, et al. Validation of a Persian-version of Knee injury and Osteoarthritis Outcome Score (KOOS) in Iranians with knee injuries. Osteoarthr Cartil. 2008;16(10):1178–82. 45. Higgins LD, Taylor MK, Park D, Ghodadra N, Marchant M, Pietrobon R, et al. Reliability and validity of the International Knee Documentation Committee (IKDC) subjective knee form. Jt Bone Spine. 2007;74(6):594–9. 46. Kujala UM, Kaprio J, Sarno S. Osteoarthritis of weight bearing joints of lower limbs in former elite male athletes. Bmj. 1994;308(6923):231–4. 47. Salini V, Pantalone A, Vanni D. Arthroscopy and Sport Injuries. Arthrosc Sport Inj. 2016;433–7. 48. Broglio K. Randomization in clinical trials: Permuted blocks and stratification. JAMA - J Am Med Assoc. 2018;319(21):2223–4. 49. Emanuel EJ, Wendler D, Grady C. What makes clinical research ethical? JAMA. 2000 May;283(20):2701–11. )

[28.	Hiemstra LA, Kerslake S, Irving C. Anterior knee pain in the athlete. Clin Sports Med. 2014;33(3):437–59.](1. Patel DR, Villalobos A. Evaluation and management of knee pain in young athletes: overuse injuries of the knee. Transl Pediatr [Internet]. 2017 Jul;6(3):190–8. Available from: https://pubmed.ncbi.nlm.nih.gov/287950102. Nicolini AP, de Carvalho RT, Matsuda MM, Filho JS, Cohen M. Common injuries in athletes' knee: Experience of a specialized center. Acta Ortop Bras. 2014;22(3):127–31. 3. Tiu T, Craig Van Dien MD. Knee overuse disorders. 4. Fuller CW, Ekstrand J, Junge A, Andersen TE, Bahr R, Dvorak J, et al. Consensus statement on injury definitions and data collection procedures in studies  of football (soccer) injuries. Br J Sports Med. 2006 Mar;40(3):193–201. 5. Majewski M, Susanne H, Klaus S. Epidemiology of athletic knee injuries: A 10-year study. Knee. 2006 Jun;13(3):184–8. 6. Wilder RP, Sethi S. Overuse injuries: tendinopathies, stress fractures, compartment syndrome, and shin  splints. Clin Sports Med. 2004 Jan;23(1):55–81, vi. 7. Aicale R, Tarantino D, Maffulli N. Overuse injuries in sport: a comprehensive overview. J Orthop Surg Res. 2018 Dec;13(1):309. 8. Yang J, Tibbetts AS, Covassin T, Cheng G, Nayar S, Heiden E. Epidemiology of overuse and acute injuries among competitive collegiate athletes. J Athl Train. 2012;47(2):198–204. 9. Bernetti A, Mangone M, Paoloni M, Di Sante L, Murgia M, Santilli V. Corticosteroid and hyaluronic acid injection therapy in tennis elbow (lateral epicondylalgia). Med Sport. 2014;67(2):289–95. 10. Iolascon G, Gimigliano F, Moretti A, De Sire A, Migliore A, Brandi ML, et al. Early osteoarthritis: How to define, diagnose, and manage. A systematic review. Eur Geriatr Med. 2017;8(5–6):383–96. 11. Drawer S, Fuller CW. Propensity for osteoarthritis and lower limb joint pain in retired professional soccer players. Br J Sports Med. 2001;35(6):402–8. 12. Vannini F, Spalding T, Andriolo L, Berruto M, Denti M, Espregueira-Mendes J, et al. Sport and early osteoarthritis: the role of sport in aetiology, progression and treatment of knee osteoarthritis. Knee Surgery, Sport Traumatol Arthrosc. 2016;24(6):1786–96. 13. Fröhlich S, Pazeller S, Cherati AS, Müller E, Frey WO, Spörri J. Overuse injuries in the knee, back and hip of top elite female alpine skiers during  the off-season preparation period: prevalence, severity and their association with traumatic preinjuries and training load. BMJ open Sport Exerc Med. 2020;6(1):e000892. 14. Rincon GA, Vyas D, Zhou J, Fu FH, Oiestad BE, Holm I, et al. \“ Knee function and prevalence of knee osteoarthritis after anterior cruciate ligament reconstruction: a prospective study with 10 to 15 years of follow-up\”. Letter to the editor. Am J Sports Med. 2011;39(4):NP3-author. 15. Kellgren JH, Lawrence J. Radiological assessment of osteo-arthrosis. Ann Rheum Dis. 1957;16(4):494. 16. Altman R, Asch E, Bloch D, Bole G, Borenstein D, Brandt K, et al. Development of criteria for the classification and reporting of osteoarthritis: classification of osteoarthritis of the knee. Arthritis Rheum Off J Am Coll Rheumatol. 1986;29(8):1039–49. 17. Luyten FP, Denti M, Filardo G, Kon E, Engebretsen L. Definition and classification of early osteoarthritis of the knee. Knee Surgery, Sport Traumatol Arthrosc. 2012;20(3):401–6. 18. Hoemann C, Kandel R, Roberts S, Saris DBF, Creemers L, Mainil-Varlet P, et al. International Cartilage Repair Society (ICRS) Recommended Guidelines for  Histological Endpoints for Cartilage Repair Studies in Animal Models and Clinical Trials. Cartilage. 2011 Apr;2(2):153–72. 19. Hunter DJ, Lo GH, Gale D, Grainger AJ, Guermazi A, Conaghan PG. The reliability of a new scoring system for knee osteoarthritis MRI and the validity of bone marrow lesion assessment: BLOKS (Boston–Leeds Osteoarthritis Knee Score). Ann Rheum Dis. 2008;67(2):206–11. 20. Peterfy CG, Guermazi A, Zaim S, Tirman PFJ, Miaux Y, White D, et al. Whole-organ magnetic resonance imaging score (WORMS) of the knee in osteoarthritis. Osteoarthr Cartil. 2004;12(3):177–90. 21. Demange MK, Sisto M, Rodeo S. Future trends for unicompartmental arthritis of the knee: injectables & stem cells. Clin Sports Med. 2014;33(1):161–74. 22. Leslie M. Knee osteoarthritis management therapies. Pain Manag Nurs. 2000;1(2):51–7. 23. Paoloni M, Bernetti A, Belelli A, Brignoli O, Buoso S, Caputi AP, et al. Appropriateness of clinical and organizational criteria for intra-articular injection therapies in osteoarthritis: A Delphi method consensus initiative among experts in Italy. Ann Ist Super Sanita. 2015;51:131–8. 24. Sandmark H, Hogstedt C, Lewold S, Vingård E. Osteoarthrosis of the knee in men and women in association with overweight, smoking, and hormone therapy. Ann Rheum Dis. 1999;58(3):151–5. 25. Christensen BV, Iuhl IU, Vilbek H, Bülow H, Dreijer NC, Rasmussen HF. Acupuncture treatment of severe knee osteoarthrosis. A long‐term study. Acta Anaesthesiol Scand. 1992;36(6):519–25. 26. de Sire A, Agostini F, Lippi L, Mangone M, Marchese S, Cisari C, et al. Oxygen-Ozone Therapy in the Rehabilitation Field: State of the Art on Mechanisms of  Action, Safety and Effectiveness in Patients with Musculoskeletal Disorders. Biomolecules. 2021 Feb;11(3). 27. Fransen M, McCONNELL S. Land-based exercise for osteoarthritis of the knee: a metaanalysis of randomized controlled trials. J Rheumatol. 2009;36(6):1109–17. 28. Hiemstra LA, Kerslake S, Irving C. Anterior knee pain in the athlete. Clin Sports Med. 2014;33(3):437–59. 29. Filardo G, Kon E, Di Matteo B, Pelotti P, Di Martino A, Marcacci M. Platelet-rich plasma for the treatment of patellar tendinopathy: clinical and imaging findings at medium-term follow-up. Int Orthop [Internet]. 2013;37(8):1583–9. Available from: https://doi.org/10.1007/s00264-013-1972-830. Trojian TH, Concoff AL, Joy SM, Hatzenbuehler JR, Saulsberry WJ, Coleman CI. AMSSM Scientific Statement Concerning Viscosupplementation Injections for Knee  Osteoarthritis: Importance for Individual Patient Outcomes. Clin J Sport Med  Off J Can Acad  Sport Med. 2016 Jan;26(1):1–11. 31. Ammar TY, Pereira TAP, Mistura SLL, Kuhn A, Saggin JI, Lopes OV. Viscosupplementation for treating knee osteoarthrosis: review of the literature. Rev Bras Ortop. 2015;50:489–94. 32. Salini V, Pantalone A, Vanni D, Abate M. Viscosupplementation in Athletes. In: Arthroscopy and Sport Injuries. Springer; 2016. p. 433–7. 33. Erdem Y, Gul D, Akpancar S. Comparison of Intraarticular Injections of Hyaluronic Acid versus Dextrose Applied with Periarticular Prolotherapy in the Treatment of Recreational Athletes with Knee Osteoarthritis. Turkish J Sport Med. 2020;55(1):6–13. 34. Kalkhoven J, Coutts AJ, Impellizzeri FM. 'Training load error' is not a more accurate term than 'overuse' injury. Br J Sports Med [Internet]. 2020 Aug 1;54(15):934 LP – 935. Available from: http://bjsm.bmj.com/content/54/15/934.abstract35. Odole AC, Agbomeji OT, Onyeso OKK, Ojo JO, Odunaiya NA. Perspectives of Nigerian Athletes About Physiotherapy Services in Sports Injury Management: Implications for Rehabilitation. J Sport Rehabil. 2021;1(aop):1–8. 36. Hendrika W, Reswari A. The effect of physiotherapy on pain improvement in patients with early knee osteoarthritis at RSU UKI. Int J Med Heal Res. 2021;7(6):52–9. 37. MacKay C, Hawker GA, Jaglal SB. How Do Physical Therapists Approach Management of People With Early Knee Osteoarthritis? A Qualitative Study. Phys Ther [Internet]. 2019;100(2):295–306. Available from: https://doi.org/10.1093/ptj/pzz16438. Hrazdira L, Pilecki Z, Pilecki G. Ultrasound guided application of HA in the soft tissues of the locomotor system in athletes–First experience. Krátká sdělení Botek, M, Stejskal, P, Klimešová, I Vliv normobarické hypoxie na Akt Auton Nerv systému Pilot Stud 191 Drbošalová, V, Stejskal, P, Klugar, M, Krejčíř, V, Hynková, O Charakt klientů komerčn. :205. 39. Ricci M, Micheloni GM, Berti M, Perusi F, Sambugaro E, Vecchini E, et al. Clinical comparison of oral administration and viscosupplementation of hyaluronic acid (HA) in early knee osteoarthritis. Musculoskelet Surg. 2017;101(1):45–9. 40. Wang C-T, Lin Y-T, Chiang B-L, Lin Y-H, Hou S-M. High molecular weight hyaluronic acid down-regulates the gene expression of osteoarthritis-associated cytokines and enzymes in fibroblast-like synoviocytes from patients with early osteoarthritis. Osteoarthr Cartil. 2006;14(12):1237–47. 41. Irrgang JJ, Anderson AF. Development and validation of health-related quality of life measures for the knee. Clin Orthop Relat Res. 2002;402:95–109. 42. Roos EM, Roos HP, Lohmander LS, Ekdahl C, Beynnon BD. Knee Injury and Osteoarthritis Outcome Score (KOOS)—development of a self-administered outcome measure. J Orthop Sport Phys Ther. 1998;28(2):88–96. 43. Perruccio A V, Canizares M, Hawker GA, Roos EM, Maillefert JF, Lohmander SL. Comparative Validity and Responsiveness of the HOOS-PS and KOOS-PS to the WOMAC Physical Function Subscale in Total Joint Replacement for Osteoarthritis. 44. Salavati M, Mazaheri M, Negahban H, Sohani SM, Ebrahimian MR, Ebrahimi I, et al. Validation of a Persian-version of Knee injury and Osteoarthritis Outcome Score (KOOS) in Iranians with knee injuries. Osteoarthr Cartil. 2008;16(10):1178–82. 45. Higgins LD, Taylor MK, Park D, Ghodadra N, Marchant M, Pietrobon R, et al. Reliability and validity of the International Knee Documentation Committee (IKDC) subjective knee form. Jt Bone Spine. 2007;74(6):594–9. 46. Kujala UM, Kaprio J, Sarno S. Osteoarthritis of weight bearing joints of lower limbs in former elite male athletes. Bmj. 1994;308(6923):231–4. 47. Salini V, Pantalone A, Vanni D. Arthroscopy and Sport Injuries. Arthrosc Sport Inj. 2016;433–7. 48. Broglio K. Randomization in clinical trials: Permuted blocks and stratification. JAMA - J Am Med Assoc. 2018;319(21):2223–4. 49. Emanuel EJ, Wendler D, Grady C. What makes clinical research ethical? JAMA. 2000 May;283(20):2701–11. )

[29.	Filardo G, Kon E, Di Matteo B, Pelotti P, Di Martino A, Marcacci M. Platelet-rich plasma for the treatment of patellar tendinopathy: clinical and imaging findings at medium-term follow-up. Int Orthop [Internet]. 2013;37(8):1583–9. Available from: https://doi.org/10.1007/s00264-013-1972-8](1. Patel DR, Villalobos A. Evaluation and management of knee pain in young athletes: overuse injuries of the knee. Transl Pediatr [Internet]. 2017 Jul;6(3):190–8. Available from: https://pubmed.ncbi.nlm.nih.gov/287950102. Nicolini AP, de Carvalho RT, Matsuda MM, Filho JS, Cohen M. Common injuries in athletes' knee: Experience of a specialized center. Acta Ortop Bras. 2014;22(3):127–31. 3. Tiu T, Craig Van Dien MD. Knee overuse disorders. 4. Fuller CW, Ekstrand J, Junge A, Andersen TE, Bahr R, Dvorak J, et al. Consensus statement on injury definitions and data collection procedures in studies  of football (soccer) injuries. Br J Sports Med. 2006 Mar;40(3):193–201. 5. Majewski M, Susanne H, Klaus S. Epidemiology of athletic knee injuries: A 10-year study. Knee. 2006 Jun;13(3):184–8. 6. Wilder RP, Sethi S. Overuse injuries: tendinopathies, stress fractures, compartment syndrome, and shin  splints. Clin Sports Med. 2004 Jan;23(1):55–81, vi. 7. Aicale R, Tarantino D, Maffulli N. Overuse injuries in sport: a comprehensive overview. J Orthop Surg Res. 2018 Dec;13(1):309. 8. Yang J, Tibbetts AS, Covassin T, Cheng G, Nayar S, Heiden E. Epidemiology of overuse and acute injuries among competitive collegiate athletes. J Athl Train. 2012;47(2):198–204. 9. Bernetti A, Mangone M, Paoloni M, Di Sante L, Murgia M, Santilli V. Corticosteroid and hyaluronic acid injection therapy in tennis elbow (lateral epicondylalgia). Med Sport. 2014;67(2):289–95. 10. Iolascon G, Gimigliano F, Moretti A, De Sire A, Migliore A, Brandi ML, et al. Early osteoarthritis: How to define, diagnose, and manage. A systematic review. Eur Geriatr Med. 2017;8(5–6):383–96. 11. Drawer S, Fuller CW. Propensity for osteoarthritis and lower limb joint pain in retired professional soccer players. Br J Sports Med. 2001;35(6):402–8. 12. Vannini F, Spalding T, Andriolo L, Berruto M, Denti M, Espregueira-Mendes J, et al. Sport and early osteoarthritis: the role of sport in aetiology, progression and treatment of knee osteoarthritis. Knee Surgery, Sport Traumatol Arthrosc. 2016;24(6):1786–96. 13. Fröhlich S, Pazeller S, Cherati AS, Müller E, Frey WO, Spörri J. Overuse injuries in the knee, back and hip of top elite female alpine skiers during  the off-season preparation period: prevalence, severity and their association with traumatic preinjuries and training load. BMJ open Sport Exerc Med. 2020;6(1):e000892. 14. Rincon GA, Vyas D, Zhou J, Fu FH, Oiestad BE, Holm I, et al. \“ Knee function and prevalence of knee osteoarthritis after anterior cruciate ligament reconstruction: a prospective study with 10 to 15 years of follow-up\”. Letter to the editor. Am J Sports Med. 2011;39(4):NP3-author. 15. Kellgren JH, Lawrence J. Radiological assessment of osteo-arthrosis. Ann Rheum Dis. 1957;16(4):494. 16. Altman R, Asch E, Bloch D, Bole G, Borenstein D, Brandt K, et al. Development of criteria for the classification and reporting of osteoarthritis: classification of osteoarthritis of the knee. Arthritis Rheum Off J Am Coll Rheumatol. 1986;29(8):1039–49. 17. Luyten FP, Denti M, Filardo G, Kon E, Engebretsen L. Definition and classification of early osteoarthritis of the knee. Knee Surgery, Sport Traumatol Arthrosc. 2012;20(3):401–6. 18. Hoemann C, Kandel R, Roberts S, Saris DBF, Creemers L, Mainil-Varlet P, et al. International Cartilage Repair Society (ICRS) Recommended Guidelines for  Histological Endpoints for Cartilage Repair Studies in Animal Models and Clinical Trials. Cartilage. 2011 Apr;2(2):153–72. 19. Hunter DJ, Lo GH, Gale D, Grainger AJ, Guermazi A, Conaghan PG. The reliability of a new scoring system for knee osteoarthritis MRI and the validity of bone marrow lesion assessment: BLOKS (Boston–Leeds Osteoarthritis Knee Score). Ann Rheum Dis. 2008;67(2):206–11. 20. Peterfy CG, Guermazi A, Zaim S, Tirman PFJ, Miaux Y, White D, et al. Whole-organ magnetic resonance imaging score (WORMS) of the knee in osteoarthritis. Osteoarthr Cartil. 2004;12(3):177–90. 21. Demange MK, Sisto M, Rodeo S. Future trends for unicompartmental arthritis of the knee: injectables & stem cells. Clin Sports Med. 2014;33(1):161–74. 22. Leslie M. Knee osteoarthritis management therapies. Pain Manag Nurs. 2000;1(2):51–7. 23. Paoloni M, Bernetti A, Belelli A, Brignoli O, Buoso S, Caputi AP, et al. Appropriateness of clinical and organizational criteria for intra-articular injection therapies in osteoarthritis: A Delphi method consensus initiative among experts in Italy. Ann Ist Super Sanita. 2015;51:131–8. 24. Sandmark H, Hogstedt C, Lewold S, Vingård E. Osteoarthrosis of the knee in men and women in association with overweight, smoking, and hormone therapy. Ann Rheum Dis. 1999;58(3):151–5. 25. Christensen BV, Iuhl IU, Vilbek H, Bülow H, Dreijer NC, Rasmussen HF. Acupuncture treatment of severe knee osteoarthrosis. A long‐term study. Acta Anaesthesiol Scand. 1992;36(6):519–25. 26. de Sire A, Agostini F, Lippi L, Mangone M, Marchese S, Cisari C, et al. Oxygen-Ozone Therapy in the Rehabilitation Field: State of the Art on Mechanisms of  Action, Safety and Effectiveness in Patients with Musculoskeletal Disorders. Biomolecules. 2021 Feb;11(3). 27. Fransen M, McCONNELL S. Land-based exercise for osteoarthritis of the knee: a metaanalysis of randomized controlled trials. J Rheumatol. 2009;36(6):1109–17. 28. Hiemstra LA, Kerslake S, Irving C. Anterior knee pain in the athlete. Clin Sports Med. 2014;33(3):437–59. 29. Filardo G, Kon E, Di Matteo B, Pelotti P, Di Martino A, Marcacci M. Platelet-rich plasma for the treatment of patellar tendinopathy: clinical and imaging findings at medium-term follow-up. Int Orthop [Internet]. 2013;37(8):1583–9. Available from: https://doi.org/10.1007/s00264-013-1972-830. Trojian TH, Concoff AL, Joy SM, Hatzenbuehler JR, Saulsberry WJ, Coleman CI. AMSSM Scientific Statement Concerning Viscosupplementation Injections for Knee  Osteoarthritis: Importance for Individual Patient Outcomes. Clin J Sport Med  Off J Can Acad  Sport Med. 2016 Jan;26(1):1–11. 31. Ammar TY, Pereira TAP, Mistura SLL, Kuhn A, Saggin JI, Lopes OV. Viscosupplementation for treating knee osteoarthrosis: review of the literature. Rev Bras Ortop. 2015;50:489–94. 32. Salini V, Pantalone A, Vanni D, Abate M. Viscosupplementation in Athletes. In: Arthroscopy and Sport Injuries. Springer; 2016. p. 433–7. 33. Erdem Y, Gul D, Akpancar S. Comparison of Intraarticular Injections of Hyaluronic Acid versus Dextrose Applied with Periarticular Prolotherapy in the Treatment of Recreational Athletes with Knee Osteoarthritis. Turkish J Sport Med. 2020;55(1):6–13. 34. Kalkhoven J, Coutts AJ, Impellizzeri FM. 'Training load error' is not a more accurate term than 'overuse' injury. Br J Sports Med [Internet]. 2020 Aug 1;54(15):934 LP – 935. Available from: http://bjsm.bmj.com/content/54/15/934.abstract35. Odole AC, Agbomeji OT, Onyeso OKK, Ojo JO, Odunaiya NA. Perspectives of Nigerian Athletes About Physiotherapy Services in Sports Injury Management: Implications for Rehabilitation. J Sport Rehabil. 2021;1(aop):1–8. 36. Hendrika W, Reswari A. The effect of physiotherapy on pain improvement in patients with early knee osteoarthritis at RSU UKI. Int J Med Heal Res. 2021;7(6):52–9. 37. MacKay C, Hawker GA, Jaglal SB. How Do Physical Therapists Approach Management of People With Early Knee Osteoarthritis? A Qualitative Study. Phys Ther [Internet]. 2019;100(2):295–306. Available from: https://doi.org/10.1093/ptj/pzz16438. Hrazdira L, Pilecki Z, Pilecki G. Ultrasound guided application of HA in the soft tissues of the locomotor system in athletes–First experience. Krátká sdělení Botek, M, Stejskal, P, Klimešová, I Vliv normobarické hypoxie na Akt Auton Nerv systému Pilot Stud 191 Drbošalová, V, Stejskal, P, Klugar, M, Krejčíř, V, Hynková, O Charakt klientů komerčn. :205. 39. Ricci M, Micheloni GM, Berti M, Perusi F, Sambugaro E, Vecchini E, et al. Clinical comparison of oral administration and viscosupplementation of hyaluronic acid (HA) in early knee osteoarthritis. Musculoskelet Surg. 2017;101(1):45–9. 40. Wang C-T, Lin Y-T, Chiang B-L, Lin Y-H, Hou S-M. High molecular weight hyaluronic acid down-regulates the gene expression of osteoarthritis-associated cytokines and enzymes in fibroblast-like synoviocytes from patients with early osteoarthritis. Osteoarthr Cartil. 2006;14(12):1237–47. 41. Irrgang JJ, Anderson AF. Development and validation of health-related quality of life measures for the knee. Clin Orthop Relat Res. 2002;402:95–109. 42. Roos EM, Roos HP, Lohmander LS, Ekdahl C, Beynnon BD. Knee Injury and Osteoarthritis Outcome Score (KOOS)—development of a self-administered outcome measure. J Orthop Sport Phys Ther. 1998;28(2):88–96. 43. Perruccio A V, Canizares M, Hawker GA, Roos EM, Maillefert JF, Lohmander SL. Comparative Validity and Responsiveness of the HOOS-PS and KOOS-PS to the WOMAC Physical Function Subscale in Total Joint Replacement for Osteoarthritis. 44. Salavati M, Mazaheri M, Negahban H, Sohani SM, Ebrahimian MR, Ebrahimi I, et al. Validation of a Persian-version of Knee injury and Osteoarthritis Outcome Score (KOOS) in Iranians with knee injuries. Osteoarthr Cartil. 2008;16(10):1178–82. 45. Higgins LD, Taylor MK, Park D, Ghodadra N, Marchant M, Pietrobon R, et al. Reliability and validity of the International Knee Documentation Committee (IKDC) subjective knee form. Jt Bone Spine. 2007;74(6):594–9. 46. Kujala UM, Kaprio J, Sarno S. Osteoarthritis of weight bearing joints of lower limbs in former elite male athletes. Bmj. 1994;308(6923):231–4. 47. Salini V, Pantalone A, Vanni D. Arthroscopy and Sport Injuries. Arthrosc Sport Inj. 2016;433–7. 48. Broglio K. Randomization in clinical trials: Permuted blocks and stratification. JAMA - J Am Med Assoc. 2018;319(21):2223–4. 49. Emanuel EJ, Wendler D, Grady C. What makes clinical research ethical? JAMA. 2000 May;283(20):2701–11. )

[30.	Trojian TH, Concoff AL, Joy SM, Hatzenbuehler JR, Saulsberry WJ, Coleman CI. AMSSM Scientific Statement Concerning Viscosupplementation Injections for Knee Osteoarthritis: Importance for Individual Patient Outcomes. Clin J Sport Med Off J Can Acad Sport Med. 2016 Jan;26(1):1–11.](1. Patel DR, Villalobos A. Evaluation and management of knee pain in young athletes: overuse injuries of the knee. Transl Pediatr [Internet]. 2017 Jul;6(3):190–8. Available from: https://pubmed.ncbi.nlm.nih.gov/287950102. Nicolini AP, de Carvalho RT, Matsuda MM, Filho JS, Cohen M. Common injuries in athletes' knee: Experience of a specialized center. Acta Ortop Bras. 2014;22(3):127–31. 3. Tiu T, Craig Van Dien MD. Knee overuse disorders. 4. Fuller CW, Ekstrand J, Junge A, Andersen TE, Bahr R, Dvorak J, et al. Consensus statement on injury definitions and data collection procedures in studies  of football (soccer) injuries. Br J Sports Med. 2006 Mar;40(3):193–201. 5. Majewski M, Susanne H, Klaus S. Epidemiology of athletic knee injuries: A 10-year study. Knee. 2006 Jun;13(3):184–8. 6. Wilder RP, Sethi S. Overuse injuries: tendinopathies, stress fractures, compartment syndrome, and shin  splints. Clin Sports Med. 2004 Jan;23(1):55–81, vi. 7. Aicale R, Tarantino D, Maffulli N. Overuse injuries in sport: a comprehensive overview. J Orthop Surg Res. 2018 Dec;13(1):309. 8. Yang J, Tibbetts AS, Covassin T, Cheng G, Nayar S, Heiden E. Epidemiology of overuse and acute injuries among competitive collegiate athletes. J Athl Train. 2012;47(2):198–204. 9. Bernetti A, Mangone M, Paoloni M, Di Sante L, Murgia M, Santilli V. Corticosteroid and hyaluronic acid injection therapy in tennis elbow (lateral epicondylalgia). Med Sport. 2014;67(2):289–95. 10. Iolascon G, Gimigliano F, Moretti A, De Sire A, Migliore A, Brandi ML, et al. Early osteoarthritis: How to define, diagnose, and manage. A systematic review. Eur Geriatr Med. 2017;8(5–6):383–96. 11. Drawer S, Fuller CW. Propensity for osteoarthritis and lower limb joint pain in retired professional soccer players. Br J Sports Med. 2001;35(6):402–8. 12. Vannini F, Spalding T, Andriolo L, Berruto M, Denti M, Espregueira-Mendes J, et al. Sport and early osteoarthritis: the role of sport in aetiology, progression and treatment of knee osteoarthritis. Knee Surgery, Sport Traumatol Arthrosc. 2016;24(6):1786–96. 13. Fröhlich S, Pazeller S, Cherati AS, Müller E, Frey WO, Spörri J. Overuse injuries in the knee, back and hip of top elite female alpine skiers during  the off-season preparation period: prevalence, severity and their association with traumatic preinjuries and training load. BMJ open Sport Exerc Med. 2020;6(1):e000892. 14. Rincon GA, Vyas D, Zhou J, Fu FH, Oiestad BE, Holm I, et al. \“ Knee function and prevalence of knee osteoarthritis after anterior cruciate ligament reconstruction: a prospective study with 10 to 15 years of follow-up\”. Letter to the editor. Am J Sports Med. 2011;39(4):NP3-author. 15. Kellgren JH, Lawrence J. Radiological assessment of osteo-arthrosis. Ann Rheum Dis. 1957;16(4):494. 16. Altman R, Asch E, Bloch D, Bole G, Borenstein D, Brandt K, et al. Development of criteria for the classification and reporting of osteoarthritis: classification of osteoarthritis of the knee. Arthritis Rheum Off J Am Coll Rheumatol. 1986;29(8):1039–49. 17. Luyten FP, Denti M, Filardo G, Kon E, Engebretsen L. Definition and classification of early osteoarthritis of the knee. Knee Surgery, Sport Traumatol Arthrosc. 2012;20(3):401–6. 18. Hoemann C, Kandel R, Roberts S, Saris DBF, Creemers L, Mainil-Varlet P, et al. International Cartilage Repair Society (ICRS) Recommended Guidelines for  Histological Endpoints for Cartilage Repair Studies in Animal Models and Clinical Trials. Cartilage. 2011 Apr;2(2):153–72. 19. Hunter DJ, Lo GH, Gale D, Grainger AJ, Guermazi A, Conaghan PG. The reliability of a new scoring system for knee osteoarthritis MRI and the validity of bone marrow lesion assessment: BLOKS (Boston–Leeds Osteoarthritis Knee Score). Ann Rheum Dis. 2008;67(2):206–11. 20. Peterfy CG, Guermazi A, Zaim S, Tirman PFJ, Miaux Y, White D, et al. Whole-organ magnetic resonance imaging score (WORMS) of the knee in osteoarthritis. Osteoarthr Cartil. 2004;12(3):177–90. 21. Demange MK, Sisto M, Rodeo S. Future trends for unicompartmental arthritis of the knee: injectables & stem cells. Clin Sports Med. 2014;33(1):161–74. 22. Leslie M. Knee osteoarthritis management therapies. Pain Manag Nurs. 2000;1(2):51–7. 23. Paoloni M, Bernetti A, Belelli A, Brignoli O, Buoso S, Caputi AP, et al. Appropriateness of clinical and organizational criteria for intra-articular injection therapies in osteoarthritis: A Delphi method consensus initiative among experts in Italy. Ann Ist Super Sanita. 2015;51:131–8. 24. Sandmark H, Hogstedt C, Lewold S, Vingård E. Osteoarthrosis of the knee in men and women in association with overweight, smoking, and hormone therapy. Ann Rheum Dis. 1999;58(3):151–5. 25. Christensen BV, Iuhl IU, Vilbek H, Bülow H, Dreijer NC, Rasmussen HF. Acupuncture treatment of severe knee osteoarthrosis. A long‐term study. Acta Anaesthesiol Scand. 1992;36(6):519–25. 26. de Sire A, Agostini F, Lippi L, Mangone M, Marchese S, Cisari C, et al. Oxygen-Ozone Therapy in the Rehabilitation Field: State of the Art on Mechanisms of  Action, Safety and Effectiveness in Patients with Musculoskeletal Disorders. Biomolecules. 2021 Feb;11(3). 27. Fransen M, McCONNELL S. Land-based exercise for osteoarthritis of the knee: a metaanalysis of randomized controlled trials. J Rheumatol. 2009;36(6):1109–17. 28. Hiemstra LA, Kerslake S, Irving C. Anterior knee pain in the athlete. Clin Sports Med. 2014;33(3):437–59. 29. Filardo G, Kon E, Di Matteo B, Pelotti P, Di Martino A, Marcacci M. Platelet-rich plasma for the treatment of patellar tendinopathy: clinical and imaging findings at medium-term follow-up. Int Orthop [Internet]. 2013;37(8):1583–9. Available from: https://doi.org/10.1007/s00264-013-1972-830. Trojian TH, Concoff AL, Joy SM, Hatzenbuehler JR, Saulsberry WJ, Coleman CI. AMSSM Scientific Statement Concerning Viscosupplementation Injections for Knee  Osteoarthritis: Importance for Individual Patient Outcomes. Clin J Sport Med  Off J Can Acad  Sport Med. 2016 Jan;26(1):1–11. 31. Ammar TY, Pereira TAP, Mistura SLL, Kuhn A, Saggin JI, Lopes OV. Viscosupplementation for treating knee osteoarthrosis: review of the literature. Rev Bras Ortop. 2015;50:489–94. 32. Salini V, Pantalone A, Vanni D, Abate M. Viscosupplementation in Athletes. In: Arthroscopy and Sport Injuries. Springer; 2016. p. 433–7. 33. Erdem Y, Gul D, Akpancar S. Comparison of Intraarticular Injections of Hyaluronic Acid versus Dextrose Applied with Periarticular Prolotherapy in the Treatment of Recreational Athletes with Knee Osteoarthritis. Turkish J Sport Med. 2020;55(1):6–13. 34. Kalkhoven J, Coutts AJ, Impellizzeri FM. 'Training load error' is not a more accurate term than 'overuse' injury. Br J Sports Med [Internet]. 2020 Aug 1;54(15):934 LP – 935. Available from: http://bjsm.bmj.com/content/54/15/934.abstract35. Odole AC, Agbomeji OT, Onyeso OKK, Ojo JO, Odunaiya NA. Perspectives of Nigerian Athletes About Physiotherapy Services in Sports Injury Management: Implications for Rehabilitation. J Sport Rehabil. 2021;1(aop):1–8. 36. Hendrika W, Reswari A. The effect of physiotherapy on pain improvement in patients with early knee osteoarthritis at RSU UKI. Int J Med Heal Res. 2021;7(6):52–9. 37. MacKay C, Hawker GA, Jaglal SB. How Do Physical Therapists Approach Management of People With Early Knee Osteoarthritis? A Qualitative Study. Phys Ther [Internet]. 2019;100(2):295–306. Available from: https://doi.org/10.1093/ptj/pzz16438. Hrazdira L, Pilecki Z, Pilecki G. Ultrasound guided application of HA in the soft tissues of the locomotor system in athletes–First experience. Krátká sdělení Botek, M, Stejskal, P, Klimešová, I Vliv normobarické hypoxie na Akt Auton Nerv systému Pilot Stud 191 Drbošalová, V, Stejskal, P, Klugar, M, Krejčíř, V, Hynková, O Charakt klientů komerčn. :205. 39. Ricci M, Micheloni GM, Berti M, Perusi F, Sambugaro E, Vecchini E, et al. Clinical comparison of oral administration and viscosupplementation of hyaluronic acid (HA) in early knee osteoarthritis. Musculoskelet Surg. 2017;101(1):45–9. 40. Wang C-T, Lin Y-T, Chiang B-L, Lin Y-H, Hou S-M. High molecular weight hyaluronic acid down-regulates the gene expression of osteoarthritis-associated cytokines and enzymes in fibroblast-like synoviocytes from patients with early osteoarthritis. Osteoarthr Cartil. 2006;14(12):1237–47. 41. Irrgang JJ, Anderson AF. Development and validation of health-related quality of life measures for the knee. Clin Orthop Relat Res. 2002;402:95–109. 42. Roos EM, Roos HP, Lohmander LS, Ekdahl C, Beynnon BD. Knee Injury and Osteoarthritis Outcome Score (KOOS)—development of a self-administered outcome measure. J Orthop Sport Phys Ther. 1998;28(2):88–96. 43. Perruccio A V, Canizares M, Hawker GA, Roos EM, Maillefert JF, Lohmander SL. Comparative Validity and Responsiveness of the HOOS-PS and KOOS-PS to the WOMAC Physical Function Subscale in Total Joint Replacement for Osteoarthritis. 44. Salavati M, Mazaheri M, Negahban H, Sohani SM, Ebrahimian MR, Ebrahimi I, et al. Validation of a Persian-version of Knee injury and Osteoarthritis Outcome Score (KOOS) in Iranians with knee injuries. Osteoarthr Cartil. 2008;16(10):1178–82. 45. Higgins LD, Taylor MK, Park D, Ghodadra N, Marchant M, Pietrobon R, et al. Reliability and validity of the International Knee Documentation Committee (IKDC) subjective knee form. Jt Bone Spine. 2007;74(6):594–9. 46. Kujala UM, Kaprio J, Sarno S. Osteoarthritis of weight bearing joints of lower limbs in former elite male athletes. Bmj. 1994;308(6923):231–4. 47. Salini V, Pantalone A, Vanni D. Arthroscopy and Sport Injuries. Arthrosc Sport Inj. 2016;433–7. 48. Broglio K. Randomization in clinical trials: Permuted blocks and stratification. JAMA - J Am Med Assoc. 2018;319(21):2223–4. 49. Emanuel EJ, Wendler D, Grady C. What makes clinical research ethical? JAMA. 2000 May;283(20):2701–11. )

[31.	Ammar TY, Pereira TAP, Mistura SLL, Kuhn A, Saggin JI, Lopes OV. Viscosupplementation for treating knee osteoarthrosis: review of the literature. Rev Bras Ortop. 2015;50:489–94.](1. Patel DR, Villalobos A. Evaluation and management of knee pain in young athletes: overuse injuries of the knee. Transl Pediatr [Internet]. 2017 Jul;6(3):190–8. Available from: https://pubmed.ncbi.nlm.nih.gov/287950102. Nicolini AP, de Carvalho RT, Matsuda MM, Filho JS, Cohen M. Common injuries in athletes' knee: Experience of a specialized center. Acta Ortop Bras. 2014;22(3):127–31. 3. Tiu T, Craig Van Dien MD. Knee overuse disorders. 4. Fuller CW, Ekstrand J, Junge A, Andersen TE, Bahr R, Dvorak J, et al. Consensus statement on injury definitions and data collection procedures in studies  of football (soccer) injuries. Br J Sports Med. 2006 Mar;40(3):193–201. 5. Majewski M, Susanne H, Klaus S. Epidemiology of athletic knee injuries: A 10-year study. Knee. 2006 Jun;13(3):184–8. 6. Wilder RP, Sethi S. Overuse injuries: tendinopathies, stress fractures, compartment syndrome, and shin  splints. Clin Sports Med. 2004 Jan;23(1):55–81, vi. 7. Aicale R, Tarantino D, Maffulli N. Overuse injuries in sport: a comprehensive overview. J Orthop Surg Res. 2018 Dec;13(1):309. 8. Yang J, Tibbetts AS, Covassin T, Cheng G, Nayar S, Heiden E. Epidemiology of overuse and acute injuries among competitive collegiate athletes. J Athl Train. 2012;47(2):198–204. 9. Bernetti A, Mangone M, Paoloni M, Di Sante L, Murgia M, Santilli V. Corticosteroid and hyaluronic acid injection therapy in tennis elbow (lateral epicondylalgia). Med Sport. 2014;67(2):289–95. 10. Iolascon G, Gimigliano F, Moretti A, De Sire A, Migliore A, Brandi ML, et al. Early osteoarthritis: How to define, diagnose, and manage. A systematic review. Eur Geriatr Med. 2017;8(5–6):383–96. 11. Drawer S, Fuller CW. Propensity for osteoarthritis and lower limb joint pain in retired professional soccer players. Br J Sports Med. 2001;35(6):402–8. 12. Vannini F, Spalding T, Andriolo L, Berruto M, Denti M, Espregueira-Mendes J, et al. Sport and early osteoarthritis: the role of sport in aetiology, progression and treatment of knee osteoarthritis. Knee Surgery, Sport Traumatol Arthrosc. 2016;24(6):1786–96. 13. Fröhlich S, Pazeller S, Cherati AS, Müller E, Frey WO, Spörri J. Overuse injuries in the knee, back and hip of top elite female alpine skiers during  the off-season preparation period: prevalence, severity and their association with traumatic preinjuries and training load. BMJ open Sport Exerc Med. 2020;6(1):e000892. 14. Rincon GA, Vyas D, Zhou J, Fu FH, Oiestad BE, Holm I, et al. \“ Knee function and prevalence of knee osteoarthritis after anterior cruciate ligament reconstruction: a prospective study with 10 to 15 years of follow-up\”. Letter to the editor. Am J Sports Med. 2011;39(4):NP3-author. 15. Kellgren JH, Lawrence J. Radiological assessment of osteo-arthrosis. Ann Rheum Dis. 1957;16(4):494. 16. Altman R, Asch E, Bloch D, Bole G, Borenstein D, Brandt K, et al. Development of criteria for the classification and reporting of osteoarthritis: classification of osteoarthritis of the knee. Arthritis Rheum Off J Am Coll Rheumatol. 1986;29(8):1039–49. 17. Luyten FP, Denti M, Filardo G, Kon E, Engebretsen L. Definition and classification of early osteoarthritis of the knee. Knee Surgery, Sport Traumatol Arthrosc. 2012;20(3):401–6. 18. Hoemann C, Kandel R, Roberts S, Saris DBF, Creemers L, Mainil-Varlet P, et al. International Cartilage Repair Society (ICRS) Recommended Guidelines for  Histological Endpoints for Cartilage Repair Studies in Animal Models and Clinical Trials. Cartilage. 2011 Apr;2(2):153–72. 19. Hunter DJ, Lo GH, Gale D, Grainger AJ, Guermazi A, Conaghan PG. The reliability of a new scoring system for knee osteoarthritis MRI and the validity of bone marrow lesion assessment: BLOKS (Boston–Leeds Osteoarthritis Knee Score). Ann Rheum Dis. 2008;67(2):206–11. 20. Peterfy CG, Guermazi A, Zaim S, Tirman PFJ, Miaux Y, White D, et al. Whole-organ magnetic resonance imaging score (WORMS) of the knee in osteoarthritis. Osteoarthr Cartil. 2004;12(3):177–90. 21. Demange MK, Sisto M, Rodeo S. Future trends for unicompartmental arthritis of the knee: injectables & stem cells. Clin Sports Med. 2014;33(1):161–74. 22. Leslie M. Knee osteoarthritis management therapies. Pain Manag Nurs. 2000;1(2):51–7. 23. Paoloni M, Bernetti A, Belelli A, Brignoli O, Buoso S, Caputi AP, et al. Appropriateness of clinical and organizational criteria for intra-articular injection therapies in osteoarthritis: A Delphi method consensus initiative among experts in Italy. Ann Ist Super Sanita. 2015;51:131–8. 24. Sandmark H, Hogstedt C, Lewold S, Vingård E. Osteoarthrosis of the knee in men and women in association with overweight, smoking, and hormone therapy. Ann Rheum Dis. 1999;58(3):151–5. 25. Christensen BV, Iuhl IU, Vilbek H, Bülow H, Dreijer NC, Rasmussen HF. Acupuncture treatment of severe knee osteoarthrosis. A long‐term study. Acta Anaesthesiol Scand. 1992;36(6):519–25. 26. de Sire A, Agostini F, Lippi L, Mangone M, Marchese S, Cisari C, et al. Oxygen-Ozone Therapy in the Rehabilitation Field: State of the Art on Mechanisms of  Action, Safety and Effectiveness in Patients with Musculoskeletal Disorders. Biomolecules. 2021 Feb;11(3). 27. Fransen M, McCONNELL S. Land-based exercise for osteoarthritis of the knee: a metaanalysis of randomized controlled trials. J Rheumatol. 2009;36(6):1109–17. 28. Hiemstra LA, Kerslake S, Irving C. Anterior knee pain in the athlete. Clin Sports Med. 2014;33(3):437–59. 29. Filardo G, Kon E, Di Matteo B, Pelotti P, Di Martino A, Marcacci M. Platelet-rich plasma for the treatment of patellar tendinopathy: clinical and imaging findings at medium-term follow-up. Int Orthop [Internet]. 2013;37(8):1583–9. Available from: https://doi.org/10.1007/s00264-013-1972-830. Trojian TH, Concoff AL, Joy SM, Hatzenbuehler JR, Saulsberry WJ, Coleman CI. AMSSM Scientific Statement Concerning Viscosupplementation Injections for Knee  Osteoarthritis: Importance for Individual Patient Outcomes. Clin J Sport Med  Off J Can Acad  Sport Med. 2016 Jan;26(1):1–11. 31. Ammar TY, Pereira TAP, Mistura SLL, Kuhn A, Saggin JI, Lopes OV. Viscosupplementation for treating knee osteoarthrosis: review of the literature. Rev Bras Ortop. 2015;50:489–94. 32. Salini V, Pantalone A, Vanni D, Abate M. Viscosupplementation in Athletes. In: Arthroscopy and Sport Injuries. Springer; 2016. p. 433–7. 33. Erdem Y, Gul D, Akpancar S. Comparison of Intraarticular Injections of Hyaluronic Acid versus Dextrose Applied with Periarticular Prolotherapy in the Treatment of Recreational Athletes with Knee Osteoarthritis. Turkish J Sport Med. 2020;55(1):6–13. 34. Kalkhoven J, Coutts AJ, Impellizzeri FM. 'Training load error' is not a more accurate term than 'overuse' injury. Br J Sports Med [Internet]. 2020 Aug 1;54(15):934 LP – 935. Available from: http://bjsm.bmj.com/content/54/15/934.abstract35. Odole AC, Agbomeji OT, Onyeso OKK, Ojo JO, Odunaiya NA. Perspectives of Nigerian Athletes About Physiotherapy Services in Sports Injury Management: Implications for Rehabilitation. J Sport Rehabil. 2021;1(aop):1–8. 36. Hendrika W, Reswari A. The effect of physiotherapy on pain improvement in patients with early knee osteoarthritis at RSU UKI. Int J Med Heal Res. 2021;7(6):52–9. 37. MacKay C, Hawker GA, Jaglal SB. How Do Physical Therapists Approach Management of People With Early Knee Osteoarthritis? A Qualitative Study. Phys Ther [Internet]. 2019;100(2):295–306. Available from: https://doi.org/10.1093/ptj/pzz16438. Hrazdira L, Pilecki Z, Pilecki G. Ultrasound guided application of HA in the soft tissues of the locomotor system in athletes–First experience. Krátká sdělení Botek, M, Stejskal, P, Klimešová, I Vliv normobarické hypoxie na Akt Auton Nerv systému Pilot Stud 191 Drbošalová, V, Stejskal, P, Klugar, M, Krejčíř, V, Hynková, O Charakt klientů komerčn. :205. 39. Ricci M, Micheloni GM, Berti M, Perusi F, Sambugaro E, Vecchini E, et al. Clinical comparison of oral administration and viscosupplementation of hyaluronic acid (HA) in early knee osteoarthritis. Musculoskelet Surg. 2017;101(1):45–9. 40. Wang C-T, Lin Y-T, Chiang B-L, Lin Y-H, Hou S-M. High molecular weight hyaluronic acid down-regulates the gene expression of osteoarthritis-associated cytokines and enzymes in fibroblast-like synoviocytes from patients with early osteoarthritis. Osteoarthr Cartil. 2006;14(12):1237–47. 41. Irrgang JJ, Anderson AF. Development and validation of health-related quality of life measures for the knee. Clin Orthop Relat Res. 2002;402:95–109. 42. Roos EM, Roos HP, Lohmander LS, Ekdahl C, Beynnon BD. Knee Injury and Osteoarthritis Outcome Score (KOOS)—development of a self-administered outcome measure. J Orthop Sport Phys Ther. 1998;28(2):88–96. 43. Perruccio A V, Canizares M, Hawker GA, Roos EM, Maillefert JF, Lohmander SL. Comparative Validity and Responsiveness of the HOOS-PS and KOOS-PS to the WOMAC Physical Function Subscale in Total Joint Replacement for Osteoarthritis. 44. Salavati M, Mazaheri M, Negahban H, Sohani SM, Ebrahimian MR, Ebrahimi I, et al. Validation of a Persian-version of Knee injury and Osteoarthritis Outcome Score (KOOS) in Iranians with knee injuries. Osteoarthr Cartil. 2008;16(10):1178–82. 45. Higgins LD, Taylor MK, Park D, Ghodadra N, Marchant M, Pietrobon R, et al. Reliability and validity of the International Knee Documentation Committee (IKDC) subjective knee form. Jt Bone Spine. 2007;74(6):594–9. 46. Kujala UM, Kaprio J, Sarno S. Osteoarthritis of weight bearing joints of lower limbs in former elite male athletes. Bmj. 1994;308(6923):231–4. 47. Salini V, Pantalone A, Vanni D. Arthroscopy and Sport Injuries. Arthrosc Sport Inj. 2016;433–7. 48. Broglio K. Randomization in clinical trials: Permuted blocks and stratification. JAMA - J Am Med Assoc. 2018;319(21):2223–4. 49. Emanuel EJ, Wendler D, Grady C. What makes clinical research ethical? JAMA. 2000 May;283(20):2701–11. )

[32.	Salini V, Pantalone A, Vanni D, Abate M. Viscosupplementation in Athletes. In: Arthroscopy and Sport Injuries. Springer; 2016. p. 433–7.](1. Patel DR, Villalobos A. Evaluation and management of knee pain in young athletes: overuse injuries of the knee. Transl Pediatr [Internet]. 2017 Jul;6(3):190–8. Available from: https://pubmed.ncbi.nlm.nih.gov/287950102. Nicolini AP, de Carvalho RT, Matsuda MM, Filho JS, Cohen M. Common injuries in athletes' knee: Experience of a specialized center. Acta Ortop Bras. 2014;22(3):127–31. 3. Tiu T, Craig Van Dien MD. Knee overuse disorders. 4. Fuller CW, Ekstrand J, Junge A, Andersen TE, Bahr R, Dvorak J, et al. Consensus statement on injury definitions and data collection procedures in studies  of football (soccer) injuries. Br J Sports Med. 2006 Mar;40(3):193–201. 5. Majewski M, Susanne H, Klaus S. Epidemiology of athletic knee injuries: A 10-year study. Knee. 2006 Jun;13(3):184–8. 6. Wilder RP, Sethi S. Overuse injuries: tendinopathies, stress fractures, compartment syndrome, and shin  splints. Clin Sports Med. 2004 Jan;23(1):55–81, vi. 7. Aicale R, Tarantino D, Maffulli N. Overuse injuries in sport: a comprehensive overview. J Orthop Surg Res. 2018 Dec;13(1):309. 8. Yang J, Tibbetts AS, Covassin T, Cheng G, Nayar S, Heiden E. Epidemiology of overuse and acute injuries among competitive collegiate athletes. J Athl Train. 2012;47(2):198–204. 9. Bernetti A, Mangone M, Paoloni M, Di Sante L, Murgia M, Santilli V. Corticosteroid and hyaluronic acid injection therapy in tennis elbow (lateral epicondylalgia). Med Sport. 2014;67(2):289–95. 10. Iolascon G, Gimigliano F, Moretti A, De Sire A, Migliore A, Brandi ML, et al. Early osteoarthritis: How to define, diagnose, and manage. A systematic review. Eur Geriatr Med. 2017;8(5–6):383–96. 11. Drawer S, Fuller CW. Propensity for osteoarthritis and lower limb joint pain in retired professional soccer players. Br J Sports Med. 2001;35(6):402–8. 12. Vannini F, Spalding T, Andriolo L, Berruto M, Denti M, Espregueira-Mendes J, et al. Sport and early osteoarthritis: the role of sport in aetiology, progression and treatment of knee osteoarthritis. Knee Surgery, Sport Traumatol Arthrosc. 2016;24(6):1786–96. 13. Fröhlich S, Pazeller S, Cherati AS, Müller E, Frey WO, Spörri J. Overuse injuries in the knee, back and hip of top elite female alpine skiers during  the off-season preparation period: prevalence, severity and their association with traumatic preinjuries and training load. BMJ open Sport Exerc Med. 2020;6(1):e000892. 14. Rincon GA, Vyas D, Zhou J, Fu FH, Oiestad BE, Holm I, et al. \“ Knee function and prevalence of knee osteoarthritis after anterior cruciate ligament reconstruction: a prospective study with 10 to 15 years of follow-up\”. Letter to the editor. Am J Sports Med. 2011;39(4):NP3-author. 15. Kellgren JH, Lawrence J. Radiological assessment of osteo-arthrosis. Ann Rheum Dis. 1957;16(4):494. 16. Altman R, Asch E, Bloch D, Bole G, Borenstein D, Brandt K, et al. Development of criteria for the classification and reporting of osteoarthritis: classification of osteoarthritis of the knee. Arthritis Rheum Off J Am Coll Rheumatol. 1986;29(8):1039–49. 17. Luyten FP, Denti M, Filardo G, Kon E, Engebretsen L. Definition and classification of early osteoarthritis of the knee. Knee Surgery, Sport Traumatol Arthrosc. 2012;20(3):401–6. 18. Hoemann C, Kandel R, Roberts S, Saris DBF, Creemers L, Mainil-Varlet P, et al. International Cartilage Repair Society (ICRS) Recommended Guidelines for  Histological Endpoints for Cartilage Repair Studies in Animal Models and Clinical Trials. Cartilage. 2011 Apr;2(2):153–72. 19. Hunter DJ, Lo GH, Gale D, Grainger AJ, Guermazi A, Conaghan PG. The reliability of a new scoring system for knee osteoarthritis MRI and the validity of bone marrow lesion assessment: BLOKS (Boston–Leeds Osteoarthritis Knee Score). Ann Rheum Dis. 2008;67(2):206–11. 20. Peterfy CG, Guermazi A, Zaim S, Tirman PFJ, Miaux Y, White D, et al. Whole-organ magnetic resonance imaging score (WORMS) of the knee in osteoarthritis. Osteoarthr Cartil. 2004;12(3):177–90. 21. Demange MK, Sisto M, Rodeo S. Future trends for unicompartmental arthritis of the knee: injectables & stem cells. Clin Sports Med. 2014;33(1):161–74. 22. Leslie M. Knee osteoarthritis management therapies. Pain Manag Nurs. 2000;1(2):51–7. 23. Paoloni M, Bernetti A, Belelli A, Brignoli O, Buoso S, Caputi AP, et al. Appropriateness of clinical and organizational criteria for intra-articular injection therapies in osteoarthritis: A Delphi method consensus initiative among experts in Italy. Ann Ist Super Sanita. 2015;51:131–8. 24. Sandmark H, Hogstedt C, Lewold S, Vingård E. Osteoarthrosis of the knee in men and women in association with overweight, smoking, and hormone therapy. Ann Rheum Dis. 1999;58(3):151–5. 25. Christensen BV, Iuhl IU, Vilbek H, Bülow H, Dreijer NC, Rasmussen HF. Acupuncture treatment of severe knee osteoarthrosis. A long‐term study. Acta Anaesthesiol Scand. 1992;36(6):519–25. 26. de Sire A, Agostini F, Lippi L, Mangone M, Marchese S, Cisari C, et al. Oxygen-Ozone Therapy in the Rehabilitation Field: State of the Art on Mechanisms of  Action, Safety and Effectiveness in Patients with Musculoskeletal Disorders. Biomolecules. 2021 Feb;11(3). 27. Fransen M, McCONNELL S. Land-based exercise for osteoarthritis of the knee: a metaanalysis of randomized controlled trials. J Rheumatol. 2009;36(6):1109–17. 28. Hiemstra LA, Kerslake S, Irving C. Anterior knee pain in the athlete. Clin Sports Med. 2014;33(3):437–59. 29. Filardo G, Kon E, Di Matteo B, Pelotti P, Di Martino A, Marcacci M. Platelet-rich plasma for the treatment of patellar tendinopathy: clinical and imaging findings at medium-term follow-up. Int Orthop [Internet]. 2013;37(8):1583–9. Available from: https://doi.org/10.1007/s00264-013-1972-830. Trojian TH, Concoff AL, Joy SM, Hatzenbuehler JR, Saulsberry WJ, Coleman CI. AMSSM Scientific Statement Concerning Viscosupplementation Injections for Knee  Osteoarthritis: Importance for Individual Patient Outcomes. Clin J Sport Med  Off J Can Acad  Sport Med. 2016 Jan;26(1):1–11. 31. Ammar TY, Pereira TAP, Mistura SLL, Kuhn A, Saggin JI, Lopes OV. Viscosupplementation for treating knee osteoarthrosis: review of the literature. Rev Bras Ortop. 2015;50:489–94. 32. Salini V, Pantalone A, Vanni D, Abate M. Viscosupplementation in Athletes. In: Arthroscopy and Sport Injuries. Springer; 2016. p. 433–7. 33. Erdem Y, Gul D, Akpancar S. Comparison of Intraarticular Injections of Hyaluronic Acid versus Dextrose Applied with Periarticular Prolotherapy in the Treatment of Recreational Athletes with Knee Osteoarthritis. Turkish J Sport Med. 2020;55(1):6–13. 34. Kalkhoven J, Coutts AJ, Impellizzeri FM. 'Training load error' is not a more accurate term than 'overuse' injury. Br J Sports Med [Internet]. 2020 Aug 1;54(15):934 LP – 935. Available from: http://bjsm.bmj.com/content/54/15/934.abstract35. Odole AC, Agbomeji OT, Onyeso OKK, Ojo JO, Odunaiya NA. Perspectives of Nigerian Athletes About Physiotherapy Services in Sports Injury Management: Implications for Rehabilitation. J Sport Rehabil. 2021;1(aop):1–8. 36. Hendrika W, Reswari A. The effect of physiotherapy on pain improvement in patients with early knee osteoarthritis at RSU UKI. Int J Med Heal Res. 2021;7(6):52–9. 37. MacKay C, Hawker GA, Jaglal SB. How Do Physical Therapists Approach Management of People With Early Knee Osteoarthritis? A Qualitative Study. Phys Ther [Internet]. 2019;100(2):295–306. Available from: https://doi.org/10.1093/ptj/pzz16438. Hrazdira L, Pilecki Z, Pilecki G. Ultrasound guided application of HA in the soft tissues of the locomotor system in athletes–First experience. Krátká sdělení Botek, M, Stejskal, P, Klimešová, I Vliv normobarické hypoxie na Akt Auton Nerv systému Pilot Stud 191 Drbošalová, V, Stejskal, P, Klugar, M, Krejčíř, V, Hynková, O Charakt klientů komerčn. :205. 39. Ricci M, Micheloni GM, Berti M, Perusi F, Sambugaro E, Vecchini E, et al. Clinical comparison of oral administration and viscosupplementation of hyaluronic acid (HA) in early knee osteoarthritis. Musculoskelet Surg. 2017;101(1):45–9. 40. Wang C-T, Lin Y-T, Chiang B-L, Lin Y-H, Hou S-M. High molecular weight hyaluronic acid down-regulates the gene expression of osteoarthritis-associated cytokines and enzymes in fibroblast-like synoviocytes from patients with early osteoarthritis. Osteoarthr Cartil. 2006;14(12):1237–47. 41. Irrgang JJ, Anderson AF. Development and validation of health-related quality of life measures for the knee. Clin Orthop Relat Res. 2002;402:95–109. 42. Roos EM, Roos HP, Lohmander LS, Ekdahl C, Beynnon BD. Knee Injury and Osteoarthritis Outcome Score (KOOS)—development of a self-administered outcome measure. J Orthop Sport Phys Ther. 1998;28(2):88–96. 43. Perruccio A V, Canizares M, Hawker GA, Roos EM, Maillefert JF, Lohmander SL. Comparative Validity and Responsiveness of the HOOS-PS and KOOS-PS to the WOMAC Physical Function Subscale in Total Joint Replacement for Osteoarthritis. 44. Salavati M, Mazaheri M, Negahban H, Sohani SM, Ebrahimian MR, Ebrahimi I, et al. Validation of a Persian-version of Knee injury and Osteoarthritis Outcome Score (KOOS) in Iranians with knee injuries. Osteoarthr Cartil. 2008;16(10):1178–82. 45. Higgins LD, Taylor MK, Park D, Ghodadra N, Marchant M, Pietrobon R, et al. Reliability and validity of the International Knee Documentation Committee (IKDC) subjective knee form. Jt Bone Spine. 2007;74(6):594–9. 46. Kujala UM, Kaprio J, Sarno S. Osteoarthritis of weight bearing joints of lower limbs in former elite male athletes. Bmj. 1994;308(6923):231–4. 47. Salini V, Pantalone A, Vanni D. Arthroscopy and Sport Injuries. Arthrosc Sport Inj. 2016;433–7. 48. Broglio K. Randomization in clinical trials: Permuted blocks and stratification. JAMA - J Am Med Assoc. 2018;319(21):2223–4. 49. Emanuel EJ, Wendler D, Grady C. What makes clinical research ethical? JAMA. 2000 May;283(20):2701–11. )

[33.	Erdem Y, Gul D, Akpancar S. Comparison of Intraarticular Injections of Hyaluronic Acid versus Dextrose Applied with Periarticular Prolotherapy in the Treatment of Recreational Athletes with Knee Osteoarthritis. Turkish J Sport Med. 2020;55(1):6–13.](1. Patel DR, Villalobos A. Evaluation and management of knee pain in young athletes: overuse injuries of the knee. Transl Pediatr [Internet]. 2017 Jul;6(3):190–8. Available from: https://pubmed.ncbi.nlm.nih.gov/287950102. Nicolini AP, de Carvalho RT, Matsuda MM, Filho JS, Cohen M. Common injuries in athletes' knee: Experience of a specialized center. Acta Ortop Bras. 2014;22(3):127–31. 3. Tiu T, Craig Van Dien MD. Knee overuse disorders. 4. Fuller CW, Ekstrand J, Junge A, Andersen TE, Bahr R, Dvorak J, et al. Consensus statement on injury definitions and data collection procedures in studies  of football (soccer) injuries. Br J Sports Med. 2006 Mar;40(3):193–201. 5. Majewski M, Susanne H, Klaus S. Epidemiology of athletic knee injuries: A 10-year study. Knee. 2006 Jun;13(3):184–8. 6. Wilder RP, Sethi S. Overuse injuries: tendinopathies, stress fractures, compartment syndrome, and shin  splints. Clin Sports Med. 2004 Jan;23(1):55–81, vi. 7. Aicale R, Tarantino D, Maffulli N. Overuse injuries in sport: a comprehensive overview. J Orthop Surg Res. 2018 Dec;13(1):309. 8. Yang J, Tibbetts AS, Covassin T, Cheng G, Nayar S, Heiden E. Epidemiology of overuse and acute injuries among competitive collegiate athletes. J Athl Train. 2012;47(2):198–204. 9. Bernetti A, Mangone M, Paoloni M, Di Sante L, Murgia M, Santilli V. Corticosteroid and hyaluronic acid injection therapy in tennis elbow (lateral epicondylalgia). Med Sport. 2014;67(2):289–95. 10. Iolascon G, Gimigliano F, Moretti A, De Sire A, Migliore A, Brandi ML, et al. Early osteoarthritis: How to define, diagnose, and manage. A systematic review. Eur Geriatr Med. 2017;8(5–6):383–96. 11. Drawer S, Fuller CW. Propensity for osteoarthritis and lower limb joint pain in retired professional soccer players. Br J Sports Med. 2001;35(6):402–8. 12. Vannini F, Spalding T, Andriolo L, Berruto M, Denti M, Espregueira-Mendes J, et al. Sport and early osteoarthritis: the role of sport in aetiology, progression and treatment of knee osteoarthritis. Knee Surgery, Sport Traumatol Arthrosc. 2016;24(6):1786–96. 13. Fröhlich S, Pazeller S, Cherati AS, Müller E, Frey WO, Spörri J. Overuse injuries in the knee, back and hip of top elite female alpine skiers during  the off-season preparation period: prevalence, severity and their association with traumatic preinjuries and training load. BMJ open Sport Exerc Med. 2020;6(1):e000892. 14. Rincon GA, Vyas D, Zhou J, Fu FH, Oiestad BE, Holm I, et al. \“ Knee function and prevalence of knee osteoarthritis after anterior cruciate ligament reconstruction: a prospective study with 10 to 15 years of follow-up\”. Letter to the editor. Am J Sports Med. 2011;39(4):NP3-author. 15. Kellgren JH, Lawrence J. Radiological assessment of osteo-arthrosis. Ann Rheum Dis. 1957;16(4):494. 16. Altman R, Asch E, Bloch D, Bole G, Borenstein D, Brandt K, et al. Development of criteria for the classification and reporting of osteoarthritis: classification of osteoarthritis of the knee. Arthritis Rheum Off J Am Coll Rheumatol. 1986;29(8):1039–49. 17. Luyten FP, Denti M, Filardo G, Kon E, Engebretsen L. Definition and classification of early osteoarthritis of the knee. Knee Surgery, Sport Traumatol Arthrosc. 2012;20(3):401–6. 18. Hoemann C, Kandel R, Roberts S, Saris DBF, Creemers L, Mainil-Varlet P, et al. International Cartilage Repair Society (ICRS) Recommended Guidelines for  Histological Endpoints for Cartilage Repair Studies in Animal Models and Clinical Trials. Cartilage. 2011 Apr;2(2):153–72. 19. Hunter DJ, Lo GH, Gale D, Grainger AJ, Guermazi A, Conaghan PG. The reliability of a new scoring system for knee osteoarthritis MRI and the validity of bone marrow lesion assessment: BLOKS (Boston–Leeds Osteoarthritis Knee Score). Ann Rheum Dis. 2008;67(2):206–11. 20. Peterfy CG, Guermazi A, Zaim S, Tirman PFJ, Miaux Y, White D, et al. Whole-organ magnetic resonance imaging score (WORMS) of the knee in osteoarthritis. Osteoarthr Cartil. 2004;12(3):177–90. 21. Demange MK, Sisto M, Rodeo S. Future trends for unicompartmental arthritis of the knee: injectables & stem cells. Clin Sports Med. 2014;33(1):161–74. 22. Leslie M. Knee osteoarthritis management therapies. Pain Manag Nurs. 2000;1(2):51–7. 23. Paoloni M, Bernetti A, Belelli A, Brignoli O, Buoso S, Caputi AP, et al. Appropriateness of clinical and organizational criteria for intra-articular injection therapies in osteoarthritis: A Delphi method consensus initiative among experts in Italy. Ann Ist Super Sanita. 2015;51:131–8. 24. Sandmark H, Hogstedt C, Lewold S, Vingård E. Osteoarthrosis of the knee in men and women in association with overweight, smoking, and hormone therapy. Ann Rheum Dis. 1999;58(3):151–5. 25. Christensen BV, Iuhl IU, Vilbek H, Bülow H, Dreijer NC, Rasmussen HF. Acupuncture treatment of severe knee osteoarthrosis. A long‐term study. Acta Anaesthesiol Scand. 1992;36(6):519–25. 26. de Sire A, Agostini F, Lippi L, Mangone M, Marchese S, Cisari C, et al. Oxygen-Ozone Therapy in the Rehabilitation Field: State of the Art on Mechanisms of  Action, Safety and Effectiveness in Patients with Musculoskeletal Disorders. Biomolecules. 2021 Feb;11(3). 27. Fransen M, McCONNELL S. Land-based exercise for osteoarthritis of the knee: a metaanalysis of randomized controlled trials. J Rheumatol. 2009;36(6):1109–17. 28. Hiemstra LA, Kerslake S, Irving C. Anterior knee pain in the athlete. Clin Sports Med. 2014;33(3):437–59. 29. Filardo G, Kon E, Di Matteo B, Pelotti P, Di Martino A, Marcacci M. Platelet-rich plasma for the treatment of patellar tendinopathy: clinical and imaging findings at medium-term follow-up. Int Orthop [Internet]. 2013;37(8):1583–9. Available from: https://doi.org/10.1007/s00264-013-1972-830. Trojian TH, Concoff AL, Joy SM, Hatzenbuehler JR, Saulsberry WJ, Coleman CI. AMSSM Scientific Statement Concerning Viscosupplementation Injections for Knee  Osteoarthritis: Importance for Individual Patient Outcomes. Clin J Sport Med  Off J Can Acad  Sport Med. 2016 Jan;26(1):1–11. 31. Ammar TY, Pereira TAP, Mistura SLL, Kuhn A, Saggin JI, Lopes OV. Viscosupplementation for treating knee osteoarthrosis: review of the literature. Rev Bras Ortop. 2015;50:489–94. 32. Salini V, Pantalone A, Vanni D, Abate M. Viscosupplementation in Athletes. In: Arthroscopy and Sport Injuries. Springer; 2016. p. 433–7. 33. Erdem Y, Gul D, Akpancar S. Comparison of Intraarticular Injections of Hyaluronic Acid versus Dextrose Applied with Periarticular Prolotherapy in the Treatment of Recreational Athletes with Knee Osteoarthritis. Turkish J Sport Med. 2020;55(1):6–13. 34. Kalkhoven J, Coutts AJ, Impellizzeri FM. 'Training load error' is not a more accurate term than 'overuse' injury. Br J Sports Med [Internet]. 2020 Aug 1;54(15):934 LP – 935. Available from: http://bjsm.bmj.com/content/54/15/934.abstract35. Odole AC, Agbomeji OT, Onyeso OKK, Ojo JO, Odunaiya NA. Perspectives of Nigerian Athletes About Physiotherapy Services in Sports Injury Management: Implications for Rehabilitation. J Sport Rehabil. 2021;1(aop):1–8. 36. Hendrika W, Reswari A. The effect of physiotherapy on pain improvement in patients with early knee osteoarthritis at RSU UKI. Int J Med Heal Res. 2021;7(6):52–9. 37. MacKay C, Hawker GA, Jaglal SB. How Do Physical Therapists Approach Management of People With Early Knee Osteoarthritis? A Qualitative Study. Phys Ther [Internet]. 2019;100(2):295–306. Available from: https://doi.org/10.1093/ptj/pzz16438. Hrazdira L, Pilecki Z, Pilecki G. Ultrasound guided application of HA in the soft tissues of the locomotor system in athletes–First experience. Krátká sdělení Botek, M, Stejskal, P, Klimešová, I Vliv normobarické hypoxie na Akt Auton Nerv systému Pilot Stud 191 Drbošalová, V, Stejskal, P, Klugar, M, Krejčíř, V, Hynková, O Charakt klientů komerčn. :205. 39. Ricci M, Micheloni GM, Berti M, Perusi F, Sambugaro E, Vecchini E, et al. Clinical comparison of oral administration and viscosupplementation of hyaluronic acid (HA) in early knee osteoarthritis. Musculoskelet Surg. 2017;101(1):45–9. 40. Wang C-T, Lin Y-T, Chiang B-L, Lin Y-H, Hou S-M. High molecular weight hyaluronic acid down-regulates the gene expression of osteoarthritis-associated cytokines and enzymes in fibroblast-like synoviocytes from patients with early osteoarthritis. Osteoarthr Cartil. 2006;14(12):1237–47. 41. Irrgang JJ, Anderson AF. Development and validation of health-related quality of life measures for the knee. Clin Orthop Relat Res. 2002;402:95–109. 42. Roos EM, Roos HP, Lohmander LS, Ekdahl C, Beynnon BD. Knee Injury and Osteoarthritis Outcome Score (KOOS)—development of a self-administered outcome measure. J Orthop Sport Phys Ther. 1998;28(2):88–96. 43. Perruccio A V, Canizares M, Hawker GA, Roos EM, Maillefert JF, Lohmander SL. Comparative Validity and Responsiveness of the HOOS-PS and KOOS-PS to the WOMAC Physical Function Subscale in Total Joint Replacement for Osteoarthritis. 44. Salavati M, Mazaheri M, Negahban H, Sohani SM, Ebrahimian MR, Ebrahimi I, et al. Validation of a Persian-version of Knee injury and Osteoarthritis Outcome Score (KOOS) in Iranians with knee injuries. Osteoarthr Cartil. 2008;16(10):1178–82. 45. Higgins LD, Taylor MK, Park D, Ghodadra N, Marchant M, Pietrobon R, et al. Reliability and validity of the International Knee Documentation Committee (IKDC) subjective knee form. Jt Bone Spine. 2007;74(6):594–9. 46. Kujala UM, Kaprio J, Sarno S. Osteoarthritis of weight bearing joints of lower limbs in former elite male athletes. Bmj. 1994;308(6923):231–4. 47. Salini V, Pantalone A, Vanni D. Arthroscopy and Sport Injuries. Arthrosc Sport Inj. 2016;433–7. 48. Broglio K. Randomization in clinical trials: Permuted blocks and stratification. JAMA - J Am Med Assoc. 2018;319(21):2223–4. 49. Emanuel EJ, Wendler D, Grady C. What makes clinical research ethical? JAMA. 2000 May;283(20):2701–11. )

[34.	Kalkhoven J, Coutts AJ, Impellizzeri FM. ‘Training load error’ is not a more accurate term than ‘overuse’ injury. Br J Sports Med [Internet]. 2020 Aug 1;54(15):934 LP – 935. Available from: http://bjsm.bmj.com/content/54/15/934.abstract](1. Patel DR, Villalobos A. Evaluation and management of knee pain in young athletes: overuse injuries of the knee. Transl Pediatr [Internet]. 2017 Jul;6(3):190–8. Available from: https://pubmed.ncbi.nlm.nih.gov/287950102. Nicolini AP, de Carvalho RT, Matsuda MM, Filho JS, Cohen M. Common injuries in athletes' knee: Experience of a specialized center. Acta Ortop Bras. 2014;22(3):127–31. 3. Tiu T, Craig Van Dien MD. Knee overuse disorders. 4. Fuller CW, Ekstrand J, Junge A, Andersen TE, Bahr R, Dvorak J, et al. Consensus statement on injury definitions and data collection procedures in studies  of football (soccer) injuries. Br J Sports Med. 2006 Mar;40(3):193–201. 5. Majewski M, Susanne H, Klaus S. Epidemiology of athletic knee injuries: A 10-year study. Knee. 2006 Jun;13(3):184–8. 6. Wilder RP, Sethi S. Overuse injuries: tendinopathies, stress fractures, compartment syndrome, and shin  splints. Clin Sports Med. 2004 Jan;23(1):55–81, vi. 7. Aicale R, Tarantino D, Maffulli N. Overuse injuries in sport: a comprehensive overview. J Orthop Surg Res. 2018 Dec;13(1):309. 8. Yang J, Tibbetts AS, Covassin T, Cheng G, Nayar S, Heiden E. Epidemiology of overuse and acute injuries among competitive collegiate athletes. J Athl Train. 2012;47(2):198–204. 9. Bernetti A, Mangone M, Paoloni M, Di Sante L, Murgia M, Santilli V. Corticosteroid and hyaluronic acid injection therapy in tennis elbow (lateral epicondylalgia). Med Sport. 2014;67(2):289–95. 10. Iolascon G, Gimigliano F, Moretti A, De Sire A, Migliore A, Brandi ML, et al. Early osteoarthritis: How to define, diagnose, and manage. A systematic review. Eur Geriatr Med. 2017;8(5–6):383–96. 11. Drawer S, Fuller CW. Propensity for osteoarthritis and lower limb joint pain in retired professional soccer players. Br J Sports Med. 2001;35(6):402–8. 12. Vannini F, Spalding T, Andriolo L, Berruto M, Denti M, Espregueira-Mendes J, et al. Sport and early osteoarthritis: the role of sport in aetiology, progression and treatment of knee osteoarthritis. Knee Surgery, Sport Traumatol Arthrosc. 2016;24(6):1786–96. 13. Fröhlich S, Pazeller S, Cherati AS, Müller E, Frey WO, Spörri J. Overuse injuries in the knee, back and hip of top elite female alpine skiers during  the off-season preparation period: prevalence, severity and their association with traumatic preinjuries and training load. BMJ open Sport Exerc Med. 2020;6(1):e000892. 14. Rincon GA, Vyas D, Zhou J, Fu FH, Oiestad BE, Holm I, et al. \“ Knee function and prevalence of knee osteoarthritis after anterior cruciate ligament reconstruction: a prospective study with 10 to 15 years of follow-up\”. Letter to the editor. Am J Sports Med. 2011;39(4):NP3-author. 15. Kellgren JH, Lawrence J. Radiological assessment of osteo-arthrosis. Ann Rheum Dis. 1957;16(4):494. 16. Altman R, Asch E, Bloch D, Bole G, Borenstein D, Brandt K, et al. Development of criteria for the classification and reporting of osteoarthritis: classification of osteoarthritis of the knee. Arthritis Rheum Off J Am Coll Rheumatol. 1986;29(8):1039–49. 17. Luyten FP, Denti M, Filardo G, Kon E, Engebretsen L. Definition and classification of early osteoarthritis of the knee. Knee Surgery, Sport Traumatol Arthrosc. 2012;20(3):401–6. 18. Hoemann C, Kandel R, Roberts S, Saris DBF, Creemers L, Mainil-Varlet P, et al. International Cartilage Repair Society (ICRS) Recommended Guidelines for  Histological Endpoints for Cartilage Repair Studies in Animal Models and Clinical Trials. Cartilage. 2011 Apr;2(2):153–72. 19. Hunter DJ, Lo GH, Gale D, Grainger AJ, Guermazi A, Conaghan PG. The reliability of a new scoring system for knee osteoarthritis MRI and the validity of bone marrow lesion assessment: BLOKS (Boston–Leeds Osteoarthritis Knee Score). Ann Rheum Dis. 2008;67(2):206–11. 20. Peterfy CG, Guermazi A, Zaim S, Tirman PFJ, Miaux Y, White D, et al. Whole-organ magnetic resonance imaging score (WORMS) of the knee in osteoarthritis. Osteoarthr Cartil. 2004;12(3):177–90. 21. Demange MK, Sisto M, Rodeo S. Future trends for unicompartmental arthritis of the knee: injectables & stem cells. Clin Sports Med. 2014;33(1):161–74. 22. Leslie M. Knee osteoarthritis management therapies. Pain Manag Nurs. 2000;1(2):51–7. 23. Paoloni M, Bernetti A, Belelli A, Brignoli O, Buoso S, Caputi AP, et al. Appropriateness of clinical and organizational criteria for intra-articular injection therapies in osteoarthritis: A Delphi method consensus initiative among experts in Italy. Ann Ist Super Sanita. 2015;51:131–8. 24. Sandmark H, Hogstedt C, Lewold S, Vingård E. Osteoarthrosis of the knee in men and women in association with overweight, smoking, and hormone therapy. Ann Rheum Dis. 1999;58(3):151–5. 25. Christensen BV, Iuhl IU, Vilbek H, Bülow H, Dreijer NC, Rasmussen HF. Acupuncture treatment of severe knee osteoarthrosis. A long‐term study. Acta Anaesthesiol Scand. 1992;36(6):519–25. 26. de Sire A, Agostini F, Lippi L, Mangone M, Marchese S, Cisari C, et al. Oxygen-Ozone Therapy in the Rehabilitation Field: State of the Art on Mechanisms of  Action, Safety and Effectiveness in Patients with Musculoskeletal Disorders. Biomolecules. 2021 Feb;11(3). 27. Fransen M, McCONNELL S. Land-based exercise for osteoarthritis of the knee: a metaanalysis of randomized controlled trials. J Rheumatol. 2009;36(6):1109–17. 28. Hiemstra LA, Kerslake S, Irving C. Anterior knee pain in the athlete. Clin Sports Med. 2014;33(3):437–59. 29. Filardo G, Kon E, Di Matteo B, Pelotti P, Di Martino A, Marcacci M. Platelet-rich plasma for the treatment of patellar tendinopathy: clinical and imaging findings at medium-term follow-up. Int Orthop [Internet]. 2013;37(8):1583–9. Available from: https://doi.org/10.1007/s00264-013-1972-830. Trojian TH, Concoff AL, Joy SM, Hatzenbuehler JR, Saulsberry WJ, Coleman CI. AMSSM Scientific Statement Concerning Viscosupplementation Injections for Knee  Osteoarthritis: Importance for Individual Patient Outcomes. Clin J Sport Med  Off J Can Acad  Sport Med. 2016 Jan;26(1):1–11. 31. Ammar TY, Pereira TAP, Mistura SLL, Kuhn A, Saggin JI, Lopes OV. Viscosupplementation for treating knee osteoarthrosis: review of the literature. Rev Bras Ortop. 2015;50:489–94. 32. Salini V, Pantalone A, Vanni D, Abate M. Viscosupplementation in Athletes. In: Arthroscopy and Sport Injuries. Springer; 2016. p. 433–7. 33. Erdem Y, Gul D, Akpancar S. Comparison of Intraarticular Injections of Hyaluronic Acid versus Dextrose Applied with Periarticular Prolotherapy in the Treatment of Recreational Athletes with Knee Osteoarthritis. Turkish J Sport Med. 2020;55(1):6–13. 34. Kalkhoven J, Coutts AJ, Impellizzeri FM. 'Training load error' is not a more accurate term than 'overuse' injury. Br J Sports Med [Internet]. 2020 Aug 1;54(15):934 LP – 935. Available from: http://bjsm.bmj.com/content/54/15/934.abstract35. Odole AC, Agbomeji OT, Onyeso OKK, Ojo JO, Odunaiya NA. Perspectives of Nigerian Athletes About Physiotherapy Services in Sports Injury Management: Implications for Rehabilitation. J Sport Rehabil. 2021;1(aop):1–8. 36. Hendrika W, Reswari A. The effect of physiotherapy on pain improvement in patients with early knee osteoarthritis at RSU UKI. Int J Med Heal Res. 2021;7(6):52–9. 37. MacKay C, Hawker GA, Jaglal SB. How Do Physical Therapists Approach Management of People With Early Knee Osteoarthritis? A Qualitative Study. Phys Ther [Internet]. 2019;100(2):295–306. Available from: https://doi.org/10.1093/ptj/pzz16438. Hrazdira L, Pilecki Z, Pilecki G. Ultrasound guided application of HA in the soft tissues of the locomotor system in athletes–First experience. Krátká sdělení Botek, M, Stejskal, P, Klimešová, I Vliv normobarické hypoxie na Akt Auton Nerv systému Pilot Stud 191 Drbošalová, V, Stejskal, P, Klugar, M, Krejčíř, V, Hynková, O Charakt klientů komerčn. :205. 39. Ricci M, Micheloni GM, Berti M, Perusi F, Sambugaro E, Vecchini E, et al. Clinical comparison of oral administration and viscosupplementation of hyaluronic acid (HA) in early knee osteoarthritis. Musculoskelet Surg. 2017;101(1):45–9. 40. Wang C-T, Lin Y-T, Chiang B-L, Lin Y-H, Hou S-M. High molecular weight hyaluronic acid down-regulates the gene expression of osteoarthritis-associated cytokines and enzymes in fibroblast-like synoviocytes from patients with early osteoarthritis. Osteoarthr Cartil. 2006;14(12):1237–47. 41. Irrgang JJ, Anderson AF. Development and validation of health-related quality of life measures for the knee. Clin Orthop Relat Res. 2002;402:95–109. 42. Roos EM, Roos HP, Lohmander LS, Ekdahl C, Beynnon BD. Knee Injury and Osteoarthritis Outcome Score (KOOS)—development of a self-administered outcome measure. J Orthop Sport Phys Ther. 1998;28(2):88–96. 43. Perruccio A V, Canizares M, Hawker GA, Roos EM, Maillefert JF, Lohmander SL. Comparative Validity and Responsiveness of the HOOS-PS and KOOS-PS to the WOMAC Physical Function Subscale in Total Joint Replacement for Osteoarthritis. 44. Salavati M, Mazaheri M, Negahban H, Sohani SM, Ebrahimian MR, Ebrahimi I, et al. Validation of a Persian-version of Knee injury and Osteoarthritis Outcome Score (KOOS) in Iranians with knee injuries. Osteoarthr Cartil. 2008;16(10):1178–82. 45. Higgins LD, Taylor MK, Park D, Ghodadra N, Marchant M, Pietrobon R, et al. Reliability and validity of the International Knee Documentation Committee (IKDC) subjective knee form. Jt Bone Spine. 2007;74(6):594–9. 46. Kujala UM, Kaprio J, Sarno S. Osteoarthritis of weight bearing joints of lower limbs in former elite male athletes. Bmj. 1994;308(6923):231–4. 47. Salini V, Pantalone A, Vanni D. Arthroscopy and Sport Injuries. Arthrosc Sport Inj. 2016;433–7. 48. Broglio K. Randomization in clinical trials: Permuted blocks and stratification. JAMA - J Am Med Assoc. 2018;319(21):2223–4. 49. Emanuel EJ, Wendler D, Grady C. What makes clinical research ethical? JAMA. 2000 May;283(20):2701–11. )

[35.	Odole AC, Agbomeji OT, Onyeso OKK, Ojo JO, Odunaiya NA. Perspectives of Nigerian Athletes About Physiotherapy Services in Sports Injury Management: Implications for Rehabilitation. J Sport Rehabil. 2021;1(aop):1–8.](1. Patel DR, Villalobos A. Evaluation and management of knee pain in young athletes: overuse injuries of the knee. Transl Pediatr [Internet]. 2017 Jul;6(3):190–8. Available from: https://pubmed.ncbi.nlm.nih.gov/287950102. Nicolini AP, de Carvalho RT, Matsuda MM, Filho JS, Cohen M. Common injuries in athletes' knee: Experience of a specialized center. Acta Ortop Bras. 2014;22(3):127–31. 3. Tiu T, Craig Van Dien MD. Knee overuse disorders. 4. Fuller CW, Ekstrand J, Junge A, Andersen TE, Bahr R, Dvorak J, et al. Consensus statement on injury definitions and data collection procedures in studies  of football (soccer) injuries. Br J Sports Med. 2006 Mar;40(3):193–201. 5. Majewski M, Susanne H, Klaus S. Epidemiology of athletic knee injuries: A 10-year study. Knee. 2006 Jun;13(3):184–8. 6. Wilder RP, Sethi S. Overuse injuries: tendinopathies, stress fractures, compartment syndrome, and shin  splints. Clin Sports Med. 2004 Jan;23(1):55–81, vi. 7. Aicale R, Tarantino D, Maffulli N. Overuse injuries in sport: a comprehensive overview. J Orthop Surg Res. 2018 Dec;13(1):309. 8. Yang J, Tibbetts AS, Covassin T, Cheng G, Nayar S, Heiden E. Epidemiology of overuse and acute injuries among competitive collegiate athletes. J Athl Train. 2012;47(2):198–204. 9. Bernetti A, Mangone M, Paoloni M, Di Sante L, Murgia M, Santilli V. Corticosteroid and hyaluronic acid injection therapy in tennis elbow (lateral epicondylalgia). Med Sport. 2014;67(2):289–95. 10. Iolascon G, Gimigliano F, Moretti A, De Sire A, Migliore A, Brandi ML, et al. Early osteoarthritis: How to define, diagnose, and manage. A systematic review. Eur Geriatr Med. 2017;8(5–6):383–96. 11. Drawer S, Fuller CW. Propensity for osteoarthritis and lower limb joint pain in retired professional soccer players. Br J Sports Med. 2001;35(6):402–8. 12. Vannini F, Spalding T, Andriolo L, Berruto M, Denti M, Espregueira-Mendes J, et al. Sport and early osteoarthritis: the role of sport in aetiology, progression and treatment of knee osteoarthritis. Knee Surgery, Sport Traumatol Arthrosc. 2016;24(6):1786–96. 13. Fröhlich S, Pazeller S, Cherati AS, Müller E, Frey WO, Spörri J. Overuse injuries in the knee, back and hip of top elite female alpine skiers during  the off-season preparation period: prevalence, severity and their association with traumatic preinjuries and training load. BMJ open Sport Exerc Med. 2020;6(1):e000892. 14. Rincon GA, Vyas D, Zhou J, Fu FH, Oiestad BE, Holm I, et al. \“ Knee function and prevalence of knee osteoarthritis after anterior cruciate ligament reconstruction: a prospective study with 10 to 15 years of follow-up\”. Letter to the editor. Am J Sports Med. 2011;39(4):NP3-author. 15. Kellgren JH, Lawrence J. Radiological assessment of osteo-arthrosis. Ann Rheum Dis. 1957;16(4):494. 16. Altman R, Asch E, Bloch D, Bole G, Borenstein D, Brandt K, et al. Development of criteria for the classification and reporting of osteoarthritis: classification of osteoarthritis of the knee. Arthritis Rheum Off J Am Coll Rheumatol. 1986;29(8):1039–49. 17. Luyten FP, Denti M, Filardo G, Kon E, Engebretsen L. Definition and classification of early osteoarthritis of the knee. Knee Surgery, Sport Traumatol Arthrosc. 2012;20(3):401–6. 18. Hoemann C, Kandel R, Roberts S, Saris DBF, Creemers L, Mainil-Varlet P, et al. International Cartilage Repair Society (ICRS) Recommended Guidelines for  Histological Endpoints for Cartilage Repair Studies in Animal Models and Clinical Trials. Cartilage. 2011 Apr;2(2):153–72. 19. Hunter DJ, Lo GH, Gale D, Grainger AJ, Guermazi A, Conaghan PG. The reliability of a new scoring system for knee osteoarthritis MRI and the validity of bone marrow lesion assessment: BLOKS (Boston–Leeds Osteoarthritis Knee Score). Ann Rheum Dis. 2008;67(2):206–11. 20. Peterfy CG, Guermazi A, Zaim S, Tirman PFJ, Miaux Y, White D, et al. Whole-organ magnetic resonance imaging score (WORMS) of the knee in osteoarthritis. Osteoarthr Cartil. 2004;12(3):177–90. 21. Demange MK, Sisto M, Rodeo S. Future trends for unicompartmental arthritis of the knee: injectables & stem cells. Clin Sports Med. 2014;33(1):161–74. 22. Leslie M. Knee osteoarthritis management therapies. Pain Manag Nurs. 2000;1(2):51–7. 23. Paoloni M, Bernetti A, Belelli A, Brignoli O, Buoso S, Caputi AP, et al. Appropriateness of clinical and organizational criteria for intra-articular injection therapies in osteoarthritis: A Delphi method consensus initiative among experts in Italy. Ann Ist Super Sanita. 2015;51:131–8. 24. Sandmark H, Hogstedt C, Lewold S, Vingård E. Osteoarthrosis of the knee in men and women in association with overweight, smoking, and hormone therapy. Ann Rheum Dis. 1999;58(3):151–5. 25. Christensen BV, Iuhl IU, Vilbek H, Bülow H, Dreijer NC, Rasmussen HF. Acupuncture treatment of severe knee osteoarthrosis. A long‐term study. Acta Anaesthesiol Scand. 1992;36(6):519–25. 26. de Sire A, Agostini F, Lippi L, Mangone M, Marchese S, Cisari C, et al. Oxygen-Ozone Therapy in the Rehabilitation Field: State of the Art on Mechanisms of  Action, Safety and Effectiveness in Patients with Musculoskeletal Disorders. Biomolecules. 2021 Feb;11(3). 27. Fransen M, McCONNELL S. Land-based exercise for osteoarthritis of the knee: a metaanalysis of randomized controlled trials. J Rheumatol. 2009;36(6):1109–17. 28. Hiemstra LA, Kerslake S, Irving C. Anterior knee pain in the athlete. Clin Sports Med. 2014;33(3):437–59. 29. Filardo G, Kon E, Di Matteo B, Pelotti P, Di Martino A, Marcacci M. Platelet-rich plasma for the treatment of patellar tendinopathy: clinical and imaging findings at medium-term follow-up. Int Orthop [Internet]. 2013;37(8):1583–9. Available from: https://doi.org/10.1007/s00264-013-1972-830. Trojian TH, Concoff AL, Joy SM, Hatzenbuehler JR, Saulsberry WJ, Coleman CI. AMSSM Scientific Statement Concerning Viscosupplementation Injections for Knee  Osteoarthritis: Importance for Individual Patient Outcomes. Clin J Sport Med  Off J Can Acad  Sport Med. 2016 Jan;26(1):1–11. 31. Ammar TY, Pereira TAP, Mistura SLL, Kuhn A, Saggin JI, Lopes OV. Viscosupplementation for treating knee osteoarthrosis: review of the literature. Rev Bras Ortop. 2015;50:489–94. 32. Salini V, Pantalone A, Vanni D, Abate M. Viscosupplementation in Athletes. In: Arthroscopy and Sport Injuries. Springer; 2016. p. 433–7. 33. Erdem Y, Gul D, Akpancar S. Comparison of Intraarticular Injections of Hyaluronic Acid versus Dextrose Applied with Periarticular Prolotherapy in the Treatment of Recreational Athletes with Knee Osteoarthritis. Turkish J Sport Med. 2020;55(1):6–13. 34. Kalkhoven J, Coutts AJ, Impellizzeri FM. 'Training load error' is not a more accurate term than 'overuse' injury. Br J Sports Med [Internet]. 2020 Aug 1;54(15):934 LP – 935. Available from: http://bjsm.bmj.com/content/54/15/934.abstract35. Odole AC, Agbomeji OT, Onyeso OKK, Ojo JO, Odunaiya NA. Perspectives of Nigerian Athletes About Physiotherapy Services in Sports Injury Management: Implications for Rehabilitation. J Sport Rehabil. 2021;1(aop):1–8. 36. Hendrika W, Reswari A. The effect of physiotherapy on pain improvement in patients with early knee osteoarthritis at RSU UKI. Int J Med Heal Res. 2021;7(6):52–9. 37. MacKay C, Hawker GA, Jaglal SB. How Do Physical Therapists Approach Management of People With Early Knee Osteoarthritis? A Qualitative Study. Phys Ther [Internet]. 2019;100(2):295–306. Available from: https://doi.org/10.1093/ptj/pzz16438. Hrazdira L, Pilecki Z, Pilecki G. Ultrasound guided application of HA in the soft tissues of the locomotor system in athletes–First experience. Krátká sdělení Botek, M, Stejskal, P, Klimešová, I Vliv normobarické hypoxie na Akt Auton Nerv systému Pilot Stud 191 Drbošalová, V, Stejskal, P, Klugar, M, Krejčíř, V, Hynková, O Charakt klientů komerčn. :205. 39. Ricci M, Micheloni GM, Berti M, Perusi F, Sambugaro E, Vecchini E, et al. Clinical comparison of oral administration and viscosupplementation of hyaluronic acid (HA) in early knee osteoarthritis. Musculoskelet Surg. 2017;101(1):45–9. 40. Wang C-T, Lin Y-T, Chiang B-L, Lin Y-H, Hou S-M. High molecular weight hyaluronic acid down-regulates the gene expression of osteoarthritis-associated cytokines and enzymes in fibroblast-like synoviocytes from patients with early osteoarthritis. Osteoarthr Cartil. 2006;14(12):1237–47. 41. Irrgang JJ, Anderson AF. Development and validation of health-related quality of life measures for the knee. Clin Orthop Relat Res. 2002;402:95–109. 42. Roos EM, Roos HP, Lohmander LS, Ekdahl C, Beynnon BD. Knee Injury and Osteoarthritis Outcome Score (KOOS)—development of a self-administered outcome measure. J Orthop Sport Phys Ther. 1998;28(2):88–96. 43. Perruccio A V, Canizares M, Hawker GA, Roos EM, Maillefert JF, Lohmander SL. Comparative Validity and Responsiveness of the HOOS-PS and KOOS-PS to the WOMAC Physical Function Subscale in Total Joint Replacement for Osteoarthritis. 44. Salavati M, Mazaheri M, Negahban H, Sohani SM, Ebrahimian MR, Ebrahimi I, et al. Validation of a Persian-version of Knee injury and Osteoarthritis Outcome Score (KOOS) in Iranians with knee injuries. Osteoarthr Cartil. 2008;16(10):1178–82. 45. Higgins LD, Taylor MK, Park D, Ghodadra N, Marchant M, Pietrobon R, et al. Reliability and validity of the International Knee Documentation Committee (IKDC) subjective knee form. Jt Bone Spine. 2007;74(6):594–9. 46. Kujala UM, Kaprio J, Sarno S. Osteoarthritis of weight bearing joints of lower limbs in former elite male athletes. Bmj. 1994;308(6923):231–4. 47. Salini V, Pantalone A, Vanni D. Arthroscopy and Sport Injuries. Arthrosc Sport Inj. 2016;433–7. 48. Broglio K. Randomization in clinical trials: Permuted blocks and stratification. JAMA - J Am Med Assoc. 2018;319(21):2223–4. 49. Emanuel EJ, Wendler D, Grady C. What makes clinical research ethical? JAMA. 2000 May;283(20):2701–11. )

[36.	Hendrika W, Reswari A. The effect of physiotherapy on pain improvement in patients with early knee osteoarthritis at RSU UKI. Int J Med Heal Res. 2021;7(6):52–9.](1. Patel DR, Villalobos A. Evaluation and management of knee pain in young athletes: overuse injuries of the knee. Transl Pediatr [Internet]. 2017 Jul;6(3):190–8. Available from: https://pubmed.ncbi.nlm.nih.gov/287950102. Nicolini AP, de Carvalho RT, Matsuda MM, Filho JS, Cohen M. Common injuries in athletes' knee: Experience of a specialized center. Acta Ortop Bras. 2014;22(3):127–31. 3. Tiu T, Craig Van Dien MD. Knee overuse disorders. 4. Fuller CW, Ekstrand J, Junge A, Andersen TE, Bahr R, Dvorak J, et al. Consensus statement on injury definitions and data collection procedures in studies  of football (soccer) injuries. Br J Sports Med. 2006 Mar;40(3):193–201. 5. Majewski M, Susanne H, Klaus S. Epidemiology of athletic knee injuries: A 10-year study. Knee. 2006 Jun;13(3):184–8. 6. Wilder RP, Sethi S. Overuse injuries: tendinopathies, stress fractures, compartment syndrome, and shin  splints. Clin Sports Med. 2004 Jan;23(1):55–81, vi. 7. Aicale R, Tarantino D, Maffulli N. Overuse injuries in sport: a comprehensive overview. J Orthop Surg Res. 2018 Dec;13(1):309. 8. Yang J, Tibbetts AS, Covassin T, Cheng G, Nayar S, Heiden E. Epidemiology of overuse and acute injuries among competitive collegiate athletes. J Athl Train. 2012;47(2):198–204. 9. Bernetti A, Mangone M, Paoloni M, Di Sante L, Murgia M, Santilli V. Corticosteroid and hyaluronic acid injection therapy in tennis elbow (lateral epicondylalgia). Med Sport. 2014;67(2):289–95. 10. Iolascon G, Gimigliano F, Moretti A, De Sire A, Migliore A, Brandi ML, et al. Early osteoarthritis: How to define, diagnose, and manage. A systematic review. Eur Geriatr Med. 2017;8(5–6):383–96. 11. Drawer S, Fuller CW. Propensity for osteoarthritis and lower limb joint pain in retired professional soccer players. Br J Sports Med. 2001;35(6):402–8. 12. Vannini F, Spalding T, Andriolo L, Berruto M, Denti M, Espregueira-Mendes J, et al. Sport and early osteoarthritis: the role of sport in aetiology, progression and treatment of knee osteoarthritis. Knee Surgery, Sport Traumatol Arthrosc. 2016;24(6):1786–96. 13. Fröhlich S, Pazeller S, Cherati AS, Müller E, Frey WO, Spörri J. Overuse injuries in the knee, back and hip of top elite female alpine skiers during  the off-season preparation period: prevalence, severity and their association with traumatic preinjuries and training load. BMJ open Sport Exerc Med. 2020;6(1):e000892. 14. Rincon GA, Vyas D, Zhou J, Fu FH, Oiestad BE, Holm I, et al. \“ Knee function and prevalence of knee osteoarthritis after anterior cruciate ligament reconstruction: a prospective study with 10 to 15 years of follow-up\”. Letter to the editor. Am J Sports Med. 2011;39(4):NP3-author. 15. Kellgren JH, Lawrence J. Radiological assessment of osteo-arthrosis. Ann Rheum Dis. 1957;16(4):494. 16. Altman R, Asch E, Bloch D, Bole G, Borenstein D, Brandt K, et al. Development of criteria for the classification and reporting of osteoarthritis: classification of osteoarthritis of the knee. Arthritis Rheum Off J Am Coll Rheumatol. 1986;29(8):1039–49. 17. Luyten FP, Denti M, Filardo G, Kon E, Engebretsen L. Definition and classification of early osteoarthritis of the knee. Knee Surgery, Sport Traumatol Arthrosc. 2012;20(3):401–6. 18. Hoemann C, Kandel R, Roberts S, Saris DBF, Creemers L, Mainil-Varlet P, et al. International Cartilage Repair Society (ICRS) Recommended Guidelines for  Histological Endpoints for Cartilage Repair Studies in Animal Models and Clinical Trials. Cartilage. 2011 Apr;2(2):153–72. 19. Hunter DJ, Lo GH, Gale D, Grainger AJ, Guermazi A, Conaghan PG. The reliability of a new scoring system for knee osteoarthritis MRI and the validity of bone marrow lesion assessment: BLOKS (Boston–Leeds Osteoarthritis Knee Score). Ann Rheum Dis. 2008;67(2):206–11. 20. Peterfy CG, Guermazi A, Zaim S, Tirman PFJ, Miaux Y, White D, et al. Whole-organ magnetic resonance imaging score (WORMS) of the knee in osteoarthritis. Osteoarthr Cartil. 2004;12(3):177–90. 21. Demange MK, Sisto M, Rodeo S. Future trends for unicompartmental arthritis of the knee: injectables & stem cells. Clin Sports Med. 2014;33(1):161–74. 22. Leslie M. Knee osteoarthritis management therapies. Pain Manag Nurs. 2000;1(2):51–7. 23. Paoloni M, Bernetti A, Belelli A, Brignoli O, Buoso S, Caputi AP, et al. Appropriateness of clinical and organizational criteria for intra-articular injection therapies in osteoarthritis: A Delphi method consensus initiative among experts in Italy. Ann Ist Super Sanita. 2015;51:131–8. 24. Sandmark H, Hogstedt C, Lewold S, Vingård E. Osteoarthrosis of the knee in men and women in association with overweight, smoking, and hormone therapy. Ann Rheum Dis. 1999;58(3):151–5. 25. Christensen BV, Iuhl IU, Vilbek H, Bülow H, Dreijer NC, Rasmussen HF. Acupuncture treatment of severe knee osteoarthrosis. A long‐term study. Acta Anaesthesiol Scand. 1992;36(6):519–25. 26. de Sire A, Agostini F, Lippi L, Mangone M, Marchese S, Cisari C, et al. Oxygen-Ozone Therapy in the Rehabilitation Field: State of the Art on Mechanisms of  Action, Safety and Effectiveness in Patients with Musculoskeletal Disorders. Biomolecules. 2021 Feb;11(3). 27. Fransen M, McCONNELL S. Land-based exercise for osteoarthritis of the knee: a metaanalysis of randomized controlled trials. J Rheumatol. 2009;36(6):1109–17. 28. Hiemstra LA, Kerslake S, Irving C. Anterior knee pain in the athlete. Clin Sports Med. 2014;33(3):437–59. 29. Filardo G, Kon E, Di Matteo B, Pelotti P, Di Martino A, Marcacci M. Platelet-rich plasma for the treatment of patellar tendinopathy: clinical and imaging findings at medium-term follow-up. Int Orthop [Internet]. 2013;37(8):1583–9. Available from: https://doi.org/10.1007/s00264-013-1972-830. Trojian TH, Concoff AL, Joy SM, Hatzenbuehler JR, Saulsberry WJ, Coleman CI. AMSSM Scientific Statement Concerning Viscosupplementation Injections for Knee  Osteoarthritis: Importance for Individual Patient Outcomes. Clin J Sport Med  Off J Can Acad  Sport Med. 2016 Jan;26(1):1–11. 31. Ammar TY, Pereira TAP, Mistura SLL, Kuhn A, Saggin JI, Lopes OV. Viscosupplementation for treating knee osteoarthrosis: review of the literature. Rev Bras Ortop. 2015;50:489–94. 32. Salini V, Pantalone A, Vanni D, Abate M. Viscosupplementation in Athletes. In: Arthroscopy and Sport Injuries. Springer; 2016. p. 433–7. 33. Erdem Y, Gul D, Akpancar S. Comparison of Intraarticular Injections of Hyaluronic Acid versus Dextrose Applied with Periarticular Prolotherapy in the Treatment of Recreational Athletes with Knee Osteoarthritis. Turkish J Sport Med. 2020;55(1):6–13. 34. Kalkhoven J, Coutts AJ, Impellizzeri FM. 'Training load error' is not a more accurate term than 'overuse' injury. Br J Sports Med [Internet]. 2020 Aug 1;54(15):934 LP – 935. Available from: http://bjsm.bmj.com/content/54/15/934.abstract35. Odole AC, Agbomeji OT, Onyeso OKK, Ojo JO, Odunaiya NA. Perspectives of Nigerian Athletes About Physiotherapy Services in Sports Injury Management: Implications for Rehabilitation. J Sport Rehabil. 2021;1(aop):1–8. 36. Hendrika W, Reswari A. The effect of physiotherapy on pain improvement in patients with early knee osteoarthritis at RSU UKI. Int J Med Heal Res. 2021;7(6):52–9. 37. MacKay C, Hawker GA, Jaglal SB. How Do Physical Therapists Approach Management of People With Early Knee Osteoarthritis? A Qualitative Study. Phys Ther [Internet]. 2019;100(2):295–306. Available from: https://doi.org/10.1093/ptj/pzz16438. Hrazdira L, Pilecki Z, Pilecki G. Ultrasound guided application of HA in the soft tissues of the locomotor system in athletes–First experience. Krátká sdělení Botek, M, Stejskal, P, Klimešová, I Vliv normobarické hypoxie na Akt Auton Nerv systému Pilot Stud 191 Drbošalová, V, Stejskal, P, Klugar, M, Krejčíř, V, Hynková, O Charakt klientů komerčn. :205. 39. Ricci M, Micheloni GM, Berti M, Perusi F, Sambugaro E, Vecchini E, et al. Clinical comparison of oral administration and viscosupplementation of hyaluronic acid (HA) in early knee osteoarthritis. Musculoskelet Surg. 2017;101(1):45–9. 40. Wang C-T, Lin Y-T, Chiang B-L, Lin Y-H, Hou S-M. High molecular weight hyaluronic acid down-regulates the gene expression of osteoarthritis-associated cytokines and enzymes in fibroblast-like synoviocytes from patients with early osteoarthritis. Osteoarthr Cartil. 2006;14(12):1237–47. 41. Irrgang JJ, Anderson AF. Development and validation of health-related quality of life measures for the knee. Clin Orthop Relat Res. 2002;402:95–109. 42. Roos EM, Roos HP, Lohmander LS, Ekdahl C, Beynnon BD. Knee Injury and Osteoarthritis Outcome Score (KOOS)—development of a self-administered outcome measure. J Orthop Sport Phys Ther. 1998;28(2):88–96. 43. Perruccio A V, Canizares M, Hawker GA, Roos EM, Maillefert JF, Lohmander SL. Comparative Validity and Responsiveness of the HOOS-PS and KOOS-PS to the WOMAC Physical Function Subscale in Total Joint Replacement for Osteoarthritis. 44. Salavati M, Mazaheri M, Negahban H, Sohani SM, Ebrahimian MR, Ebrahimi I, et al. Validation of a Persian-version of Knee injury and Osteoarthritis Outcome Score (KOOS) in Iranians with knee injuries. Osteoarthr Cartil. 2008;16(10):1178–82. 45. Higgins LD, Taylor MK, Park D, Ghodadra N, Marchant M, Pietrobon R, et al. Reliability and validity of the International Knee Documentation Committee (IKDC) subjective knee form. Jt Bone Spine. 2007;74(6):594–9. 46. Kujala UM, Kaprio J, Sarno S. Osteoarthritis of weight bearing joints of lower limbs in former elite male athletes. Bmj. 1994;308(6923):231–4. 47. Salini V, Pantalone A, Vanni D. Arthroscopy and Sport Injuries. Arthrosc Sport Inj. 2016;433–7. 48. Broglio K. Randomization in clinical trials: Permuted blocks and stratification. JAMA - J Am Med Assoc. 2018;319(21):2223–4. 49. Emanuel EJ, Wendler D, Grady C. What makes clinical research ethical? JAMA. 2000 May;283(20):2701–11. )

[37.	MacKay C, Hawker GA, Jaglal SB. How Do Physical Therapists Approach Management of People With Early Knee Osteoarthritis? A Qualitative Study. Phys Ther [Internet]. 2019;100(2):295–306. Available from: https://doi.org/10.1093/ptj/pzz164](1. Patel DR, Villalobos A. Evaluation and management of knee pain in young athletes: overuse injuries of the knee. Transl Pediatr [Internet]. 2017 Jul;6(3):190–8. Available from: https://pubmed.ncbi.nlm.nih.gov/287950102. Nicolini AP, de Carvalho RT, Matsuda MM, Filho JS, Cohen M. Common injuries in athletes' knee: Experience of a specialized center. Acta Ortop Bras. 2014;22(3):127–31. 3. Tiu T, Craig Van Dien MD. Knee overuse disorders. 4. Fuller CW, Ekstrand J, Junge A, Andersen TE, Bahr R, Dvorak J, et al. Consensus statement on injury definitions and data collection procedures in studies  of football (soccer) injuries. Br J Sports Med. 2006 Mar;40(3):193–201. 5. Majewski M, Susanne H, Klaus S. Epidemiology of athletic knee injuries: A 10-year study. Knee. 2006 Jun;13(3):184–8. 6. Wilder RP, Sethi S. Overuse injuries: tendinopathies, stress fractures, compartment syndrome, and shin  splints. Clin Sports Med. 2004 Jan;23(1):55–81, vi. 7. Aicale R, Tarantino D, Maffulli N. Overuse injuries in sport: a comprehensive overview. J Orthop Surg Res. 2018 Dec;13(1):309. 8. Yang J, Tibbetts AS, Covassin T, Cheng G, Nayar S, Heiden E. Epidemiology of overuse and acute injuries among competitive collegiate athletes. J Athl Train. 2012;47(2):198–204. 9. Bernetti A, Mangone M, Paoloni M, Di Sante L, Murgia M, Santilli V. Corticosteroid and hyaluronic acid injection therapy in tennis elbow (lateral epicondylalgia). Med Sport. 2014;67(2):289–95. 10. Iolascon G, Gimigliano F, Moretti A, De Sire A, Migliore A, Brandi ML, et al. Early osteoarthritis: How to define, diagnose, and manage. A systematic review. Eur Geriatr Med. 2017;8(5–6):383–96. 11. Drawer S, Fuller CW. Propensity for osteoarthritis and lower limb joint pain in retired professional soccer players. Br J Sports Med. 2001;35(6):402–8. 12. Vannini F, Spalding T, Andriolo L, Berruto M, Denti M, Espregueira-Mendes J, et al. Sport and early osteoarthritis: the role of sport in aetiology, progression and treatment of knee osteoarthritis. Knee Surgery, Sport Traumatol Arthrosc. 2016;24(6):1786–96. 13. Fröhlich S, Pazeller S, Cherati AS, Müller E, Frey WO, Spörri J. Overuse injuries in the knee, back and hip of top elite female alpine skiers during  the off-season preparation period: prevalence, severity and their association with traumatic preinjuries and training load. BMJ open Sport Exerc Med. 2020;6(1):e000892. 14. Rincon GA, Vyas D, Zhou J, Fu FH, Oiestad BE, Holm I, et al. \“ Knee function and prevalence of knee osteoarthritis after anterior cruciate ligament reconstruction: a prospective study with 10 to 15 years of follow-up\”. Letter to the editor. Am J Sports Med. 2011;39(4):NP3-author. 15. Kellgren JH, Lawrence J. Radiological assessment of osteo-arthrosis. Ann Rheum Dis. 1957;16(4):494. 16. Altman R, Asch E, Bloch D, Bole G, Borenstein D, Brandt K, et al. Development of criteria for the classification and reporting of osteoarthritis: classification of osteoarthritis of the knee. Arthritis Rheum Off J Am Coll Rheumatol. 1986;29(8):1039–49. 17. Luyten FP, Denti M, Filardo G, Kon E, Engebretsen L. Definition and classification of early osteoarthritis of the knee. Knee Surgery, Sport Traumatol Arthrosc. 2012;20(3):401–6. 18. Hoemann C, Kandel R, Roberts S, Saris DBF, Creemers L, Mainil-Varlet P, et al. International Cartilage Repair Society (ICRS) Recommended Guidelines for  Histological Endpoints for Cartilage Repair Studies in Animal Models and Clinical Trials. Cartilage. 2011 Apr;2(2):153–72. 19. Hunter DJ, Lo GH, Gale D, Grainger AJ, Guermazi A, Conaghan PG. The reliability of a new scoring system for knee osteoarthritis MRI and the validity of bone marrow lesion assessment: BLOKS (Boston–Leeds Osteoarthritis Knee Score). Ann Rheum Dis. 2008;67(2):206–11. 20. Peterfy CG, Guermazi A, Zaim S, Tirman PFJ, Miaux Y, White D, et al. Whole-organ magnetic resonance imaging score (WORMS) of the knee in osteoarthritis. Osteoarthr Cartil. 2004;12(3):177–90. 21. Demange MK, Sisto M, Rodeo S. Future trends for unicompartmental arthritis of the knee: injectables & stem cells. Clin Sports Med. 2014;33(1):161–74. 22. Leslie M. Knee osteoarthritis management therapies. Pain Manag Nurs. 2000;1(2):51–7. 23. Paoloni M, Bernetti A, Belelli A, Brignoli O, Buoso S, Caputi AP, et al. Appropriateness of clinical and organizational criteria for intra-articular injection therapies in osteoarthritis: A Delphi method consensus initiative among experts in Italy. Ann Ist Super Sanita. 2015;51:131–8. 24. Sandmark H, Hogstedt C, Lewold S, Vingård E. Osteoarthrosis of the knee in men and women in association with overweight, smoking, and hormone therapy. Ann Rheum Dis. 1999;58(3):151–5. 25. Christensen BV, Iuhl IU, Vilbek H, Bülow H, Dreijer NC, Rasmussen HF. Acupuncture treatment of severe knee osteoarthrosis. A long‐term study. Acta Anaesthesiol Scand. 1992;36(6):519–25. 26. de Sire A, Agostini F, Lippi L, Mangone M, Marchese S, Cisari C, et al. Oxygen-Ozone Therapy in the Rehabilitation Field: State of the Art on Mechanisms of  Action, Safety and Effectiveness in Patients with Musculoskeletal Disorders. Biomolecules. 2021 Feb;11(3). 27. Fransen M, McCONNELL S. Land-based exercise for osteoarthritis of the knee: a metaanalysis of randomized controlled trials. J Rheumatol. 2009;36(6):1109–17. 28. Hiemstra LA, Kerslake S, Irving C. Anterior knee pain in the athlete. Clin Sports Med. 2014;33(3):437–59. 29. Filardo G, Kon E, Di Matteo B, Pelotti P, Di Martino A, Marcacci M. Platelet-rich plasma for the treatment of patellar tendinopathy: clinical and imaging findings at medium-term follow-up. Int Orthop [Internet]. 2013;37(8):1583–9. Available from: https://doi.org/10.1007/s00264-013-1972-830. Trojian TH, Concoff AL, Joy SM, Hatzenbuehler JR, Saulsberry WJ, Coleman CI. AMSSM Scientific Statement Concerning Viscosupplementation Injections for Knee  Osteoarthritis: Importance for Individual Patient Outcomes. Clin J Sport Med  Off J Can Acad  Sport Med. 2016 Jan;26(1):1–11. 31. Ammar TY, Pereira TAP, Mistura SLL, Kuhn A, Saggin JI, Lopes OV. Viscosupplementation for treating knee osteoarthrosis: review of the literature. Rev Bras Ortop. 2015;50:489–94. 32. Salini V, Pantalone A, Vanni D, Abate M. Viscosupplementation in Athletes. In: Arthroscopy and Sport Injuries. Springer; 2016. p. 433–7. 33. Erdem Y, Gul D, Akpancar S. Comparison of Intraarticular Injections of Hyaluronic Acid versus Dextrose Applied with Periarticular Prolotherapy in the Treatment of Recreational Athletes with Knee Osteoarthritis. Turkish J Sport Med. 2020;55(1):6–13. 34. Kalkhoven J, Coutts AJ, Impellizzeri FM. 'Training load error' is not a more accurate term than 'overuse' injury. Br J Sports Med [Internet]. 2020 Aug 1;54(15):934 LP – 935. Available from: http://bjsm.bmj.com/content/54/15/934.abstract35. Odole AC, Agbomeji OT, Onyeso OKK, Ojo JO, Odunaiya NA. Perspectives of Nigerian Athletes About Physiotherapy Services in Sports Injury Management: Implications for Rehabilitation. J Sport Rehabil. 2021;1(aop):1–8. 36. Hendrika W, Reswari A. The effect of physiotherapy on pain improvement in patients with early knee osteoarthritis at RSU UKI. Int J Med Heal Res. 2021;7(6):52–9. 37. MacKay C, Hawker GA, Jaglal SB. How Do Physical Therapists Approach Management of People With Early Knee Osteoarthritis? A Qualitative Study. Phys Ther [Internet]. 2019;100(2):295–306. Available from: https://doi.org/10.1093/ptj/pzz16438. Hrazdira L, Pilecki Z, Pilecki G. Ultrasound guided application of HA in the soft tissues of the locomotor system in athletes–First experience. Krátká sdělení Botek, M, Stejskal, P, Klimešová, I Vliv normobarické hypoxie na Akt Auton Nerv systému Pilot Stud 191 Drbošalová, V, Stejskal, P, Klugar, M, Krejčíř, V, Hynková, O Charakt klientů komerčn. :205. 39. Ricci M, Micheloni GM, Berti M, Perusi F, Sambugaro E, Vecchini E, et al. Clinical comparison of oral administration and viscosupplementation of hyaluronic acid (HA) in early knee osteoarthritis. Musculoskelet Surg. 2017;101(1):45–9. 40. Wang C-T, Lin Y-T, Chiang B-L, Lin Y-H, Hou S-M. High molecular weight hyaluronic acid down-regulates the gene expression of osteoarthritis-associated cytokines and enzymes in fibroblast-like synoviocytes from patients with early osteoarthritis. Osteoarthr Cartil. 2006;14(12):1237–47. 41. Irrgang JJ, Anderson AF. Development and validation of health-related quality of life measures for the knee. Clin Orthop Relat Res. 2002;402:95–109. 42. Roos EM, Roos HP, Lohmander LS, Ekdahl C, Beynnon BD. Knee Injury and Osteoarthritis Outcome Score (KOOS)—development of a self-administered outcome measure. J Orthop Sport Phys Ther. 1998;28(2):88–96. 43. Perruccio A V, Canizares M, Hawker GA, Roos EM, Maillefert JF, Lohmander SL. Comparative Validity and Responsiveness of the HOOS-PS and KOOS-PS to the WOMAC Physical Function Subscale in Total Joint Replacement for Osteoarthritis. 44. Salavati M, Mazaheri M, Negahban H, Sohani SM, Ebrahimian MR, Ebrahimi I, et al. Validation of a Persian-version of Knee injury and Osteoarthritis Outcome Score (KOOS) in Iranians with knee injuries. Osteoarthr Cartil. 2008;16(10):1178–82. 45. Higgins LD, Taylor MK, Park D, Ghodadra N, Marchant M, Pietrobon R, et al. Reliability and validity of the International Knee Documentation Committee (IKDC) subjective knee form. Jt Bone Spine. 2007;74(6):594–9. 46. Kujala UM, Kaprio J, Sarno S. Osteoarthritis of weight bearing joints of lower limbs in former elite male athletes. Bmj. 1994;308(6923):231–4. 47. Salini V, Pantalone A, Vanni D. Arthroscopy and Sport Injuries. Arthrosc Sport Inj. 2016;433–7. 48. Broglio K. Randomization in clinical trials: Permuted blocks and stratification. JAMA - J Am Med Assoc. 2018;319(21):2223–4. 49. Emanuel EJ, Wendler D, Grady C. What makes clinical research ethical? JAMA. 2000 May;283(20):2701–11. )

[38.	Hrazdira L, Pilecki Z, Pilecki G. Ultrasound guided application of HA in the soft tissues of the locomotor system in athletes–First experience. Krátká sdělení Botek, M, Stejskal, P, Klimešová, I Vliv normobarické hypoxie na Akt Auton Nerv systému Pilot Stud 191 Drbošalová, V, Stejskal, P, Klugar, M, Krejčíř, V, Hynková, O Charakt klientů komerčn. :205.](1. Patel DR, Villalobos A. Evaluation and management of knee pain in young athletes: overuse injuries of the knee. Transl Pediatr [Internet]. 2017 Jul;6(3):190–8. Available from: https://pubmed.ncbi.nlm.nih.gov/287950102. Nicolini AP, de Carvalho RT, Matsuda MM, Filho JS, Cohen M. Common injuries in athletes' knee: Experience of a specialized center. Acta Ortop Bras. 2014;22(3):127–31. 3. Tiu T, Craig Van Dien MD. Knee overuse disorders. 4. Fuller CW, Ekstrand J, Junge A, Andersen TE, Bahr R, Dvorak J, et al. Consensus statement on injury definitions and data collection procedures in studies  of football (soccer) injuries. Br J Sports Med. 2006 Mar;40(3):193–201. 5. Majewski M, Susanne H, Klaus S. Epidemiology of athletic knee injuries: A 10-year study. Knee. 2006 Jun;13(3):184–8. 6. Wilder RP, Sethi S. Overuse injuries: tendinopathies, stress fractures, compartment syndrome, and shin  splints. Clin Sports Med. 2004 Jan;23(1):55–81, vi. 7. Aicale R, Tarantino D, Maffulli N. Overuse injuries in sport: a comprehensive overview. J Orthop Surg Res. 2018 Dec;13(1):309. 8. Yang J, Tibbetts AS, Covassin T, Cheng G, Nayar S, Heiden E. Epidemiology of overuse and acute injuries among competitive collegiate athletes. J Athl Train. 2012;47(2):198–204. 9. Bernetti A, Mangone M, Paoloni M, Di Sante L, Murgia M, Santilli V. Corticosteroid and hyaluronic acid injection therapy in tennis elbow (lateral epicondylalgia). Med Sport. 2014;67(2):289–95. 10. Iolascon G, Gimigliano F, Moretti A, De Sire A, Migliore A, Brandi ML, et al. Early osteoarthritis: How to define, diagnose, and manage. A systematic review. Eur Geriatr Med. 2017;8(5–6):383–96. 11. Drawer S, Fuller CW. Propensity for osteoarthritis and lower limb joint pain in retired professional soccer players. Br J Sports Med. 2001;35(6):402–8. 12. Vannini F, Spalding T, Andriolo L, Berruto M, Denti M, Espregueira-Mendes J, et al. Sport and early osteoarthritis: the role of sport in aetiology, progression and treatment of knee osteoarthritis. Knee Surgery, Sport Traumatol Arthrosc. 2016;24(6):1786–96. 13. Fröhlich S, Pazeller S, Cherati AS, Müller E, Frey WO, Spörri J. Overuse injuries in the knee, back and hip of top elite female alpine skiers during  the off-season preparation period: prevalence, severity and their association with traumatic preinjuries and training load. BMJ open Sport Exerc Med. 2020;6(1):e000892. 14. Rincon GA, Vyas D, Zhou J, Fu FH, Oiestad BE, Holm I, et al. \“ Knee function and prevalence of knee osteoarthritis after anterior cruciate ligament reconstruction: a prospective study with 10 to 15 years of follow-up\”. Letter to the editor. Am J Sports Med. 2011;39(4):NP3-author. 15. Kellgren JH, Lawrence J. Radiological assessment of osteo-arthrosis. Ann Rheum Dis. 1957;16(4):494. 16. Altman R, Asch E, Bloch D, Bole G, Borenstein D, Brandt K, et al. Development of criteria for the classification and reporting of osteoarthritis: classification of osteoarthritis of the knee. Arthritis Rheum Off J Am Coll Rheumatol. 1986;29(8):1039–49. 17. Luyten FP, Denti M, Filardo G, Kon E, Engebretsen L. Definition and classification of early osteoarthritis of the knee. Knee Surgery, Sport Traumatol Arthrosc. 2012;20(3):401–6. 18. Hoemann C, Kandel R, Roberts S, Saris DBF, Creemers L, Mainil-Varlet P, et al. International Cartilage Repair Society (ICRS) Recommended Guidelines for  Histological Endpoints for Cartilage Repair Studies in Animal Models and Clinical Trials. Cartilage. 2011 Apr;2(2):153–72. 19. Hunter DJ, Lo GH, Gale D, Grainger AJ, Guermazi A, Conaghan PG. The reliability of a new scoring system for knee osteoarthritis MRI and the validity of bone marrow lesion assessment: BLOKS (Boston–Leeds Osteoarthritis Knee Score). Ann Rheum Dis. 2008;67(2):206–11. 20. Peterfy CG, Guermazi A, Zaim S, Tirman PFJ, Miaux Y, White D, et al. Whole-organ magnetic resonance imaging score (WORMS) of the knee in osteoarthritis. Osteoarthr Cartil. 2004;12(3):177–90. 21. Demange MK, Sisto M, Rodeo S. Future trends for unicompartmental arthritis of the knee: injectables & stem cells. Clin Sports Med. 2014;33(1):161–74. 22. Leslie M. Knee osteoarthritis management therapies. Pain Manag Nurs. 2000;1(2):51–7. 23. Paoloni M, Bernetti A, Belelli A, Brignoli O, Buoso S, Caputi AP, et al. Appropriateness of clinical and organizational criteria for intra-articular injection therapies in osteoarthritis: A Delphi method consensus initiative among experts in Italy. Ann Ist Super Sanita. 2015;51:131–8. 24. Sandmark H, Hogstedt C, Lewold S, Vingård E. Osteoarthrosis of the knee in men and women in association with overweight, smoking, and hormone therapy. Ann Rheum Dis. 1999;58(3):151–5. 25. Christensen BV, Iuhl IU, Vilbek H, Bülow H, Dreijer NC, Rasmussen HF. Acupuncture treatment of severe knee osteoarthrosis. A long‐term study. Acta Anaesthesiol Scand. 1992;36(6):519–25. 26. de Sire A, Agostini F, Lippi L, Mangone M, Marchese S, Cisari C, et al. Oxygen-Ozone Therapy in the Rehabilitation Field: State of the Art on Mechanisms of  Action, Safety and Effectiveness in Patients with Musculoskeletal Disorders. Biomolecules. 2021 Feb;11(3). 27. Fransen M, McCONNELL S. Land-based exercise for osteoarthritis of the knee: a metaanalysis of randomized controlled trials. J Rheumatol. 2009;36(6):1109–17. 28. Hiemstra LA, Kerslake S, Irving C. Anterior knee pain in the athlete. Clin Sports Med. 2014;33(3):437–59. 29. Filardo G, Kon E, Di Matteo B, Pelotti P, Di Martino A, Marcacci M. Platelet-rich plasma for the treatment of patellar tendinopathy: clinical and imaging findings at medium-term follow-up. Int Orthop [Internet]. 2013;37(8):1583–9. Available from: https://doi.org/10.1007/s00264-013-1972-830. Trojian TH, Concoff AL, Joy SM, Hatzenbuehler JR, Saulsberry WJ, Coleman CI. AMSSM Scientific Statement Concerning Viscosupplementation Injections for Knee  Osteoarthritis: Importance for Individual Patient Outcomes. Clin J Sport Med  Off J Can Acad  Sport Med. 2016 Jan;26(1):1–11. 31. Ammar TY, Pereira TAP, Mistura SLL, Kuhn A, Saggin JI, Lopes OV. Viscosupplementation for treating knee osteoarthrosis: review of the literature. Rev Bras Ortop. 2015;50:489–94. 32. Salini V, Pantalone A, Vanni D, Abate M. Viscosupplementation in Athletes. In: Arthroscopy and Sport Injuries. Springer; 2016. p. 433–7. 33. Erdem Y, Gul D, Akpancar S. Comparison of Intraarticular Injections of Hyaluronic Acid versus Dextrose Applied with Periarticular Prolotherapy in the Treatment of Recreational Athletes with Knee Osteoarthritis. Turkish J Sport Med. 2020;55(1):6–13. 34. Kalkhoven J, Coutts AJ, Impellizzeri FM. 'Training load error' is not a more accurate term than 'overuse' injury. Br J Sports Med [Internet]. 2020 Aug 1;54(15):934 LP – 935. Available from: http://bjsm.bmj.com/content/54/15/934.abstract35. Odole AC, Agbomeji OT, Onyeso OKK, Ojo JO, Odunaiya NA. Perspectives of Nigerian Athletes About Physiotherapy Services in Sports Injury Management: Implications for Rehabilitation. J Sport Rehabil. 2021;1(aop):1–8. 36. Hendrika W, Reswari A. The effect of physiotherapy on pain improvement in patients with early knee osteoarthritis at RSU UKI. Int J Med Heal Res. 2021;7(6):52–9. 37. MacKay C, Hawker GA, Jaglal SB. How Do Physical Therapists Approach Management of People With Early Knee Osteoarthritis? A Qualitative Study. Phys Ther [Internet]. 2019;100(2):295–306. Available from: https://doi.org/10.1093/ptj/pzz16438. Hrazdira L, Pilecki Z, Pilecki G. Ultrasound guided application of HA in the soft tissues of the locomotor system in athletes–First experience. Krátká sdělení Botek, M, Stejskal, P, Klimešová, I Vliv normobarické hypoxie na Akt Auton Nerv systému Pilot Stud 191 Drbošalová, V, Stejskal, P, Klugar, M, Krejčíř, V, Hynková, O Charakt klientů komerčn. :205. 39. Ricci M, Micheloni GM, Berti M, Perusi F, Sambugaro E, Vecchini E, et al. Clinical comparison of oral administration and viscosupplementation of hyaluronic acid (HA) in early knee osteoarthritis. Musculoskelet Surg. 2017;101(1):45–9. 40. Wang C-T, Lin Y-T, Chiang B-L, Lin Y-H, Hou S-M. High molecular weight hyaluronic acid down-regulates the gene expression of osteoarthritis-associated cytokines and enzymes in fibroblast-like synoviocytes from patients with early osteoarthritis. Osteoarthr Cartil. 2006;14(12):1237–47. 41. Irrgang JJ, Anderson AF. Development and validation of health-related quality of life measures for the knee. Clin Orthop Relat Res. 2002;402:95–109. 42. Roos EM, Roos HP, Lohmander LS, Ekdahl C, Beynnon BD. Knee Injury and Osteoarthritis Outcome Score (KOOS)—development of a self-administered outcome measure. J Orthop Sport Phys Ther. 1998;28(2):88–96. 43. Perruccio A V, Canizares M, Hawker GA, Roos EM, Maillefert JF, Lohmander SL. Comparative Validity and Responsiveness of the HOOS-PS and KOOS-PS to the WOMAC Physical Function Subscale in Total Joint Replacement for Osteoarthritis. 44. Salavati M, Mazaheri M, Negahban H, Sohani SM, Ebrahimian MR, Ebrahimi I, et al. Validation of a Persian-version of Knee injury and Osteoarthritis Outcome Score (KOOS) in Iranians with knee injuries. Osteoarthr Cartil. 2008;16(10):1178–82. 45. Higgins LD, Taylor MK, Park D, Ghodadra N, Marchant M, Pietrobon R, et al. Reliability and validity of the International Knee Documentation Committee (IKDC) subjective knee form. Jt Bone Spine. 2007;74(6):594–9. 46. Kujala UM, Kaprio J, Sarno S. Osteoarthritis of weight bearing joints of lower limbs in former elite male athletes. Bmj. 1994;308(6923):231–4. 47. Salini V, Pantalone A, Vanni D. Arthroscopy and Sport Injuries. Arthrosc Sport Inj. 2016;433–7. 48. Broglio K. Randomization in clinical trials: Permuted blocks and stratification. JAMA - J Am Med Assoc. 2018;319(21):2223–4. 49. Emanuel EJ, Wendler D, Grady C. What makes clinical research ethical? JAMA. 2000 May;283(20):2701–11. )

[39.	Ricci M, Micheloni GM, Berti M, Perusi F, Sambugaro E, Vecchini E, et al. Clinical comparison of oral administration and viscosupplementation of hyaluronic acid (HA) in early knee osteoarthritis. Musculoskelet Surg. 2017;101(1):45–9.](1. Patel DR, Villalobos A. Evaluation and management of knee pain in young athletes: overuse injuries of the knee. Transl Pediatr [Internet]. 2017 Jul;6(3):190–8. Available from: https://pubmed.ncbi.nlm.nih.gov/287950102. Nicolini AP, de Carvalho RT, Matsuda MM, Filho JS, Cohen M. Common injuries in athletes' knee: Experience of a specialized center. Acta Ortop Bras. 2014;22(3):127–31. 3. Tiu T, Craig Van Dien MD. Knee overuse disorders. 4. Fuller CW, Ekstrand J, Junge A, Andersen TE, Bahr R, Dvorak J, et al. Consensus statement on injury definitions and data collection procedures in studies  of football (soccer) injuries. Br J Sports Med. 2006 Mar;40(3):193–201. 5. Majewski M, Susanne H, Klaus S. Epidemiology of athletic knee injuries: A 10-year study. Knee. 2006 Jun;13(3):184–8. 6. Wilder RP, Sethi S. Overuse injuries: tendinopathies, stress fractures, compartment syndrome, and shin  splints. Clin Sports Med. 2004 Jan;23(1):55–81, vi. 7. Aicale R, Tarantino D, Maffulli N. Overuse injuries in sport: a comprehensive overview. J Orthop Surg Res. 2018 Dec;13(1):309. 8. Yang J, Tibbetts AS, Covassin T, Cheng G, Nayar S, Heiden E. Epidemiology of overuse and acute injuries among competitive collegiate athletes. J Athl Train. 2012;47(2):198–204. 9. Bernetti A, Mangone M, Paoloni M, Di Sante L, Murgia M, Santilli V. Corticosteroid and hyaluronic acid injection therapy in tennis elbow (lateral epicondylalgia). Med Sport. 2014;67(2):289–95. 10. Iolascon G, Gimigliano F, Moretti A, De Sire A, Migliore A, Brandi ML, et al. Early osteoarthritis: How to define, diagnose, and manage. A systematic review. Eur Geriatr Med. 2017;8(5–6):383–96. 11. Drawer S, Fuller CW. Propensity for osteoarthritis and lower limb joint pain in retired professional soccer players. Br J Sports Med. 2001;35(6):402–8. 12. Vannini F, Spalding T, Andriolo L, Berruto M, Denti M, Espregueira-Mendes J, et al. Sport and early osteoarthritis: the role of sport in aetiology, progression and treatment of knee osteoarthritis. Knee Surgery, Sport Traumatol Arthrosc. 2016;24(6):1786–96. 13. Fröhlich S, Pazeller S, Cherati AS, Müller E, Frey WO, Spörri J. Overuse injuries in the knee, back and hip of top elite female alpine skiers during  the off-season preparation period: prevalence, severity and their association with traumatic preinjuries and training load. BMJ open Sport Exerc Med. 2020;6(1):e000892. 14. Rincon GA, Vyas D, Zhou J, Fu FH, Oiestad BE, Holm I, et al. \“ Knee function and prevalence of knee osteoarthritis after anterior cruciate ligament reconstruction: a prospective study with 10 to 15 years of follow-up\”. Letter to the editor. Am J Sports Med. 2011;39(4):NP3-author. 15. Kellgren JH, Lawrence J. Radiological assessment of osteo-arthrosis. Ann Rheum Dis. 1957;16(4):494. 16. Altman R, Asch E, Bloch D, Bole G, Borenstein D, Brandt K, et al. Development of criteria for the classification and reporting of osteoarthritis: classification of osteoarthritis of the knee. Arthritis Rheum Off J Am Coll Rheumatol. 1986;29(8):1039–49. 17. Luyten FP, Denti M, Filardo G, Kon E, Engebretsen L. Definition and classification of early osteoarthritis of the knee. Knee Surgery, Sport Traumatol Arthrosc. 2012;20(3):401–6. 18. Hoemann C, Kandel R, Roberts S, Saris DBF, Creemers L, Mainil-Varlet P, et al. International Cartilage Repair Society (ICRS) Recommended Guidelines for  Histological Endpoints for Cartilage Repair Studies in Animal Models and Clinical Trials. Cartilage. 2011 Apr;2(2):153–72. 19. Hunter DJ, Lo GH, Gale D, Grainger AJ, Guermazi A, Conaghan PG. The reliability of a new scoring system for knee osteoarthritis MRI and the validity of bone marrow lesion assessment: BLOKS (Boston–Leeds Osteoarthritis Knee Score). Ann Rheum Dis. 2008;67(2):206–11. 20. Peterfy CG, Guermazi A, Zaim S, Tirman PFJ, Miaux Y, White D, et al. Whole-organ magnetic resonance imaging score (WORMS) of the knee in osteoarthritis. Osteoarthr Cartil. 2004;12(3):177–90. 21. Demange MK, Sisto M, Rodeo S. Future trends for unicompartmental arthritis of the knee: injectables & stem cells. Clin Sports Med. 2014;33(1):161–74. 22. Leslie M. Knee osteoarthritis management therapies. Pain Manag Nurs. 2000;1(2):51–7. 23. Paoloni M, Bernetti A, Belelli A, Brignoli O, Buoso S, Caputi AP, et al. Appropriateness of clinical and organizational criteria for intra-articular injection therapies in osteoarthritis: A Delphi method consensus initiative among experts in Italy. Ann Ist Super Sanita. 2015;51:131–8. 24. Sandmark H, Hogstedt C, Lewold S, Vingård E. Osteoarthrosis of the knee in men and women in association with overweight, smoking, and hormone therapy. Ann Rheum Dis. 1999;58(3):151–5. 25. Christensen BV, Iuhl IU, Vilbek H, Bülow H, Dreijer NC, Rasmussen HF. Acupuncture treatment of severe knee osteoarthrosis. A long‐term study. Acta Anaesthesiol Scand. 1992;36(6):519–25. 26. de Sire A, Agostini F, Lippi L, Mangone M, Marchese S, Cisari C, et al. Oxygen-Ozone Therapy in the Rehabilitation Field: State of the Art on Mechanisms of  Action, Safety and Effectiveness in Patients with Musculoskeletal Disorders. Biomolecules. 2021 Feb;11(3). 27. Fransen M, McCONNELL S. Land-based exercise for osteoarthritis of the knee: a metaanalysis of randomized controlled trials. J Rheumatol. 2009;36(6):1109–17. 28. Hiemstra LA, Kerslake S, Irving C. Anterior knee pain in the athlete. Clin Sports Med. 2014;33(3):437–59. 29. Filardo G, Kon E, Di Matteo B, Pelotti P, Di Martino A, Marcacci M. Platelet-rich plasma for the treatment of patellar tendinopathy: clinical and imaging findings at medium-term follow-up. Int Orthop [Internet]. 2013;37(8):1583–9. Available from: https://doi.org/10.1007/s00264-013-1972-830. Trojian TH, Concoff AL, Joy SM, Hatzenbuehler JR, Saulsberry WJ, Coleman CI. AMSSM Scientific Statement Concerning Viscosupplementation Injections for Knee  Osteoarthritis: Importance for Individual Patient Outcomes. Clin J Sport Med  Off J Can Acad  Sport Med. 2016 Jan;26(1):1–11. 31. Ammar TY, Pereira TAP, Mistura SLL, Kuhn A, Saggin JI, Lopes OV. Viscosupplementation for treating knee osteoarthrosis: review of the literature. Rev Bras Ortop. 2015;50:489–94. 32. Salini V, Pantalone A, Vanni D, Abate M. Viscosupplementation in Athletes. In: Arthroscopy and Sport Injuries. Springer; 2016. p. 433–7. 33. Erdem Y, Gul D, Akpancar S. Comparison of Intraarticular Injections of Hyaluronic Acid versus Dextrose Applied with Periarticular Prolotherapy in the Treatment of Recreational Athletes with Knee Osteoarthritis. Turkish J Sport Med. 2020;55(1):6–13. 34. Kalkhoven J, Coutts AJ, Impellizzeri FM. 'Training load error' is not a more accurate term than 'overuse' injury. Br J Sports Med [Internet]. 2020 Aug 1;54(15):934 LP – 935. Available from: http://bjsm.bmj.com/content/54/15/934.abstract35. Odole AC, Agbomeji OT, Onyeso OKK, Ojo JO, Odunaiya NA. Perspectives of Nigerian Athletes About Physiotherapy Services in Sports Injury Management: Implications for Rehabilitation. J Sport Rehabil. 2021;1(aop):1–8. 36. Hendrika W, Reswari A. The effect of physiotherapy on pain improvement in patients with early knee osteoarthritis at RSU UKI. Int J Med Heal Res. 2021;7(6):52–9. 37. MacKay C, Hawker GA, Jaglal SB. How Do Physical Therapists Approach Management of People With Early Knee Osteoarthritis? A Qualitative Study. Phys Ther [Internet]. 2019;100(2):295–306. Available from: https://doi.org/10.1093/ptj/pzz16438. Hrazdira L, Pilecki Z, Pilecki G. Ultrasound guided application of HA in the soft tissues of the locomotor system in athletes–First experience. Krátká sdělení Botek, M, Stejskal, P, Klimešová, I Vliv normobarické hypoxie na Akt Auton Nerv systému Pilot Stud 191 Drbošalová, V, Stejskal, P, Klugar, M, Krejčíř, V, Hynková, O Charakt klientů komerčn. :205. 39. Ricci M, Micheloni GM, Berti M, Perusi F, Sambugaro E, Vecchini E, et al. Clinical comparison of oral administration and viscosupplementation of hyaluronic acid (HA) in early knee osteoarthritis. Musculoskelet Surg. 2017;101(1):45–9. 40. Wang C-T, Lin Y-T, Chiang B-L, Lin Y-H, Hou S-M. High molecular weight hyaluronic acid down-regulates the gene expression of osteoarthritis-associated cytokines and enzymes in fibroblast-like synoviocytes from patients with early osteoarthritis. Osteoarthr Cartil. 2006;14(12):1237–47. 41. Irrgang JJ, Anderson AF. Development and validation of health-related quality of life measures for the knee. Clin Orthop Relat Res. 2002;402:95–109. 42. Roos EM, Roos HP, Lohmander LS, Ekdahl C, Beynnon BD. Knee Injury and Osteoarthritis Outcome Score (KOOS)—development of a self-administered outcome measure. J Orthop Sport Phys Ther. 1998;28(2):88–96. 43. Perruccio A V, Canizares M, Hawker GA, Roos EM, Maillefert JF, Lohmander SL. Comparative Validity and Responsiveness of the HOOS-PS and KOOS-PS to the WOMAC Physical Function Subscale in Total Joint Replacement for Osteoarthritis. 44. Salavati M, Mazaheri M, Negahban H, Sohani SM, Ebrahimian MR, Ebrahimi I, et al. Validation of a Persian-version of Knee injury and Osteoarthritis Outcome Score (KOOS) in Iranians with knee injuries. Osteoarthr Cartil. 2008;16(10):1178–82. 45. Higgins LD, Taylor MK, Park D, Ghodadra N, Marchant M, Pietrobon R, et al. Reliability and validity of the International Knee Documentation Committee (IKDC) subjective knee form. Jt Bone Spine. 2007;74(6):594–9. 46. Kujala UM, Kaprio J, Sarno S. Osteoarthritis of weight bearing joints of lower limbs in former elite male athletes. Bmj. 1994;308(6923):231–4. 47. Salini V, Pantalone A, Vanni D. Arthroscopy and Sport Injuries. Arthrosc Sport Inj. 2016;433–7. 48. Broglio K. Randomization in clinical trials: Permuted blocks and stratification. JAMA - J Am Med Assoc. 2018;319(21):2223–4. 49. Emanuel EJ, Wendler D, Grady C. What makes clinical research ethical? JAMA. 2000 May;283(20):2701–11. )

[40.	Wang C-T, Lin Y-T, Chiang B-L, Lin Y-H, Hou S-M. High molecular weight hyaluronic acid down-regulates the gene expression of osteoarthritis-associated cytokines and enzymes in fibroblast-like synoviocytes from patients with early osteoarthritis. Osteoarthr Cartil. 2006;14(12):1237–47.](1. Patel DR, Villalobos A. Evaluation and management of knee pain in young athletes: overuse injuries of the knee. Transl Pediatr [Internet]. 2017 Jul;6(3):190–8. Available from: https://pubmed.ncbi.nlm.nih.gov/287950102. Nicolini AP, de Carvalho RT, Matsuda MM, Filho JS, Cohen M. Common injuries in athletes' knee: Experience of a specialized center. Acta Ortop Bras. 2014;22(3):127–31. 3. Tiu T, Craig Van Dien MD. Knee overuse disorders. 4. Fuller CW, Ekstrand J, Junge A, Andersen TE, Bahr R, Dvorak J, et al. Consensus statement on injury definitions and data collection procedures in studies  of football (soccer) injuries. Br J Sports Med. 2006 Mar;40(3):193–201. 5. Majewski M, Susanne H, Klaus S. Epidemiology of athletic knee injuries: A 10-year study. Knee. 2006 Jun;13(3):184–8. 6. Wilder RP, Sethi S. Overuse injuries: tendinopathies, stress fractures, compartment syndrome, and shin  splints. Clin Sports Med. 2004 Jan;23(1):55–81, vi. 7. Aicale R, Tarantino D, Maffulli N. Overuse injuries in sport: a comprehensive overview. J Orthop Surg Res. 2018 Dec;13(1):309. 8. Yang J, Tibbetts AS, Covassin T, Cheng G, Nayar S, Heiden E. Epidemiology of overuse and acute injuries among competitive collegiate athletes. J Athl Train. 2012;47(2):198–204. 9. Bernetti A, Mangone M, Paoloni M, Di Sante L, Murgia M, Santilli V. Corticosteroid and hyaluronic acid injection therapy in tennis elbow (lateral epicondylalgia). Med Sport. 2014;67(2):289–95. 10. Iolascon G, Gimigliano F, Moretti A, De Sire A, Migliore A, Brandi ML, et al. Early osteoarthritis: How to define, diagnose, and manage. A systematic review. Eur Geriatr Med. 2017;8(5–6):383–96. 11. Drawer S, Fuller CW. Propensity for osteoarthritis and lower limb joint pain in retired professional soccer players. Br J Sports Med. 2001;35(6):402–8. 12. Vannini F, Spalding T, Andriolo L, Berruto M, Denti M, Espregueira-Mendes J, et al. Sport and early osteoarthritis: the role of sport in aetiology, progression and treatment of knee osteoarthritis. Knee Surgery, Sport Traumatol Arthrosc. 2016;24(6):1786–96. 13. Fröhlich S, Pazeller S, Cherati AS, Müller E, Frey WO, Spörri J. Overuse injuries in the knee, back and hip of top elite female alpine skiers during  the off-season preparation period: prevalence, severity and their association with traumatic preinjuries and training load. BMJ open Sport Exerc Med. 2020;6(1):e000892. 14. Rincon GA, Vyas D, Zhou J, Fu FH, Oiestad BE, Holm I, et al. \“ Knee function and prevalence of knee osteoarthritis after anterior cruciate ligament reconstruction: a prospective study with 10 to 15 years of follow-up\”. Letter to the editor. Am J Sports Med. 2011;39(4):NP3-author. 15. Kellgren JH, Lawrence J. Radiological assessment of osteo-arthrosis. Ann Rheum Dis. 1957;16(4):494. 16. Altman R, Asch E, Bloch D, Bole G, Borenstein D, Brandt K, et al. Development of criteria for the classification and reporting of osteoarthritis: classification of osteoarthritis of the knee. Arthritis Rheum Off J Am Coll Rheumatol. 1986;29(8):1039–49. 17. Luyten FP, Denti M, Filardo G, Kon E, Engebretsen L. Definition and classification of early osteoarthritis of the knee. Knee Surgery, Sport Traumatol Arthrosc. 2012;20(3):401–6. 18. Hoemann C, Kandel R, Roberts S, Saris DBF, Creemers L, Mainil-Varlet P, et al. International Cartilage Repair Society (ICRS) Recommended Guidelines for  Histological Endpoints for Cartilage Repair Studies in Animal Models and Clinical Trials. Cartilage. 2011 Apr;2(2):153–72. 19. Hunter DJ, Lo GH, Gale D, Grainger AJ, Guermazi A, Conaghan PG. The reliability of a new scoring system for knee osteoarthritis MRI and the validity of bone marrow lesion assessment: BLOKS (Boston–Leeds Osteoarthritis Knee Score). Ann Rheum Dis. 2008;67(2):206–11. 20. Peterfy CG, Guermazi A, Zaim S, Tirman PFJ, Miaux Y, White D, et al. Whole-organ magnetic resonance imaging score (WORMS) of the knee in osteoarthritis. Osteoarthr Cartil. 2004;12(3):177–90. 21. Demange MK, Sisto M, Rodeo S. Future trends for unicompartmental arthritis of the knee: injectables & stem cells. Clin Sports Med. 2014;33(1):161–74. 22. Leslie M. Knee osteoarthritis management therapies. Pain Manag Nurs. 2000;1(2):51–7. 23. Paoloni M, Bernetti A, Belelli A, Brignoli O, Buoso S, Caputi AP, et al. Appropriateness of clinical and organizational criteria for intra-articular injection therapies in osteoarthritis: A Delphi method consensus initiative among experts in Italy. Ann Ist Super Sanita. 2015;51:131–8. 24. Sandmark H, Hogstedt C, Lewold S, Vingård E. Osteoarthrosis of the knee in men and women in association with overweight, smoking, and hormone therapy. Ann Rheum Dis. 1999;58(3):151–5. 25. Christensen BV, Iuhl IU, Vilbek H, Bülow H, Dreijer NC, Rasmussen HF. Acupuncture treatment of severe knee osteoarthrosis. A long‐term study. Acta Anaesthesiol Scand. 1992;36(6):519–25. 26. de Sire A, Agostini F, Lippi L, Mangone M, Marchese S, Cisari C, et al. Oxygen-Ozone Therapy in the Rehabilitation Field: State of the Art on Mechanisms of  Action, Safety and Effectiveness in Patients with Musculoskeletal Disorders. Biomolecules. 2021 Feb;11(3). 27. Fransen M, McCONNELL S. Land-based exercise for osteoarthritis of the knee: a metaanalysis of randomized controlled trials. J Rheumatol. 2009;36(6):1109–17. 28. Hiemstra LA, Kerslake S, Irving C. Anterior knee pain in the athlete. Clin Sports Med. 2014;33(3):437–59. 29. Filardo G, Kon E, Di Matteo B, Pelotti P, Di Martino A, Marcacci M. Platelet-rich plasma for the treatment of patellar tendinopathy: clinical and imaging findings at medium-term follow-up. Int Orthop [Internet]. 2013;37(8):1583–9. Available from: https://doi.org/10.1007/s00264-013-1972-830. Trojian TH, Concoff AL, Joy SM, Hatzenbuehler JR, Saulsberry WJ, Coleman CI. AMSSM Scientific Statement Concerning Viscosupplementation Injections for Knee  Osteoarthritis: Importance for Individual Patient Outcomes. Clin J Sport Med  Off J Can Acad  Sport Med. 2016 Jan;26(1):1–11. 31. Ammar TY, Pereira TAP, Mistura SLL, Kuhn A, Saggin JI, Lopes OV. Viscosupplementation for treating knee osteoarthrosis: review of the literature. Rev Bras Ortop. 2015;50:489–94. 32. Salini V, Pantalone A, Vanni D, Abate M. Viscosupplementation in Athletes. In: Arthroscopy and Sport Injuries. Springer; 2016. p. 433–7. 33. Erdem Y, Gul D, Akpancar S. Comparison of Intraarticular Injections of Hyaluronic Acid versus Dextrose Applied with Periarticular Prolotherapy in the Treatment of Recreational Athletes with Knee Osteoarthritis. Turkish J Sport Med. 2020;55(1):6–13. 34. Kalkhoven J, Coutts AJ, Impellizzeri FM. 'Training load error' is not a more accurate term than 'overuse' injury. Br J Sports Med [Internet]. 2020 Aug 1;54(15):934 LP – 935. Available from: http://bjsm.bmj.com/content/54/15/934.abstract35. Odole AC, Agbomeji OT, Onyeso OKK, Ojo JO, Odunaiya NA. Perspectives of Nigerian Athletes About Physiotherapy Services in Sports Injury Management: Implications for Rehabilitation. J Sport Rehabil. 2021;1(aop):1–8. 36. Hendrika W, Reswari A. The effect of physiotherapy on pain improvement in patients with early knee osteoarthritis at RSU UKI. Int J Med Heal Res. 2021;7(6):52–9. 37. MacKay C, Hawker GA, Jaglal SB. How Do Physical Therapists Approach Management of People With Early Knee Osteoarthritis? A Qualitative Study. Phys Ther [Internet]. 2019;100(2):295–306. Available from: https://doi.org/10.1093/ptj/pzz16438. Hrazdira L, Pilecki Z, Pilecki G. Ultrasound guided application of HA in the soft tissues of the locomotor system in athletes–First experience. Krátká sdělení Botek, M, Stejskal, P, Klimešová, I Vliv normobarické hypoxie na Akt Auton Nerv systému Pilot Stud 191 Drbošalová, V, Stejskal, P, Klugar, M, Krejčíř, V, Hynková, O Charakt klientů komerčn. :205. 39. Ricci M, Micheloni GM, Berti M, Perusi F, Sambugaro E, Vecchini E, et al. Clinical comparison of oral administration and viscosupplementation of hyaluronic acid (HA) in early knee osteoarthritis. Musculoskelet Surg. 2017;101(1):45–9. 40. Wang C-T, Lin Y-T, Chiang B-L, Lin Y-H, Hou S-M. High molecular weight hyaluronic acid down-regulates the gene expression of osteoarthritis-associated cytokines and enzymes in fibroblast-like synoviocytes from patients with early osteoarthritis. Osteoarthr Cartil. 2006;14(12):1237–47. 41. Irrgang JJ, Anderson AF. Development and validation of health-related quality of life measures for the knee. Clin Orthop Relat Res. 2002;402:95–109. 42. Roos EM, Roos HP, Lohmander LS, Ekdahl C, Beynnon BD. Knee Injury and Osteoarthritis Outcome Score (KOOS)—development of a self-administered outcome measure. J Orthop Sport Phys Ther. 1998;28(2):88–96. 43. Perruccio A V, Canizares M, Hawker GA, Roos EM, Maillefert JF, Lohmander SL. Comparative Validity and Responsiveness of the HOOS-PS and KOOS-PS to the WOMAC Physical Function Subscale in Total Joint Replacement for Osteoarthritis. 44. Salavati M, Mazaheri M, Negahban H, Sohani SM, Ebrahimian MR, Ebrahimi I, et al. Validation of a Persian-version of Knee injury and Osteoarthritis Outcome Score (KOOS) in Iranians with knee injuries. Osteoarthr Cartil. 2008;16(10):1178–82. 45. Higgins LD, Taylor MK, Park D, Ghodadra N, Marchant M, Pietrobon R, et al. Reliability and validity of the International Knee Documentation Committee (IKDC) subjective knee form. Jt Bone Spine. 2007;74(6):594–9. 46. Kujala UM, Kaprio J, Sarno S. Osteoarthritis of weight bearing joints of lower limbs in former elite male athletes. Bmj. 1994;308(6923):231–4. 47. Salini V, Pantalone A, Vanni D. Arthroscopy and Sport Injuries. Arthrosc Sport Inj. 2016;433–7. 48. Broglio K. Randomization in clinical trials: Permuted blocks and stratification. JAMA - J Am Med Assoc. 2018;319(21):2223–4. 49. Emanuel EJ, Wendler D, Grady C. What makes clinical research ethical? JAMA. 2000 May;283(20):2701–11. )

[41.	Irrgang JJ, Anderson AF. Development and validation of health-related quality of life measures for the knee. Clin Orthop Relat Res. 2002;402:95–109.](1. Patel DR, Villalobos A. Evaluation and management of knee pain in young athletes: overuse injuries of the knee. Transl Pediatr [Internet]. 2017 Jul;6(3):190–8. Available from: https://pubmed.ncbi.nlm.nih.gov/287950102. Nicolini AP, de Carvalho RT, Matsuda MM, Filho JS, Cohen M. Common injuries in athletes' knee: Experience of a specialized center. Acta Ortop Bras. 2014;22(3):127–31. 3. Tiu T, Craig Van Dien MD. Knee overuse disorders. 4. Fuller CW, Ekstrand J, Junge A, Andersen TE, Bahr R, Dvorak J, et al. Consensus statement on injury definitions and data collection procedures in studies  of football (soccer) injuries. Br J Sports Med. 2006 Mar;40(3):193–201. 5. Majewski M, Susanne H, Klaus S. Epidemiology of athletic knee injuries: A 10-year study. Knee. 2006 Jun;13(3):184–8. 6. Wilder RP, Sethi S. Overuse injuries: tendinopathies, stress fractures, compartment syndrome, and shin  splints. Clin Sports Med. 2004 Jan;23(1):55–81, vi. 7. Aicale R, Tarantino D, Maffulli N. Overuse injuries in sport: a comprehensive overview. J Orthop Surg Res. 2018 Dec;13(1):309. 8. Yang J, Tibbetts AS, Covassin T, Cheng G, Nayar S, Heiden E. Epidemiology of overuse and acute injuries among competitive collegiate athletes. J Athl Train. 2012;47(2):198–204. 9. Bernetti A, Mangone M, Paoloni M, Di Sante L, Murgia M, Santilli V. Corticosteroid and hyaluronic acid injection therapy in tennis elbow (lateral epicondylalgia). Med Sport. 2014;67(2):289–95. 10. Iolascon G, Gimigliano F, Moretti A, De Sire A, Migliore A, Brandi ML, et al. Early osteoarthritis: How to define, diagnose, and manage. A systematic review. Eur Geriatr Med. 2017;8(5–6):383–96. 11. Drawer S, Fuller CW. Propensity for osteoarthritis and lower limb joint pain in retired professional soccer players. Br J Sports Med. 2001;35(6):402–8. 12. Vannini F, Spalding T, Andriolo L, Berruto M, Denti M, Espregueira-Mendes J, et al. Sport and early osteoarthritis: the role of sport in aetiology, progression and treatment of knee osteoarthritis. Knee Surgery, Sport Traumatol Arthrosc. 2016;24(6):1786–96. 13. Fröhlich S, Pazeller S, Cherati AS, Müller E, Frey WO, Spörri J. Overuse injuries in the knee, back and hip of top elite female alpine skiers during  the off-season preparation period: prevalence, severity and their association with traumatic preinjuries and training load. BMJ open Sport Exerc Med. 2020;6(1):e000892. 14. Rincon GA, Vyas D, Zhou J, Fu FH, Oiestad BE, Holm I, et al. \“ Knee function and prevalence of knee osteoarthritis after anterior cruciate ligament reconstruction: a prospective study with 10 to 15 years of follow-up\”. Letter to the editor. Am J Sports Med. 2011;39(4):NP3-author. 15. Kellgren JH, Lawrence J. Radiological assessment of osteo-arthrosis. Ann Rheum Dis. 1957;16(4):494. 16. Altman R, Asch E, Bloch D, Bole G, Borenstein D, Brandt K, et al. Development of criteria for the classification and reporting of osteoarthritis: classification of osteoarthritis of the knee. Arthritis Rheum Off J Am Coll Rheumatol. 1986;29(8):1039–49. 17. Luyten FP, Denti M, Filardo G, Kon E, Engebretsen L. Definition and classification of early osteoarthritis of the knee. Knee Surgery, Sport Traumatol Arthrosc. 2012;20(3):401–6. 18. Hoemann C, Kandel R, Roberts S, Saris DBF, Creemers L, Mainil-Varlet P, et al. International Cartilage Repair Society (ICRS) Recommended Guidelines for  Histological Endpoints for Cartilage Repair Studies in Animal Models and Clinical Trials. Cartilage. 2011 Apr;2(2):153–72. 19. Hunter DJ, Lo GH, Gale D, Grainger AJ, Guermazi A, Conaghan PG. The reliability of a new scoring system for knee osteoarthritis MRI and the validity of bone marrow lesion assessment: BLOKS (Boston–Leeds Osteoarthritis Knee Score). Ann Rheum Dis. 2008;67(2):206–11. 20. Peterfy CG, Guermazi A, Zaim S, Tirman PFJ, Miaux Y, White D, et al. Whole-organ magnetic resonance imaging score (WORMS) of the knee in osteoarthritis. Osteoarthr Cartil. 2004;12(3):177–90. 21. Demange MK, Sisto M, Rodeo S. Future trends for unicompartmental arthritis of the knee: injectables & stem cells. Clin Sports Med. 2014;33(1):161–74. 22. Leslie M. Knee osteoarthritis management therapies. Pain Manag Nurs. 2000;1(2):51–7. 23. Paoloni M, Bernetti A, Belelli A, Brignoli O, Buoso S, Caputi AP, et al. Appropriateness of clinical and organizational criteria for intra-articular injection therapies in osteoarthritis: A Delphi method consensus initiative among experts in Italy. Ann Ist Super Sanita. 2015;51:131–8. 24. Sandmark H, Hogstedt C, Lewold S, Vingård E. Osteoarthrosis of the knee in men and women in association with overweight, smoking, and hormone therapy. Ann Rheum Dis. 1999;58(3):151–5. 25. Christensen BV, Iuhl IU, Vilbek H, Bülow H, Dreijer NC, Rasmussen HF. Acupuncture treatment of severe knee osteoarthrosis. A long‐term study. Acta Anaesthesiol Scand. 1992;36(6):519–25. 26. de Sire A, Agostini F, Lippi L, Mangone M, Marchese S, Cisari C, et al. Oxygen-Ozone Therapy in the Rehabilitation Field: State of the Art on Mechanisms of  Action, Safety and Effectiveness in Patients with Musculoskeletal Disorders. Biomolecules. 2021 Feb;11(3). 27. Fransen M, McCONNELL S. Land-based exercise for osteoarthritis of the knee: a metaanalysis of randomized controlled trials. J Rheumatol. 2009;36(6):1109–17. 28. Hiemstra LA, Kerslake S, Irving C. Anterior knee pain in the athlete. Clin Sports Med. 2014;33(3):437–59. 29. Filardo G, Kon E, Di Matteo B, Pelotti P, Di Martino A, Marcacci M. Platelet-rich plasma for the treatment of patellar tendinopathy: clinical and imaging findings at medium-term follow-up. Int Orthop [Internet]. 2013;37(8):1583–9. Available from: https://doi.org/10.1007/s00264-013-1972-830. Trojian TH, Concoff AL, Joy SM, Hatzenbuehler JR, Saulsberry WJ, Coleman CI. AMSSM Scientific Statement Concerning Viscosupplementation Injections for Knee  Osteoarthritis: Importance for Individual Patient Outcomes. Clin J Sport Med  Off J Can Acad  Sport Med. 2016 Jan;26(1):1–11. 31. Ammar TY, Pereira TAP, Mistura SLL, Kuhn A, Saggin JI, Lopes OV. Viscosupplementation for treating knee osteoarthrosis: review of the literature. Rev Bras Ortop. 2015;50:489–94. 32. Salini V, Pantalone A, Vanni D, Abate M. Viscosupplementation in Athletes. In: Arthroscopy and Sport Injuries. Springer; 2016. p. 433–7. 33. Erdem Y, Gul D, Akpancar S. Comparison of Intraarticular Injections of Hyaluronic Acid versus Dextrose Applied with Periarticular Prolotherapy in the Treatment of Recreational Athletes with Knee Osteoarthritis. Turkish J Sport Med. 2020;55(1):6–13. 34. Kalkhoven J, Coutts AJ, Impellizzeri FM. 'Training load error' is not a more accurate term than 'overuse' injury. Br J Sports Med [Internet]. 2020 Aug 1;54(15):934 LP – 935. Available from: http://bjsm.bmj.com/content/54/15/934.abstract35. Odole AC, Agbomeji OT, Onyeso OKK, Ojo JO, Odunaiya NA. Perspectives of Nigerian Athletes About Physiotherapy Services in Sports Injury Management: Implications for Rehabilitation. J Sport Rehabil. 2021;1(aop):1–8. 36. Hendrika W, Reswari A. The effect of physiotherapy on pain improvement in patients with early knee osteoarthritis at RSU UKI. Int J Med Heal Res. 2021;7(6):52–9. 37. MacKay C, Hawker GA, Jaglal SB. How Do Physical Therapists Approach Management of People With Early Knee Osteoarthritis? A Qualitative Study. Phys Ther [Internet]. 2019;100(2):295–306. Available from: https://doi.org/10.1093/ptj/pzz16438. Hrazdira L, Pilecki Z, Pilecki G. Ultrasound guided application of HA in the soft tissues of the locomotor system in athletes–First experience. Krátká sdělení Botek, M, Stejskal, P, Klimešová, I Vliv normobarické hypoxie na Akt Auton Nerv systému Pilot Stud 191 Drbošalová, V, Stejskal, P, Klugar, M, Krejčíř, V, Hynková, O Charakt klientů komerčn. :205. 39. Ricci M, Micheloni GM, Berti M, Perusi F, Sambugaro E, Vecchini E, et al. Clinical comparison of oral administration and viscosupplementation of hyaluronic acid (HA) in early knee osteoarthritis. Musculoskelet Surg. 2017;101(1):45–9. 40. Wang C-T, Lin Y-T, Chiang B-L, Lin Y-H, Hou S-M. High molecular weight hyaluronic acid down-regulates the gene expression of osteoarthritis-associated cytokines and enzymes in fibroblast-like synoviocytes from patients with early osteoarthritis. Osteoarthr Cartil. 2006;14(12):1237–47. 41. Irrgang JJ, Anderson AF. Development and validation of health-related quality of life measures for the knee. Clin Orthop Relat Res. 2002;402:95–109. 42. Roos EM, Roos HP, Lohmander LS, Ekdahl C, Beynnon BD. Knee Injury and Osteoarthritis Outcome Score (KOOS)—development of a self-administered outcome measure. J Orthop Sport Phys Ther. 1998;28(2):88–96. 43. Perruccio A V, Canizares M, Hawker GA, Roos EM, Maillefert JF, Lohmander SL. Comparative Validity and Responsiveness of the HOOS-PS and KOOS-PS to the WOMAC Physical Function Subscale in Total Joint Replacement for Osteoarthritis. 44. Salavati M, Mazaheri M, Negahban H, Sohani SM, Ebrahimian MR, Ebrahimi I, et al. Validation of a Persian-version of Knee injury and Osteoarthritis Outcome Score (KOOS) in Iranians with knee injuries. Osteoarthr Cartil. 2008;16(10):1178–82. 45. Higgins LD, Taylor MK, Park D, Ghodadra N, Marchant M, Pietrobon R, et al. Reliability and validity of the International Knee Documentation Committee (IKDC) subjective knee form. Jt Bone Spine. 2007;74(6):594–9. 46. Kujala UM, Kaprio J, Sarno S. Osteoarthritis of weight bearing joints of lower limbs in former elite male athletes. Bmj. 1994;308(6923):231–4. 47. Salini V, Pantalone A, Vanni D. Arthroscopy and Sport Injuries. Arthrosc Sport Inj. 2016;433–7. 48. Broglio K. Randomization in clinical trials: Permuted blocks and stratification. JAMA - J Am Med Assoc. 2018;319(21):2223–4. 49. Emanuel EJ, Wendler D, Grady C. What makes clinical research ethical? JAMA. 2000 May;283(20):2701–11. )

[42.	Roos EM, Roos HP, Lohmander LS, Ekdahl C, Beynnon BD. Knee Injury and Osteoarthritis Outcome Score (KOOS)—development of a self-administered outcome measure. J Orthop Sport Phys Ther. 1998;28(2):88–96.](1. Patel DR, Villalobos A. Evaluation and management of knee pain in young athletes: overuse injuries of the knee. Transl Pediatr [Internet]. 2017 Jul;6(3):190–8. Available from: https://pubmed.ncbi.nlm.nih.gov/287950102. Nicolini AP, de Carvalho RT, Matsuda MM, Filho JS, Cohen M. Common injuries in athletes' knee: Experience of a specialized center. Acta Ortop Bras. 2014;22(3):127–31. 3. Tiu T, Craig Van Dien MD. Knee overuse disorders. 4. Fuller CW, Ekstrand J, Junge A, Andersen TE, Bahr R, Dvorak J, et al. Consensus statement on injury definitions and data collection procedures in studies  of football (soccer) injuries. Br J Sports Med. 2006 Mar;40(3):193–201. 5. Majewski M, Susanne H, Klaus S. Epidemiology of athletic knee injuries: A 10-year study. Knee. 2006 Jun;13(3):184–8. 6. Wilder RP, Sethi S. Overuse injuries: tendinopathies, stress fractures, compartment syndrome, and shin  splints. Clin Sports Med. 2004 Jan;23(1):55–81, vi. 7. Aicale R, Tarantino D, Maffulli N. Overuse injuries in sport: a comprehensive overview. J Orthop Surg Res. 2018 Dec;13(1):309. 8. Yang J, Tibbetts AS, Covassin T, Cheng G, Nayar S, Heiden E. Epidemiology of overuse and acute injuries among competitive collegiate athletes. J Athl Train. 2012;47(2):198–204. 9. Bernetti A, Mangone M, Paoloni M, Di Sante L, Murgia M, Santilli V. Corticosteroid and hyaluronic acid injection therapy in tennis elbow (lateral epicondylalgia). Med Sport. 2014;67(2):289–95. 10. Iolascon G, Gimigliano F, Moretti A, De Sire A, Migliore A, Brandi ML, et al. Early osteoarthritis: How to define, diagnose, and manage. A systematic review. Eur Geriatr Med. 2017;8(5–6):383–96. 11. Drawer S, Fuller CW. Propensity for osteoarthritis and lower limb joint pain in retired professional soccer players. Br J Sports Med. 2001;35(6):402–8. 12. Vannini F, Spalding T, Andriolo L, Berruto M, Denti M, Espregueira-Mendes J, et al. Sport and early osteoarthritis: the role of sport in aetiology, progression and treatment of knee osteoarthritis. Knee Surgery, Sport Traumatol Arthrosc. 2016;24(6):1786–96. 13. Fröhlich S, Pazeller S, Cherati AS, Müller E, Frey WO, Spörri J. Overuse injuries in the knee, back and hip of top elite female alpine skiers during  the off-season preparation period: prevalence, severity and their association with traumatic preinjuries and training load. BMJ open Sport Exerc Med. 2020;6(1):e000892. 14. Rincon GA, Vyas D, Zhou J, Fu FH, Oiestad BE, Holm I, et al. \“ Knee function and prevalence of knee osteoarthritis after anterior cruciate ligament reconstruction: a prospective study with 10 to 15 years of follow-up\”. Letter to the editor. Am J Sports Med. 2011;39(4):NP3-author. 15. Kellgren JH, Lawrence J. Radiological assessment of osteo-arthrosis. Ann Rheum Dis. 1957;16(4):494. 16. Altman R, Asch E, Bloch D, Bole G, Borenstein D, Brandt K, et al. Development of criteria for the classification and reporting of osteoarthritis: classification of osteoarthritis of the knee. Arthritis Rheum Off J Am Coll Rheumatol. 1986;29(8):1039–49. 17. Luyten FP, Denti M, Filardo G, Kon E, Engebretsen L. Definition and classification of early osteoarthritis of the knee. Knee Surgery, Sport Traumatol Arthrosc. 2012;20(3):401–6. 18. Hoemann C, Kandel R, Roberts S, Saris DBF, Creemers L, Mainil-Varlet P, et al. International Cartilage Repair Society (ICRS) Recommended Guidelines for  Histological Endpoints for Cartilage Repair Studies in Animal Models and Clinical Trials. Cartilage. 2011 Apr;2(2):153–72. 19. Hunter DJ, Lo GH, Gale D, Grainger AJ, Guermazi A, Conaghan PG. The reliability of a new scoring system for knee osteoarthritis MRI and the validity of bone marrow lesion assessment: BLOKS (Boston–Leeds Osteoarthritis Knee Score). Ann Rheum Dis. 2008;67(2):206–11. 20. Peterfy CG, Guermazi A, Zaim S, Tirman PFJ, Miaux Y, White D, et al. Whole-organ magnetic resonance imaging score (WORMS) of the knee in osteoarthritis. Osteoarthr Cartil. 2004;12(3):177–90. 21. Demange MK, Sisto M, Rodeo S. Future trends for unicompartmental arthritis of the knee: injectables & stem cells. Clin Sports Med. 2014;33(1):161–74. 22. Leslie M. Knee osteoarthritis management therapies. Pain Manag Nurs. 2000;1(2):51–7. 23. Paoloni M, Bernetti A, Belelli A, Brignoli O, Buoso S, Caputi AP, et al. Appropriateness of clinical and organizational criteria for intra-articular injection therapies in osteoarthritis: A Delphi method consensus initiative among experts in Italy. Ann Ist Super Sanita. 2015;51:131–8. 24. Sandmark H, Hogstedt C, Lewold S, Vingård E. Osteoarthrosis of the knee in men and women in association with overweight, smoking, and hormone therapy. Ann Rheum Dis. 1999;58(3):151–5. 25. Christensen BV, Iuhl IU, Vilbek H, Bülow H, Dreijer NC, Rasmussen HF. Acupuncture treatment of severe knee osteoarthrosis. A long‐term study. Acta Anaesthesiol Scand. 1992;36(6):519–25. 26. de Sire A, Agostini F, Lippi L, Mangone M, Marchese S, Cisari C, et al. Oxygen-Ozone Therapy in the Rehabilitation Field: State of the Art on Mechanisms of  Action, Safety and Effectiveness in Patients with Musculoskeletal Disorders. Biomolecules. 2021 Feb;11(3). 27. Fransen M, McCONNELL S. Land-based exercise for osteoarthritis of the knee: a metaanalysis of randomized controlled trials. J Rheumatol. 2009;36(6):1109–17. 28. Hiemstra LA, Kerslake S, Irving C. Anterior knee pain in the athlete. Clin Sports Med. 2014;33(3):437–59. 29. Filardo G, Kon E, Di Matteo B, Pelotti P, Di Martino A, Marcacci M. Platelet-rich plasma for the treatment of patellar tendinopathy: clinical and imaging findings at medium-term follow-up. Int Orthop [Internet]. 2013;37(8):1583–9. Available from: https://doi.org/10.1007/s00264-013-1972-830. Trojian TH, Concoff AL, Joy SM, Hatzenbuehler JR, Saulsberry WJ, Coleman CI. AMSSM Scientific Statement Concerning Viscosupplementation Injections for Knee  Osteoarthritis: Importance for Individual Patient Outcomes. Clin J Sport Med  Off J Can Acad  Sport Med. 2016 Jan;26(1):1–11. 31. Ammar TY, Pereira TAP, Mistura SLL, Kuhn A, Saggin JI, Lopes OV. Viscosupplementation for treating knee osteoarthrosis: review of the literature. Rev Bras Ortop. 2015;50:489–94. 32. Salini V, Pantalone A, Vanni D, Abate M. Viscosupplementation in Athletes. In: Arthroscopy and Sport Injuries. Springer; 2016. p. 433–7. 33. Erdem Y, Gul D, Akpancar S. Comparison of Intraarticular Injections of Hyaluronic Acid versus Dextrose Applied with Periarticular Prolotherapy in the Treatment of Recreational Athletes with Knee Osteoarthritis. Turkish J Sport Med. 2020;55(1):6–13. 34. Kalkhoven J, Coutts AJ, Impellizzeri FM. 'Training load error' is not a more accurate term than 'overuse' injury. Br J Sports Med [Internet]. 2020 Aug 1;54(15):934 LP – 935. Available from: http://bjsm.bmj.com/content/54/15/934.abstract35. Odole AC, Agbomeji OT, Onyeso OKK, Ojo JO, Odunaiya NA. Perspectives of Nigerian Athletes About Physiotherapy Services in Sports Injury Management: Implications for Rehabilitation. J Sport Rehabil. 2021;1(aop):1–8. 36. Hendrika W, Reswari A. The effect of physiotherapy on pain improvement in patients with early knee osteoarthritis at RSU UKI. Int J Med Heal Res. 2021;7(6):52–9. 37. MacKay C, Hawker GA, Jaglal SB. How Do Physical Therapists Approach Management of People With Early Knee Osteoarthritis? A Qualitative Study. Phys Ther [Internet]. 2019;100(2):295–306. Available from: https://doi.org/10.1093/ptj/pzz16438. Hrazdira L, Pilecki Z, Pilecki G. Ultrasound guided application of HA in the soft tissues of the locomotor system in athletes–First experience. Krátká sdělení Botek, M, Stejskal, P, Klimešová, I Vliv normobarické hypoxie na Akt Auton Nerv systému Pilot Stud 191 Drbošalová, V, Stejskal, P, Klugar, M, Krejčíř, V, Hynková, O Charakt klientů komerčn. :205. 39. Ricci M, Micheloni GM, Berti M, Perusi F, Sambugaro E, Vecchini E, et al. Clinical comparison of oral administration and viscosupplementation of hyaluronic acid (HA) in early knee osteoarthritis. Musculoskelet Surg. 2017;101(1):45–9. 40. Wang C-T, Lin Y-T, Chiang B-L, Lin Y-H, Hou S-M. High molecular weight hyaluronic acid down-regulates the gene expression of osteoarthritis-associated cytokines and enzymes in fibroblast-like synoviocytes from patients with early osteoarthritis. Osteoarthr Cartil. 2006;14(12):1237–47. 41. Irrgang JJ, Anderson AF. Development and validation of health-related quality of life measures for the knee. Clin Orthop Relat Res. 2002;402:95–109. 42. Roos EM, Roos HP, Lohmander LS, Ekdahl C, Beynnon BD. Knee Injury and Osteoarthritis Outcome Score (KOOS)—development of a self-administered outcome measure. J Orthop Sport Phys Ther. 1998;28(2):88–96. 43. Perruccio A V, Canizares M, Hawker GA, Roos EM, Maillefert JF, Lohmander SL. Comparative Validity and Responsiveness of the HOOS-PS and KOOS-PS to the WOMAC Physical Function Subscale in Total Joint Replacement for Osteoarthritis. 44. Salavati M, Mazaheri M, Negahban H, Sohani SM, Ebrahimian MR, Ebrahimi I, et al. Validation of a Persian-version of Knee injury and Osteoarthritis Outcome Score (KOOS) in Iranians with knee injuries. Osteoarthr Cartil. 2008;16(10):1178–82. 45. Higgins LD, Taylor MK, Park D, Ghodadra N, Marchant M, Pietrobon R, et al. Reliability and validity of the International Knee Documentation Committee (IKDC) subjective knee form. Jt Bone Spine. 2007;74(6):594–9. 46. Kujala UM, Kaprio J, Sarno S. Osteoarthritis of weight bearing joints of lower limbs in former elite male athletes. Bmj. 1994;308(6923):231–4. 47. Salini V, Pantalone A, Vanni D. Arthroscopy and Sport Injuries. Arthrosc Sport Inj. 2016;433–7. 48. Broglio K. Randomization in clinical trials: Permuted blocks and stratification. JAMA - J Am Med Assoc. 2018;319(21):2223–4. 49. Emanuel EJ, Wendler D, Grady C. What makes clinical research ethical? JAMA. 2000 May;283(20):2701–11. )

[43.	Perruccio A V, Canizares M, Hawker GA, Roos EM, Maillefert JF, Lohmander SL. Comparative Validity and Responsiveness of the HOOS-PS and KOOS-PS to the WOMAC Physical Function Subscale in Total Joint Replacement for Osteoarthritis.](1. Patel DR, Villalobos A. Evaluation and management of knee pain in young athletes: overuse injuries of the knee. Transl Pediatr [Internet]. 2017 Jul;6(3):190–8. Available from: https://pubmed.ncbi.nlm.nih.gov/287950102. Nicolini AP, de Carvalho RT, Matsuda MM, Filho JS, Cohen M. Common injuries in athletes' knee: Experience of a specialized center. Acta Ortop Bras. 2014;22(3):127–31. 3. Tiu T, Craig Van Dien MD. Knee overuse disorders. 4. Fuller CW, Ekstrand J, Junge A, Andersen TE, Bahr R, Dvorak J, et al. Consensus statement on injury definitions and data collection procedures in studies  of football (soccer) injuries. Br J Sports Med. 2006 Mar;40(3):193–201. 5. Majewski M, Susanne H, Klaus S. Epidemiology of athletic knee injuries: A 10-year study. Knee. 2006 Jun;13(3):184–8. 6. Wilder RP, Sethi S. Overuse injuries: tendinopathies, stress fractures, compartment syndrome, and shin  splints. Clin Sports Med. 2004 Jan;23(1):55–81, vi. 7. Aicale R, Tarantino D, Maffulli N. Overuse injuries in sport: a comprehensive overview. J Orthop Surg Res. 2018 Dec;13(1):309. 8. Yang J, Tibbetts AS, Covassin T, Cheng G, Nayar S, Heiden E. Epidemiology of overuse and acute injuries among competitive collegiate athletes. J Athl Train. 2012;47(2):198–204. 9. Bernetti A, Mangone M, Paoloni M, Di Sante L, Murgia M, Santilli V. Corticosteroid and hyaluronic acid injection therapy in tennis elbow (lateral epicondylalgia). Med Sport. 2014;67(2):289–95. 10. Iolascon G, Gimigliano F, Moretti A, De Sire A, Migliore A, Brandi ML, et al. Early osteoarthritis: How to define, diagnose, and manage. A systematic review. Eur Geriatr Med. 2017;8(5–6):383–96. 11. Drawer S, Fuller CW. Propensity for osteoarthritis and lower limb joint pain in retired professional soccer players. Br J Sports Med. 2001;35(6):402–8. 12. Vannini F, Spalding T, Andriolo L, Berruto M, Denti M, Espregueira-Mendes J, et al. Sport and early osteoarthritis: the role of sport in aetiology, progression and treatment of knee osteoarthritis. Knee Surgery, Sport Traumatol Arthrosc. 2016;24(6):1786–96. 13. Fröhlich S, Pazeller S, Cherati AS, Müller E, Frey WO, Spörri J. Overuse injuries in the knee, back and hip of top elite female alpine skiers during  the off-season preparation period: prevalence, severity and their association with traumatic preinjuries and training load. BMJ open Sport Exerc Med. 2020;6(1):e000892. 14. Rincon GA, Vyas D, Zhou J, Fu FH, Oiestad BE, Holm I, et al. \“ Knee function and prevalence of knee osteoarthritis after anterior cruciate ligament reconstruction: a prospective study with 10 to 15 years of follow-up\”. Letter to the editor. Am J Sports Med. 2011;39(4):NP3-author. 15. Kellgren JH, Lawrence J. Radiological assessment of osteo-arthrosis. Ann Rheum Dis. 1957;16(4):494. 16. Altman R, Asch E, Bloch D, Bole G, Borenstein D, Brandt K, et al. Development of criteria for the classification and reporting of osteoarthritis: classification of osteoarthritis of the knee. Arthritis Rheum Off J Am Coll Rheumatol. 1986;29(8):1039–49. 17. Luyten FP, Denti M, Filardo G, Kon E, Engebretsen L. Definition and classification of early osteoarthritis of the knee. Knee Surgery, Sport Traumatol Arthrosc. 2012;20(3):401–6. 18. Hoemann C, Kandel R, Roberts S, Saris DBF, Creemers L, Mainil-Varlet P, et al. International Cartilage Repair Society (ICRS) Recommended Guidelines for  Histological Endpoints for Cartilage Repair Studies in Animal Models and Clinical Trials. Cartilage. 2011 Apr;2(2):153–72. 19. Hunter DJ, Lo GH, Gale D, Grainger AJ, Guermazi A, Conaghan PG. The reliability of a new scoring system for knee osteoarthritis MRI and the validity of bone marrow lesion assessment: BLOKS (Boston–Leeds Osteoarthritis Knee Score). Ann Rheum Dis. 2008;67(2):206–11. 20. Peterfy CG, Guermazi A, Zaim S, Tirman PFJ, Miaux Y, White D, et al. Whole-organ magnetic resonance imaging score (WORMS) of the knee in osteoarthritis. Osteoarthr Cartil. 2004;12(3):177–90. 21. Demange MK, Sisto M, Rodeo S. Future trends for unicompartmental arthritis of the knee: injectables & stem cells. Clin Sports Med. 2014;33(1):161–74. 22. Leslie M. Knee osteoarthritis management therapies. Pain Manag Nurs. 2000;1(2):51–7. 23. Paoloni M, Bernetti A, Belelli A, Brignoli O, Buoso S, Caputi AP, et al. Appropriateness of clinical and organizational criteria for intra-articular injection therapies in osteoarthritis: A Delphi method consensus initiative among experts in Italy. Ann Ist Super Sanita. 2015;51:131–8. 24. Sandmark H, Hogstedt C, Lewold S, Vingård E. Osteoarthrosis of the knee in men and women in association with overweight, smoking, and hormone therapy. Ann Rheum Dis. 1999;58(3):151–5. 25. Christensen BV, Iuhl IU, Vilbek H, Bülow H, Dreijer NC, Rasmussen HF. Acupuncture treatment of severe knee osteoarthrosis. A long‐term study. Acta Anaesthesiol Scand. 1992;36(6):519–25. 26. de Sire A, Agostini F, Lippi L, Mangone M, Marchese S, Cisari C, et al. Oxygen-Ozone Therapy in the Rehabilitation Field: State of the Art on Mechanisms of  Action, Safety and Effectiveness in Patients with Musculoskeletal Disorders. Biomolecules. 2021 Feb;11(3). 27. Fransen M, McCONNELL S. Land-based exercise for osteoarthritis of the knee: a metaanalysis of randomized controlled trials. J Rheumatol. 2009;36(6):1109–17. 28. Hiemstra LA, Kerslake S, Irving C. Anterior knee pain in the athlete. Clin Sports Med. 2014;33(3):437–59. 29. Filardo G, Kon E, Di Matteo B, Pelotti P, Di Martino A, Marcacci M. Platelet-rich plasma for the treatment of patellar tendinopathy: clinical and imaging findings at medium-term follow-up. Int Orthop [Internet]. 2013;37(8):1583–9. Available from: https://doi.org/10.1007/s00264-013-1972-830. Trojian TH, Concoff AL, Joy SM, Hatzenbuehler JR, Saulsberry WJ, Coleman CI. AMSSM Scientific Statement Concerning Viscosupplementation Injections for Knee  Osteoarthritis: Importance for Individual Patient Outcomes. Clin J Sport Med  Off J Can Acad  Sport Med. 2016 Jan;26(1):1–11. 31. Ammar TY, Pereira TAP, Mistura SLL, Kuhn A, Saggin JI, Lopes OV. Viscosupplementation for treating knee osteoarthrosis: review of the literature. Rev Bras Ortop. 2015;50:489–94. 32. Salini V, Pantalone A, Vanni D, Abate M. Viscosupplementation in Athletes. In: Arthroscopy and Sport Injuries. Springer; 2016. p. 433–7. 33. Erdem Y, Gul D, Akpancar S. Comparison of Intraarticular Injections of Hyaluronic Acid versus Dextrose Applied with Periarticular Prolotherapy in the Treatment of Recreational Athletes with Knee Osteoarthritis. Turkish J Sport Med. 2020;55(1):6–13. 34. Kalkhoven J, Coutts AJ, Impellizzeri FM. 'Training load error' is not a more accurate term than 'overuse' injury. Br J Sports Med [Internet]. 2020 Aug 1;54(15):934 LP – 935. Available from: http://bjsm.bmj.com/content/54/15/934.abstract35. Odole AC, Agbomeji OT, Onyeso OKK, Ojo JO, Odunaiya NA. Perspectives of Nigerian Athletes About Physiotherapy Services in Sports Injury Management: Implications for Rehabilitation. J Sport Rehabil. 2021;1(aop):1–8. 36. Hendrika W, Reswari A. The effect of physiotherapy on pain improvement in patients with early knee osteoarthritis at RSU UKI. Int J Med Heal Res. 2021;7(6):52–9. 37. MacKay C, Hawker GA, Jaglal SB. How Do Physical Therapists Approach Management of People With Early Knee Osteoarthritis? A Qualitative Study. Phys Ther [Internet]. 2019;100(2):295–306. Available from: https://doi.org/10.1093/ptj/pzz16438. Hrazdira L, Pilecki Z, Pilecki G. Ultrasound guided application of HA in the soft tissues of the locomotor system in athletes–First experience. Krátká sdělení Botek, M, Stejskal, P, Klimešová, I Vliv normobarické hypoxie na Akt Auton Nerv systému Pilot Stud 191 Drbošalová, V, Stejskal, P, Klugar, M, Krejčíř, V, Hynková, O Charakt klientů komerčn. :205. 39. Ricci M, Micheloni GM, Berti M, Perusi F, Sambugaro E, Vecchini E, et al. Clinical comparison of oral administration and viscosupplementation of hyaluronic acid (HA) in early knee osteoarthritis. Musculoskelet Surg. 2017;101(1):45–9. 40. Wang C-T, Lin Y-T, Chiang B-L, Lin Y-H, Hou S-M. High molecular weight hyaluronic acid down-regulates the gene expression of osteoarthritis-associated cytokines and enzymes in fibroblast-like synoviocytes from patients with early osteoarthritis. Osteoarthr Cartil. 2006;14(12):1237–47. 41. Irrgang JJ, Anderson AF. Development and validation of health-related quality of life measures for the knee. Clin Orthop Relat Res. 2002;402:95–109. 42. Roos EM, Roos HP, Lohmander LS, Ekdahl C, Beynnon BD. Knee Injury and Osteoarthritis Outcome Score (KOOS)—development of a self-administered outcome measure. J Orthop Sport Phys Ther. 1998;28(2):88–96. 43. Perruccio A V, Canizares M, Hawker GA, Roos EM, Maillefert JF, Lohmander SL. Comparative Validity and Responsiveness of the HOOS-PS and KOOS-PS to the WOMAC Physical Function Subscale in Total Joint Replacement for Osteoarthritis. 44. Salavati M, Mazaheri M, Negahban H, Sohani SM, Ebrahimian MR, Ebrahimi I, et al. Validation of a Persian-version of Knee injury and Osteoarthritis Outcome Score (KOOS) in Iranians with knee injuries. Osteoarthr Cartil. 2008;16(10):1178–82. 45. Higgins LD, Taylor MK, Park D, Ghodadra N, Marchant M, Pietrobon R, et al. Reliability and validity of the International Knee Documentation Committee (IKDC) subjective knee form. Jt Bone Spine. 2007;74(6):594–9. 46. Kujala UM, Kaprio J, Sarno S. Osteoarthritis of weight bearing joints of lower limbs in former elite male athletes. Bmj. 1994;308(6923):231–4. 47. Salini V, Pantalone A, Vanni D. Arthroscopy and Sport Injuries. Arthrosc Sport Inj. 2016;433–7. 48. Broglio K. Randomization in clinical trials: Permuted blocks and stratification. JAMA - J Am Med Assoc. 2018;319(21):2223–4. 49. Emanuel EJ, Wendler D, Grady C. What makes clinical research ethical? JAMA. 2000 May;283(20):2701–11. )

[44.	Salavati M, Mazaheri M, Negahban H, Sohani SM, Ebrahimian MR, Ebrahimi I, et al. Validation of a Persian-version of Knee injury and Osteoarthritis Outcome Score (KOOS) in Iranians with knee injuries. Osteoarthr Cartil. 2008;16(10):1178–82.](1. Patel DR, Villalobos A. Evaluation and management of knee pain in young athletes: overuse injuries of the knee. Transl Pediatr [Internet]. 2017 Jul;6(3):190–8. Available from: https://pubmed.ncbi.nlm.nih.gov/287950102. Nicolini AP, de Carvalho RT, Matsuda MM, Filho JS, Cohen M. Common injuries in athletes' knee: Experience of a specialized center. Acta Ortop Bras. 2014;22(3):127–31. 3. Tiu T, Craig Van Dien MD. Knee overuse disorders. 4. Fuller CW, Ekstrand J, Junge A, Andersen TE, Bahr R, Dvorak J, et al. Consensus statement on injury definitions and data collection procedures in studies  of football (soccer) injuries. Br J Sports Med. 2006 Mar;40(3):193–201. 5. Majewski M, Susanne H, Klaus S. Epidemiology of athletic knee injuries: A 10-year study. Knee. 2006 Jun;13(3):184–8. 6. Wilder RP, Sethi S. Overuse injuries: tendinopathies, stress fractures, compartment syndrome, and shin  splints. Clin Sports Med. 2004 Jan;23(1):55–81, vi. 7. Aicale R, Tarantino D, Maffulli N. Overuse injuries in sport: a comprehensive overview. J Orthop Surg Res. 2018 Dec;13(1):309. 8. Yang J, Tibbetts AS, Covassin T, Cheng G, Nayar S, Heiden E. Epidemiology of overuse and acute injuries among competitive collegiate athletes. J Athl Train. 2012;47(2):198–204. 9. Bernetti A, Mangone M, Paoloni M, Di Sante L, Murgia M, Santilli V. Corticosteroid and hyaluronic acid injection therapy in tennis elbow (lateral epicondylalgia). Med Sport. 2014;67(2):289–95. 10. Iolascon G, Gimigliano F, Moretti A, De Sire A, Migliore A, Brandi ML, et al. Early osteoarthritis: How to define, diagnose, and manage. A systematic review. Eur Geriatr Med. 2017;8(5–6):383–96. 11. Drawer S, Fuller CW. Propensity for osteoarthritis and lower limb joint pain in retired professional soccer players. Br J Sports Med. 2001;35(6):402–8. 12. Vannini F, Spalding T, Andriolo L, Berruto M, Denti M, Espregueira-Mendes J, et al. Sport and early osteoarthritis: the role of sport in aetiology, progression and treatment of knee osteoarthritis. Knee Surgery, Sport Traumatol Arthrosc. 2016;24(6):1786–96. 13. Fröhlich S, Pazeller S, Cherati AS, Müller E, Frey WO, Spörri J. Overuse injuries in the knee, back and hip of top elite female alpine skiers during  the off-season preparation period: prevalence, severity and their association with traumatic preinjuries and training load. BMJ open Sport Exerc Med. 2020;6(1):e000892. 14. Rincon GA, Vyas D, Zhou J, Fu FH, Oiestad BE, Holm I, et al. \“ Knee function and prevalence of knee osteoarthritis after anterior cruciate ligament reconstruction: a prospective study with 10 to 15 years of follow-up\”. Letter to the editor. Am J Sports Med. 2011;39(4):NP3-author. 15. Kellgren JH, Lawrence J. Radiological assessment of osteo-arthrosis. Ann Rheum Dis. 1957;16(4):494. 16. Altman R, Asch E, Bloch D, Bole G, Borenstein D, Brandt K, et al. Development of criteria for the classification and reporting of osteoarthritis: classification of osteoarthritis of the knee. Arthritis Rheum Off J Am Coll Rheumatol. 1986;29(8):1039–49. 17. Luyten FP, Denti M, Filardo G, Kon E, Engebretsen L. Definition and classification of early osteoarthritis of the knee. Knee Surgery, Sport Traumatol Arthrosc. 2012;20(3):401–6. 18. Hoemann C, Kandel R, Roberts S, Saris DBF, Creemers L, Mainil-Varlet P, et al. International Cartilage Repair Society (ICRS) Recommended Guidelines for  Histological Endpoints for Cartilage Repair Studies in Animal Models and Clinical Trials. Cartilage. 2011 Apr;2(2):153–72. 19. Hunter DJ, Lo GH, Gale D, Grainger AJ, Guermazi A, Conaghan PG. The reliability of a new scoring system for knee osteoarthritis MRI and the validity of bone marrow lesion assessment: BLOKS (Boston–Leeds Osteoarthritis Knee Score). Ann Rheum Dis. 2008;67(2):206–11. 20. Peterfy CG, Guermazi A, Zaim S, Tirman PFJ, Miaux Y, White D, et al. Whole-organ magnetic resonance imaging score (WORMS) of the knee in osteoarthritis. Osteoarthr Cartil. 2004;12(3):177–90. 21. Demange MK, Sisto M, Rodeo S. Future trends for unicompartmental arthritis of the knee: injectables & stem cells. Clin Sports Med. 2014;33(1):161–74. 22. Leslie M. Knee osteoarthritis management therapies. Pain Manag Nurs. 2000;1(2):51–7. 23. Paoloni M, Bernetti A, Belelli A, Brignoli O, Buoso S, Caputi AP, et al. Appropriateness of clinical and organizational criteria for intra-articular injection therapies in osteoarthritis: A Delphi method consensus initiative among experts in Italy. Ann Ist Super Sanita. 2015;51:131–8. 24. Sandmark H, Hogstedt C, Lewold S, Vingård E. Osteoarthrosis of the knee in men and women in association with overweight, smoking, and hormone therapy. Ann Rheum Dis. 1999;58(3):151–5. 25. Christensen BV, Iuhl IU, Vilbek H, Bülow H, Dreijer NC, Rasmussen HF. Acupuncture treatment of severe knee osteoarthrosis. A long‐term study. Acta Anaesthesiol Scand. 1992;36(6):519–25. 26. de Sire A, Agostini F, Lippi L, Mangone M, Marchese S, Cisari C, et al. Oxygen-Ozone Therapy in the Rehabilitation Field: State of the Art on Mechanisms of  Action, Safety and Effectiveness in Patients with Musculoskeletal Disorders. Biomolecules. 2021 Feb;11(3). 27. Fransen M, McCONNELL S. Land-based exercise for osteoarthritis of the knee: a metaanalysis of randomized controlled trials. J Rheumatol. 2009;36(6):1109–17. 28. Hiemstra LA, Kerslake S, Irving C. Anterior knee pain in the athlete. Clin Sports Med. 2014;33(3):437–59. 29. Filardo G, Kon E, Di Matteo B, Pelotti P, Di Martino A, Marcacci M. Platelet-rich plasma for the treatment of patellar tendinopathy: clinical and imaging findings at medium-term follow-up. Int Orthop [Internet]. 2013;37(8):1583–9. Available from: https://doi.org/10.1007/s00264-013-1972-830. Trojian TH, Concoff AL, Joy SM, Hatzenbuehler JR, Saulsberry WJ, Coleman CI. AMSSM Scientific Statement Concerning Viscosupplementation Injections for Knee  Osteoarthritis: Importance for Individual Patient Outcomes. Clin J Sport Med  Off J Can Acad  Sport Med. 2016 Jan;26(1):1–11. 31. Ammar TY, Pereira TAP, Mistura SLL, Kuhn A, Saggin JI, Lopes OV. Viscosupplementation for treating knee osteoarthrosis: review of the literature. Rev Bras Ortop. 2015;50:489–94. 32. Salini V, Pantalone A, Vanni D, Abate M. Viscosupplementation in Athletes. In: Arthroscopy and Sport Injuries. Springer; 2016. p. 433–7. 33. Erdem Y, Gul D, Akpancar S. Comparison of Intraarticular Injections of Hyaluronic Acid versus Dextrose Applied with Periarticular Prolotherapy in the Treatment of Recreational Athletes with Knee Osteoarthritis. Turkish J Sport Med. 2020;55(1):6–13. 34. Kalkhoven J, Coutts AJ, Impellizzeri FM. 'Training load error' is not a more accurate term than 'overuse' injury. Br J Sports Med [Internet]. 2020 Aug 1;54(15):934 LP – 935. Available from: http://bjsm.bmj.com/content/54/15/934.abstract35. Odole AC, Agbomeji OT, Onyeso OKK, Ojo JO, Odunaiya NA. Perspectives of Nigerian Athletes About Physiotherapy Services in Sports Injury Management: Implications for Rehabilitation. J Sport Rehabil. 2021;1(aop):1–8. 36. Hendrika W, Reswari A. The effect of physiotherapy on pain improvement in patients with early knee osteoarthritis at RSU UKI. Int J Med Heal Res. 2021;7(6):52–9. 37. MacKay C, Hawker GA, Jaglal SB. How Do Physical Therapists Approach Management of People With Early Knee Osteoarthritis? A Qualitative Study. Phys Ther [Internet]. 2019;100(2):295–306. Available from: https://doi.org/10.1093/ptj/pzz16438. Hrazdira L, Pilecki Z, Pilecki G. Ultrasound guided application of HA in the soft tissues of the locomotor system in athletes–First experience. Krátká sdělení Botek, M, Stejskal, P, Klimešová, I Vliv normobarické hypoxie na Akt Auton Nerv systému Pilot Stud 191 Drbošalová, V, Stejskal, P, Klugar, M, Krejčíř, V, Hynková, O Charakt klientů komerčn. :205. 39. Ricci M, Micheloni GM, Berti M, Perusi F, Sambugaro E, Vecchini E, et al. Clinical comparison of oral administration and viscosupplementation of hyaluronic acid (HA) in early knee osteoarthritis. Musculoskelet Surg. 2017;101(1):45–9. 40. Wang C-T, Lin Y-T, Chiang B-L, Lin Y-H, Hou S-M. High molecular weight hyaluronic acid down-regulates the gene expression of osteoarthritis-associated cytokines and enzymes in fibroblast-like synoviocytes from patients with early osteoarthritis. Osteoarthr Cartil. 2006;14(12):1237–47. 41. Irrgang JJ, Anderson AF. Development and validation of health-related quality of life measures for the knee. Clin Orthop Relat Res. 2002;402:95–109. 42. Roos EM, Roos HP, Lohmander LS, Ekdahl C, Beynnon BD. Knee Injury and Osteoarthritis Outcome Score (KOOS)—development of a self-administered outcome measure. J Orthop Sport Phys Ther. 1998;28(2):88–96. 43. Perruccio A V, Canizares M, Hawker GA, Roos EM, Maillefert JF, Lohmander SL. Comparative Validity and Responsiveness of the HOOS-PS and KOOS-PS to the WOMAC Physical Function Subscale in Total Joint Replacement for Osteoarthritis. 44. Salavati M, Mazaheri M, Negahban H, Sohani SM, Ebrahimian MR, Ebrahimi I, et al. Validation of a Persian-version of Knee injury and Osteoarthritis Outcome Score (KOOS) in Iranians with knee injuries. Osteoarthr Cartil. 2008;16(10):1178–82. 45. Higgins LD, Taylor MK, Park D, Ghodadra N, Marchant M, Pietrobon R, et al. Reliability and validity of the International Knee Documentation Committee (IKDC) subjective knee form. Jt Bone Spine. 2007;74(6):594–9. 46. Kujala UM, Kaprio J, Sarno S. Osteoarthritis of weight bearing joints of lower limbs in former elite male athletes. Bmj. 1994;308(6923):231–4. 47. Salini V, Pantalone A, Vanni D. Arthroscopy and Sport Injuries. Arthrosc Sport Inj. 2016;433–7. 48. Broglio K. Randomization in clinical trials: Permuted blocks and stratification. JAMA - J Am Med Assoc. 2018;319(21):2223–4. 49. Emanuel EJ, Wendler D, Grady C. What makes clinical research ethical? JAMA. 2000 May;283(20):2701–11. )

[45.	Higgins LD, Taylor MK, Park D, Ghodadra N, Marchant M, Pietrobon R, et al. Reliability and validity of the International Knee Documentation Committee (IKDC) subjective knee form. Jt Bone Spine. 2007;74(6):594–9.](1. Patel DR, Villalobos A. Evaluation and management of knee pain in young athletes: overuse injuries of the knee. Transl Pediatr [Internet]. 2017 Jul;6(3):190–8. Available from: https://pubmed.ncbi.nlm.nih.gov/287950102. Nicolini AP, de Carvalho RT, Matsuda MM, Filho JS, Cohen M. Common injuries in athletes' knee: Experience of a specialized center. Acta Ortop Bras. 2014;22(3):127–31. 3. Tiu T, Craig Van Dien MD. Knee overuse disorders. 4. Fuller CW, Ekstrand J, Junge A, Andersen TE, Bahr R, Dvorak J, et al. Consensus statement on injury definitions and data collection procedures in studies  of football (soccer) injuries. Br J Sports Med. 2006 Mar;40(3):193–201. 5. Majewski M, Susanne H, Klaus S. Epidemiology of athletic knee injuries: A 10-year study. Knee. 2006 Jun;13(3):184–8. 6. Wilder RP, Sethi S. Overuse injuries: tendinopathies, stress fractures, compartment syndrome, and shin  splints. Clin Sports Med. 2004 Jan;23(1):55–81, vi. 7. Aicale R, Tarantino D, Maffulli N. Overuse injuries in sport: a comprehensive overview. J Orthop Surg Res. 2018 Dec;13(1):309. 8. Yang J, Tibbetts AS, Covassin T, Cheng G, Nayar S, Heiden E. Epidemiology of overuse and acute injuries among competitive collegiate athletes. J Athl Train. 2012;47(2):198–204. 9. Bernetti A, Mangone M, Paoloni M, Di Sante L, Murgia M, Santilli V. Corticosteroid and hyaluronic acid injection therapy in tennis elbow (lateral epicondylalgia). Med Sport. 2014;67(2):289–95. 10. Iolascon G, Gimigliano F, Moretti A, De Sire A, Migliore A, Brandi ML, et al. Early osteoarthritis: How to define, diagnose, and manage. A systematic review. Eur Geriatr Med. 2017;8(5–6):383–96. 11. Drawer S, Fuller CW. Propensity for osteoarthritis and lower limb joint pain in retired professional soccer players. Br J Sports Med. 2001;35(6):402–8. 12. Vannini F, Spalding T, Andriolo L, Berruto M, Denti M, Espregueira-Mendes J, et al. Sport and early osteoarthritis: the role of sport in aetiology, progression and treatment of knee osteoarthritis. Knee Surgery, Sport Traumatol Arthrosc. 2016;24(6):1786–96. 13. Fröhlich S, Pazeller S, Cherati AS, Müller E, Frey WO, Spörri J. Overuse injuries in the knee, back and hip of top elite female alpine skiers during  the off-season preparation period: prevalence, severity and their association with traumatic preinjuries and training load. BMJ open Sport Exerc Med. 2020;6(1):e000892. 14. Rincon GA, Vyas D, Zhou J, Fu FH, Oiestad BE, Holm I, et al. \“ Knee function and prevalence of knee osteoarthritis after anterior cruciate ligament reconstruction: a prospective study with 10 to 15 years of follow-up\”. Letter to the editor. Am J Sports Med. 2011;39(4):NP3-author. 15. Kellgren JH, Lawrence J. Radiological assessment of osteo-arthrosis. Ann Rheum Dis. 1957;16(4):494. 16. Altman R, Asch E, Bloch D, Bole G, Borenstein D, Brandt K, et al. Development of criteria for the classification and reporting of osteoarthritis: classification of osteoarthritis of the knee. Arthritis Rheum Off J Am Coll Rheumatol. 1986;29(8):1039–49. 17. Luyten FP, Denti M, Filardo G, Kon E, Engebretsen L. Definition and classification of early osteoarthritis of the knee. Knee Surgery, Sport Traumatol Arthrosc. 2012;20(3):401–6. 18. Hoemann C, Kandel R, Roberts S, Saris DBF, Creemers L, Mainil-Varlet P, et al. International Cartilage Repair Society (ICRS) Recommended Guidelines for  Histological Endpoints for Cartilage Repair Studies in Animal Models and Clinical Trials. Cartilage. 2011 Apr;2(2):153–72. 19. Hunter DJ, Lo GH, Gale D, Grainger AJ, Guermazi A, Conaghan PG. The reliability of a new scoring system for knee osteoarthritis MRI and the validity of bone marrow lesion assessment: BLOKS (Boston–Leeds Osteoarthritis Knee Score). Ann Rheum Dis. 2008;67(2):206–11. 20. Peterfy CG, Guermazi A, Zaim S, Tirman PFJ, Miaux Y, White D, et al. Whole-organ magnetic resonance imaging score (WORMS) of the knee in osteoarthritis. Osteoarthr Cartil. 2004;12(3):177–90. 21. Demange MK, Sisto M, Rodeo S. Future trends for unicompartmental arthritis of the knee: injectables & stem cells. Clin Sports Med. 2014;33(1):161–74. 22. Leslie M. Knee osteoarthritis management therapies. Pain Manag Nurs. 2000;1(2):51–7. 23. Paoloni M, Bernetti A, Belelli A, Brignoli O, Buoso S, Caputi AP, et al. Appropriateness of clinical and organizational criteria for intra-articular injection therapies in osteoarthritis: A Delphi method consensus initiative among experts in Italy. Ann Ist Super Sanita. 2015;51:131–8. 24. Sandmark H, Hogstedt C, Lewold S, Vingård E. Osteoarthrosis of the knee in men and women in association with overweight, smoking, and hormone therapy. Ann Rheum Dis. 1999;58(3):151–5. 25. Christensen BV, Iuhl IU, Vilbek H, Bülow H, Dreijer NC, Rasmussen HF. Acupuncture treatment of severe knee osteoarthrosis. A long‐term study. Acta Anaesthesiol Scand. 1992;36(6):519–25. 26. de Sire A, Agostini F, Lippi L, Mangone M, Marchese S, Cisari C, et al. Oxygen-Ozone Therapy in the Rehabilitation Field: State of the Art on Mechanisms of  Action, Safety and Effectiveness in Patients with Musculoskeletal Disorders. Biomolecules. 2021 Feb;11(3). 27. Fransen M, McCONNELL S. Land-based exercise for osteoarthritis of the knee: a metaanalysis of randomized controlled trials. J Rheumatol. 2009;36(6):1109–17. 28. Hiemstra LA, Kerslake S, Irving C. Anterior knee pain in the athlete. Clin Sports Med. 2014;33(3):437–59. 29. Filardo G, Kon E, Di Matteo B, Pelotti P, Di Martino A, Marcacci M. Platelet-rich plasma for the treatment of patellar tendinopathy: clinical and imaging findings at medium-term follow-up. Int Orthop [Internet]. 2013;37(8):1583–9. Available from: https://doi.org/10.1007/s00264-013-1972-830. Trojian TH, Concoff AL, Joy SM, Hatzenbuehler JR, Saulsberry WJ, Coleman CI. AMSSM Scientific Statement Concerning Viscosupplementation Injections for Knee  Osteoarthritis: Importance for Individual Patient Outcomes. Clin J Sport Med  Off J Can Acad  Sport Med. 2016 Jan;26(1):1–11. 31. Ammar TY, Pereira TAP, Mistura SLL, Kuhn A, Saggin JI, Lopes OV. Viscosupplementation for treating knee osteoarthrosis: review of the literature. Rev Bras Ortop. 2015;50:489–94. 32. Salini V, Pantalone A, Vanni D, Abate M. Viscosupplementation in Athletes. In: Arthroscopy and Sport Injuries. Springer; 2016. p. 433–7. 33. Erdem Y, Gul D, Akpancar S. Comparison of Intraarticular Injections of Hyaluronic Acid versus Dextrose Applied with Periarticular Prolotherapy in the Treatment of Recreational Athletes with Knee Osteoarthritis. Turkish J Sport Med. 2020;55(1):6–13. 34. Kalkhoven J, Coutts AJ, Impellizzeri FM. 'Training load error' is not a more accurate term than 'overuse' injury. Br J Sports Med [Internet]. 2020 Aug 1;54(15):934 LP – 935. Available from: http://bjsm.bmj.com/content/54/15/934.abstract35. Odole AC, Agbomeji OT, Onyeso OKK, Ojo JO, Odunaiya NA. Perspectives of Nigerian Athletes About Physiotherapy Services in Sports Injury Management: Implications for Rehabilitation. J Sport Rehabil. 2021;1(aop):1–8. 36. Hendrika W, Reswari A. The effect of physiotherapy on pain improvement in patients with early knee osteoarthritis at RSU UKI. Int J Med Heal Res. 2021;7(6):52–9. 37. MacKay C, Hawker GA, Jaglal SB. How Do Physical Therapists Approach Management of People With Early Knee Osteoarthritis? A Qualitative Study. Phys Ther [Internet]. 2019;100(2):295–306. Available from: https://doi.org/10.1093/ptj/pzz16438. Hrazdira L, Pilecki Z, Pilecki G. Ultrasound guided application of HA in the soft tissues of the locomotor system in athletes–First experience. Krátká sdělení Botek, M, Stejskal, P, Klimešová, I Vliv normobarické hypoxie na Akt Auton Nerv systému Pilot Stud 191 Drbošalová, V, Stejskal, P, Klugar, M, Krejčíř, V, Hynková, O Charakt klientů komerčn. :205. 39. Ricci M, Micheloni GM, Berti M, Perusi F, Sambugaro E, Vecchini E, et al. Clinical comparison of oral administration and viscosupplementation of hyaluronic acid (HA) in early knee osteoarthritis. Musculoskelet Surg. 2017;101(1):45–9. 40. Wang C-T, Lin Y-T, Chiang B-L, Lin Y-H, Hou S-M. High molecular weight hyaluronic acid down-regulates the gene expression of osteoarthritis-associated cytokines and enzymes in fibroblast-like synoviocytes from patients with early osteoarthritis. Osteoarthr Cartil. 2006;14(12):1237–47. 41. Irrgang JJ, Anderson AF. Development and validation of health-related quality of life measures for the knee. Clin Orthop Relat Res. 2002;402:95–109. 42. Roos EM, Roos HP, Lohmander LS, Ekdahl C, Beynnon BD. Knee Injury and Osteoarthritis Outcome Score (KOOS)—development of a self-administered outcome measure. J Orthop Sport Phys Ther. 1998;28(2):88–96. 43. Perruccio A V, Canizares M, Hawker GA, Roos EM, Maillefert JF, Lohmander SL. Comparative Validity and Responsiveness of the HOOS-PS and KOOS-PS to the WOMAC Physical Function Subscale in Total Joint Replacement for Osteoarthritis. 44. Salavati M, Mazaheri M, Negahban H, Sohani SM, Ebrahimian MR, Ebrahimi I, et al. Validation of a Persian-version of Knee injury and Osteoarthritis Outcome Score (KOOS) in Iranians with knee injuries. Osteoarthr Cartil. 2008;16(10):1178–82. 45. Higgins LD, Taylor MK, Park D, Ghodadra N, Marchant M, Pietrobon R, et al. Reliability and validity of the International Knee Documentation Committee (IKDC) subjective knee form. Jt Bone Spine. 2007;74(6):594–9. 46. Kujala UM, Kaprio J, Sarno S. Osteoarthritis of weight bearing joints of lower limbs in former elite male athletes. Bmj. 1994;308(6923):231–4. 47. Salini V, Pantalone A, Vanni D. Arthroscopy and Sport Injuries. Arthrosc Sport Inj. 2016;433–7. 48. Broglio K. Randomization in clinical trials: Permuted blocks and stratification. JAMA - J Am Med Assoc. 2018;319(21):2223–4. 49. Emanuel EJ, Wendler D, Grady C. What makes clinical research ethical? JAMA. 2000 May;283(20):2701–11. )

[46.	Kujala UM, Kaprio J, Sarno S. Osteoarthritis of weight bearing joints of lower limbs in former elite male athletes. Bmj. 1994;308(6923):231–4.](1. Patel DR, Villalobos A. Evaluation and management of knee pain in young athletes: overuse injuries of the knee. Transl Pediatr [Internet]. 2017 Jul;6(3):190–8. Available from: https://pubmed.ncbi.nlm.nih.gov/287950102. Nicolini AP, de Carvalho RT, Matsuda MM, Filho JS, Cohen M. Common injuries in athletes' knee: Experience of a specialized center. Acta Ortop Bras. 2014;22(3):127–31. 3. Tiu T, Craig Van Dien MD. Knee overuse disorders. 4. Fuller CW, Ekstrand J, Junge A, Andersen TE, Bahr R, Dvorak J, et al. Consensus statement on injury definitions and data collection procedures in studies  of football (soccer) injuries. Br J Sports Med. 2006 Mar;40(3):193–201. 5. Majewski M, Susanne H, Klaus S. Epidemiology of athletic knee injuries: A 10-year study. Knee. 2006 Jun;13(3):184–8. 6. Wilder RP, Sethi S. Overuse injuries: tendinopathies, stress fractures, compartment syndrome, and shin  splints. Clin Sports Med. 2004 Jan;23(1):55–81, vi. 7. Aicale R, Tarantino D, Maffulli N. Overuse injuries in sport: a comprehensive overview. J Orthop Surg Res. 2018 Dec;13(1):309. 8. Yang J, Tibbetts AS, Covassin T, Cheng G, Nayar S, Heiden E. Epidemiology of overuse and acute injuries among competitive collegiate athletes. J Athl Train. 2012;47(2):198–204. 9. Bernetti A, Mangone M, Paoloni M, Di Sante L, Murgia M, Santilli V. Corticosteroid and hyaluronic acid injection therapy in tennis elbow (lateral epicondylalgia). Med Sport. 2014;67(2):289–95. 10. Iolascon G, Gimigliano F, Moretti A, De Sire A, Migliore A, Brandi ML, et al. Early osteoarthritis: How to define, diagnose, and manage. A systematic review. Eur Geriatr Med. 2017;8(5–6):383–96. 11. Drawer S, Fuller CW. Propensity for osteoarthritis and lower limb joint pain in retired professional soccer players. Br J Sports Med. 2001;35(6):402–8. 12. Vannini F, Spalding T, Andriolo L, Berruto M, Denti M, Espregueira-Mendes J, et al. Sport and early osteoarthritis: the role of sport in aetiology, progression and treatment of knee osteoarthritis. Knee Surgery, Sport Traumatol Arthrosc. 2016;24(6):1786–96. 13. Fröhlich S, Pazeller S, Cherati AS, Müller E, Frey WO, Spörri J. Overuse injuries in the knee, back and hip of top elite female alpine skiers during  the off-season preparation period: prevalence, severity and their association with traumatic preinjuries and training load. BMJ open Sport Exerc Med. 2020;6(1):e000892. 14. Rincon GA, Vyas D, Zhou J, Fu FH, Oiestad BE, Holm I, et al. \“ Knee function and prevalence of knee osteoarthritis after anterior cruciate ligament reconstruction: a prospective study with 10 to 15 years of follow-up\”. Letter to the editor. Am J Sports Med. 2011;39(4):NP3-author. 15. Kellgren JH, Lawrence J. Radiological assessment of osteo-arthrosis. Ann Rheum Dis. 1957;16(4):494. 16. Altman R, Asch E, Bloch D, Bole G, Borenstein D, Brandt K, et al. Development of criteria for the classification and reporting of osteoarthritis: classification of osteoarthritis of the knee. Arthritis Rheum Off J Am Coll Rheumatol. 1986;29(8):1039–49. 17. Luyten FP, Denti M, Filardo G, Kon E, Engebretsen L. Definition and classification of early osteoarthritis of the knee. Knee Surgery, Sport Traumatol Arthrosc. 2012;20(3):401–6. 18. Hoemann C, Kandel R, Roberts S, Saris DBF, Creemers L, Mainil-Varlet P, et al. International Cartilage Repair Society (ICRS) Recommended Guidelines for  Histological Endpoints for Cartilage Repair Studies in Animal Models and Clinical Trials. Cartilage. 2011 Apr;2(2):153–72. 19. Hunter DJ, Lo GH, Gale D, Grainger AJ, Guermazi A, Conaghan PG. The reliability of a new scoring system for knee osteoarthritis MRI and the validity of bone marrow lesion assessment: BLOKS (Boston–Leeds Osteoarthritis Knee Score). Ann Rheum Dis. 2008;67(2):206–11. 20. Peterfy CG, Guermazi A, Zaim S, Tirman PFJ, Miaux Y, White D, et al. Whole-organ magnetic resonance imaging score (WORMS) of the knee in osteoarthritis. Osteoarthr Cartil. 2004;12(3):177–90. 21. Demange MK, Sisto M, Rodeo S. Future trends for unicompartmental arthritis of the knee: injectables & stem cells. Clin Sports Med. 2014;33(1):161–74. 22. Leslie M. Knee osteoarthritis management therapies. Pain Manag Nurs. 2000;1(2):51–7. 23. Paoloni M, Bernetti A, Belelli A, Brignoli O, Buoso S, Caputi AP, et al. Appropriateness of clinical and organizational criteria for intra-articular injection therapies in osteoarthritis: A Delphi method consensus initiative among experts in Italy. Ann Ist Super Sanita. 2015;51:131–8. 24. Sandmark H, Hogstedt C, Lewold S, Vingård E. Osteoarthrosis of the knee in men and women in association with overweight, smoking, and hormone therapy. Ann Rheum Dis. 1999;58(3):151–5. 25. Christensen BV, Iuhl IU, Vilbek H, Bülow H, Dreijer NC, Rasmussen HF. Acupuncture treatment of severe knee osteoarthrosis. A long‐term study. Acta Anaesthesiol Scand. 1992;36(6):519–25. 26. de Sire A, Agostini F, Lippi L, Mangone M, Marchese S, Cisari C, et al. Oxygen-Ozone Therapy in the Rehabilitation Field: State of the Art on Mechanisms of  Action, Safety and Effectiveness in Patients with Musculoskeletal Disorders. Biomolecules. 2021 Feb;11(3). 27. Fransen M, McCONNELL S. Land-based exercise for osteoarthritis of the knee: a metaanalysis of randomized controlled trials. J Rheumatol. 2009;36(6):1109–17. 28. Hiemstra LA, Kerslake S, Irving C. Anterior knee pain in the athlete. Clin Sports Med. 2014;33(3):437–59. 29. Filardo G, Kon E, Di Matteo B, Pelotti P, Di Martino A, Marcacci M. Platelet-rich plasma for the treatment of patellar tendinopathy: clinical and imaging findings at medium-term follow-up. Int Orthop [Internet]. 2013;37(8):1583–9. Available from: https://doi.org/10.1007/s00264-013-1972-830. Trojian TH, Concoff AL, Joy SM, Hatzenbuehler JR, Saulsberry WJ, Coleman CI. AMSSM Scientific Statement Concerning Viscosupplementation Injections for Knee  Osteoarthritis: Importance for Individual Patient Outcomes. Clin J Sport Med  Off J Can Acad  Sport Med. 2016 Jan;26(1):1–11. 31. Ammar TY, Pereira TAP, Mistura SLL, Kuhn A, Saggin JI, Lopes OV. Viscosupplementation for treating knee osteoarthrosis: review of the literature. Rev Bras Ortop. 2015;50:489–94. 32. Salini V, Pantalone A, Vanni D, Abate M. Viscosupplementation in Athletes. In: Arthroscopy and Sport Injuries. Springer; 2016. p. 433–7. 33. Erdem Y, Gul D, Akpancar S. Comparison of Intraarticular Injections of Hyaluronic Acid versus Dextrose Applied with Periarticular Prolotherapy in the Treatment of Recreational Athletes with Knee Osteoarthritis. Turkish J Sport Med. 2020;55(1):6–13. 34. Kalkhoven J, Coutts AJ, Impellizzeri FM. 'Training load error' is not a more accurate term than 'overuse' injury. Br J Sports Med [Internet]. 2020 Aug 1;54(15):934 LP – 935. Available from: http://bjsm.bmj.com/content/54/15/934.abstract35. Odole AC, Agbomeji OT, Onyeso OKK, Ojo JO, Odunaiya NA. Perspectives of Nigerian Athletes About Physiotherapy Services in Sports Injury Management: Implications for Rehabilitation. J Sport Rehabil. 2021;1(aop):1–8. 36. Hendrika W, Reswari A. The effect of physiotherapy on pain improvement in patients with early knee osteoarthritis at RSU UKI. Int J Med Heal Res. 2021;7(6):52–9. 37. MacKay C, Hawker GA, Jaglal SB. How Do Physical Therapists Approach Management of People With Early Knee Osteoarthritis? A Qualitative Study. Phys Ther [Internet]. 2019;100(2):295–306. Available from: https://doi.org/10.1093/ptj/pzz16438. Hrazdira L, Pilecki Z, Pilecki G. Ultrasound guided application of HA in the soft tissues of the locomotor system in athletes–First experience. Krátká sdělení Botek, M, Stejskal, P, Klimešová, I Vliv normobarické hypoxie na Akt Auton Nerv systému Pilot Stud 191 Drbošalová, V, Stejskal, P, Klugar, M, Krejčíř, V, Hynková, O Charakt klientů komerčn. :205. 39. Ricci M, Micheloni GM, Berti M, Perusi F, Sambugaro E, Vecchini E, et al. Clinical comparison of oral administration and viscosupplementation of hyaluronic acid (HA) in early knee osteoarthritis. Musculoskelet Surg. 2017;101(1):45–9. 40. Wang C-T, Lin Y-T, Chiang B-L, Lin Y-H, Hou S-M. High molecular weight hyaluronic acid down-regulates the gene expression of osteoarthritis-associated cytokines and enzymes in fibroblast-like synoviocytes from patients with early osteoarthritis. Osteoarthr Cartil. 2006;14(12):1237–47. 41. Irrgang JJ, Anderson AF. Development and validation of health-related quality of life measures for the knee. Clin Orthop Relat Res. 2002;402:95–109. 42. Roos EM, Roos HP, Lohmander LS, Ekdahl C, Beynnon BD. Knee Injury and Osteoarthritis Outcome Score (KOOS)—development of a self-administered outcome measure. J Orthop Sport Phys Ther. 1998;28(2):88–96. 43. Perruccio A V, Canizares M, Hawker GA, Roos EM, Maillefert JF, Lohmander SL. Comparative Validity and Responsiveness of the HOOS-PS and KOOS-PS to the WOMAC Physical Function Subscale in Total Joint Replacement for Osteoarthritis. 44. Salavati M, Mazaheri M, Negahban H, Sohani SM, Ebrahimian MR, Ebrahimi I, et al. Validation of a Persian-version of Knee injury and Osteoarthritis Outcome Score (KOOS) in Iranians with knee injuries. Osteoarthr Cartil. 2008;16(10):1178–82. 45. Higgins LD, Taylor MK, Park D, Ghodadra N, Marchant M, Pietrobon R, et al. Reliability and validity of the International Knee Documentation Committee (IKDC) subjective knee form. Jt Bone Spine. 2007;74(6):594–9. 46. Kujala UM, Kaprio J, Sarno S. Osteoarthritis of weight bearing joints of lower limbs in former elite male athletes. Bmj. 1994;308(6923):231–4. 47. Salini V, Pantalone A, Vanni D. Arthroscopy and Sport Injuries. Arthrosc Sport Inj. 2016;433–7. 48. Broglio K. Randomization in clinical trials: Permuted blocks and stratification. JAMA - J Am Med Assoc. 2018;319(21):2223–4. 49. Emanuel EJ, Wendler D, Grady C. What makes clinical research ethical? JAMA. 2000 May;283(20):2701–11. )

[47.	Salini V, Pantalone A, Vanni D. Arthroscopy and Sport Injuries. Arthrosc Sport Inj. 2016;433–7.](1. Patel DR, Villalobos A. Evaluation and management of knee pain in young athletes: overuse injuries of the knee. Transl Pediatr [Internet]. 2017 Jul;6(3):190–8. Available from: https://pubmed.ncbi.nlm.nih.gov/287950102. Nicolini AP, de Carvalho RT, Matsuda MM, Filho JS, Cohen M. Common injuries in athletes' knee: Experience of a specialized center. Acta Ortop Bras. 2014;22(3):127–31. 3. Tiu T, Craig Van Dien MD. Knee overuse disorders. 4. Fuller CW, Ekstrand J, Junge A, Andersen TE, Bahr R, Dvorak J, et al. Consensus statement on injury definitions and data collection procedures in studies  of football (soccer) injuries. Br J Sports Med. 2006 Mar;40(3):193–201. 5. Majewski M, Susanne H, Klaus S. Epidemiology of athletic knee injuries: A 10-year study. Knee. 2006 Jun;13(3):184–8. 6. Wilder RP, Sethi S. Overuse injuries: tendinopathies, stress fractures, compartment syndrome, and shin  splints. Clin Sports Med. 2004 Jan;23(1):55–81, vi. 7. Aicale R, Tarantino D, Maffulli N. Overuse injuries in sport: a comprehensive overview. J Orthop Surg Res. 2018 Dec;13(1):309. 8. Yang J, Tibbetts AS, Covassin T, Cheng G, Nayar S, Heiden E. Epidemiology of overuse and acute injuries among competitive collegiate athletes. J Athl Train. 2012;47(2):198–204. 9. Bernetti A, Mangone M, Paoloni M, Di Sante L, Murgia M, Santilli V. Corticosteroid and hyaluronic acid injection therapy in tennis elbow (lateral epicondylalgia). Med Sport. 2014;67(2):289–95. 10. Iolascon G, Gimigliano F, Moretti A, De Sire A, Migliore A, Brandi ML, et al. Early osteoarthritis: How to define, diagnose, and manage. A systematic review. Eur Geriatr Med. 2017;8(5–6):383–96. 11. Drawer S, Fuller CW. Propensity for osteoarthritis and lower limb joint pain in retired professional soccer players. Br J Sports Med. 2001;35(6):402–8. 12. Vannini F, Spalding T, Andriolo L, Berruto M, Denti M, Espregueira-Mendes J, et al. Sport and early osteoarthritis: the role of sport in aetiology, progression and treatment of knee osteoarthritis. Knee Surgery, Sport Traumatol Arthrosc. 2016;24(6):1786–96. 13. Fröhlich S, Pazeller S, Cherati AS, Müller E, Frey WO, Spörri J. Overuse injuries in the knee, back and hip of top elite female alpine skiers during  the off-season preparation period: prevalence, severity and their association with traumatic preinjuries and training load. BMJ open Sport Exerc Med. 2020;6(1):e000892. 14. Rincon GA, Vyas D, Zhou J, Fu FH, Oiestad BE, Holm I, et al. \“ Knee function and prevalence of knee osteoarthritis after anterior cruciate ligament reconstruction: a prospective study with 10 to 15 years of follow-up\”. Letter to the editor. Am J Sports Med. 2011;39(4):NP3-author. 15. Kellgren JH, Lawrence J. Radiological assessment of osteo-arthrosis. Ann Rheum Dis. 1957;16(4):494. 16. Altman R, Asch E, Bloch D, Bole G, Borenstein D, Brandt K, et al. Development of criteria for the classification and reporting of osteoarthritis: classification of osteoarthritis of the knee. Arthritis Rheum Off J Am Coll Rheumatol. 1986;29(8):1039–49. 17. Luyten FP, Denti M, Filardo G, Kon E, Engebretsen L. Definition and classification of early osteoarthritis of the knee. Knee Surgery, Sport Traumatol Arthrosc. 2012;20(3):401–6. 18. Hoemann C, Kandel R, Roberts S, Saris DBF, Creemers L, Mainil-Varlet P, et al. International Cartilage Repair Society (ICRS) Recommended Guidelines for  Histological Endpoints for Cartilage Repair Studies in Animal Models and Clinical Trials. Cartilage. 2011 Apr;2(2):153–72. 19. Hunter DJ, Lo GH, Gale D, Grainger AJ, Guermazi A, Conaghan PG. The reliability of a new scoring system for knee osteoarthritis MRI and the validity of bone marrow lesion assessment: BLOKS (Boston–Leeds Osteoarthritis Knee Score). Ann Rheum Dis. 2008;67(2):206–11. 20. Peterfy CG, Guermazi A, Zaim S, Tirman PFJ, Miaux Y, White D, et al. Whole-organ magnetic resonance imaging score (WORMS) of the knee in osteoarthritis. Osteoarthr Cartil. 2004;12(3):177–90. 21. Demange MK, Sisto M, Rodeo S. Future trends for unicompartmental arthritis of the knee: injectables & stem cells. Clin Sports Med. 2014;33(1):161–74. 22. Leslie M. Knee osteoarthritis management therapies. Pain Manag Nurs. 2000;1(2):51–7. 23. Paoloni M, Bernetti A, Belelli A, Brignoli O, Buoso S, Caputi AP, et al. Appropriateness of clinical and organizational criteria for intra-articular injection therapies in osteoarthritis: A Delphi method consensus initiative among experts in Italy. Ann Ist Super Sanita. 2015;51:131–8. 24. Sandmark H, Hogstedt C, Lewold S, Vingård E. Osteoarthrosis of the knee in men and women in association with overweight, smoking, and hormone therapy. Ann Rheum Dis. 1999;58(3):151–5. 25. Christensen BV, Iuhl IU, Vilbek H, Bülow H, Dreijer NC, Rasmussen HF. Acupuncture treatment of severe knee osteoarthrosis. A long‐term study. Acta Anaesthesiol Scand. 1992;36(6):519–25. 26. de Sire A, Agostini F, Lippi L, Mangone M, Marchese S, Cisari C, et al. Oxygen-Ozone Therapy in the Rehabilitation Field: State of the Art on Mechanisms of  Action, Safety and Effectiveness in Patients with Musculoskeletal Disorders. Biomolecules. 2021 Feb;11(3). 27. Fransen M, McCONNELL S. Land-based exercise for osteoarthritis of the knee: a metaanalysis of randomized controlled trials. J Rheumatol. 2009;36(6):1109–17. 28. Hiemstra LA, Kerslake S, Irving C. Anterior knee pain in the athlete. Clin Sports Med. 2014;33(3):437–59. 29. Filardo G, Kon E, Di Matteo B, Pelotti P, Di Martino A, Marcacci M. Platelet-rich plasma for the treatment of patellar tendinopathy: clinical and imaging findings at medium-term follow-up. Int Orthop [Internet]. 2013;37(8):1583–9. Available from: https://doi.org/10.1007/s00264-013-1972-830. Trojian TH, Concoff AL, Joy SM, Hatzenbuehler JR, Saulsberry WJ, Coleman CI. AMSSM Scientific Statement Concerning Viscosupplementation Injections for Knee  Osteoarthritis: Importance for Individual Patient Outcomes. Clin J Sport Med  Off J Can Acad  Sport Med. 2016 Jan;26(1):1–11. 31. Ammar TY, Pereira TAP, Mistura SLL, Kuhn A, Saggin JI, Lopes OV. Viscosupplementation for treating knee osteoarthrosis: review of the literature. Rev Bras Ortop. 2015;50:489–94. 32. Salini V, Pantalone A, Vanni D, Abate M. Viscosupplementation in Athletes. In: Arthroscopy and Sport Injuries. Springer; 2016. p. 433–7. 33. Erdem Y, Gul D, Akpancar S. Comparison of Intraarticular Injections of Hyaluronic Acid versus Dextrose Applied with Periarticular Prolotherapy in the Treatment of Recreational Athletes with Knee Osteoarthritis. Turkish J Sport Med. 2020;55(1):6–13. 34. Kalkhoven J, Coutts AJ, Impellizzeri FM. 'Training load error' is not a more accurate term than 'overuse' injury. Br J Sports Med [Internet]. 2020 Aug 1;54(15):934 LP – 935. Available from: http://bjsm.bmj.com/content/54/15/934.abstract35. Odole AC, Agbomeji OT, Onyeso OKK, Ojo JO, Odunaiya NA. Perspectives of Nigerian Athletes About Physiotherapy Services in Sports Injury Management: Implications for Rehabilitation. J Sport Rehabil. 2021;1(aop):1–8. 36. Hendrika W, Reswari A. The effect of physiotherapy on pain improvement in patients with early knee osteoarthritis at RSU UKI. Int J Med Heal Res. 2021;7(6):52–9. 37. MacKay C, Hawker GA, Jaglal SB. How Do Physical Therapists Approach Management of People With Early Knee Osteoarthritis? A Qualitative Study. Phys Ther [Internet]. 2019;100(2):295–306. Available from: https://doi.org/10.1093/ptj/pzz16438. Hrazdira L, Pilecki Z, Pilecki G. Ultrasound guided application of HA in the soft tissues of the locomotor system in athletes–First experience. Krátká sdělení Botek, M, Stejskal, P, Klimešová, I Vliv normobarické hypoxie na Akt Auton Nerv systému Pilot Stud 191 Drbošalová, V, Stejskal, P, Klugar, M, Krejčíř, V, Hynková, O Charakt klientů komerčn. :205. 39. Ricci M, Micheloni GM, Berti M, Perusi F, Sambugaro E, Vecchini E, et al. Clinical comparison of oral administration and viscosupplementation of hyaluronic acid (HA) in early knee osteoarthritis. Musculoskelet Surg. 2017;101(1):45–9. 40. Wang C-T, Lin Y-T, Chiang B-L, Lin Y-H, Hou S-M. High molecular weight hyaluronic acid down-regulates the gene expression of osteoarthritis-associated cytokines and enzymes in fibroblast-like synoviocytes from patients with early osteoarthritis. Osteoarthr Cartil. 2006;14(12):1237–47. 41. Irrgang JJ, Anderson AF. Development and validation of health-related quality of life measures for the knee. Clin Orthop Relat Res. 2002;402:95–109. 42. Roos EM, Roos HP, Lohmander LS, Ekdahl C, Beynnon BD. Knee Injury and Osteoarthritis Outcome Score (KOOS)—development of a self-administered outcome measure. J Orthop Sport Phys Ther. 1998;28(2):88–96. 43. Perruccio A V, Canizares M, Hawker GA, Roos EM, Maillefert JF, Lohmander SL. Comparative Validity and Responsiveness of the HOOS-PS and KOOS-PS to the WOMAC Physical Function Subscale in Total Joint Replacement for Osteoarthritis. 44. Salavati M, Mazaheri M, Negahban H, Sohani SM, Ebrahimian MR, Ebrahimi I, et al. Validation of a Persian-version of Knee injury and Osteoarthritis Outcome Score (KOOS) in Iranians with knee injuries. Osteoarthr Cartil. 2008;16(10):1178–82. 45. Higgins LD, Taylor MK, Park D, Ghodadra N, Marchant M, Pietrobon R, et al. Reliability and validity of the International Knee Documentation Committee (IKDC) subjective knee form. Jt Bone Spine. 2007;74(6):594–9. 46. Kujala UM, Kaprio J, Sarno S. Osteoarthritis of weight bearing joints of lower limbs in former elite male athletes. Bmj. 1994;308(6923):231–4. 47. Salini V, Pantalone A, Vanni D. Arthroscopy and Sport Injuries. Arthrosc Sport Inj. 2016;433–7. 48. Broglio K. Randomization in clinical trials: Permuted blocks and stratification. JAMA - J Am Med Assoc. 2018;319(21):2223–4. 49. Emanuel EJ, Wendler D, Grady C. What makes clinical research ethical? JAMA. 2000 May;283(20):2701–11. )

[48.	Broglio K. Randomization in clinical trials: Permuted blocks and stratification. JAMA - J Am Med Assoc. 2018;319(21):2223–4.](1. Patel DR, Villalobos A. Evaluation and management of knee pain in young athletes: overuse injuries of the knee. Transl Pediatr [Internet]. 2017 Jul;6(3):190–8. Available from: https://pubmed.ncbi.nlm.nih.gov/287950102. Nicolini AP, de Carvalho RT, Matsuda MM, Filho JS, Cohen M. Common injuries in athletes' knee: Experience of a specialized center. Acta Ortop Bras. 2014;22(3):127–31. 3. Tiu T, Craig Van Dien MD. Knee overuse disorders. 4. Fuller CW, Ekstrand J, Junge A, Andersen TE, Bahr R, Dvorak J, et al. Consensus statement on injury definitions and data collection procedures in studies  of football (soccer) injuries. Br J Sports Med. 2006 Mar;40(3):193–201. 5. Majewski M, Susanne H, Klaus S. Epidemiology of athletic knee injuries: A 10-year study. Knee. 2006 Jun;13(3):184–8. 6. Wilder RP, Sethi S. Overuse injuries: tendinopathies, stress fractures, compartment syndrome, and shin  splints. Clin Sports Med. 2004 Jan;23(1):55–81, vi. 7. Aicale R, Tarantino D, Maffulli N. Overuse injuries in sport: a comprehensive overview. J Orthop Surg Res. 2018 Dec;13(1):309. 8. Yang J, Tibbetts AS, Covassin T, Cheng G, Nayar S, Heiden E. Epidemiology of overuse and acute injuries among competitive collegiate athletes. J Athl Train. 2012;47(2):198–204. 9. Bernetti A, Mangone M, Paoloni M, Di Sante L, Murgia M, Santilli V. Corticosteroid and hyaluronic acid injection therapy in tennis elbow (lateral epicondylalgia). Med Sport. 2014;67(2):289–95. 10. Iolascon G, Gimigliano F, Moretti A, De Sire A, Migliore A, Brandi ML, et al. Early osteoarthritis: How to define, diagnose, and manage. A systematic review. Eur Geriatr Med. 2017;8(5–6):383–96. 11. Drawer S, Fuller CW. Propensity for osteoarthritis and lower limb joint pain in retired professional soccer players. Br J Sports Med. 2001;35(6):402–8. 12. Vannini F, Spalding T, Andriolo L, Berruto M, Denti M, Espregueira-Mendes J, et al. Sport and early osteoarthritis: the role of sport in aetiology, progression and treatment of knee osteoarthritis. Knee Surgery, Sport Traumatol Arthrosc. 2016;24(6):1786–96. 13. Fröhlich S, Pazeller S, Cherati AS, Müller E, Frey WO, Spörri J. Overuse injuries in the knee, back and hip of top elite female alpine skiers during  the off-season preparation period: prevalence, severity and their association with traumatic preinjuries and training load. BMJ open Sport Exerc Med. 2020;6(1):e000892. 14. Rincon GA, Vyas D, Zhou J, Fu FH, Oiestad BE, Holm I, et al. \“ Knee function and prevalence of knee osteoarthritis after anterior cruciate ligament reconstruction: a prospective study with 10 to 15 years of follow-up\”. Letter to the editor. Am J Sports Med. 2011;39(4):NP3-author. 15. Kellgren JH, Lawrence J. Radiological assessment of osteo-arthrosis. Ann Rheum Dis. 1957;16(4):494. 16. Altman R, Asch E, Bloch D, Bole G, Borenstein D, Brandt K, et al. Development of criteria for the classification and reporting of osteoarthritis: classification of osteoarthritis of the knee. Arthritis Rheum Off J Am Coll Rheumatol. 1986;29(8):1039–49. 17. Luyten FP, Denti M, Filardo G, Kon E, Engebretsen L. Definition and classification of early osteoarthritis of the knee. Knee Surgery, Sport Traumatol Arthrosc. 2012;20(3):401–6. 18. Hoemann C, Kandel R, Roberts S, Saris DBF, Creemers L, Mainil-Varlet P, et al. International Cartilage Repair Society (ICRS) Recommended Guidelines for  Histological Endpoints for Cartilage Repair Studies in Animal Models and Clinical Trials. Cartilage. 2011 Apr;2(2):153–72. 19. Hunter DJ, Lo GH, Gale D, Grainger AJ, Guermazi A, Conaghan PG. The reliability of a new scoring system for knee osteoarthritis MRI and the validity of bone marrow lesion assessment: BLOKS (Boston–Leeds Osteoarthritis Knee Score). Ann Rheum Dis. 2008;67(2):206–11. 20. Peterfy CG, Guermazi A, Zaim S, Tirman PFJ, Miaux Y, White D, et al. Whole-organ magnetic resonance imaging score (WORMS) of the knee in osteoarthritis. Osteoarthr Cartil. 2004;12(3):177–90. 21. Demange MK, Sisto M, Rodeo S. Future trends for unicompartmental arthritis of the knee: injectables & stem cells. Clin Sports Med. 2014;33(1):161–74. 22. Leslie M. Knee osteoarthritis management therapies. Pain Manag Nurs. 2000;1(2):51–7. 23. Paoloni M, Bernetti A, Belelli A, Brignoli O, Buoso S, Caputi AP, et al. Appropriateness of clinical and organizational criteria for intra-articular injection therapies in osteoarthritis: A Delphi method consensus initiative among experts in Italy. Ann Ist Super Sanita. 2015;51:131–8. 24. Sandmark H, Hogstedt C, Lewold S, Vingård E. Osteoarthrosis of the knee in men and women in association with overweight, smoking, and hormone therapy. Ann Rheum Dis. 1999;58(3):151–5. 25. Christensen BV, Iuhl IU, Vilbek H, Bülow H, Dreijer NC, Rasmussen HF. Acupuncture treatment of severe knee osteoarthrosis. A long‐term study. Acta Anaesthesiol Scand. 1992;36(6):519–25. 26. de Sire A, Agostini F, Lippi L, Mangone M, Marchese S, Cisari C, et al. Oxygen-Ozone Therapy in the Rehabilitation Field: State of the Art on Mechanisms of  Action, Safety and Effectiveness in Patients with Musculoskeletal Disorders. Biomolecules. 2021 Feb;11(3). 27. Fransen M, McCONNELL S. Land-based exercise for osteoarthritis of the knee: a metaanalysis of randomized controlled trials. J Rheumatol. 2009;36(6):1109–17. 28. Hiemstra LA, Kerslake S, Irving C. Anterior knee pain in the athlete. Clin Sports Med. 2014;33(3):437–59. 29. Filardo G, Kon E, Di Matteo B, Pelotti P, Di Martino A, Marcacci M. Platelet-rich plasma for the treatment of patellar tendinopathy: clinical and imaging findings at medium-term follow-up. Int Orthop [Internet]. 2013;37(8):1583–9. Available from: https://doi.org/10.1007/s00264-013-1972-830. Trojian TH, Concoff AL, Joy SM, Hatzenbuehler JR, Saulsberry WJ, Coleman CI. AMSSM Scientific Statement Concerning Viscosupplementation Injections for Knee  Osteoarthritis: Importance for Individual Patient Outcomes. Clin J Sport Med  Off J Can Acad  Sport Med. 2016 Jan;26(1):1–11. 31. Ammar TY, Pereira TAP, Mistura SLL, Kuhn A, Saggin JI, Lopes OV. Viscosupplementation for treating knee osteoarthrosis: review of the literature. Rev Bras Ortop. 2015;50:489–94. 32. Salini V, Pantalone A, Vanni D, Abate M. Viscosupplementation in Athletes. In: Arthroscopy and Sport Injuries. Springer; 2016. p. 433–7. 33. Erdem Y, Gul D, Akpancar S. Comparison of Intraarticular Injections of Hyaluronic Acid versus Dextrose Applied with Periarticular Prolotherapy in the Treatment of Recreational Athletes with Knee Osteoarthritis. Turkish J Sport Med. 2020;55(1):6–13. 34. Kalkhoven J, Coutts AJ, Impellizzeri FM. 'Training load error' is not a more accurate term than 'overuse' injury. Br J Sports Med [Internet]. 2020 Aug 1;54(15):934 LP – 935. Available from: http://bjsm.bmj.com/content/54/15/934.abstract35. Odole AC, Agbomeji OT, Onyeso OKK, Ojo JO, Odunaiya NA. Perspectives of Nigerian Athletes About Physiotherapy Services in Sports Injury Management: Implications for Rehabilitation. J Sport Rehabil. 2021;1(aop):1–8. 36. Hendrika W, Reswari A. The effect of physiotherapy on pain improvement in patients with early knee osteoarthritis at RSU UKI. Int J Med Heal Res. 2021;7(6):52–9. 37. MacKay C, Hawker GA, Jaglal SB. How Do Physical Therapists Approach Management of People With Early Knee Osteoarthritis? A Qualitative Study. Phys Ther [Internet]. 2019;100(2):295–306. Available from: https://doi.org/10.1093/ptj/pzz16438. Hrazdira L, Pilecki Z, Pilecki G. Ultrasound guided application of HA in the soft tissues of the locomotor system in athletes–First experience. Krátká sdělení Botek, M, Stejskal, P, Klimešová, I Vliv normobarické hypoxie na Akt Auton Nerv systému Pilot Stud 191 Drbošalová, V, Stejskal, P, Klugar, M, Krejčíř, V, Hynková, O Charakt klientů komerčn. :205. 39. Ricci M, Micheloni GM, Berti M, Perusi F, Sambugaro E, Vecchini E, et al. Clinical comparison of oral administration and viscosupplementation of hyaluronic acid (HA) in early knee osteoarthritis. Musculoskelet Surg. 2017;101(1):45–9. 40. Wang C-T, Lin Y-T, Chiang B-L, Lin Y-H, Hou S-M. High molecular weight hyaluronic acid down-regulates the gene expression of osteoarthritis-associated cytokines and enzymes in fibroblast-like synoviocytes from patients with early osteoarthritis. Osteoarthr Cartil. 2006;14(12):1237–47. 41. Irrgang JJ, Anderson AF. Development and validation of health-related quality of life measures for the knee. Clin Orthop Relat Res. 2002;402:95–109. 42. Roos EM, Roos HP, Lohmander LS, Ekdahl C, Beynnon BD. Knee Injury and Osteoarthritis Outcome Score (KOOS)—development of a self-administered outcome measure. J Orthop Sport Phys Ther. 1998;28(2):88–96. 43. Perruccio A V, Canizares M, Hawker GA, Roos EM, Maillefert JF, Lohmander SL. Comparative Validity and Responsiveness of the HOOS-PS and KOOS-PS to the WOMAC Physical Function Subscale in Total Joint Replacement for Osteoarthritis. 44. Salavati M, Mazaheri M, Negahban H, Sohani SM, Ebrahimian MR, Ebrahimi I, et al. Validation of a Persian-version of Knee injury and Osteoarthritis Outcome Score (KOOS) in Iranians with knee injuries. Osteoarthr Cartil. 2008;16(10):1178–82. 45. Higgins LD, Taylor MK, Park D, Ghodadra N, Marchant M, Pietrobon R, et al. Reliability and validity of the International Knee Documentation Committee (IKDC) subjective knee form. Jt Bone Spine. 2007;74(6):594–9. 46. Kujala UM, Kaprio J, Sarno S. Osteoarthritis of weight bearing joints of lower limbs in former elite male athletes. Bmj. 1994;308(6923):231–4. 47. Salini V, Pantalone A, Vanni D. Arthroscopy and Sport Injuries. Arthrosc Sport Inj. 2016;433–7. 48. Broglio K. Randomization in clinical trials: Permuted blocks and stratification. JAMA - J Am Med Assoc. 2018;319(21):2223–4. 49. Emanuel EJ, Wendler D, Grady C. What makes clinical research ethical? JAMA. 2000 May;283(20):2701–11. )

[49.	Emanuel EJ, Wendler D, Grady C. What makes clinical research ethical? JAMA. 2000 May;283(20):2701–11.](1. Patel DR, Villalobos A. Evaluation and management of knee pain in young athletes: overuse injuries of the knee. Transl Pediatr [Internet]. 2017 Jul;6(3):190–8. Available from: https://pubmed.ncbi.nlm.nih.gov/287950102. Nicolini AP, de Carvalho RT, Matsuda MM, Filho JS, Cohen M. Common injuries in athletes' knee: Experience of a specialized center. Acta Ortop Bras. 2014;22(3):127–31. 3. Tiu T, Craig Van Dien MD. Knee overuse disorders. 4. Fuller CW, Ekstrand J, Junge A, Andersen TE, Bahr R, Dvorak J, et al. Consensus statement on injury definitions and data collection procedures in studies  of football (soccer) injuries. Br J Sports Med. 2006 Mar;40(3):193–201. 5. Majewski M, Susanne H, Klaus S. Epidemiology of athletic knee injuries: A 10-year study. Knee. 2006 Jun;13(3):184–8. 6. Wilder RP, Sethi S. Overuse injuries: tendinopathies, stress fractures, compartment syndrome, and shin  splints. Clin Sports Med. 2004 Jan;23(1):55–81, vi. 7. Aicale R, Tarantino D, Maffulli N. Overuse injuries in sport: a comprehensive overview. J Orthop Surg Res. 2018 Dec;13(1):309. 8. Yang J, Tibbetts AS, Covassin T, Cheng G, Nayar S, Heiden E. Epidemiology of overuse and acute injuries among competitive collegiate athletes. J Athl Train. 2012;47(2):198–204. 9. Bernetti A, Mangone M, Paoloni M, Di Sante L, Murgia M, Santilli V. Corticosteroid and hyaluronic acid injection therapy in tennis elbow (lateral epicondylalgia). Med Sport. 2014;67(2):289–95. 10. Iolascon G, Gimigliano F, Moretti A, De Sire A, Migliore A, Brandi ML, et al. Early osteoarthritis: How to define, diagnose, and manage. A systematic review. Eur Geriatr Med. 2017;8(5–6):383–96. 11. Drawer S, Fuller CW. Propensity for osteoarthritis and lower limb joint pain in retired professional soccer players. Br J Sports Med. 2001;35(6):402–8. 12. Vannini F, Spalding T, Andriolo L, Berruto M, Denti M, Espregueira-Mendes J, et al. Sport and early osteoarthritis: the role of sport in aetiology, progression and treatment of knee osteoarthritis. Knee Surgery, Sport Traumatol Arthrosc. 2016;24(6):1786–96. 13. Fröhlich S, Pazeller S, Cherati AS, Müller E, Frey WO, Spörri J. Overuse injuries in the knee, back and hip of top elite female alpine skiers during  the off-season preparation period: prevalence, severity and their association with traumatic preinjuries and training load. BMJ open Sport Exerc Med. 2020;6(1):e000892. 14. Rincon GA, Vyas D, Zhou J, Fu FH, Oiestad BE, Holm I, et al. \“ Knee function and prevalence of knee osteoarthritis after anterior cruciate ligament reconstruction: a prospective study with 10 to 15 years of follow-up\”. Letter to the editor. Am J Sports Med. 2011;39(4):NP3-author. 15. Kellgren JH, Lawrence J. Radiological assessment of osteo-arthrosis. Ann Rheum Dis. 1957;16(4):494. 16. Altman R, Asch E, Bloch D, Bole G, Borenstein D, Brandt K, et al. Development of criteria for the classification and reporting of osteoarthritis: classification of osteoarthritis of the knee. Arthritis Rheum Off J Am Coll Rheumatol. 1986;29(8):1039–49. 17. Luyten FP, Denti M, Filardo G, Kon E, Engebretsen L. Definition and classification of early osteoarthritis of the knee. Knee Surgery, Sport Traumatol Arthrosc. 2012;20(3):401–6. 18. Hoemann C, Kandel R, Roberts S, Saris DBF, Creemers L, Mainil-Varlet P, et al. International Cartilage Repair Society (ICRS) Recommended Guidelines for  Histological Endpoints for Cartilage Repair Studies in Animal Models and Clinical Trials. Cartilage. 2011 Apr;2(2):153–72. 19. Hunter DJ, Lo GH, Gale D, Grainger AJ, Guermazi A, Conaghan PG. The reliability of a new scoring system for knee osteoarthritis MRI and the validity of bone marrow lesion assessment: BLOKS (Boston–Leeds Osteoarthritis Knee Score). Ann Rheum Dis. 2008;67(2):206–11. 20. Peterfy CG, Guermazi A, Zaim S, Tirman PFJ, Miaux Y, White D, et al. Whole-organ magnetic resonance imaging score (WORMS) of the knee in osteoarthritis. Osteoarthr Cartil. 2004;12(3):177–90. 21. Demange MK, Sisto M, Rodeo S. Future trends for unicompartmental arthritis of the knee: injectables & stem cells. Clin Sports Med. 2014;33(1):161–74. 22. Leslie M. Knee osteoarthritis management therapies. Pain Manag Nurs. 2000;1(2):51–7. 23. Paoloni M, Bernetti A, Belelli A, Brignoli O, Buoso S, Caputi AP, et al. Appropriateness of clinical and organizational criteria for intra-articular injection therapies in osteoarthritis: A Delphi method consensus initiative among experts in Italy. Ann Ist Super Sanita. 2015;51:131–8. 24. Sandmark H, Hogstedt C, Lewold S, Vingård E. Osteoarthrosis of the knee in men and women in association with overweight, smoking, and hormone therapy. Ann Rheum Dis. 1999;58(3):151–5. 25. Christensen BV, Iuhl IU, Vilbek H, Bülow H, Dreijer NC, Rasmussen HF. Acupuncture treatment of severe knee osteoarthrosis. A long‐term study. Acta Anaesthesiol Scand. 1992;36(6):519–25. 26. de Sire A, Agostini F, Lippi L, Mangone M, Marchese S, Cisari C, et al. Oxygen-Ozone Therapy in the Rehabilitation Field: State of the Art on Mechanisms of  Action, Safety and Effectiveness in Patients with Musculoskeletal Disorders. Biomolecules. 2021 Feb;11(3). 27. Fransen M, McCONNELL S. Land-based exercise for osteoarthritis of the knee: a metaanalysis of randomized controlled trials. J Rheumatol. 2009;36(6):1109–17. 28. Hiemstra LA, Kerslake S, Irving C. Anterior knee pain in the athlete. Clin Sports Med. 2014;33(3):437–59. 29. Filardo G, Kon E, Di Matteo B, Pelotti P, Di Martino A, Marcacci M. Platelet-rich plasma for the treatment of patellar tendinopathy: clinical and imaging findings at medium-term follow-up. Int Orthop [Internet]. 2013;37(8):1583–9. Available from: https://doi.org/10.1007/s00264-013-1972-830. Trojian TH, Concoff AL, Joy SM, Hatzenbuehler JR, Saulsberry WJ, Coleman CI. AMSSM Scientific Statement Concerning Viscosupplementation Injections for Knee  Osteoarthritis: Importance for Individual Patient Outcomes. Clin J Sport Med  Off J Can Acad  Sport Med. 2016 Jan;26(1):1–11. 31. Ammar TY, Pereira TAP, Mistura SLL, Kuhn A, Saggin JI, Lopes OV. Viscosupplementation for treating knee osteoarthrosis: review of the literature. Rev Bras Ortop. 2015;50:489–94. 32. Salini V, Pantalone A, Vanni D, Abate M. Viscosupplementation in Athletes. In: Arthroscopy and Sport Injuries. Springer; 2016. p. 433–7. 33. Erdem Y, Gul D, Akpancar S. Comparison of Intraarticular Injections of Hyaluronic Acid versus Dextrose Applied with Periarticular Prolotherapy in the Treatment of Recreational Athletes with Knee Osteoarthritis. Turkish J Sport Med. 2020;55(1):6–13. 34. Kalkhoven J, Coutts AJ, Impellizzeri FM. 'Training load error' is not a more accurate term than 'overuse' injury. Br J Sports Med [Internet]. 2020 Aug 1;54(15):934 LP – 935. Available from: http://bjsm.bmj.com/content/54/15/934.abstract35. Odole AC, Agbomeji OT, Onyeso OKK, Ojo JO, Odunaiya NA. Perspectives of Nigerian Athletes About Physiotherapy Services in Sports Injury Management: Implications for Rehabilitation. J Sport Rehabil. 2021;1(aop):1–8. 36. Hendrika W, Reswari A. The effect of physiotherapy on pain improvement in patients with early knee osteoarthritis at RSU UKI. Int J Med Heal Res. 2021;7(6):52–9. 37. MacKay C, Hawker GA, Jaglal SB. How Do Physical Therapists Approach Management of People With Early Knee Osteoarthritis? A Qualitative Study. Phys Ther [Internet]. 2019;100(2):295–306. Available from: https://doi.org/10.1093/ptj/pzz16438. Hrazdira L, Pilecki Z, Pilecki G. Ultrasound guided application of HA in the soft tissues of the locomotor system in athletes–First experience. Krátká sdělení Botek, M, Stejskal, P, Klimešová, I Vliv normobarické hypoxie na Akt Auton Nerv systému Pilot Stud 191 Drbošalová, V, Stejskal, P, Klugar, M, Krejčíř, V, Hynková, O Charakt klientů komerčn. :205. 39. Ricci M, Micheloni GM, Berti M, Perusi F, Sambugaro E, Vecchini E, et al. Clinical comparison of oral administration and viscosupplementation of hyaluronic acid (HA) in early knee osteoarthritis. Musculoskelet Surg. 2017;101(1):45–9. 40. Wang C-T, Lin Y-T, Chiang B-L, Lin Y-H, Hou S-M. High molecular weight hyaluronic acid down-regulates the gene expression of osteoarthritis-associated cytokines and enzymes in fibroblast-like synoviocytes from patients with early osteoarthritis. Osteoarthr Cartil. 2006;14(12):1237–47. 41. Irrgang JJ, Anderson AF. Development and validation of health-related quality of life measures for the knee. Clin Orthop Relat Res. 2002;402:95–109. 42. Roos EM, Roos HP, Lohmander LS, Ekdahl C, Beynnon BD. Knee Injury and Osteoarthritis Outcome Score (KOOS)—development of a self-administered outcome measure. J Orthop Sport Phys Ther. 1998;28(2):88–96. 43. Perruccio A V, Canizares M, Hawker GA, Roos EM, Maillefert JF, Lohmander SL. Comparative Validity and Responsiveness of the HOOS-PS and KOOS-PS to the WOMAC Physical Function Subscale in Total Joint Replacement for Osteoarthritis. 44. Salavati M, Mazaheri M, Negahban H, Sohani SM, Ebrahimian MR, Ebrahimi I, et al. Validation of a Persian-version of Knee injury and Osteoarthritis Outcome Score (KOOS) in Iranians with knee injuries. Osteoarthr Cartil. 2008;16(10):1178–82. 45. Higgins LD, Taylor MK, Park D, Ghodadra N, Marchant M, Pietrobon R, et al. Reliability and validity of the International Knee Documentation Committee (IKDC) subjective knee form. Jt Bone Spine. 2007;74(6):594–9. 46. Kujala UM, Kaprio J, Sarno S. Osteoarthritis of weight bearing joints of lower limbs in former elite male athletes. Bmj. 1994;308(6923):231–4. 47. Salini V, Pantalone A, Vanni D. Arthroscopy and Sport Injuries. Arthrosc Sport Inj. 2016;433–7. 48. Broglio K. Randomization in clinical trials: Permuted blocks and stratification. JAMA - J Am Med Assoc. 2018;319(21):2223–4. 49. Emanuel EJ, Wendler D, Grady C. What makes clinical research ethical? JAMA. 2000 May;283(20):2701–11. )

APPENDIX 1

APPENDIX 2

Appendix 3

Apendix 3: Data collection sheet

Date sheet colection

Safety and efficacy of hyaluronic acid plus sorbitol in sports man with overuse knee pain.

1.- Participant indetification cardex

Date:________________________

Participant ID:______________________________ Age_____________________

Gender: Male_____	Female______

Afected Side____________Right___	Left______ Both______

Weight___________Kgs	High_________________cms	BMI:______________

2.- Sports Activities:

Activity:____________________________ Elapsed Time since begin this sport_________

Frecuency

_________________days per week	________________minutes per day

3.- Treatment:

INFORMED CONSENT

Principal Reserch: Julio Carlos Vélez de Lachica

Associated Research: Susana Serrano Reyes

Clinical trial: Safefty and effectiveness of hyaluronic acid and sorbitol with overuse knee pain in sportsmen.

The main objective of this clinical research pretend to know the efficacy, safety and clinical benefits of Hialuronic Acid plus Sorbitol, in sportsman with overuse knee syndrome

This clinical trial have the following characteristics:

1.- Research description: This trial pretends to collect knee scoring data for a selected population according to inclusion criteria before and after application of hialuronic acid plus sorbitol in one or both knees.

2.- Risks and discomforts: In any case there may be no clinical difference in their symptoms and they may continue with pain or functional limitation that partially or totally prevents sports activity, also the participant may have anaphylaxis to hyaluronic acid components, knee redness, edema, and temporary residual pain.

3. Expected benefits: No benefit or profit will be obtained by enrolling this study, however its results will be useful for international medical scientific development.

4.-Compensation provided in case of damage: In the event of any adverse effect related to the applied treatment, the effects caused by the infiltration will be monitored and treated.

5.-Economic Remuneration: There is no economic remuneration when enrolling this research. The decision to join this research is completely voluntary and there is the possibility of withdrawing from it at any time without loss of any benefit that could be obtained. Medical assessments, visco-supplementation therapy or sports coaching will have no cost.

6.- Protection of personal data: The researchers will have access to the medical history and clinical file, safeguarding the protection and confidentiality of personal data and at no time will the identity of the patient be exposed in the event of publication of results.

7.- Update of information: During the study, participants will be informed of new information that could be relevant in their decision to continue being part of the study protocol.

8.- Duration of the investigation and data collection: Information related to symptoms and lifestyle will be requested during a period of 6 months of having been part of this investigation. The contact may be in a medical office area, by video call, phone call or email. You will be given an emergency phone number in case you require additional information or assessment to what is scheduled.

9. Number of patients: This research is intended to be carried out on approximately 120 participants.

**I do accept to participate in this clnical trial**

YES	NO

Participant Name:_________________________ Sign:_________________

Date: ______________

Witness:________________________________ Sign:_________________

Date: _____________

Name of clinical research staff:_______________________________

Sign: ____________Date:_________

# References

1.	Patel DR, Villalobos A. Evaluation and management of knee pain in young athletes: overuse injuries of the knee. Transl Pediatr [Internet]. 2017 Jul;6(3):190–8. Available from: https://pubmed.ncbi.nlm.nih.gov/28795010

2.	Nicolini AP, de Carvalho RT, Matsuda MM, Filho JS, Cohen M. Common injuries in athletes’ knee: Experience of a specialized center. Acta Ortop Bras. 2014;22(3):127–31.

3.	Tiu T, Craig Van Dien MD. Knee overuse disorders.

4.	Fuller CW, Ekstrand J, Junge A, Andersen TE, Bahr R, Dvorak J, et al. Consensus statement on injury definitions and data collection procedures in studies of football (soccer) injuries. Br J Sports Med. 2006 Mar;40(3):193–201.

5.	Majewski M, Susanne H, Klaus S. Epidemiology of athletic knee injuries: A 10-year study. Knee. 2006 Jun;13(3):184–8.

6.	Wilder RP, Sethi S. Overuse injuries: tendinopathies, stress fractures, compartment syndrome, and shin splints. Clin Sports Med. 2004 Jan;23(1):55–81, vi.

7.	Aicale R, Tarantino D, Maffulli N. Overuse injuries in sport: a comprehensive overview. J Orthop Surg Res. 2018 Dec;13(1):309.

8.	Yang J, Tibbetts AS, Covassin T, Cheng G, Nayar S, Heiden E. Epidemiology of overuse and acute injuries among competitive collegiate athletes. J Athl Train. 2012;47(2):198–204.

9.	Bernetti A, Mangone M, Paoloni M, Di Sante L, Murgia M, Santilli V. Corticosteroid and hyaluronic acid injection therapy in tennis elbow (lateral epicondylalgia). Med Sport. 2014;67(2):289–95.

10.	Iolascon G, Gimigliano F, Moretti A, De Sire A, Migliore A, Brandi ML, et al. Early osteoarthritis: How to define, diagnose, and manage. A systematic review. Eur Geriatr Med. 2017;8(5–6):383–96.

11.	Drawer S, Fuller CW. Propensity for osteoarthritis and lower limb joint pain in retired professional soccer players. Br J Sports Med. 2001;35(6):402–8.

12.	Vannini F, Spalding T, Andriolo L, Berruto M, Denti M, Espregueira-Mendes J, et al. Sport and early osteoarthritis: the role of sport in aetiology, progression and treatment of knee osteoarthritis. Knee Surgery, Sport Traumatol Arthrosc. 2016;24(6):1786–96.

13.	Fröhlich S, Pazeller S, Cherati AS, Müller E, Frey WO, Spörri J. Overuse injuries in the knee, back and hip of top elite female alpine skiers during the off-season preparation period: prevalence, severity and their association with traumatic preinjuries and training load. BMJ open Sport Exerc Med. 2020;6(1):e000892.

14.	Rincon GA, Vyas D, Zhou J, Fu FH, Oiestad BE, Holm I, et al. “ Knee function and prevalence of knee osteoarthritis after anterior cruciate ligament reconstruction: a prospective study with 10 to 15 years of follow-up”. Letter to the editor. Am J Sports Med. 2011;39(4):NP3-author.

15.	Kellgren JH, Lawrence J. Radiological assessment of osteo-arthrosis. Ann Rheum Dis. 1957;16(4):494.

16.	Altman R, Asch E, Bloch D, Bole G, Borenstein D, Brandt K, et al. Development of criteria for the classification and reporting of osteoarthritis: classification of osteoarthritis of the knee. Arthritis Rheum Off J Am Coll Rheumatol. 1986;29(8):1039–49.

17.	Luyten FP, Denti M, Filardo G, Kon E, Engebretsen L. Definition and classification of early osteoarthritis of the knee. Knee Surgery, Sport Traumatol Arthrosc. 2012;20(3):401–6.

18.	Hoemann C, Kandel R, Roberts S, Saris DBF, Creemers L, Mainil-Varlet P, et al. International Cartilage Repair Society (ICRS) Recommended Guidelines for Histological Endpoints for Cartilage Repair Studies in Animal Models and Clinical Trials. Cartilage. 2011 Apr;2(2):153–72.

19.	Hunter DJ, Lo GH, Gale D, Grainger AJ, Guermazi A, Conaghan PG. The reliability of a new scoring system for knee osteoarthritis MRI and the validity of bone marrow lesion assessment: BLOKS (Boston–Leeds Osteoarthritis Knee Score). Ann Rheum Dis. 2008;67(2):206–11.

20.	Peterfy CG, Guermazi A, Zaim S, Tirman PFJ, Miaux Y, White D, et al. Whole-organ magnetic resonance imaging score (WORMS) of the knee in osteoarthritis. Osteoarthr Cartil. 2004;12(3):177–90.

21.	Demange MK, Sisto M, Rodeo S. Future trends for unicompartmental arthritis of the knee: injectables & stem cells. Clin Sports Med. 2014;33(1):161–74.

22.	Leslie M. Knee osteoarthritis management therapies. Pain Manag Nurs. 2000;1(2):51–7.

23.	Paoloni M, Bernetti A, Belelli A, Brignoli O, Buoso S, Caputi AP, et al. Appropriateness of clinical and organizational criteria for intra-articular injection therapies in osteoarthritis: A Delphi method consensus initiative among experts in Italy. Ann Ist Super Sanita. 2015;51:131–8.

24.	Sandmark H, Hogstedt C, Lewold S, Vingård E. Osteoarthrosis of the knee in men and women in association with overweight, smoking, and hormone therapy. Ann Rheum Dis. 1999;58(3):151–5.

25.	Christensen BV, Iuhl IU, Vilbek H, Bülow H, Dreijer NC, Rasmussen HF. Acupuncture treatment of severe knee osteoarthrosis. A long‐term study. Acta Anaesthesiol Scand. 1992;36(6):519–25.

26.	de Sire A, Agostini F, Lippi L, Mangone M, Marchese S, Cisari C, et al. Oxygen-Ozone Therapy in the Rehabilitation Field: State of the Art on Mechanisms of Action, Safety and Effectiveness in Patients with Musculoskeletal Disorders. Biomolecules. 2021 Feb;11(3).

27.	Fransen M, McCONNELL S. Land-based exercise for osteoarthritis of the knee: a metaanalysis of randomized controlled trials. J Rheumatol. 2009;36(6):1109–17.

28.	Hiemstra LA, Kerslake S, Irving C. Anterior knee pain in the athlete. Clin Sports Med. 2014;33(3):437–59.

29.	Filardo G, Kon E, Di Matteo B, Pelotti P, Di Martino A, Marcacci M. Platelet-rich plasma for the treatment of patellar tendinopathy: clinical and imaging findings at medium-term follow-up. Int Orthop [Internet]. 2013;37(8):1583–9. Available from: https://doi.org/10.1007/s00264-013-1972-8

30.	Trojian TH, Concoff AL, Joy SM, Hatzenbuehler JR, Saulsberry WJ, Coleman CI. AMSSM Scientific Statement Concerning Viscosupplementation Injections for Knee Osteoarthritis: Importance for Individual Patient Outcomes. Clin J Sport Med Off J Can Acad Sport Med. 2016 Jan;26(1):1–11.

31.	Ammar TY, Pereira TAP, Mistura SLL, Kuhn A, Saggin JI, Lopes OV. Viscosupplementation for treating knee osteoarthrosis: review of the literature. Rev Bras Ortop. 2015;50:489–94.

32.	Salini V, Pantalone A, Vanni D, Abate M. Viscosupplementation in Athletes. In: Arthroscopy and Sport Injuries. Springer; 2016. p. 433–7.

33.	Erdem Y, Gul D, Akpancar S. Comparison of Intraarticular Injections of Hyaluronic Acid versus Dextrose Applied with Periarticular Prolotherapy in the Treatment of Recreational Athletes with Knee Osteoarthritis. Turkish J Sport Med. 2020;55(1):6–13.

34.	Kalkhoven J, Coutts AJ, Impellizzeri FM. ‘Training load error’ is not a more accurate term than ‘overuse’ injury. Br J Sports Med [Internet]. 2020 Aug 1;54(15):934 LP – 935. Available from: http://bjsm.bmj.com/content/54/15/934.abstract

35.	Odole AC, Agbomeji OT, Onyeso OKK, Ojo JO, Odunaiya NA. Perspectives of Nigerian Athletes About Physiotherapy Services in Sports Injury Management: Implications for Rehabilitation. J Sport Rehabil. 2021;1(aop):1–8.

36.	Hendrika W, Reswari A. The effect of physiotherapy on pain improvement in patients with early knee osteoarthritis at RSU UKI. Int J Med Heal Res. 2021;7(6):52–9.

37.	MacKay C, Hawker GA, Jaglal SB. How Do Physical Therapists Approach Management of People With Early Knee Osteoarthritis? A Qualitative Study. Phys Ther [Internet]. 2019;100(2):295–306. Available from: https://doi.org/10.1093/ptj/pzz164

38.	Hrazdira L, Pilecki Z, Pilecki G. Ultrasound guided application of HA in the soft tissues of the locomotor system in athletes–First experience. Krátká sdělení Botek, M, Stejskal, P, Klimešová, I Vliv normobarické hypoxie na Akt Auton Nerv systému Pilot Stud 191 Drbošalová, V, Stejskal, P, Klugar, M, Krejčíř, V, Hynková, O Charakt klientů komerčn. :205.

39.	Ricci M, Micheloni GM, Berti M, Perusi F, Sambugaro E, Vecchini E, et al. Clinical comparison of oral administration and viscosupplementation of hyaluronic acid (HA) in early knee osteoarthritis. Musculoskelet Surg. 2017;101(1):45–9.

40.	Wang C-T, Lin Y-T, Chiang B-L, Lin Y-H, Hou S-M. High molecular weight hyaluronic acid down-regulates the gene expression of osteoarthritis-associated cytokines and enzymes in fibroblast-like synoviocytes from patients with early osteoarthritis. Osteoarthr Cartil. 2006;14(12):1237–47.

41.	Irrgang JJ, Anderson AF. Development and validation of health-related quality of life measures for the knee. Clin Orthop Relat Res. 2002;402:95–109.

42.	Roos EM, Roos HP, Lohmander LS, Ekdahl C, Beynnon BD. Knee Injury and Osteoarthritis Outcome Score (KOOS)—development of a self-administered outcome measure. J Orthop Sport Phys Ther. 1998;28(2):88–96.

43.	Perruccio A V, Canizares M, Hawker GA, Roos EM, Maillefert JF, Lohmander SL. Comparative Validity and Responsiveness of the HOOS-PS and KOOS-PS to the WOMAC Physical Function Subscale in Total Joint Replacement for Osteoarthritis.

44.	Salavati M, Mazaheri M, Negahban H, Sohani SM, Ebrahimian MR, Ebrahimi I, et al. Validation of a Persian-version of Knee injury and Osteoarthritis Outcome Score (KOOS) in Iranians with knee injuries. Osteoarthr Cartil. 2008;16(10):1178–82.

45.	Higgins LD, Taylor MK, Park D, Ghodadra N, Marchant M, Pietrobon R, et al. Reliability and validity of the International Knee Documentation Committee (IKDC) subjective knee form. Jt Bone Spine. 2007;74(6):594–9.

46.	Kujala UM, Kaprio J, Sarno S. Osteoarthritis of weight bearing joints of lower limbs in former elite male athletes. Bmj. 1994;308(6923):231–4.

47.	Salini V, Pantalone A, Vanni D. Arthroscopy and Sport Injuries. Arthrosc Sport Inj. 2016;433–7.

48.	Broglio K. Randomization in clinical trials: Permuted blocks and stratification. JAMA - J Am Med Assoc. 2018;319(21):2223–4.

49.	Emanuel EJ, Wendler D, Grady C. What makes clinical research ethical? JAMA. 2000 May;283(20):2701–11.
