# Supplementary material for: Clinical outcomes of hyaluronic acid–sorbitol injection in athletes with patellofemoral pain: Evaluation of the potential influence of body mass index
Source: PLoS One. 2026 Sep 30;21(9):e0346269. doi: 10.1371/journal.pone.0346269 (PMC13626342; doi:10.1371/journal.pone.0346269)
Supplement: Supplementary file 2 [file pone.0346269.s003.docx]

TREND Statement Checklist

*Manuscript: Clinical outcomes of hyaluronic acid–sorbitol injection in athletes with patellofemoral pain: Evaluation of the potential influence of body mass index (PONE-D-26-06678R3)*

Checklist adapted from Des Jarlais DC, Lyles C, Crepaz N, and the TREND Group. Am J Public Health. 2004;94:361-366 (CDC TREND Statement, public domain).

| **Item #** | **TREND Section** | **Item Description** | **Reported?** | **Manuscript location** |
| --- | --- | --- | --- | --- |
| 1 | Title and Abstract | Information on how units were allocated to interventions; structured abstract describing target population, sample size, intervention/exposure, outcomes, results, and conclusions. | Reported | Title page; Abstract (single-arm, non-randomized, n=60 analyzed; IKDC/KOOS outcomes at 6 months) |
| 2 | Introduction / Background | Scientific background and explanation of rationale. | Reported | Introduction |
| 3 | Methods / Participants | Eligibility criteria for participants; methods of recruitment; settings and locations of data collection. | Reported | Methods > Participants (age 30–55, Tegner-classified athletes; ISSEMyM Tlalnepantla, Feb 2023–Oct 2024) |
| 4 | Methods / Interventions | Details of interventions: content, delivery, unit of delivery, deliverer, setting, exposure quantity/duration, time span, adherence/compliance. | Reported | Methods > Procedures (single 4 mL HA-sorbitol intra-articular injection; single injection-prevention education session) |
| 5 | Objectives | Specific objectives and hypotheses. | Reported | Introduction (final paragraph, study aims); Abstract > Objective |
| 6 | Outcomes | Clearly defined primary/secondary outcomes; methods for assessing them; instrument reliability/validity if applicable. | Reported | Methods > Procedures and Statistical analysis (IKDC, KOOS at baseline, 15 days, 3 and 6 months) |
| 7 | Sample Size | How sample size was determined; any interim analyses and stopping rules. | Reported | Methods > Participants (a priori minimum n=58; 64 enrolled to allow for attrition; no interim analyses/stopping rule, fixed follow-up design) |
| 8 | Assignment Method | Unit of assignment; method used to assign units to intervention (masking, randomization details). | Not applicable | Methods > Study design and ethics — single-arm design, all participants received the same intervention; no allocation/randomization performed |
| 9 | Blinding | Whether participants, providers, and outcome assessors were blinded; if so, how; if not, why not. | Reported | Methods > Study design/Procedures — open-label, single-arm study; blinding not applicable given fixed single intervention (noted as a limitation) |
| 10 | Unit of Analysis | Description of the smallest unit analyzed; if different from unit of assignment, description of the method used to account for this. | Reported | Methods > Statistical analysis — patient-level data; unit of assignment = unit of analysis (individual participant) |
| 11 | Statistical Methods | Statistical methods used to compare groups/assess change; methods for handling missing data; multiplicity control. | Reported | Methods > Statistical analysis (LMM with GEE sensitivity analysis; complete-case analysis, no missing IKDC/KOOS values among 60 completers) |
| 12 | Participant Flow | Flow of participants through each stage (diagram recommended): numbers assessed/eligible, enrolled, receiving intervention, completing follow-up, analyzed. | Reported | Methods > Participants; Fig 1 (CONSORT/TREND-style flow diagram): 64 enrolled → 4 excluded during follow-up → 60 analyzed |
| 13 | Recruitment | Dates defining periods of recruitment and follow-up. | Reported | Methods > Study design and ethics (enrollment 1 Feb 2023–30 Oct 2024; final follow-up 30 Apr 2025) |
| 14 | Baseline Data | Baseline demographic and clinical characteristics of participants. | Reported | Results > Study population and baseline characteristics; Table 1 |
| 15 | Numbers Analyzed | Number of participants in each analysis; whether analysis was by original assigned group (ITT-equivalent for single-arm). | Reported | Results > Study population and baseline characteristics (n=60 complete-case analysis) |
| 16 | Outcomes and Estimation | For each outcome, results with effect size and precision (e.g., 95% CI). | Reported | Results (Tables 2–5; +18.58 pts IKDC, 95% CI 16.81–20.36; +19.28 pts KOOS, 95% CI 17.52–21.04) |
| 17 | Ancillary Analyses | Other analyses performed, including subgroup and adjusted analyses, indicating which were pre-specified and which exploratory. | Reported | Results > Exploratory BMI subgroup analysis; explicitly labeled exploratory throughout Results and Discussion |
| 18 | Adverse Events | All important adverse events or side effects for each intervention group. | Reported | Results > Safety and adverse events (4/60 minor local events; none serious; none led to withdrawal) |
| 19 | Interpretation | Interpretation of results considering hypotheses, sources of bias/imprecision, and multiplicity of analyses. | Reported | Discussion > Principal findings; Interpretation of longitudinal improvement and TREND bias considerations; Limitations |
| 20 | Generalizability | Generalizability (external validity) of the findings. | Reported | Discussion > Limitations; Future directions |
| 21 | Overall Evidence | General interpretation of results in the context of current evidence. | Reported | Discussion > Biological plausibility and potential mechanisms; Future directions |
| 22 | Ethics and Registration | Ethical approval, informed consent procedures, trial/study registration, and funding/COI statements. | Reported | Methods > Study design and ethics (CEI/0504/PI/2022; ClinicalTrials.gov NCT05450458); Acknowledgments/Conflict of Interest |
